# Multi-omics analysis reveals epithelial-mesenchymal transition-related gene FOXM1 as a novel prognostic biomarker in clear cell renal carcinoma

**DOI:** 10.18632/aging.102459

**Published:** 2019-11-19

**Authors:** Jing Song, Fangzhou Song, Kun Liu, Wanfeng Zhang, Ruihan Luo, Yongyao Tang, Longke Ran

**Affiliations:** 1 Department of Bioinformatics, The Basic Medical School of Chongqing Medical University, Chongqing 400016, China; 2 Molecular and Tumor Research Center, Chongqing Medical University, Chongqing 400016, China

**Keywords:** multi-omics, epithelial-mesenchymal transition, clear cell renal carcinoma, FOXM1

## Abstract

Identification of novel clinical biomarker in clear cell renal carcinoma (ccRCC) is warranted. Integrating transcriptome (n=1669), DNA methylation (n=577) and copy number data (n=832), we developed a method to identify driver biomarkers by analyzing the omics-level dynamics of Epithelial-Mesenchymal Transition (EMT)-related genes in ccRCC. We first identified 504 expression dynamic changed genes involved in ccRCC-associated key pathways such as EMT, cell cycle, EGFR and PI3K/AKT signaling. Further analysis identified 229 (90 gene promoters) aberrant expression quantitative trait methylation (eQTM) and 256 genes with expression quantitative trait copy number (eQTCN) alterations. Among them, FOXM1 was affected by both eQTM and eQTCN. FOXM1 copy number amplification (115/500, 23% of patients), occurred in an amplified peak in chromosome 12q13.3, was enriched in late-stage ccRCC samples and was associated with worse survival. FOXM1-overexpressed pT3 patients with distant metastasis showed ~25% shorter overall survival in both training (log-rank P=0.006) and validation (log-rank P=0.018) cohorts. The eQTM-gene hybrid signature (cg00044170 and FOXM1), superior to either gene expression or DNA methylation alone, showed great potential in diagnosing localized ccRCC in training (area under curve = 0.958) and validation datasets. FOXM1 could be a novel prognostic biomarker and shed light for early diagnosis at molecular level in ccRCC.

## INTRODUCTION

In 2018, approximately 403,000 new cases of kidney cancer were diagnosed worldwide, with >43% patients succumbing to the disease [1]. Renal cell carcinoma (RCC) is the most common type of kidney cancer [2], while the most common histologic subtype of RCC is clear cell RCC (ccRCC) [3]. Patients achieved 5-year survival >90% only if they were diagnosed with early and localized kidney cancer, which is defined as patients with pT1/pT2 disease but without regional lymph node metastasis nor distant metastasis (stage I/II, American Joint Committee on Cancer 8^th^ edition) [4]. 5-year survival rate drops to 12% for patients with distant metastasis [5]. However, only about 65% of patients were diagnosed with localized disease [5]. Thus, improving early diagnostic rate is beneficial for patient survival. Currently, specific prognostic biomarkers and classification hallmarks for advanced ccRCC is still lack and has largely contributed to the poor outcome. The advanced ccRCC is usually characterized by highly invasiveness, regional and distant metastasis, and postsurgical relapse [6, 7]. Therefore, systematic identification of the driving regulators in progression of ccRCC is crucial and valuable.

Epithelial-mesenchymal transition (EMT), first recognized as a crucial process of embryogenesis in the 1980s, allows polarized epithelial cells lose their adhesion and gain migratory and invasive properties of highly mobile mesenchymal cells [8]. EMT can be activated by many genes (e.g. Zeb1/2, Twist1/2 and Snail1/2) through inhibiting CDH1 and/or activating the hallmarks (N-cadherin, vimentin [VIM] and fibronectin) of mesenchymal-epithelial transition [9]. Loss of E-cadherin (CDH1) is considered as the basic event of EMT activation [9]. It has been revealed that EMT plays important roles in invasion, drug resistance, recurrence and initiation of cancer metastasis [9, 10]. Thus, systematic analysis of EMT-related genes may contribute to identification of prognostic marker for advanced ccRCC.

**Figure 1:**
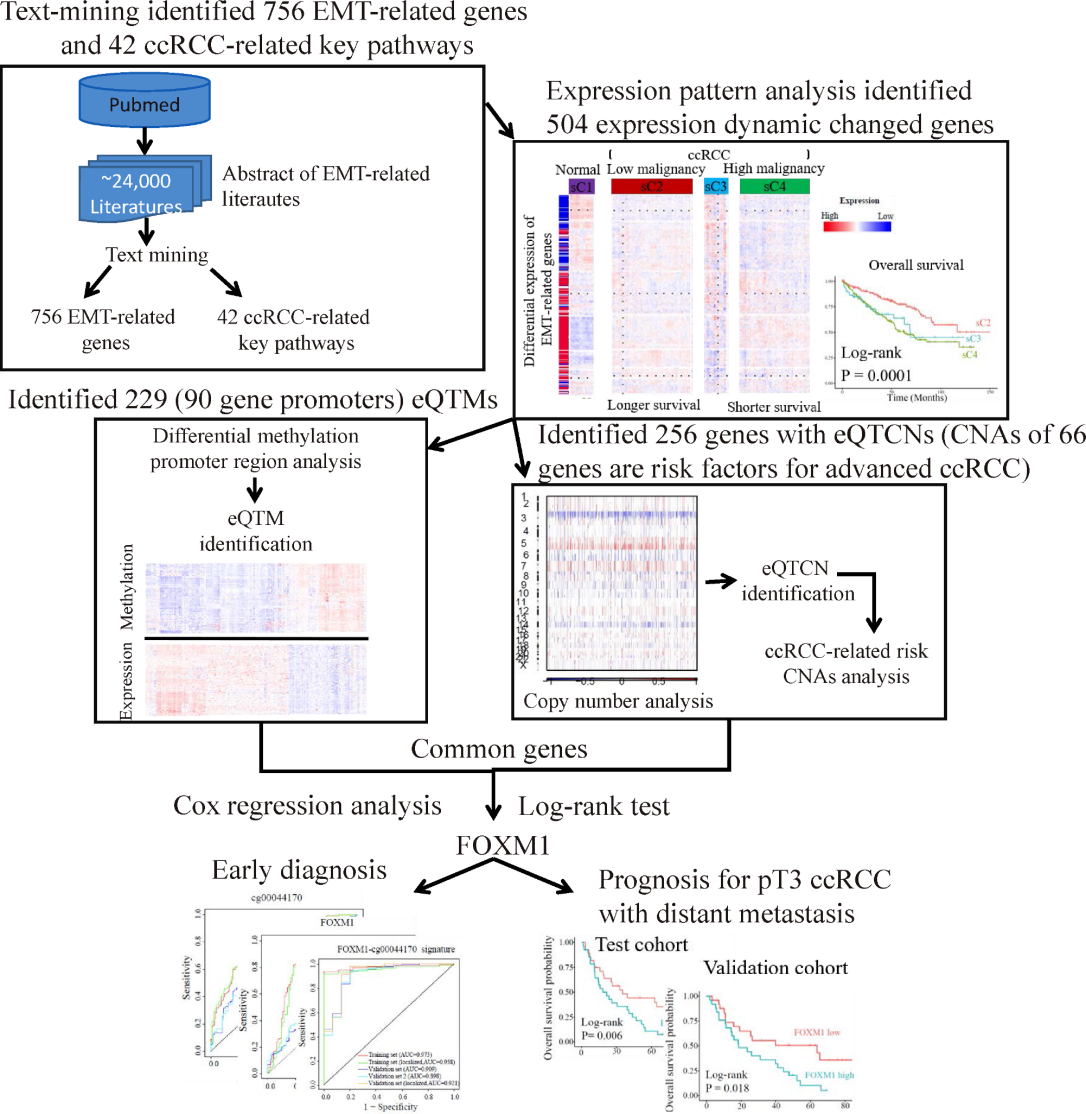
**The flowchart of strategy to identify EMT-related biomarkers in ccRCC.** Firstly, text-mining of abstract of literatures associated with EMT from PubMed database identified 756 EMT-related genes and 42 ccRCC-related key pathways. Secondly, expression pattern analysis of EMT-related gene identified two main tumor clusters differ in tumor malignancy and survival. A total of 504 dynamic expression changed genes among normal controls and the two tumor clusters were identified as key genes, which may be critical in ccRCC. Further analysis identified 229 eQTMs located in 90 gene promoters and 256 gene with eQTCNs by integrating transcriptome, DNA methylation and copy number alteration (CNA) data. Finally, ccRCC-related CNAs calling analysis and survival analysis revealed FOXM1 was a driver gene, which could be a biomarker for early diagnosis and overall prognosis in ccRCC.

Cancer driver genes are the crucial nodes of signaling pathways and regulatory networks. Identification of driver genes in cancer may contribute to personalized therapy, subtype classification, clinical diagnosis and prognosis [11]. Integrating transcriptome, DNA methylation and copy number alteration data of same subjects is especially useful for identifying driver genes that perturbed by diverse factors, but remains challenge [12]. In this study, from the perspective of multi-omics, we identified driver genes in ccRCC by investigation of the information underlies the dynamic changes of EMT-related genes (Figure 1).

## RESULTS

### EMT-related genes play critical roles in ccRCC

**Figure 2:**
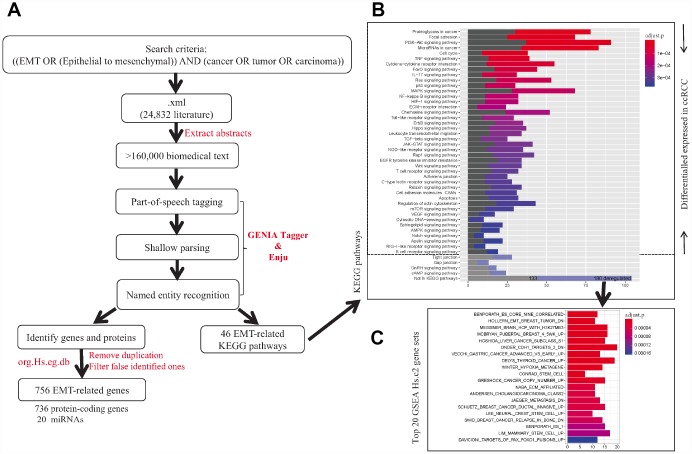
**The workflow of identification of EMT-related genes and pathways in cancers.** (**A**) Text-mining of literatures associated with EMT from PubMed database. (**B**) KEGG pathway enrichment of identified EMT-related genes. (**C**) Gene set enrichment analysis for EMT-related genes not included in KEGG pathway using Molecular Signature Database Hs.c2 gene sets.

We first identified EMT-related genes and pathways by text mining from literatures across cancer types (Figure 2A). A total of 20 miRNAs and 736 protein-coding genes were identified (Supplementary Table 1), and they were enriched in 46 signaling pathways (FDR <0.05). Nine pathways were widely known as EMT-inducer pathways, namely PI3K-AKT, Ras, MAPK, NF-kappaB, Hippo, TGF-beta, JAK-STAT, Wnt and Notch. Among the 756 EMT-related genes, 474 genes involved in 42 KEGG pathways (FDR <0.05, Figure 2B) were dysregulated in ccRCC (FDR <0.05), suggesting the important roles of EMT and EMT-related genes in ccRCC. Gene Set Enrichment Analysis [13] based on Molecular Signatures Database Hs.c2 curated gene sets showed that 180 genes were closely associated with EMT signatures (FDR < 0.05, Figure 2C), such as ‘HOLLERN_EMT_BREAST_TUMOR_DN’ and ‘ONDER_CDH1_TARGETS_2_DN’. Taken together, identified 756 genes were widely involved in EMT-associated pathways and their dysregulation may contribute to the progression of tumors through downstream pathways.

### Gene expression patterns reveal pathway dynamics associated with progression of ccRCC

**Figure 3:**
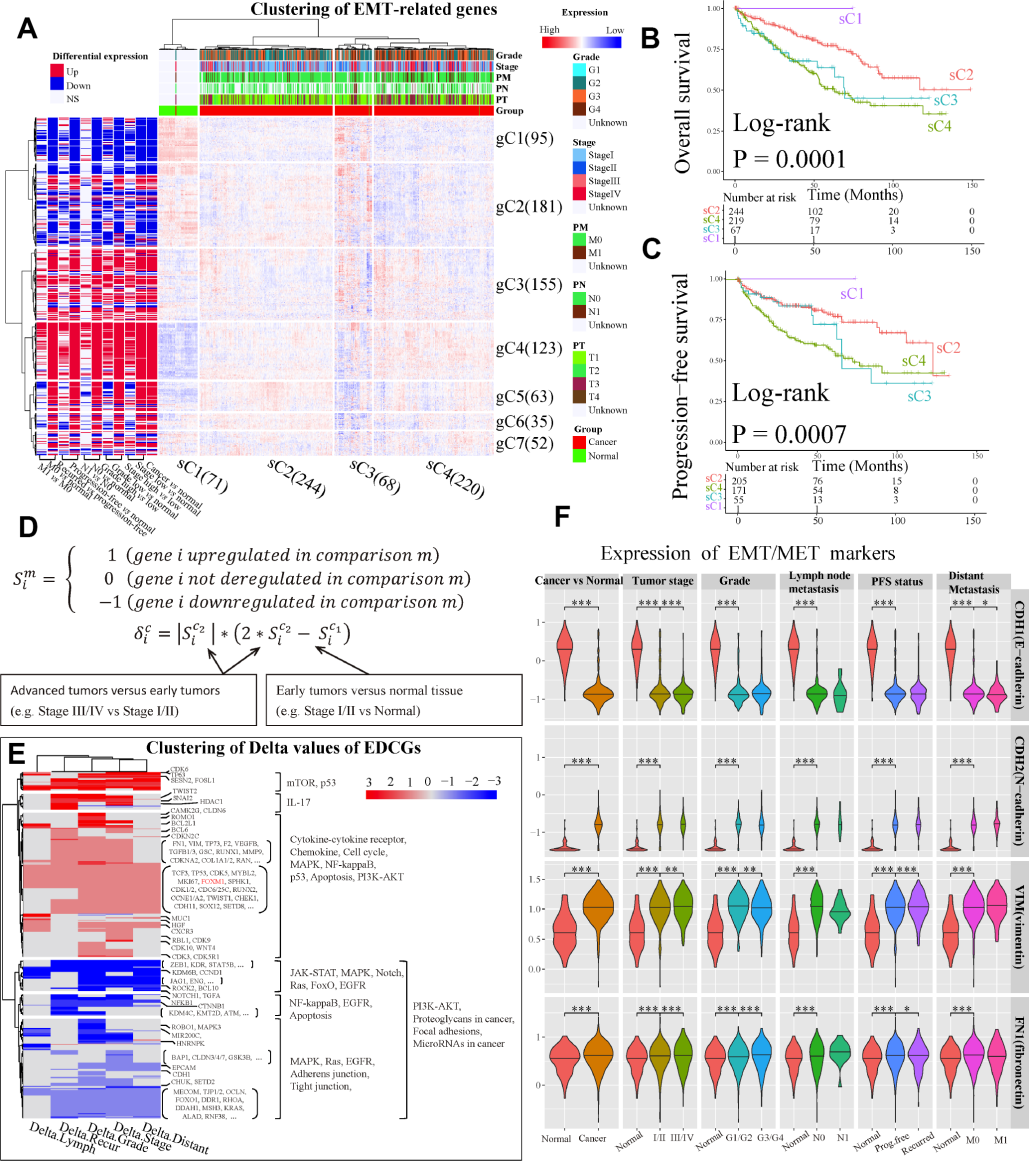
**Analysis of expression dynamic changed genes (EDCGs).** (**A**) Unsupervised hierarchical clustering for patients with ccRCC (n = 603) based on 756 EMT-related genes. The log2 count per million were used. The samples (x-axis) and genes (y-axis) were clustered into four clusters (sC1 to sC4) and seven clusters (gC1 to gC7), respectively. (**B**) Overall survival analysis for sample clusters. (**C**) Progression-free survival analysis for sample clusters. (**D**) Definition of EDCGs and calculation of the degree of expression change (Delta [δ]) for specific gene. (**E**) Clustering of Delta values of EDCGs for stage, grade, lymph node metastasis, distant metastasis and recurrence. (**F**) The expression of EMT/MET markers in ccRCC. Only differentially expressed in at least two out of three datasets were considered statistical significance. * P <0.05, ** P <0.01, *** P <0.001.

Unsupervised hierarchical clustering of ccRCC samples based on Log2 transformed count per million expression levels of 704 genes detected from GDC RNA-Seq dataset showed four clusters (sC1 to sC4) (Figure 3A). The delta area under cumulative distribution functions was remarkably increased when the number of clusters was set at k=4 compared to k<4, however it did not show significant increase with the continue increase of k value (Supplementary Figure 1A and 1B). Most tumor samples were clustered together (Figure 3A). Among the tumor samples, two clusters sC2 (n=244) and sC4 (n=220) dominated the directions of dysregulation of genes in the whole panel, which including >98% patients with high-grade (G3/G4) or high-stage (stage III/IV) disease. Furthermore, the number of patients with stage III/IV (χ^2^ test, P = 2.70e-06), G3/G4 (χ^2^ test, P = 8.74e-07), higher pathological primary tumor stage (T3/T4, χ^2^ test, P = 3.06e-05), invasive regional lymph nodes (N1, χ^2^ test, P = 0.002) and distant metastasis (M1, χ^2^ test, P = 0.002) in sC4 were significantly larger than those in sC2 (Figure 3A). In addition, the overall survival (OS) and progression-free survival (PFS) of patients in sC2 were better than those in sC4 (Figure 3B and 3C). The middle cluster sC3 (n=68, 12.8% of tumors) was composed of 23.5% (16/68) tumors of stage III /IV and 76.5% (52/68) tumors of stage I/II, which were the tumors with greatest expression differences. Cluster sC3 showed a closer relationship with cluster sC4 rather than cluster sC2, suggesting that sC3 might be a small sub-population of tumors that are on the verge of tumor progression. Taken together, the expression pattern of EMT-related genes implied that most of EMT-related genes may undergo second-time dysregulation, and contribute to ccRCC progression.

Seven gene clusters (Supplementary Figure 1C and 1D) were determined using the same methods. It was observed that 644 of 704 EMT-related genes (85.2%) were dysregulated in at least one grouping method (see Materials and Methods) in ccRCC, and the majority of them were up-regulated (gC3 to gC7, Figure 3A). Moreover, by using 11 grouping methods, a great number of genes such as those in gC3 and gC4 were up-regulated, and many genes in gC1 were down-regulated. Interestingly, the gene expression levels in early ccRCC were significantly changed (BH-adjusted P <0.05 in at least two datasets) compared to normal tissues. However, the expression levels of these genes were completely reversed in advanced tumors compared to early ones, such as genes in gC2, gC5 and gC6 (Figure 3A). These results highlight the importance of focusing on the dynamic changes of EMT-related genes in ccRCC tumors progression.

Furthermore, we encoded the expression status (S) of genes and defined their genes expression changes using Delta values (Figure 3D). The genes with Delta value not equal to 0 were defined as expression dynamic changed genes (EDCGs). A total of 504 EDCGs were identified, the expression levels of 145 EDCGs (|Delta| = 3) were reversed in both tumorigenesis and progression of ccRCC. Clustering of Delta values of EDCGs using Euclidean as distance metric with Ward linkage resulted five gene clusters (Figure 3E). The pathways of EDCGs involved may be affected and underwent dynamic changes, such as JAK-STAT, NF-kappaB and PI3K-AKT, Ras, MAPK and cell cycle (Figure 3E). However, the dynamic change analysis of EMT markers showed that expression of CDH1 was sustained downregulated during ccRCC progression, while the expressions of N-cadherin, VIM and fibronectin were upregulated consistently (Figure 3F), suggesting that the EMT was activated during both tumorigenesis and ccRCC progression.

### DNA methylation mediated deregulation of EDCGs in early ccRCC

**Figure 4:**
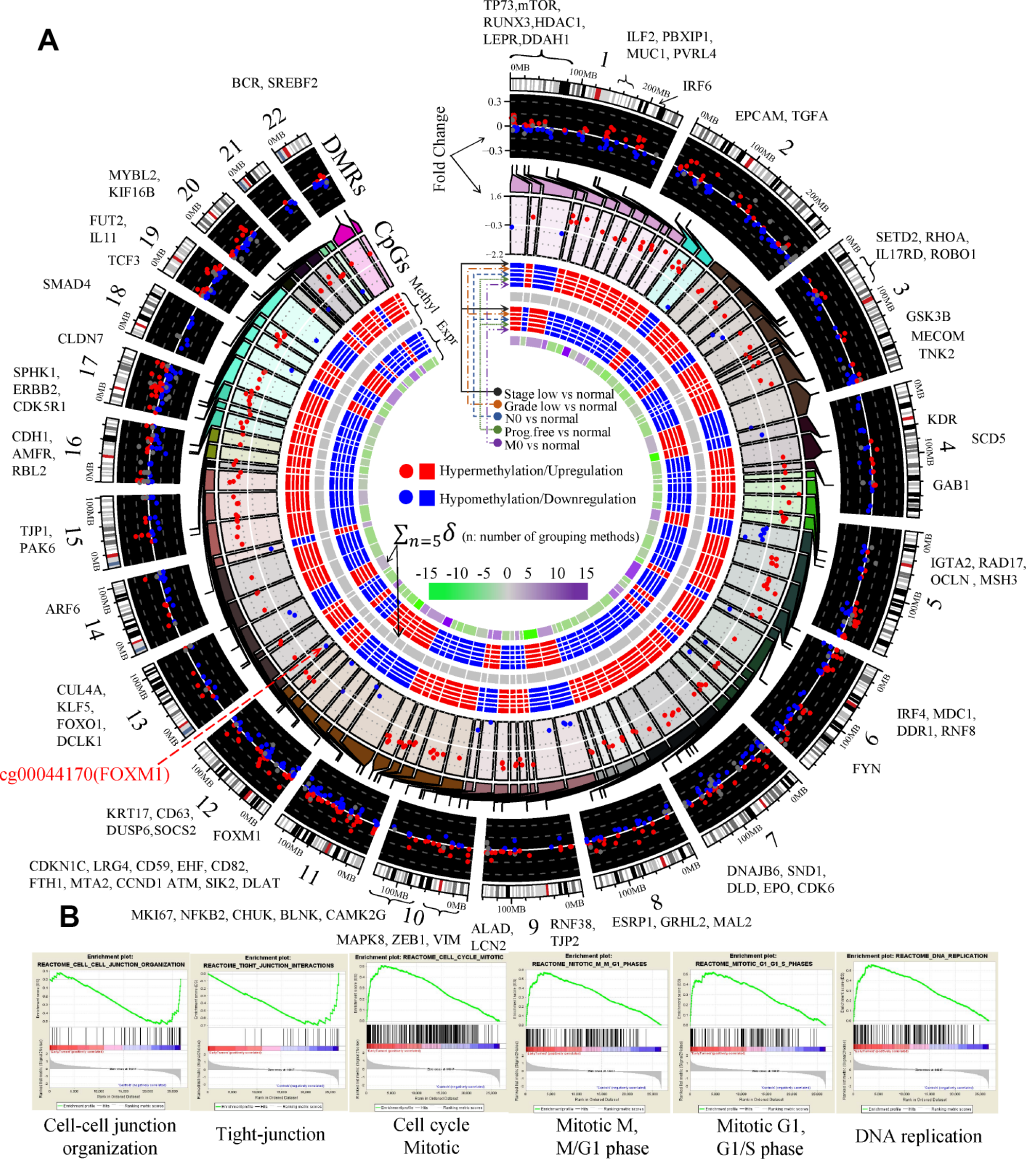
**Aberrant DNA methylation of EDCGs affected their expression in ccRCC.** (**A**) The landscape of differentially methylated regions (DMRs) and expression quantitative trait methylations (eQTMs) in human genome (hg19). 186 DMRs not in promoter were shown using gray dots. The red symbols (both circle and square) represent the higher status (either a higher status of DNA methylation or a higher status of expression), while the blue symbols represent the lower/opposite status. (**B**) Gene set enrichment analysis between localized ccRCC and controls.

To investigate the potential factors of the phased behaviors of the EDCGs, we analyzed the DNA methylation levels and CNAs. Firstly, 13629 CpGs of 504 EDCGs were extracted for differential methylation analysis and 6745 differentially methylated CpGs and 1020 DMRs were identified (FDR <0.05) between tumor and normal. The eQTMs analysis identified 229 CpG-gene pairs with negative correlations (FDR<0.05) and they were located in promoter regions of 90 genes (Figure 4A). The expression of 44 genes were associated with multiple CpGs, such as the EMT marker CDH1 (hypermethylation) and ZEB1 (hypomethylation), while others (46 genes) were affected by single CpG, such as FOXM1 (cg00044170, hypomethylation, Figure 4A) and VIM (hypomethylation). Given that the abnormal methylation of eQTMs is associated with the expression change of EDCGs, we investigated their DNA methylation patterns in ccRCC based on eleven grouping methods (see Materials and Methods). Interestingly, significant differential methylation events of the 229 CpGs were only observed between early tumors and normal tissue (FDR <0.05), but not in advanced tumors compared to early ones after adjustment for multiple tests (FDR >0.05, Figure 4A). Same results were obtained even if all CpGs of EDCGs were included for another independent testing (FDR >0.05). These results suggested that the differential methylation of EDCGs were more likely to associate with tumorigenesis of ccRCC, rather than its progression.

Gene set enrichment analysis results showed that hypermethylated eQTMs involved (e.g., CDH1 and CLDN7) in cell junction organization and tight junction were downregulated (FDR < 0.25, Figure 4B). Hypomethylated eQTMs involved (e.g., CCND1, BUB1 and FOXM1) in G1 phase, G1/S phase, cell cycle mitotic and DNA replication were upregulated. The hypermethylation of CDH1 and hypomethylation of its transcriptional repressors (such as ZEB1 and TCF3) were consistent with EMT activation (FDR < 0.25, Figure 4B). GO analysis also showed that the EDCGs affected by hypomethylated eQTMs were mainly involved in cell cycle (10 genes, FDR < 0.05, Supplementary Figure 2A). Furthermore, three hypomethylated EDCGs (FOXM1, TP73, MYBL2) affected by eQTMs also played roles in regulating gene transcription through RNA polymerase II regulatory regions sequence-specific DNA binding (FDR < 0.05, Supplementary Figure 2A).

### FOXM1 CNAs may be critical events in advanced ccRCC and associated with survival

**Figure 5:**
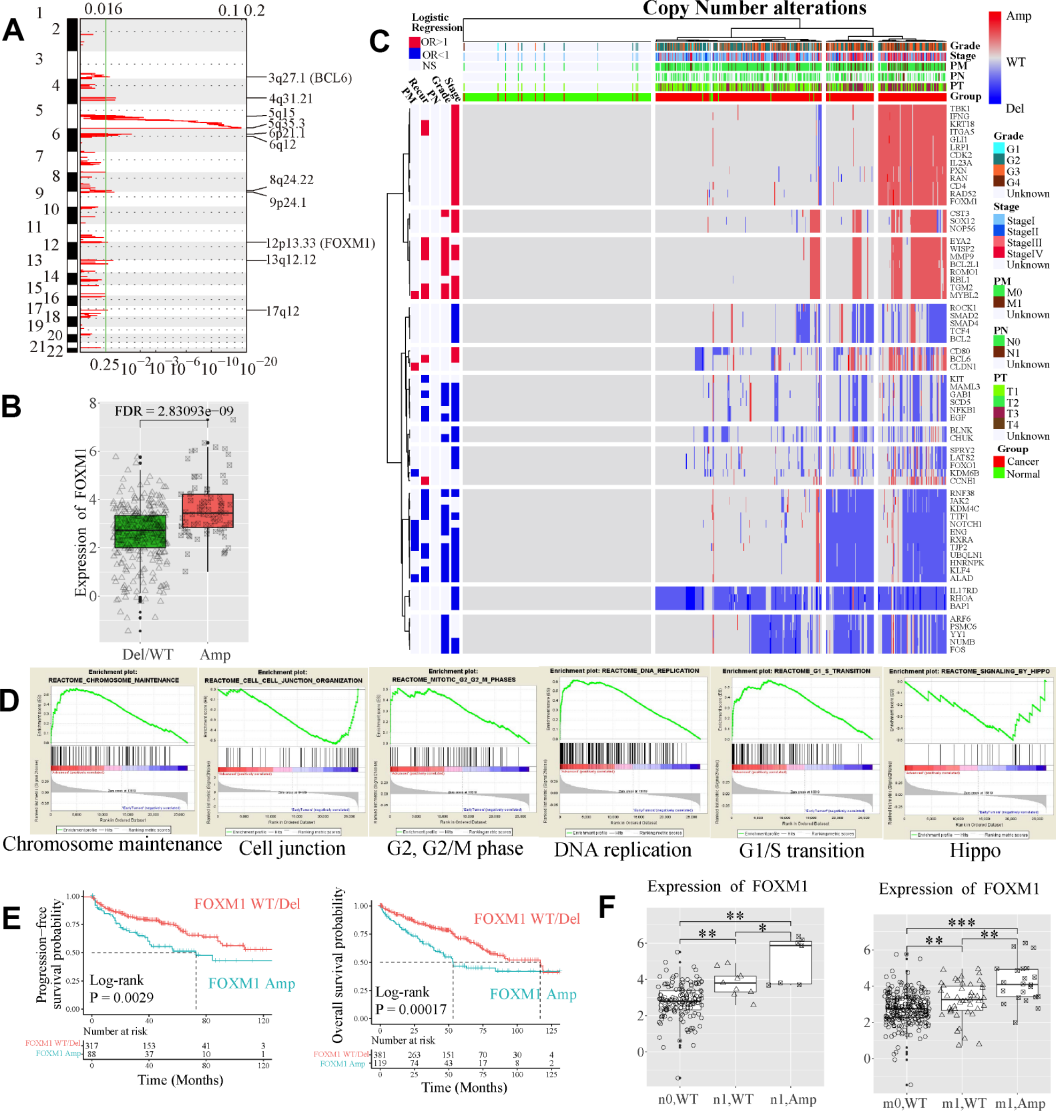
**Copy number (CN) alterations of EDCGs affected their expression in advanced ccRCC.** (**A**) The EDCGs located in the focal CN amplification peaks. False-discovery rates (q values) and scores generated by GISTIC 2.0 for amplifications (x-axis) are plotted against chromosome locations (y-axis). Dotted lines indicate the centromeres. The green line represents cut-off (q = 0.25) that determines statistical significance. (**B**) The expression of FOXM1 affected by expression quantitative trait CN (eQTCNs). (**C**) Clustering of CNAs of genes affected by eQTCNs. The deletion of genes with odds ratio (OR) <1 and FDR <0.05 was associated with advanced tumors. The amplification of genes with OR >1 and FDR <0.05 was related to advanced tumors. (**D**) Gene set enrichment analysis between ccRCC with regional/distant metastasis and localized tumors. (**E**) Overall survival and progression-free survival of ccRCC patients with FOXM1 amplifications versus those with FOXM1 wild type (WT). (**F**) The expression changes of FOXM1 between samples with FOXM1 amplification and FOXM1 deletion/WT.

CNA was a potential driving factor in ccRCC progression. To investigate whether and how CNAs play roles in the dynamic changes of EDCGs expression, gene-level CNAs were analyzed by GISTIC2.0 software (n = 832). The results showed that a total of 256 EDCGs were significantly affected. FOXM1 was located in an amplified peak in chromosome 12q13.3 (Figure 5A), whereas FOS, FOXO1 and LATS1 were located in deletion peaks (Supplementary Figure 3A). Among them, FOXM1 was a critical transcriptional factor that played a role in cell cycle progression. CN amplification of FOXM1 (115/500, 23%) was affected by CN amplifications (FDR = 2.83e-09, Figure 5B), and associated with high stage (OR = 2.701, 95% CI: 1.766–4.162, FDR = 5.36e-06, Figure 5C). When divided samples into regional/distant metastasis and localized tumors, Gene set enrichment analysis results revealed that the deleted genes (e.g. LATS1, TJP1 and TJP2) were enriched in Hippo signaling pathway and cell-cell junction organization (FDR <0.25, Figure 5D), and involved GO functions included protein kinase activity, cell adhesion and transcriptional regulation (FDR <0.05, Supplementary Figure 3B). In contrast, the amplified genes were involved in cell cycle, DNA replication and chromosome maintenance pathways (FDR <0.25, Figure 5D), and related GO functions (FDR <0.05) included cell cycle, DNA damage/repair and intracellular primary metabolic process (Supplementary Figure 3C). Furthermore, the OS (log-rank P = 0.0002) and PFS (log-rank P = 0.0029) of ccRCC patients with FOXM1 CN amplification were worse than those with FOXM1 wild type (WT, Figure 5E). In addition, upregulation of FOXM1 affected by CN amplifications (t-test, P < 0.05) was observed both in patients with regional and distant metastasis as well (Figure 5F).

### FOXM1 could be a prognostic marker in pT3 tumors with distant metastasis

**Figure 6:**
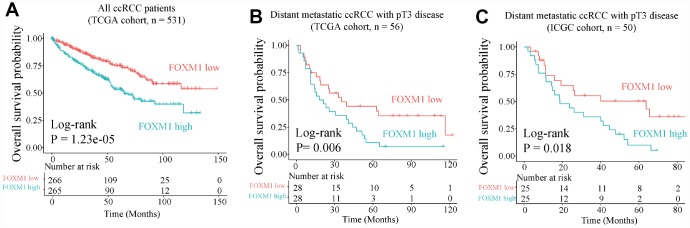
**Overall survival analysis of FOXM1.** (**A**) Overall survival of all ccRCC patient in TCGA cohort. (**B**) and (**C**) Overall survival for pT3 patients with distant metastasis in TCGA (training) and International Cancer Genome Consortium (ICGC, validation) cohorts.

We further investigated the clinical value of EDCGs affected by eQTMs or eQTCNs. We found that FOXM1 was associated with overall survival of patients with ccRCC (log-rank P = 1.23e-05, hazard ratio [HR] = 2.007, 95% CI = 1.46–2.742, Figure 6A). Patients with FOXM1 overexpression showed worse survival. Multivariate Cox analysis showed that FOXM1 (P=0.003, HR=1.693, 95% CI = 1.202–2.383) was also associated with pT3, stage III/IV and age (P<0.05, Supplementary Table 2). FOXM1 could be an independent prognostic factor for pT3 ccRCC patients with distant metastasis (P=0.006, HR=1.719, 95% CI = 1.164–2.538, Supplementary Table 2). The 5-year OS of pT3 patients with distant metastasis in high-group was approximately 30% shorter than that in low-group (Figure 6B). The prognostic value of FOXM1 in pT3 ccRCC with distant metastasis was further validated using International Cancer Genome Consortium cohort (Figure 6C, Supplementary Table 2).

### FOXM1-cg00044170 signature showed high sensitivity and specificity in early diagnosis of ccRCC

**Figure 7:**
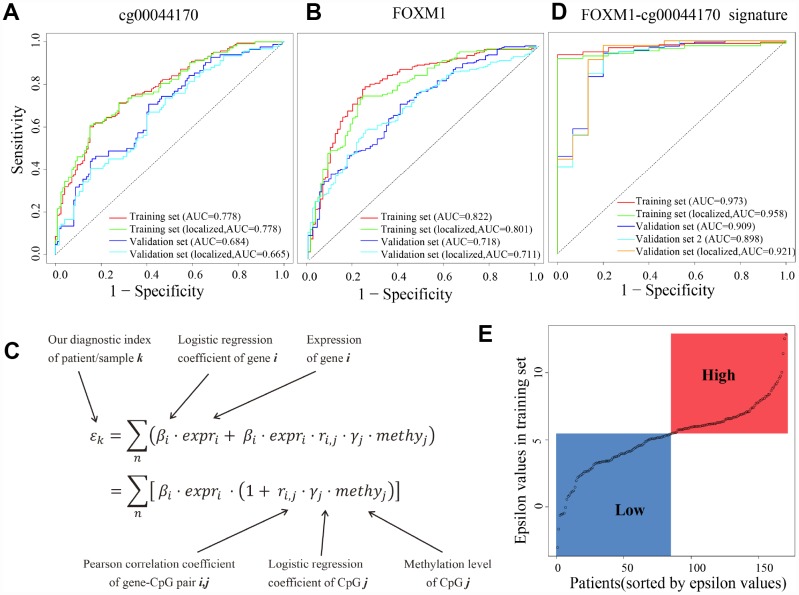
**cg00044170, FOXM1 and the hybrid signature for diagnosis of ccRCC.** (**A**) Receiver operating characteristic (ROC) curves of eQTM (cg00044170 of FOXM1) in classifying all/localized tumors and normal. (**B**) ROC curves of FOXM1 (**C**) The calculation of epsilon value to simultaneously consider expression of genes and methylation of eQTMs. (**D**) ROC curves of eQTM-gene hybrid signature (cg00044170 and FOXM1) in classifying all/localized tumors and normal. (**E**) The distribution of epsilon values of patients in the training set. The median epsilon value was used as cut-off point to divide ccRCC patients into high- and low-group. The datasets named “tumors” (the whole dataset) are stage I/II tumors and stage III/IV tumors, while the datasets named “localized tumors” (the subset) are stage I/II tumors.

We further evaluated the potential of FOXM1 in early diagnosis of ccRCC. Here, we used the logistic regression to classify localized (stage I/II) tumors and normal samples, based on the methylation levels of eQTM cg00044170, expression levels of FOXM1, or epsilon values of FOXM1-cg00044170 signature. A logistic model of the methylation levels of eQTM (cg00044170 of FOXM1) showed high sensitivity and specificity for identifying tumors (area under curve [AUC] = 0.778) and localized tumors (AUC = 0.778) in the training dataset and validation dataset (GEO methylation dataset, AUC = 0.684 and 0.665 respectively, Figure 7A). The diagnostic performance of another model fitted by FOXM1 gene expression might be better than the eQTM model in training set (GDC HT-Seq dataset, AUC = 0.822 and 0.801, respectively, Figure 7B). However, the results in another dataset (TCGA-GTEX dataset) was also dropped (AUC = 0.718 and 0.711, respectively, Figure 7B). We used 10-fold cross-validation and applied the regularization parameter to avoid overfitting in Figure7B. In the present study, an improved method was applied to improve the sensitivity and specificity of early diagnosis by using the genes expression levels and the methylation levels of the corresponding eQTM (Figure 7C). The GDC Expression-Methylation dataset was randomly divided into two datasets and were used for training and validation. Results showed that both the sensitivity and specificity for ccRCC diagnosis were conspicuously improved (AUC = 0.973 in the training set, AUC = 0.909 in the validation set, Figure 7D). This FOXM1-cg00044170 model (the epsilon values of samples as independent variable) also showed high sensitivity and specificity for ccRCC tumors diagnosis in validation dataset 2 (GEO Expression-Methylation paired dataset, GSE105288, AUC = 0.898). Especially for diagnosing localized tumors, the epsilon value has superior performance (AUC = 0.958 in the training set, AUC = 0.921 in the validation set, Figure 7D). The epsilon values of patients in the training set were divided into high- and low-group using median as cut-off (Figure 7E).

## DISCUSSION

EMT-mediated tumor progression was widely observed in various cancer types [9, 10]. Increasing evidences suggested that comprehensive study of genome instability and chromatin modifications dynamics is crucial for identifying cancer biomarkers [14, 15] and remains challenging as well. Our study, for the first time, systematically analyzed the expression and DNA methylation patterns by leveraging the quantified degree of changes to analyze the omics-level dynamics of EMT-related genes and signaling pathways. We found that cell cycle-related gene FOXM1 was affected by both eQTM and eQTCN. FOXM1 copy number amplification (115/500, 23% of patients), occurred in an amplified peak in chromosome 12q13.3, was enriched in late-stage ccRCC samples and associated with worse overall survival and progression-free survival. FOXM1 may be an independent prognostic marker for overall prognosis of pT3 patients with distant metastasis. Our eQTM-gene signature (FOXM1 and cg00044170) showed high sensitivity and specificity in diagnosis of ccRCC, especially for localized tumors.

Text mining technology has been broadly applied to a wide variety of biological and biomedical sciences, including computational approaches to assist researchers with studies in protein-disease associations, which provides us an opportunity to systematically investigate complex diseases, such as cancer [16]. Here, we identified 756 EMT-related genes by text mining of 24,832 literatures and found that EMT was constantly activated. However, EMT is a complex process affected by genomic and epigenetic alterations [17, 18] via complex signaling networks [19]. FOXM1, a key regulator of cell cycle, proliferation, invasion/migration that involved in tumorigenesis and progression [20–22], has reported to be upregulated in ccRCC [23, 24]. Knockdown of FOXM1 expression levels in ccRCC induced cell cycle arrest with reduced expression of CCNB1, CCND1 and CDK2, and increased expression of p21 and p27 [23]. However, our work have made more progress. First, we validated the prognostic value of FOXM1 in bigger, independent cohorts of ccRCC and also found that FOXM1 has great potential in overall prognosis of metastatic ccRCC. The prognosis of patients with metastatic ccRCC is very poor and currently lack of independent prognostic marker in molecular level. Second, we revealed that FOXM1 was not only a cell cycle-associated gene but also play critical roles in EMT process. Third, our data showed that upregulation of FOXM1 may be affected by both eQTM and eQTCN in progression of ccRCC. Importantly, we revealed that FOXM1 expression was dynamically changed in ccRCC progression. We also revealed and validated the early diagnostic potential of FOXM1-cg00044170 signature (AUC>0.9). Thus, FOXM1 may be a clinical biomarker for independent prognosis and early diagnosis in ccRCC. RCC is characterized by a reprogramming of energetic metabolism. In particular the metabolic flux through glycolysis is partitioned [7], and mitochondrial bioenergetics and OxPhox are impaired [25]. It has been shown that FOXM1 promotes reprogramming of glucose metabolism [22, 26]. Pathways may also undergo dynamic changes following genes expression changes. We showed that EMT, cell cycle and DNA replication were continuously activated, while cell-cell junction was continuously inhibited. Therefore, investigation of the dynamic patterns of EDCGs contributes to deeper understanding of tumor progression and may be helpful for further investigation of cancer driver genes in the future.

Multi-omics data containing transcriptome, genome and epigenome that from the same subjects was valuable and may be critical for dissecting the potential factors of dynamic behaviors of EDCGs in cancer [12, 25], and identifying the association between gene expression and DNA methylation or copy number alterations [27]. We performed rigorous association analysis to identify eQTMs and eQTCNs by integrating expression, DNA methylation and CNA data. About the statistical power, for each analysis with multiple tests, the P values were BH-adjusted for reducing potential false-positive discoveries. In fact, we were surprised that the DNA methylations of 229 eQTMs were not significantly changed between advanced tumors and early ones after BH-adjustment when initial observation. The same phenomenon was observed in all CpGs of EDCGs, which implied the close relevance between aberrant DNA methylation and tumorigenesis in ccRCC. Based on eQTMs analysis, rigorously speaking, FOXM1 upregulation and promoter hypomethylation are significantly correlated (FDR < 0.05), while if hypomethylation leads to upregulation of FOXM1 or upregulation of FOXM1 leads to hypomethylation are unclear and require further functional experiments. Previous evidences showed that loss of H3K36me3 demethylase SETD2 due to genomic alterations and hypermethylation was identified in both primary and metastases of ccRCC [15], while decreased methylation in regional H3K36me3 was only observed in lesions of distant metastases [28]. In fact, hypomethylated CpGs among ~420,000 probes were observed in tumors with distant metastasis, while hypomethylation of EDCGs affected by eQTMs were not identified. Together, the DNA methylation and CNAs during progression of ccRCC might deepen our understanding of the roles of epigenetic dysregulation in activation of cell cycle and EMT.

RCC patients often have advanced disease by the time when observed due to the body is remarkably good at hiding the symptoms. Thus, improving the sensitivity of early diagnosis of tumors is helpful for reducing clinical adverse events [29]. Here, we developed a method by combined examination of gene expression levels and eQTM methylation levels to improve the performance of early diagnosis. As a result, based on the model of FOXM1 and its eQTM cg00044170, the sensitivity and specificity of early diagnosis in ccRCC were apparently raised. However, we noticed that there are only 24 controls in the paired methylation data and gene expression data in TCGA database. Future studies with larger, well-controlled datasets may be needed to achieve more accurate performance. In addition, the strategy proposed that simultaneous apply the expression and DNA methylation levels could be a valuable and promising method for early diagnosis of early ccRCCs. Moreover, FOXM1 was associated with overall survival of patients with ccRCC. Importantly, FOXM1 could be an independent prognostic marker for pT3 patients with distant metastasis. Thus, FOXM1 may be an important clinical biomarker in ccRCC.

In summary, we identified that the signature (FOXM1 and cg00044170) and FOXM1 may be valuable for early diagnosis of ccRCC and OS prognosis for pT3 patients with distant metastasis respectively. Our approaches may be utilized for omics-level spectrum investigations in future studies encompassing larger gene sets, or gene signatures involved in specific biological function modules, which will uncover more staged behaviors of key modulators in cancer.

## MATERIALS AND METHODS

### Multi-omics data acquisition, quality control and preprocessing

HTSeq-counts data of RNA-sequencing (RNA-Seq) and miRNA-seq of ccRCC were downloaded from Genomic Data Commons (GDC, https://portal.gdc.cancer.gov/repository) data portal and used for transcriptomic analysis. The samples with RNA Integrity Number > 7.0 were included. RSEM expected-counts data was re-analyzed using raw sequencing data of The Cancer Genome Atlas (TCGA) and The Genotype-Tissue Expression (GTEx) Consortium downloaded from UCSC Xena (http://xena.ucsc.edu/public) [30]. Twenty four microarray profiles (Affymetrix Human Genome U133 Plus 2.0 Array platform, Illumina, San Diego, CA, USA) were downloaded from Gene Expression Omnibus (GEO) database. The corrected raw background CEL files were analyzed using robust multi-chip average method [31]. All samples from GEO were combined and quantile normalized.

DNA methylation profiles of 312 primary tumors and 155 control samples of Illumina Infinium DNA HumanMethylation450 BeadChip (Illumina, San Diego, CA, USA, 450k) were obtained from GDC legacy archive. Fourteen tumors and 96 controls of 9 studies from GEO database were obtained as GEO methylation dataset. Another dataset GSE105288 with expression-methylation data was composed of 9 primary tumors and 9 normal controls. The raw IDAT files were preprocessed using minfi package [32] in R software (v3.2.5). The background correction was performed using ‘preprocessIllumina’ function without normalization. The samples with mean of detection P value of probes >0.05 or with bad probes (detection P > 0.01) >10% were excluded. The non-specific probes listed by previous study were removed [33]. The CpG probes affected by SNPs or from sex chromosomes were also removed. Moreover, the beta values of bad probes were replaced with NA. The beta-value were transformed to M-value [34]. Finally, the M-values of ~420,000 probes of totally 326 tumors and 251 controls were combined and quantile normalized. The masked CN segment of ccRCC were downloaded to analyze ccRCC-related CN alterations (CNA). The gene-level CNA were generated using GISTIC 2.0.23 [35]. All datasets used in this study were shown in Table 1 and the patient clinical information were provided in Supplementary Tables 3–8. The major code was provided as Supplementary Code 1.

**Table 1:** Datasets used in this study.

**Dataset name**	**Tumor**	**Localized**	**Normal**	**Source/Identifier**
GDC HT-Seq count	531	329	72	GDC data portal
TCGA-GTEx RSEM count	527	325	99	
GDC miRNA HT-Seq count	544	-	71	GDC data portal
GEO expression	189	-	251	GSE11151, GSE11166, GSE12606, GSE13818, GSE18549, GSE19249, GSE19750, GSE20615, GSE20677, GSE22541, GSE25471, GSE25861, GSE27556, GSE28050, GSE33371, GSE34437, GSE41137, GSE46699, GSE66272, GSE7307, GSE75693, GSE76948, GSE8050, GSE81156
GEO DNA methylation	14	8	96	GSE89648, GSE66872, GSE59157, GSE77871, GSE79100, GSE54719, GSE69502, GSE52955, GSE43293
GDC DNA methylation	312	185	155	GDC data portal
GEO expression-methylation	9	-	9	GSE105288
GDC expression-methylation	308	186	24	GDC data portal
GDC copy number	500	-	332	GDC data portal
Expression-CNA paired	445	-	38	GDC data portal

### Text mining

To identify genes correlated with EMT, we performed text mining based on abstracts of literatures in the PubMed database. Specifically, the search criteria “((EMT OR (Epithelial to mesenchymal)) AND (cancer OR tumor OR carcinoma))” were used. The abstracts of 24,832 articles were extracted as input to perform part-of-speech tagging, shallow parsing, and named entity recognition using both GENIA Tagger and Enju software (NaCTeM Software Tools) [36]. Only genes identified by both above softwares were submitted to filter false identified ones and remove duplication using org.Hs.eg.db package (version 3.6).

### Batch effects analysis

Batch effects of five potential confounding factors listed in the recent study [37] and the plate id (a part of TCGA barcode) were assessed by hierarchical clustering and principal component analysis based on MBatch v1.0 software [37]. The batch variables with Dispersion Separability Criterion ≥0.3 and P value <0.05 were considered as significant batch effects. The significant batch variables (batches of the samples processed and the date shipped the data to process) were added as covariates into the design model for differential expression analysis of GDC HT-Seq counts, rather than direct adjustment. Batch effects of the GEO expression dataset were only corrected for the year the data generated using ComBat algorithm [38]. The somatic mutations and CNA data were already discretized and adjusted for background loads.

### Differential expression and DNA methylation analysis

For count data from TCGA, DESeq2 R package (v1.10.1) [39] was used to identify differential expression. For microarray expression profiles, limma package (v3.36.5) [40] was used. For DNA methylation M-values, the limma package [40] and DMRcate package (v1.6.53) [41] were used to identify differentially methylated CpGs and regions, respectively. The P-values were adjusted for multiple test using Benjamini-Hochberg (BH) algorithm. Genes, CpGs or methylated regions with False Discovery Rate (FDR) <0.05 were collected. Only genes differentially expressed in at least two datasets were considered as deregulated. The samples were randomly divided into two datasets with equal sample size to perform differential methylation analysis for mutual validation.

We performed differential expression and differential DNA methylation analysis based on eleven grouping methods: (1) all patients with ccRCC versus normal; (2) low-stage patients versus normal; (3) high-stage patients versus low-stage patients; (4) low-grade patients versus normal; (5) high-grade patients versus low-grade patients; (6) patients without lymph nodes metastasis versus normal; (7) patients with lymph nodes metastasis versus patients without it; (8) patients of progression free versus normal; and (9) recurred patients versus patients are progression free; (10) patients without distant metastasis versus normal; (11) patients with distant metastasis versus those without it.

### Unsupervised clustering analysis

Log2 transformed count per million data of HTSeq-counts was used for gene expression pattern investigation. Gene-level CNAs was used for clustering. The function *dist* in R was used to compute the distance matrix and the function *hclust* was used for clustering. We utilized Euclidean as our distance metric with Ward linkage to cluster both the rows and the columns for gene expression clustering, while Euclidean as distance metric with Ward2 linkage was used for CNAs clustering. For each clustering, the number of clusters was determined using the same distance metric and linkage method by *ConsensusClusterPlus* package (v1.44.0) [42] in R. Clusters shown in heatmap were separated using the *cutree* function.

### eQTMs and eQTCNs identification

GTEx project identified expression quantitative trait loci in ~53 human tissues that influence gene expression [27]. Similarly, the expression quantitative trait methylation (eQTMs) and expression quantitative trait CN (eQTCNs) were also able to affect gene expression. In this study, we performed eQTMs analysis based on deregulated genes and corresponding differentially methylated CpG islands in their gene region mapped using *IlluminaHumanMethylation450kanno.ilmn12.hg19* package. The matched samples (n=332) with gene expression data and methylation data of TCGA were used to perform Pearson correlation analysis and non-zero correlation. CpG-gene pairs with negative Pearson correlation and FDR <0.05 were considered as eQTMs. The eQTCNs analysis was based on genes obtained by GISTIC2.0 with CNA frequency >5% in ccRCC. The CNAs of genes with significant expression changes (unpaired student t-test, FDR <0.05) between samples with CN deletions and samples with CN amplifications were considered as eQTCNs.

### Generalized linear regression analysis

The univariate Cox proportional hazard regression was used to determine the prognosis-related genes. The genes with BH-adjusted P value <0.05 were considered as candidate variables and were subjected to multivariate Cox regression model. Variables with log-rank P-values <0.05 were considered associated with patient survival. The expression data and overall survival rate of pT3 ccRCC patients with distant metastasis from International Cancer Genome Consortium (n=50) cohort were used for overall survival validation. The logistic regression was used to determine diagnostic markers. We used the logistic model to distinguish localized tumors (AJCC stage I/II) and normal samples based on the eQTM methylation, gene expression, or expression-methylation signature levels (epsilon values). The epsilon values of samples were calculated based on the expression levels of specific gene and the methylation levels of corresponding eQTM. Moreover, receiver operating characteristic curves and area under curves were used to evaluate the performance of the classifier. The original methylation datasets and expression datasets were randomly divided into two subsets of equal sample size for training and testing, respectively. This step was repeated for 1000 times. The model with median sensitivity and specificity was eventually considered. In the early diagnosis analysis of FOXM1 methylation, cg00044170 methylation level was used as a classifier to distinguish localized ccRCC tumors (even pT1a tumors) and normal samples. In the early diagnosis analysis of FOXM1-cg00044170 signature, the epsilon values calculated from FOXM1 expression levels and cg00044170 methylation levels by our formula (Figure 7C) was served as a new classifier for early diagnosis. The logistic regression was used to determine whether CN deletion/amplification was associated with advanced tumors. A deleted gene with odds ratio (OR) <1 and FDR <0.05 represents that its deletion was associated with advanced tumors. In contrast, an amplified gene with OR >1 and FDR <0.05 represents its amplification was related to advanced tumors.

## Supplementary Material

Supplementary Figures

Supplementary Tables

Supplementary Code

Supplementary Table 1

Supplementary Table 3

Supplementary Table 4

Supplementary Table 5

Supplementary Table 6

Supplementary Table 7

Supplementary Table 8

Supplementary Code
