# Supplementary material for: Multi-omics analysis reveals epithelial-mesenchymal transition-related gene FOXM1 as a novel prognostic biomarker in clear cell renal carcinoma
Source: Aging (Albany NY). 2019 Nov 19;11(22):10316–37. doi: 10.18632/aging.102459 (PMC6914426; doi:10.18632/aging.102459)
Supplement: Supplementary Figures [file aging-11-102459-s011..pdf]

SUPPLEMENTARY FIGURES

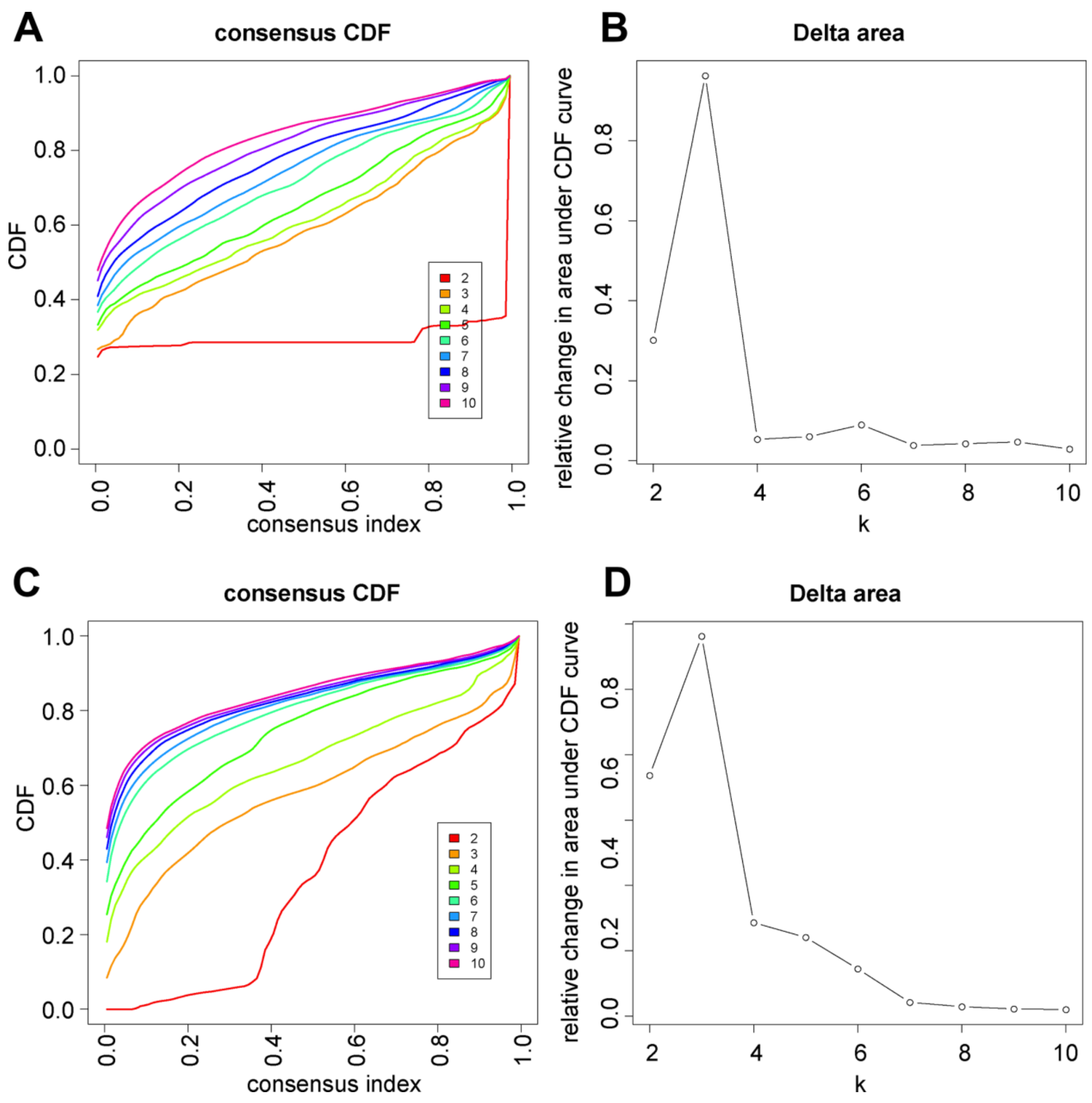

Supplementary Figure 1. Consensus clustering of expression of EMT-related genes using Euclidean as distance metric with Ward linkage. (**A**) and (**C**) Consensus Cumulative Distribution Function (CDF) plots to determine at what number of clusters, k, the CDF reaches an approximate maximum. (**B**) and (**D**) Delta area plots show the relative change in area under the CDF curve comparing k and k-1. They showed that the delta areas had no appreciable increase when k=4 and k=7 for sample clustering (**B**) and gene clustering (**D**), respectively.

	Ontology	ID	Description	P.adjust	Gene ID	
Hypomethylation	MF	GO:0001227	transcriptional repressor activity, RNA polymerase II transcription regulatory region sequence-specific DNA binding	*	FOXM1/TCF3/ZEB1	***Promoter binding***
	MF	GO:0000978	RNA polymerase II proximal promoter sequence-specific DNA binding	**	IRF4/MYBL2/NFKB2/TCF3/TP73/ZEB1	
	MF	GO:0070888	E-box binding	*	TCF3/ZEB1	
	BP	GO:0007050	cell cycle arrest	**	ATM/CCND1/CDK5R1/FOXM1/TP73	***Cell cycle***
	BP	GO:0045931	positive regulation of mitotic cell cycle	*	CCND1/SPHK1/TGFA	
	BP	GO:0045787	positive regulation of cell cycle	**	ATM/CCND1/CDK5R1/SPHK1/TGFA/TP73	
	BP	GO:0048285	organelle fission	*	ATM/KDR/MKI67/MYBL2/TGFA	
	BP	GO:0140014	mitotic nuclear division	*	ATM/MKI67/MYBL2/TGFA	
	BP	GO:0018105	peptidyl-serine phosphorylation	*	ATM/CDK5R1/DCLK1/EPO	***(auto)phosphorylation***
	MF	GO:0035004	phosphatidylinositol 3-kinase activity	*	ATM/FYN	
	BP	GO:0071902	positive regulation of protein serine/threonine kinase activity	*	CCND1/CDK5R1/ROBO1/TGFA/TP73	
	BP	GO:0046425	regulation of JAK-STAT cascade	*	CDK5R1/EPO/FYN	
	BP	GO:0050731	positive regulation of peptidyl-tyrosine phosphorylation	**	EPO/FYN/TGFA/TNK2	
	BP	GO:0038083	peptidyl-tyrosine autophosphorylation	**	FYN/KDR/TNK2	
	BP	GO:0050673	epithelial cell proliferation	*	CCND1/KDR/ROBO1/RUNX3/TGFA	***Epithelial cell proliferation***
	BP	GO:0050679	positive regulation of epithelial cell proliferation	*	CCND1/KDR/TGFA	
	BP	GO:0033598	mammary gland epithelial cell proliferation	*	CCND1/ROBO1	
	BP	GO:0030857	negative regulation of epithelial cell differentiation	*	CCND1/ZEB1	***Epithelial cell differentiation***
Hypermethylation	BP	GO:0007162	negative regulation of cell adhesion	*	CDH1/CLDN7/EPCAM/ERBB2/MUC1/RHOA	***Cell junction***
	CC	GO:0005911	cell-cell junction	**	CDH1/CLDN7/EPCAM/GRHL2/OCLN/RHOA/TJP1/TJP2	
	CC	GO:0043296	apical junction complex	***	CDH1/CLDN7/EPCAM/OCLN/RHOA/TJP1/TJP2	
	BP	GO:0045216	cell-cell junction organization	*	CDH1/GRHL2/ITGA2/OCLN/RHOA/TJP1	
	BP	GO:0007160	cell-matrix adhesion	**	CDK6/DDR1/GSK3B/ITGA2/RHOA	
	BP	GO:0046777	protein autophosphorylation	*	BCR/CAMK2G/DDR1/ERBB2/GSK3B/MTOR/SIK2	***(auto)phosphorylation***
	BP	GO:0018108	peptidyl-tyrosine phosphorylation	*	BCR/DDR1/ERBB2/HDAC1/IL11/MTOR	
	BP	GO:0018105	peptidyl-serine phosphorylation	*	CAMK2G/CHUK/GSK3B/IL11/MAPK8/MTOR	
	BP	GO:0018107	peptidyl-threonine phosphorylation	*	CAMK2G/GSK3B/MAPK8/MTOR	
	BP	GO:0042326	negative regulation of phosphorylation	*	CDK6/CDKN1C/DUSP6/FOXO1/MECOM/MTOR/RHOA/SMAD4/SOCS2	
	BP	GO:2000756	regulation of peptidyl-lysine acetylation	*	HDAC1/MUC1/SMAD4	***Histone modification***
	BP	GO:0016570	histone modification	*	HDAC1/MAPK8/MECOM/MUC1/RNF8/SETD2/SMAD4	

Supplementary Figure 2. Gene ontology enrichment for expression quantitative trait methylation (eQTMs).

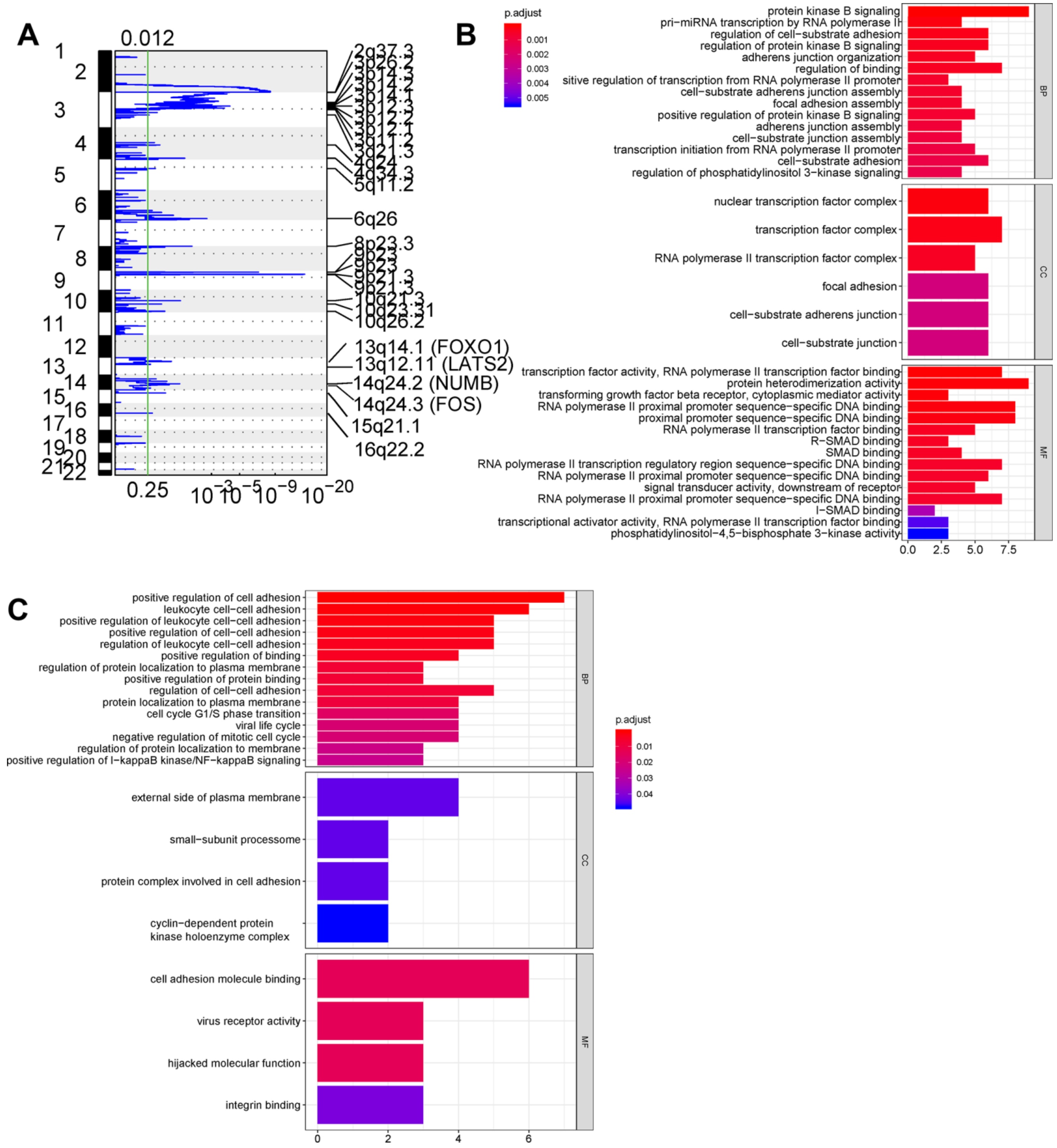

Supplementary Figure 3. Frequent deleted areas of ccRCC genome and involved functions of expression quantitative trait copy number (eQTCNs). (**A**) The EDCGs located in the focal CN deletion peaks. False-discovery rates (q values) and scores generated by GISTIC 2.0 for amplifications (x-axis) are plotted against chromosome locations (y-axis). Dotted lines indicate the centromeres. The green line represents cut-off (q = 0.25) that determines statistical significance. (**B**) Deleted EDCGs involve GO functions. (**C**) Amplified EDCGs involve GO functions.
