# Supplementary material for: Multi-omics analysis reveals epithelial-mesenchymal transition-related gene FOXM1 as a novel prognostic biomarker in clear cell renal carcinoma
Source: Aging (Albany NY). 2019 Nov 19;11(22):10316–37. doi: 10.18632/aging.102459 (PMC6914426; doi:10.18632/aging.102459)
Supplement: Supplementary Tables [file aging-11-102459-s010..pdf]

SUPPLEMENTARY TABLES

Please browse Full Text version to see the data of Supplementary Tables 1, 3–8.

Supplementary Table 1. 756 EMT-related genes identified by text-mining in this study.

Supplementary Table 2. The univariate and multivariate Cox regression model.

Variable	Univariate regression		Multivariate regression	
	**P value**	**Hazard ratio (95% CI)**	**P value**	**Hazard ratio (95% CI)**
Overall survival of FOXM1 (TCGA cohort, n =531)				
FOXM1 (High vs Low)	**2.19E-07**	2.309(1.683–3.169)	**2.56E-03**	1.693(1.202–2.383)
Age (median = 60)	**3.99E-04**	1.748(1.283–2.380)	**1.05E-03**	1.700(1.237–2.334)
Gender (ref = Female)				
Male	0.781	0.957(0.700–1.307)	0.639	0.924(0.664–1.286)
Stage (ref = I)				
II	0.48	1.250(0.673–2.321)	0.088	3.184(0.841–12.057)
III	**4.89E-06**	2.609(1.729–3.936)	**0.002**	5.237(1.817–15.094)
IV	**2.00E-16**	6.880(4.694–10.084)	**2.00E-04**	1.773(3.762–83.486)
Unknown	**0.049**	7.394(1.008–54.236)	**0.014**	2.055(1.841–229.394)
Grade (ref = G1)				
G2	0.993	1.329 (0.512–1.968)	0.992	2.594(0.698–3.855)
G3	0.993	1.843 (0.453–1.572)	0.991	2.657(0.789–5.815)
G4	0.994	2.015(0.679–5.320)	0.992	4.621(0.664–6.144)
Unknown	0.994	1.552(0.684–1.801)	0.992	2.318(0.678–4.314)
pT (ref = T1)				
T2	0.069	1.606(0.963–2.678)	0.069	3.349(0.103–1.109)
T3	**1.98E-13**	3.285(2.321–4.651)	**0.022**	0.313(0.123–0.894)
T4	**9.60E-12**	10.763(5.433–21.323)	0.106	0.381(0.118–1.227)
pN (ref = N0)				
N1	**7.68E-05**	3.585(1.904–6.750)	0.648	2.529 (0.618–10.362)
Unknown	0.259	0.836(0.613–1.141)	0.139	0.785(0.570–1.082)
pM (ref = M0)				
M1	**2.00E-16**	1.638 (1.423–18.603)	0.626	2.529 (0.618–10.362)
Unknown	0.695	0.795(0.251–2.511)	0.468	0.611(0.162–2.312)
TCGA cohort (training, n=56)	Overall survival (distant metastatic patients with pT3 disease)			
FOXM1 (High vs Low)	**4.86E-04**	1.878 (1.318–2.677)	**6.41E-03**	1.719 (1.164–2.538)
Age (median = 60)	0.946	1.021 (0.564–1.848)	0.76	1.005 (0.982–1.027)
Gender (Male vs Female)	0.085	0.567 (0.299–1.081)	0.182	1.317 (0.806–2.151)
Grade (ref = G1)				
G2	**0.043**	0.329 (0.112–0.968)	0.168	0.454 (0.147–1.395)
G3	0.593	0.843 (0.453–1.572)	0.645	0.858 (0.447–1.645)
G4	NA	NA	NA	NA
pN (N1 vs N0)	**0.0125**	1.638 (1.423–18.603)	0.197	2.529 (0.618–10.362)
ICGC cohort (validation, n=50)	Overall survival (distant metastatic patients with pT3 disease)			
FOXM1 (High vs Low)	**3.62E-03**	1.762(1.203–2.582)	**0.018**	1.622(1.079–2.437)

Age (median = 61)	0.695	0.876(0.453–1.696)	0.926	0.967(0.472–1.979)
Gender (Male vs Female)	0.091	0.538(0.262–1.105)	0.245	0.626(0.285–1.378)
Grade (ref = G1)				
G2	NA	NA	NA	NA
G3	0.136	2.323(0.767–7.041)	0.356	1.752(0.520–5.899)
G4	0.087	2.604(0.871–7.790)	0.25	1.969(0.621–6.241)
pN (N1 vs N0)	0.11	1.248(0.754–16.092)	0.468	1.826(0.359–9.285)

Notes: P values in Bold represent statistical significant. NA represent no samples in that category or all samples belong to the same category.

Supplementary Table 3. The patients and corresponding clinical information of GDC Htseq-counts dataset (n=603).

Supplementary Table 4. The patients and corresponding clinical information of GDC miRNA-Seq dataset (n=615).

Supplementary Table 5. The patients and corresponding clinical information of TCGA-GTEx dataset (n=626).

Supplementary Table 6. The patients and corresponding clinical information of GEO expression dataset (n=440).

Supplementary Table 7. The patients and corresponding clinical information of DNA methylation dataset (n=577).

Supplementary Table 8. The patients and corresponding clinical information of GDC copy number dataset (n=832).
