# Supplementary material for: Multi-omics analysis reveals epithelial-mesenchymal transition-related gene FOXM1 as a novel prognostic biomarker in clear cell renal carcinoma
Source: Aging (Albany NY). 2019 Nov 19;11(22):10316–37. doi: 10.18632/aging.102459 (PMC6914426; doi:10.18632/aging.102459)
Supplement: Supplementary Code [file aging-11-102459-s009..pdf]

## SUPPLEMENTARY CODE

Please browse Full Text version to see the data of Supplementary Code.

**Supplementary Code.** (**i**) the code (Perl language) used to perform text mining in order to extract raw EMT related genes/proteins from Pubmed query xml results; (**ii**) the code (R language) for unsupervised clustering analysis based on expression levels of 756 EMT-related genes using multiple R packages; (**iii**) the code (Perl language) of our custom Perl functions to perform batch effect evaluation using MBatch v1.0 software; (**iv**) the code (Perl language) used to perform expression quantitative trait methylation (eQTM) and expression quantitative trait copy number alterations (eQTCN) analysis.
