# Supplementary material for: Multi-omics analysis reveals epithelial-mesenchymal transition-related gene FOXM1 as a novel prognostic biomarker in clear cell renal carcinoma
Source: Aging (Albany NY). 2019 Nov 19;11(22):10316–37. doi: 10.18632/aging.102459 (PMC6914426; doi:10.18632/aging.102459)
Supplement: Supplementary Table 1 [file aging-11-102459-s003..docx]

| Supplementary Table S1. 756 EMT-related genes identified by text-mining in this study | | |
| --- | --- | --- |
| SYMBOL | ENTREZID | GENENAME |
| ABCA3 | 21 | ATP binding cassette subfamily A member 3 |
| ABCB1 | 5243 | ATP binding cassette subfamily B member 1 |
| ABCC1 | 4363 | ATP binding cassette subfamily C member 1 |
| ACTA2 | 59 | actin, alpha 2, smooth muscle, aorta |
| ACVR1 | 90 | activin A receptor type 1 |
| ACVRL1 | 94 | activin A receptor like type 1 |
| ADAM10 | 102 | ADAM metallopeptidase domain 10 |
| ADAM9 | 8754 | ADAM metallopeptidase domain 9 |
| ADGRL1 | 22859 | adhesion G protein-coupled receptor L1 |
| ADIPOQ | 9370 | adiponectin, C1Q and collagen domain containing |
| AIMP2 | 7965 | aminoacyl tRNA synthetase complex interacting multifunctional protein 2 |
| AKAP12 | 9590 | A-kinase anchoring protein 12 |
| ALAD | 210 | aminolevulinate dehydratase |
| ALCAM | 214 | activated leukocyte cell adhesion molecule |
| ALDH1A2 | 8854 | aldehyde dehydrogenase 1 family member A2 |
| ALDH7A1 | 501 | aldehyde dehydrogenase 7 family member A1 |
| ALOX5 | 240 | arachidonate 5-lipoxygenase |
| ALX1 | 8092 | ALX homeobox 1 |
| ALX4 | 60529 | ALX homeobox 4 |
| AMFR | 267 | autocrine motility factor receptor |
| AMY1A | 276 | amylase, alpha 1A (salivary) |
| ANG | 283 | angiogenin |
| ANGPT2 | 285 | angiopoietin 2 |
| ANXA1 | 301 | annexin A1 |
| ANXA2 | 302 | annexin A2 |
| ANXA8 | 653145 | annexin A8 |
| APAF1 | 317 | apoptotic peptidase activating factor 1 |
| AR | 367 | androgen receptor |
| ARF6 | 382 | ADP ribosylation factor 6 |
| ARG1 | 383 | arginase 1 |
| ARHGAP1 | 392 | Rho GTPase activating protein 1 |
| ARHGAP10 | 79658 | Rho GTPase activating protein 10 |
| ARPC2 | 10109 | actin related protein 2/3 complex subunit 2 |
| ASCL2 | 430 | achaete-scute family bHLH transcription factor 2 |
| ATM | 472 | ATM serine/threonine kinase |
| ATXN1 | 6310 | ataxin 1 |
| AXIN1 | 8312 | axin 1 |
| AXIN2 | 8313 | axin 2 |
| AXL | 558 | AXL receptor tyrosine kinase |
| BACE2 | 25825 | beta-site APP-cleaving enzyme 2 |
| BAD | 572 | BCL2 associated agonist of cell death |
| BAK1 | 578 | BCL2 antagonist/killer 1 |
| BAP1 | 8314 | BRCA1 associated protein 1 |
| BAX | 581 | BCL2 associated X, apoptosis regulator |
| BCAR1 | 9564 | BCAR1, Cas family scaffold protein |
| BCL10 | 8915 | B cell CLL/lymphoma 10 |
| BCL2 | 596 | BCL2, apoptosis regulator |
| BCL2A1 | 597 | BCL2 related protein A1 |
| BCL2L1 | 598 | BCL2 like 1 |
| BCL6 | 604 | B cell CLL/lymphoma 6 |
| BCL9 | 607 | B cell CLL/lymphoma 9 |
| BCL9L | 283149 | B cell CLL/lymphoma 9 like |
| BCR | 613 | BCR, RhoGEF and GTPase activating protein |
| BHLHE41 | 79365 | basic helix-loop-helix family member e41 |
| BIRC2 | 329 | baculoviral IAP repeat containing 2 |
| BIRC3 | 330 | baculoviral IAP repeat containing 3 |
| BLNK | 29760 | B cell linker |
| BMI1 | 648 | BMI1 proto-oncogene, polycomb ring finger |
| BMP2 | 650 | bone morphogenetic protein 2 |
| BRCA1 | 672 | BRCA1, DNA repair associated |
| BRD4 | 23476 | bromodomain containing 4 |
| BRD7 | 29117 | bromodomain containing 7 |
| BRF2 | 55290 | BRF2, RNA polymerase III transcription initiation factor subunit |
| BRMS1 | 25855 | breast cancer metastasis suppressor 1 |
| BST1 | 683 | bone marrow stromal cell antigen 1 |
| BUB1 | 699 | BUB1 mitotic checkpoint serine/threonine kinase |
| BUB1B | 701 | BUB1 mitotic checkpoint serine/threonine kinase B |
| C1orf61 | 10485 | chromosome 1 open reading frame 61 |
| C3 | 718 | complement C3 |
| CABIN1 | 23523 | calcineurin binding protein 1 |
| CAMK2G | 818 | calcium/calmodulin dependent protein kinase II gamma |
| CAMP | 820 | cathelicidin antimicrobial peptide |
| CAT | 847 | catalase |
| CAV1 | 857 | caveolin 1 |
| CAV2 | 858 | caveolin 2 |
| CBL | 867 | Cbl proto-oncogene |
| CBX5 | 23468 | chromobox 5 |
| CCL11 | 6356 | C-C motif chemokine ligand 11 |
| CCL2 | 6347 | C-C motif chemokine ligand 2 |
| CCL3 | 6348 | C-C motif chemokine ligand 3 |
| CCL4 | 6351 | C-C motif chemokine ligand 4 |
| CCL5 | 6352 | C-C motif chemokine ligand 5 |
| CCNA2 | 890 | cyclin A2 |
| CCND1 | 595 | cyclin D1 |
| CCND2 | 894 | cyclin D2 |
| CCNE1 | 898 | cyclin E1 |
| CCR1 | 1230 | C-C motif chemokine receptor 1 |
| CCR2 | 729230 | C-C motif chemokine receptor 2 |
| CCR5 | 1234 | C-C motif chemokine receptor 5 (gene/pseudogene) |
| CCR6 | 1235 | C-C motif chemokine receptor 6 |
| CCR7 | 1236 | C-C motif chemokine receptor 7 |
| CD14 | 929 | CD14 molecule |
| CD151 | 977 | CD151 molecule (Raph blood group) |
| CD163 | 9332 | CD163 molecule |
| CD24 | 100133941 | CD24 molecule |
| CD34 | 947 | CD34 molecule |
| CD36 | 948 | CD36 molecule |
| CD4 | 920 | CD4 molecule |
| CD40LG | 959 | CD40 ligand |
| CD44 | 960 | CD44 molecule (Indian blood group) |
| CD58 | 965 | CD58 molecule |
| CD59 | 966 | CD59 molecule (CD59 blood group) |
| CD63 | 967 | CD63 molecule |
| CD68 | 968 | CD68 molecule |
| CD74 | 972 | CD74 molecule |
| CD80 | 941 | CD80 molecule |
| CD82 | 3732 | CD82 molecule |
| CD86 | 942 | CD86 molecule |
| CD9 | 928 | CD9 molecule |
| CD99L2 | 83692 | CD99 molecule like 2 |
| CDC25C | 995 | cell division cycle 25C |
| CDC42 | 998 | cell division cycle 42 |
| CDC6 | 990 | cell division cycle 6 |
| CDH1 | 999 | cadherin 1 |
| CDH11 | 1009 | cadherin 11 |
| CDH2 | 1000 | cadherin 2 |
| CDH3 | 1001 | cadherin 3 |
| CDH6 | 1004 | cadherin 6 |
| CDK1 | 983 | cyclin dependent kinase 1 |
| CDK10 | 8558 | cyclin dependent kinase 10 |
| CDK2 | 1017 | cyclin dependent kinase 2 |
| CDK3 | 1018 | cyclin dependent kinase 3 |
| CDK4 | 1019 | cyclin dependent kinase 4 |
| CDK5 | 1020 | cyclin dependent kinase 5 |
| CDK5R1 | 8851 | cyclin dependent kinase 5 regulatory subunit 1 |
| CDK6 | 1021 | cyclin dependent kinase 6 |
| CDK8 | 1024 | cyclin dependent kinase 8 |
| CDK9 | 1025 | cyclin dependent kinase 9 |
| CDKN1A | 1026 | cyclin dependent kinase inhibitor 1A |
| CDKN1B | 1027 | cyclin dependent kinase inhibitor 1B |
| CDKN1C | 1028 | cyclin dependent kinase inhibitor 1C |
| CDKN2A | 1029 | cyclin dependent kinase inhibitor 2A |
| CDKN2B | 1030 | cyclin dependent kinase inhibitor 2B |
| CDKN2C | 1031 | cyclin dependent kinase inhibitor 2C |
| CDX1 | 1044 | caudal type homeobox 1 |
| CDX2 | 1045 | caudal type homeobox 2 |
| CHD5 | 26038 | chromodomain helicase DNA binding protein 5 |
| CHEK1 | 1111 | checkpoint kinase 1 |
| CHEK2 | 11200 | checkpoint kinase 2 |
| CHST10 | 9486 | carbohydrate sulfotransferase 10 |
| CHUK | 1147 | conserved helix-loop-helix ubiquitous kinase |
| CITED2 | 10370 | Cbp/p300 interacting transactivator with Glu/Asp rich carboxy-terminal domain 2 |
| CLDN1 | 9076 | claudin 1 |
| CLDN3 | 1365 | claudin 3 |
| CLDN4 | 1364 | claudin 4 |
| CLDN6 | 9074 | claudin 6 |
| CLDN7 | 1366 | claudin 7 |
| CLEC1B | 51266 | C-type lectin domain family 1 member B |
| COL13A1 | 1305 | collagen type XIII alpha 1 chain |
| COL1A1 | 1277 | collagen type I alpha 1 chain |
| COL1A2 | 1278 | collagen type I alpha 2 chain |
| COL4A1 | 1282 | collagen type IV alpha 1 chain |
| COPS5 | 10987 | COP9 signalosome subunit 5 |
| COPS6 | 10980 | COP9 signalosome subunit 6 |
| CPA1 | 1357 | carboxypeptidase A1 |
| CR1 | 1378 | complement C3b/C4b receptor 1 (Knops blood group) |
| CRB3 | 92359 | crumbs 3, cell polarity complex component |
| CREB1 | 1385 | cAMP responsive element binding protein 1 |
| CRHR2 | 1395 | corticotropin releasing hormone receptor 2 |
| CRK | 1398 | CRK proto-oncogene, adaptor protein |
| CRKL | 1399 | CRK like proto-oncogene, adaptor protein |
| CSF1 | 1435 | colony stimulating factor 1 |
| CSF2 | 1437 | colony stimulating factor 2 |
| CSF3 | 1440 | colony stimulating factor 3 |
| CSN2 | 1447 | casein beta |
| CSNK2A1 | 1457 | casein kinase 2 alpha 1 |
| CST3 | 1471 | cystatin C |
| CST5 | 1473 | cystatin D |
| CTGF | 1490 | connective tissue growth factor |
| CTNNA1 | 1495 | catenin alpha 1 |
| CTNNAL1 | 8727 | catenin alpha like 1 |
| CTNNB1 | 1499 | catenin beta 1 |
| CTSB | 1508 | cathepsin B |
| CTSD | 1509 | cathepsin D |
| CTSL | 1514 | cathepsin L |
| CUL4A | 8451 | cullin 4A |
| CUL7 | 9820 | cullin 7 |
| CUX1 | 1523 | cut like homeobox 1 |
| CX3CR1 | 1524 | C-X3-C motif chemokine receptor 1 |
| CXCL1 | 2919 | C-X-C motif chemokine ligand 1 |
| CXCL12 | 6387 | C-X-C motif chemokine ligand 12 |
| CXCL2 | 2920 | C-X-C motif chemokine ligand 2 |
| CXCL8 | 3576 | C-X-C motif chemokine ligand 8 |
| CXCR1 | 3577 | C-X-C motif chemokine receptor 1 |
| CXCR2 | 3579 | C-X-C motif chemokine receptor 2 |
| CXCR3 | 2833 | C-X-C motif chemokine receptor 3 |
| CXCR4 | 7852 | C-X-C motif chemokine receptor 4 |
| CYBA | 1535 | cytochrome b-245 alpha chain |
| CYR61 | 3491 | cysteine rich angiogenic inducer 61 |
| DAB2 | 1601 | DAB2, clathrin adaptor protein |
| DCLK1 | 9201 | doublecortin like kinase 1 |
| DDA1 | 79016 | DET1 and DDB1 associated 1 |
| DDAH1 | 23576 | dimethylarginine dimethylaminohydrolase 1 |
| DDB2 | 1643 | damage specific DNA binding protein 2 |
| DDR1 | 780 | discoidin domain receptor tyrosine kinase 1 |
| DDR2 | 4921 | discoidin domain receptor tyrosine kinase 2 |
| DDX3X | 1654 | DEAD-box helicase 3, X-linked |
| DDX5 | 1655 | DEAD-box helicase 5 |
| DIAPH3 | 81624 | diaphanous related formin 3 |
| DKK1 | 22943 | dickkopf WNT signaling pathway inhibitor 1 |
| DKK2 | 27123 | dickkopf WNT signaling pathway inhibitor 2 |
| DKK4 | 27121 | dickkopf WNT signaling pathway inhibitor 4 |
| DLAT | 1737 | dihydrolipoamide S-acetyltransferase |
| DLC1 | 10395 | DLC1 Rho GTPase activating protein |
| DLD | 1738 | dihydrolipoamide dehydrogenase |
| DLK1 | 8788 | delta like non-canonical Notch ligand 1 |
| DLL4 | 54567 | delta like canonical Notch ligand 4 |
| DLX2 | 1746 | distal-less homeobox 2 |
| DNAJB6 | 10049 | DnaJ heat shock protein family (Hsp40) member B6 |
| DNM1L | 10059 | dynamin 1 like |
| DOCK10 | 55619 | dedicator of cytokinesis 10 |
| DOCK3 | 1795 | dedicator of cytokinesis 3 |
| DPP4 | 1803 | dipeptidyl peptidase 4 |
| DUOX2 | 50506 | dual oxidase 2 |
| DUSP6 | 1848 | dual specificity phosphatase 6 |
| DVL2 | 1856 | dishevelled segment polarity protein 2 |
| EBNA1BP2 | 10969 | EBNA1 binding protein 2 |
| ECE1 | 1889 | endothelin converting enzyme 1 |
| EDN1 | 1906 | endothelin 1 |
| EEF2 | 1938 | eukaryotic translation elongation factor 2 |
| EGF | 1950 | epidermal growth factor |
| EGFR | 1956 | epidermal growth factor receptor |
| EGR1 | 1958 | early growth response 1 |
| EHD2 | 30846 | EH domain containing 2 |
| EHF | 26298 | ETS homologous factor |
| ELF3 | 1999 | E74 like ETS transcription factor 3 |
| ELF5 | 2001 | E74 like ETS transcription factor 5 |
| ELK1 | 2002 | ELK1, ETS transcription factor |
| ELMO1 | 9844 | engulfment and cell motility 1 |
| ENG | 2022 | endoglin |
| ENO1 | 2023 | enolase 1 |
| EP300 | 2033 | E1A binding protein p300 |
| EPAS1 | 2034 | endothelial PAS domain protein 1 |
| EPCAM | 4072 | epithelial cell adhesion molecule |
| EPO | 2056 | erythropoietin |
| ERBB2 | 2064 | erb-b2 receptor tyrosine kinase 2 |
| ERBB3 | 2065 | erb-b2 receptor tyrosine kinase 3 |
| ERN1 | 2081 | endoplasmic reticulum to nucleus signaling 1 |
| ERRFI1 | 54206 | ERBB receptor feedback inhibitor 1 |
| ESM1 | 11082 | endothelial cell specific molecule 1 |
| ESR1 | 2099 | estrogen receptor 1 |
| ESRP1 | 54845 | epithelial splicing regulatory protein 1 |
| ETS1 | 2113 | ETS proto-oncogene 1, transcription factor |
| ETV4 | 2118 | ETS variant 4 |
| EWSR1 | 2130 | EWS RNA binding protein 1 |
| EYA2 | 2139 | EYA transcriptional coactivator and phosphatase 2 |
| EZH2 | 2146 | enhancer of zeste 2 polycomb repressive complex 2 subunit |
| F2 | 2147 | coagulation factor II, thrombin |
| FANK1 | 92565 | fibronectin type III and ankyrin repeat domains 1 |
| FAS | 355 | Fas cell surface death receptor |
| FBN1 | 2200 | fibrillin 1 |
| FBXL5 | 26234 | F-box and leucine rich repeat protein 5 |
| FBXW7 | 55294 | F-box and WD repeat domain containing 7 |
| FCGBP | 8857 | Fc fragment of IgG binding protein |
| FDXR | 2232 | ferredoxin reductase |
| FERMT1 | 55612 | fermitin family member 1 |
| FGF1 | 2246 | fibroblast growth factor 1 |
| FGFR4 | 2264 | fibroblast growth factor receptor 4 |
| FHOD1 | 29109 | formin homology 2 domain containing 1 |
| FIGF | NA | NA |
| FLI1 | 2313 | Fli-1 proto-oncogene, ETS transcription factor |
| FLOT2 | 2319 | flotillin 2 |
| FN1 | 2335 | fibronectin 1 |
| FOS | 2353 | Fos proto-oncogene, AP-1 transcription factor subunit |
| FOSL1 | 8061 | FOS like 1, AP-1 transcription factor subunit |
| FOSL2 | 2355 | FOS like 2, AP-1 transcription factor subunit |
| FOXA1 | 3169 | forkhead box A1 |
| FOXC1 | 2296 | forkhead box C1 |
| FOXC2 | 2303 | forkhead box C2 |
| FOXF2 | 2295 | forkhead box F2 |
| FOXK1 | 221937 | forkhead box K1 |
| FOXM1 | 2305 | forkhead box M1 |
| FOXO1 | 2308 | forkhead box O1 |
| FOXO4 | 4303 | forkhead box O4 |
| FOXQ1 | 94234 | forkhead box Q1 |
| FPR2 | 2358 | formyl peptide receptor 2 |
| FRMD5 | 84978 | FERM domain containing 5 |
| FSCN1 | 6624 | fascin actin-bundling protein 1 |
| FTH1 | 2495 | ferritin heavy chain 1 |
| FUT2 | 2524 | fucosyltransferase 2 |
| FUT3 | 2525 | fucosyltransferase 3 (Lewis blood group) |
| FXYD3 | 5349 | FXYD domain containing ion transport regulator 3 |
| FXYD5 | 53827 | FXYD domain containing ion transport regulator 5 |
| FYN | 2534 | FYN proto-oncogene, Src family tyrosine kinase |
| FZD4 | 8322 | frizzled class receptor 4 |
| FZD5 | 7855 | frizzled class receptor 5 |
| FZD7 | 8324 | frizzled class receptor 7 |
| GAB1 | 2549 | GRB2 associated binding protein 1 |
| GCNT2 | 2651 | glucosaminyl (N-acetyl) transferase 2 (I blood group) |
| GDF9 | 2661 | growth differentiation factor 9 |
| GFER | 2671 | growth factor, augmenter of liver regeneration |
| GFPT1 | 2673 | glutamine--fructose-6-phosphate transaminase 1 |
| GIPC1 | 10755 | GIPC PDZ domain containing family member 1 |
| GJA1 | 2697 | gap junction protein alpha 1 |
| GJB5 | 2709 | gap junction protein beta 5 |
| GLI1 | 2735 | GLI family zinc finger 1 |
| GLIS1 | 148979 | GLIS family zinc finger 1 |
| GLS | 2744 | glutaminase |
| GLS2 | 27165 | glutaminase 2 |
| GPC3 | 2719 | glypican 3 |
| GPSM2 | 29899 | G protein signaling modulator 2 |
| GRB2 | 2885 | growth factor receptor bound protein 2 |
| GRHL2 | 79977 | grainyhead like transcription factor 2 |
| GSC | 145258 | goosecoid homeobox |
| GSK3B | 2932 | glycogen synthase kinase 3 beta |
| GZMB | 3002 | granzyme B |
| HAS2 | 3037 | hyaluronan synthase 2 |
| HAVCR1 | 26762 | hepatitis A virus cellular receptor 1 |
| HDAC1 | 3065 | histone deacetylase 1 |
| HDAC4 | 9759 | histone deacetylase 4 |
| HEY1 | 23462 | hes related family bHLH transcription factor with YRPW motif 1 |
| HEY2 | 23493 | hes related family bHLH transcription factor with YRPW motif 2 |
| HGF | 3082 | hepatocyte growth factor |
| HIF1A | 3091 | hypoxia inducible factor 1 alpha subunit |
| HLA-DRB1 | 3123 | major histocompatibility complex, class II, DR beta 1 |
| HMGA1 | 3159 | high mobility group AT-hook 1 |
| HMMR | 3161 | hyaluronan mediated motility receptor |
| HMOX1 | 3162 | heme oxygenase 1 |
| HNF4A | 3172 | hepatocyte nuclear factor 4 alpha |
| HNRNPK | 3190 | heterogeneous nuclear ribonucleoprotein K |
| HOXA10 | 3206 | homeobox A10 |
| HOXA13 | 3209 | homeobox A13 |
| HOXB7 | 3217 | homeobox B7 |
| HOXB9 | 3219 | homeobox B9 |
| HSF1 | 3297 | heat shock transcription factor 1 |
| HSP90AA1 | 3320 | heat shock protein 90 alpha family class A member 1 |
| ICAM1 | 3383 | intercellular adhesion molecule 1 |
| ID1 | 3397 | inhibitor of DNA binding 1, HLH protein |
| ID2 | 3398 | inhibitor of DNA binding 2 |
| ID3 | 3399 | inhibitor of DNA binding 3, HLH protein |
| IFNG | 3458 | interferon gamma |
| IFNL3 | 282617 | interferon lambda 3 |
| IGF1 | 3479 | insulin like growth factor 1 |
| IGF1R | 3480 | insulin like growth factor 1 receptor |
| IGF2 | 3481 | insulin like growth factor 2 |
| IGFBP3 | 3486 | insulin like growth factor binding protein 3 |
| IGFBP5 | 3488 | insulin like growth factor binding protein 5 |
| IGFBP7 | 3490 | insulin like growth factor binding protein 7 |
| IL10 | 3586 | interleukin 10 |
| IL11 | 3589 | interleukin 11 |
| IL13 | 3596 | interleukin 13 |
| IL15 | 3600 | interleukin 15 |
| IL17RD | 54756 | interleukin 17 receptor D |
| IL18 | 3606 | interleukin 18 |
| IL1A | 3552 | interleukin 1 alpha |
| IL1B | 3553 | interleukin 1 beta |
| IL21 | 59067 | interleukin 21 |
| IL23A | 51561 | interleukin 23 subunit alpha |
| IL2RA | 3559 | interleukin 2 receptor subunit alpha |
| IL32 | 9235 | interleukin 32 |
| IL4 | 3565 | interleukin 4 |
| IL6 | 3569 | interleukin 6 |
| ILF2 | 3608 | interleukin enhancer binding factor 2 |
| ILK | 3611 | integrin linked kinase |
| IMMP2L | 83943 | inner mitochondrial membrane peptidase subunit 2 |
| ING4 | 51147 | inhibitor of growth family member 4 |
| INS | 3630 | insulin |
| INSR | 3643 | insulin receptor |
| IRAK1 | 3654 | interleukin 1 receptor associated kinase 1 |
| IRF1 | 3659 | interferon regulatory factor 1 |
| IRF2 | 3660 | interferon regulatory factor 2 |
| IRF3 | 3661 | interferon regulatory factor 3 |
| IRF4 | 3662 | interferon regulatory factor 4 |
| IRF5 | 3663 | interferon regulatory factor 5 |
| IRF6 | 3664 | interferon regulatory factor 6 |
| IRF9 | 10379 | interferon regulatory factor 9 |
| ITGA1 | 3672 | integrin subunit alpha 1 |
| ITGA2 | 3673 | integrin subunit alpha 2 |
| ITGA4 | 3676 | integrin subunit alpha 4 |
| ITGA5 | 3678 | integrin subunit alpha 5 |
| ITGA6 | 3655 | integrin subunit alpha 6 |
| ITGA9 | 3680 | integrin subunit alpha 9 |
| ITGAM | 3684 | integrin subunit alpha M |
| ITGB1 | 3688 | integrin subunit beta 1 |
| ITGB3 | 3690 | integrin subunit beta 3 |
| ITGB4 | 3691 | integrin subunit beta 4 |
| ITGB6 | 3694 | integrin subunit beta 6 |
| ITK | 3702 | IL2 inducible T cell kinase |
| JAG1 | 182 | jagged 1 |
| JAK1 | 3716 | Janus kinase 1 |
| JAK2 | 3717 | Janus kinase 2 |
| JUN | 3725 | Jun proto-oncogene, AP-1 transcription factor subunit |
| JUNB | 3726 | JunB proto-oncogene, AP-1 transcription factor subunit |
| KCTD11 | 147040 | potassium channel tetramerization domain containing 11 |
| KDM1A | 23028 | lysine demethylase 1A |
| KDM4C | 23081 | lysine demethylase 4C |
| KDM5A | 5927 | lysine demethylase 5A |
| KDM5B | 10765 | lysine demethylase 5B |
| KDM5C | 8242 | lysine demethylase 5C |
| KDM6B | 23135 | lysine demethylase 6B |
| KDM8 | 79831 | lysine demethylase 8 |
| KDR | 3791 | kinase insert domain receptor |
| KHDRBS1 | 10657 | KH RNA binding domain containing, signal transduction associated 1 |
| KIF16B | 55614 | kinesin family member 16B |
| KIT | 3815 | KIT proto-oncogene receptor tyrosine kinase |
| KLF11 | 8462 | Kruppel like factor 11 |
| KLF17 | 128209 | Kruppel like factor 17 |
| KLF4 | 9314 | Kruppel like factor 4 |
| KLF5 | 688 | Kruppel like factor 5 |
| KLF8 | 11279 | Kruppel like factor 8 |
| KMT2B | 9757 | lysine methyltransferase 2B |
| KMT2D | 8085 | lysine methyltransferase 2D |
| KRAS | 3845 | KRAS proto-oncogene, GTPase |
| KRT14 | 3861 | keratin 14 |
| KRT18 | 3875 | keratin 18 |
| KRT19 | 3880 | keratin 19 |
| KRT7 | 3855 | keratin 7 |
| KRT8 | 3856 | keratin 8 |
| L1CAM | 3897 | L1 cell adhesion molecule |
| L1TD1 | 54596 | LINE1 type transposase domain containing 1 |
| LAMB1 | 3912 | laminin subunit beta 1 |
| LAMC2 | 3918 | laminin subunit gamma 2 |
| LATS1 | 9113 | large tumor suppressor kinase 1 |
| LATS2 | 26524 | large tumor suppressor kinase 2 |
| LBX1 | 10660 | ladybird homeobox 1 |
| LCK | 3932 | LCK proto-oncogene, Src family tyrosine kinase |
| LCN2 | 3934 | lipocalin 2 |
| LEF1 | 51176 | lymphoid enhancer binding factor 1 |
| LEPR | 3953 | leptin receptor |
| LGALS1 | 3956 | galectin 1 |
| LGALS3 | 3958 | galectin 3 |
| LGALS4 | 3960 | galectin 4 |
| LGALS9 | 3965 | galectin 9 |
| LGR4 | 55366 | leucine rich repeat containing G protein-coupled receptor 4 |
| LGR5 | 8549 | leucine rich repeat containing G protein-coupled receptor 5 |
| LHX2 | 9355 | LIM homeobox 2 |
| LIN28A | 79727 | lin-28 homolog A |
| LLGL1 | 3996 | LLGL1, scribble cell polarity complex component |
| LMO4 | 8543 | LIM domain only 4 |
| LMX1A | 4009 | LIM homeobox transcription factor 1 alpha |
| LOX | 4015 | lysyl oxidase |
| LRIG1 | 26018 | leucine rich repeats and immunoglobulin like domains 1 |
| LRP1 | 4035 | LDL receptor related protein 1 |
| LSR | 51599 | lipolysis stimulated lipoprotein receptor |
| LYN | 4067 | LYN proto-oncogene, Src family tyrosine kinase |
| LYPD3 | 27076 | LY6/PLAUR domain containing 3 |
| MACC1 | 346389 | MACC1, MET transcriptional regulator |
| MAL2 | 114569 | mal, T cell differentiation protein 2 (gene/pseudogene) |
| MAML2 | 84441 | mastermind like transcriptional coactivator 2 |
| MAML3 | 55534 | mastermind like transcriptional coactivator 3 |
| MAP2K1 | 5604 | mitogen-activated protein kinase kinase 1 |
| MAP2K2 | 5605 | mitogen-activated protein kinase kinase 2 |
| MAP3K4 | 4216 | mitogen-activated protein kinase kinase kinase 4 |
| MAP3K7 | 6885 | mitogen-activated protein kinase kinase kinase 7 |
| MAPK14 | 1432 | mitogen-activated protein kinase 14 |
| MAPK3 | 5595 | mitogen-activated protein kinase 3 |
| MAPK8 | 5599 | mitogen-activated protein kinase 8 |
| MAPT | 4137 | microtubule associated protein tau |
| MCAM | 4162 | melanoma cell adhesion molecule |
| MCL1 | 4170 | MCL1, BCL2 family apoptosis regulator |
| MDC1 | 9656 | mediator of DNA damage checkpoint 1 |
| MDM2 | 4193 | MDM2 proto-oncogene |
| MDM4 | 4194 | MDM4, p53 regulator |
| MECOM | 2122 | MDS1 and EVI1 complex locus |
| MED1 | 5469 | mediator complex subunit 1 |
| MED30 | 90390 | mediator complex subunit 30 |
| MEFV | 4210 | MEFV, pyrin innate immunity regulator |
| MEG3 | 55384 | maternally expressed 3 (non-protein coding) |
| MEMO1 | 7795 | Methylation modifier for class I HLA |
| MEOX2 | 4223 | mesenchyme homeobox 2 |
| MET | 4233 | MET proto-oncogene, receptor tyrosine kinase |
| MGAT5 | 4249 | mannosyl (alpha-1,6-)-glycoprotein beta-1,6-N-acetyl-glucosaminyltransferase |
| MIF | 4282 | macrophage migration inhibitory factor |
| MIR101-1 | 406893 | microRNA 101-1 |
| MIR101-2 | 406894 | microRNA 101-2 |
| MIR141 | 406933 | microRNA 141 |
| MIR200A | 406983 | microRNA 200a |
| MIR200B | 406984 | microRNA 200b |
| MIR200C | 406985 | microRNA 200c |
| MIR205 | 406988 | microRNA 205 |
| MIR21 | 406991 | microRNA 21 |
| MIR30C1 | 407031 | microRNA 30c-1 |
| MIR34A | 407040 | microRNA 34a |
| MIR34B | 407041 | microRNA 34b |
| MIR34C | 407042 | microRNA 34c |
| MIR429 | 554210 | microRNA 429 |
| MIR7-1 | 407043 | microRNA 7-1 |
| MIR7-2 | 407044 | microRNA 7-2 |
| MIR7-3 | 407045 | microRNA 7-3 |
| MIR9-1 | 407046 | microRNA 9-1 |
| MIR9-2 | 407047 | microRNA 9-2 |
| MIR9-3 | 407051 | microRNA 9-3 |
| MIRLET7A1 | 406881 | microRNA let-7a-1 |
| MKI67 | 4288 | marker of proliferation Ki-67 |
| MLH1 | 4292 | mutL homolog 1 |
| MME | 4311 | membrane metalloendopeptidase |
| MMP1 | 4312 | matrix metallopeptidase 1 |
| MMP2 | 4313 | matrix metallopeptidase 2 |
| MMP3 | 4314 | matrix metallopeptidase 3 |
| MMP9 | 4318 | matrix metallopeptidase 9 |
| MPO | 4353 | myeloperoxidase |
| MPZL2 | 10205 | myelin protein zero like 2 |
| MS4A3 | 932 | membrane spanning 4-domains A3 |
| MSGN1 | 343930 | mesogenin 1 |
| MSH3 | 4437 | mutS homolog 3 |
| MSH6 | 2956 | mutS homolog 6 |
| MST1 | 4485 | macrophage stimulating 1 |
| MSX1 | 4487 | msh homeobox 1 |
| MSX2 | 4488 | msh homeobox 2 |
| MTA1 | 9112 | metastasis associated 1 |
| MTA2 | 9219 | metastasis associated 1 family member 2 |
| MTA3 | 57504 | metastasis associated 1 family member 3 |
| MTOR | 2475 | mechanistic target of rapamycin kinase |
| MTSS1 | 9788 | MTSS1, I-BAR domain containing |
| MUC1 | 4582 | mucin 1, cell surface associated |
| MUC16 | 94025 | mucin 16, cell surface associated |
| MUC4 | 4585 | mucin 4, cell surface associated |
| MYBL2 | 4605 | MYB proto-oncogene like 2 |
| MYC | 4609 | MYC proto-oncogene, bHLH transcription factor |
| MYCN | 4613 | MYCN proto-oncogene, bHLH transcription factor |
| MYH11 | 4629 | myosin heavy chain 11 |
| MYL2 | 4633 | myosin light chain 2 |
| NACC1 | 112939 | nucleus accumbens associated 1 |
| NANOG | 79923 | Nanog homeobox |
| NBN | 4683 | nibrin |
| NCAM1 | 4684 | neural cell adhesion molecule 1 |
| NCF1 | 653361 | neutrophil cytosolic factor 1 |
| NCL | 4691 | nucleolin |
| NCOA3 | 8202 | nuclear receptor coactivator 3 |
| NDRG1 | 10397 | N-myc downstream regulated 1 |
| NDUFS3 | 4722 | NADH:ubiquinone oxidoreductase core subunit S3 |
| NEDD9 | 4739 | neural precursor cell expressed, developmentally down-regulated 9 |
| NET1 | 10276 | neuroepithelial cell transforming 1 |
| NFE2L2 | 4780 | nuclear factor, erythroid 2 like 2 |
| NFKB1 | 4790 | nuclear factor kappa B subunit 1 |
| NFKB2 | 4791 | nuclear factor kappa B subunit 2 |
| NGF | 4803 | nerve growth factor |
| NGFR | 4804 | nerve growth factor receptor |
| NHEJ1 | 79840 | non-homologous end joining factor 1 |
| NID1 | 4811 | nidogen 1 |
| NKX6-1 | 4825 | NK6 homeobox 1 |
| NME1 | 4830 | NME/NM23 nucleoside diphosphate kinase 1 |
| NOP56 | 10528 | NOP56 ribonucleoprotein |
| NOS2 | 4843 | nitric oxide synthase 2 |
| NOTCH1 | 4851 | notch 1 |
| NOTCH2 | 4853 | notch 2 |
| NOX1 | 27035 | NADPH oxidase 1 |
| NOX4 | 50507 | NADPH oxidase 4 |
| NR1H4 | 9971 | nuclear receptor subfamily 1 group H member 4 |
| NR4A1 | 3164 | nuclear receptor subfamily 4 group A member 1 |
| NR5A1 | 2516 | nuclear receptor subfamily 5 group A member 1 |
| NRP2 | 8828 | neuropilin 2 |
| NT5E | 4907 | 5'-nucleotidase ecto |
| NTF3 | 4908 | neurotrophin 3 |
| NUDT1 | 4521 | nudix hydrolase 1 |
| NUMB | 8650 | NUMB, endocytic adaptor protein |
| OCLN | 100506658 | occludin |
| OLA1 | 29789 | Obg like ATPase 1 |
| OLFM4 | 10562 | olfactomedin 4 |
| OLIG1 | 116448 | oligodendrocyte transcription factor 1 |
| ORAI1 | 84876 | ORAI calcium release-activated calcium modulator 1 |
| OSM | 5008 | oncostatin M |
| OSR1 | 130497 | odd-skipped related transciption factor 1 |
| OVOL1 | 5017 | ovo like transcriptional repressor 1 |
| OVOL2 | 58495 | ovo like zinc finger 2 |
| PAK1 | 5058 | p21 (RAC1) activated kinase 1 |
| PAK6 | 56924 | p21 (RAC1) activated kinase 6 |
| PARK7 | 11315 | Parkinsonism associated deglycase |
| PBXIP1 | 57326 | PBX homeobox interacting protein 1 |
| PECAM1 | 5175 | platelet and endothelial cell adhesion molecule 1 |
| PIK3R1 | 5295 | phosphoinositide-3-kinase regulatory subunit 1 |
| PITPNM3 | 83394 | PITPNM family member 3 |
| PKP2 | 5318 | plakophilin 2 |
| POLD3 | 10714 | DNA polymerase delta 3, accessory subunit |
| POLR2C | 5432 | RNA polymerase II subunit C |
| POU5F1 | 5460 | POU class 5 homeobox 1 |
| PRDM1 | 639 | PR/SET domain 1 |
| PROM1 | 8842 | prominin 1 |
| PRRX1 | 5396 | paired related homeobox 1 |
| PSMC6 | 5706 | proteasome 26S subunit, ATPase 6 |
| PTGER2 | 5732 | prostaglandin E receptor 2 |
| PTGER4 | 5734 | prostaglandin E receptor 4 |
| PTGS1 | 5742 | prostaglandin-endoperoxide synthase 1 |
| PTGS2 | 5743 | prostaglandin-endoperoxide synthase 2 |
| PTK2 | 5747 | protein tyrosine kinase 2 |
| PTPN11 | 5781 | protein tyrosine phosphatase, non-receptor type 11 |
| PVRL1 | NA | NA |
| PVRL4 | NA | NA |
| PXN | 5829 | paxillin |
| RAB1A | 5861 | RAB1A, member RAS oncogene family |
| RAB25 | 57111 | RAB25, member RAS oncogene family |
| RAB31 | 11031 | RAB31, member RAS oncogene family |
| RAB5A | 5868 | RAB5A, member RAS oncogene family |
| RAB7A | 7879 | RAB7A, member RAS oncogene family |
| RAC1 | 5879 | Rac family small GTPase 1 |
| RAD17 | 5884 | RAD17 checkpoint clamp loader component |
| RAD52 | 5893 | RAD52 homolog, DNA repair protein |
| RAF1 | 5894 | Raf-1 proto-oncogene, serine/threonine kinase |
| RALB | 5899 | RAS like proto-oncogene B |
| RAN | 5901 | RAN, member RAS oncogene family |
| RAP1A | 5906 | RAP1A, member of RAS oncogene family |
| RAPH1 | 65059 | Ras association (RalGDS/AF-6) and pleckstrin homology domains 1 |
| RB1 | 5925 | RB transcriptional corepressor 1 |
| RBL1 | 5933 | RB transcriptional corepressor like 1 |
| RBL2 | 5934 | RB transcriptional corepressor like 2 |
| RECQL | 5965 | RecQ like helicase |
| REL | 5966 | REL proto-oncogene, NF-kB subunit |
| RELA | 5970 | RELA proto-oncogene, NF-kB subunit |
| RELB | 5971 | RELB proto-oncogene, NF-kB subunit |
| RFX2 | 5990 | regulatory factor X2 |
| RHOA | 387 | ras homolog family member A |
| RNASE3 | 6037 | ribonuclease A family member 3 |
| RND1 | 27289 | Rho family GTPase 1 |
| RND3 | 390 | Rho family GTPase 3 |
| RNF38 | 152006 | ring finger protein 38 |
| RNF8 | 9025 | ring finger protein 8 |
| ROBO1 | 6091 | roundabout guidance receptor 1 |
| ROCK1 | 6093 | Rho associated coiled-coil containing protein kinase 1 |
| ROCK2 | 9475 | Rho associated coiled-coil containing protein kinase 2 |
| ROMO1 | 140823 | reactive oxygen species modulator 1 |
| ROR1 | 4919 | receptor tyrosine kinase like orphan receptor 1 |
| ROR2 | 4920 | receptor tyrosine kinase like orphan receptor 2 |
| RPS6KB1 | 6198 | ribosomal protein S6 kinase B1 |
| RUNX1 | 861 | runt related transcription factor 1 |
| RUNX2 | 860 | runt related transcription factor 2 |
| RUNX3 | 864 | runt related transcription factor 3 |
| RXRA | 6256 | retinoid X receptor alpha |
| RXRB | 6257 | retinoid X receptor beta |
| S100A10 | 6281 | S100 calcium binding protein A10 |
| S100A11 | 6282 | S100 calcium binding protein A11 |
| S100A4 | 6275 | S100 calcium binding protein A4 |
| S100B | 6285 | S100 calcium binding protein B |
| SALL4 | 57167 | spalt like transcription factor 4 |
| SAPCD2 | 89958 | suppressor APC domain containing 2 |
| SATB1 | 6304 | SATB homeobox 1 |
| SCD | 6319 | stearoyl-CoA desaturase |
| SCD5 | 79966 | stearoyl-CoA desaturase 5 |
| SDC1 | 6382 | syndecan 1 |
| SDC2 | 6383 | syndecan 2 |
| SDC3 | 9672 | syndecan 3 |
| SDCBP | 6386 | syndecan binding protein |
| SELE | 6401 | selectin E |
| SERPINB3 | 6317 | serpin family B member 3 |
| SESN1 | 27244 | sestrin 1 |
| SESN2 | 83667 | sestrin 2 |
| SETD2 | 29072 | SET domain containing 2 |
| SETD8 | NA | NA |
| SHC1 | 6464 | SHC adaptor protein 1 |
| SIAH1 | 6477 | siah E3 ubiquitin protein ligase 1 |
| SIK1 | 150094 | salt inducible kinase 1 |
| SIK2 | 23235 | salt inducible kinase 2 |
| SIVA1 | 10572 | SIVA1 apoptosis inducing factor |
| SIX1 | 6495 | SIX homeobox 1 |
| SKP2 | 6502 | S-phase kinase associated protein 2 |
| SLC39A10 | 57181 | solute carrier family 39 member 10 |
| SLC7A5 | 8140 | solute carrier family 7 member 5 |
| SLIT2 | 9353 | slit guidance ligand 2 |
| SMAD2 | 4087 | SMAD family member 2 |
| SMAD3 | 4088 | SMAD family member 3 |
| SMAD4 | 4089 | SMAD family member 4 |
| SMARCC1 | 6599 | SWI/SNF related, matrix associated, actin dependent regulator of chromatin subfamily c member 1 |
| SMC1A | 8243 | structural maintenance of chromosomes 1A |
| SMEK1 | NA | NA |
| SMO | 6608 | smoothened, frizzled class receptor |
| SMURF2 | 64750 | SMAD specific E3 ubiquitin protein ligase 2 |
| SNAI1 | 6615 | snail family transcriptional repressor 1 |
| SNAI2 | 6591 | snail family transcriptional repressor 2 |
| SND1 | 27044 | staphylococcal nuclease and tudor domain containing 1 |
| SOCS1 | 8651 | suppressor of cytokine signaling 1 |
| SOCS2 | 8835 | suppressor of cytokine signaling 2 |
| SOCS3 | 9021 | suppressor of cytokine signaling 3 |
| SOD1 | 6647 | superoxide dismutase 1 |
| SOD2 | 6648 | superoxide dismutase 2 |
| SOS1 | 6654 | SOS Ras/Rac guanine nucleotide exchange factor 1 |
| SOX10 | 6663 | SRY-box 10 |
| SOX12 | 6666 | SRY-box 12 |
| SOX17 | 64321 | SRY-box 17 |
| SOX2 | 6657 | SRY-box 2 |
| SOX3 | 6658 | SRY-box 3 |
| SOX5 | 6660 | SRY-box 5 |
| SOX9 | 6662 | SRY-box 9 |
| SP1 | 6667 | Sp1 transcription factor |
| SP3 | 6670 | Sp3 transcription factor |
| SPHK1 | 8877 | sphingosine kinase 1 |
| SPINT1 | 6692 | serine peptidase inhibitor, Kunitz type 1 |
| SPP1 | 6696 | secreted phosphoprotein 1 |
| SPRED2 | 200734 | sprouty related EVH1 domain containing 2 |
| SPRY1 | 10252 | sprouty RTK signaling antagonist 1 |
| SPRY2 | 10253 | sprouty RTK signaling antagonist 2 |
| SPRY4 | 81848 | sprouty RTK signaling antagonist 4 |
| SQSTM1 | 8878 | sequestosome 1 |
| SRC | 6714 | SRC proto-oncogene, non-receptor tyrosine kinase |
| SREBF2 | 6721 | sterol regulatory element binding transcription factor 2 |
| SRF | 6722 | serum response factor |
| SSX2 | 6757 | SSX family member 2 |
| STAT3 | 6774 | signal transducer and activator of transcription 3 |
| STAT5A | 6776 | signal transducer and activator of transcription 5A |
| STAT5B | 6777 | signal transducer and activator of transcription 5B |
| STK11 | 6794 | serine/threonine kinase 11 |
| SYK | 6850 | spleen associated tyrosine kinase |
| TAB1 | 10454 | TGF-beta activated kinase 1 (MAP3K7) binding protein 1 |
| TAOK2 | 9344 | TAO kinase 2 |
| TBK1 | 29110 | TANK binding kinase 1 |
| TBX2 | 6909 | T-box 2 |
| TCF3 | 6929 | transcription factor 3 |
| TCF4 | 6925 | transcription factor 4 |
| TERF2 | 7014 | telomeric repeat binding factor 2 |
| TGFA | 7039 | transforming growth factor alpha |
| TGFB1 | 7040 | transforming growth factor beta 1 |
| TGFB1I1 | 7041 | transforming growth factor beta 1 induced transcript 1 |
| TGFB2 | 7042 | transforming growth factor beta 2 |
| TGFB3 | 7043 | transforming growth factor beta 3 |
| TGFBR1 | 7046 | transforming growth factor beta receptor 1 |
| TGM2 | 7052 | transglutaminase 2 |
| THBD | 7056 | thrombomodulin |
| THBS1 | 7057 | thrombospondin 1 |
| TIMP2 | 7077 | TIMP metallopeptidase inhibitor 2 |
| TIMP4 | 7079 | TIMP metallopeptidase inhibitor 4 |
| TJP1 | 7082 | tight junction protein 1 |
| TJP2 | 9414 | tight junction protein 2 |
| TJP3 | 27134 | tight junction protein 3 |
| TNC | 3371 | tenascin C |
| TNF | 7124 | tumor necrosis factor |
| TNFSF13B | 10673 | TNF superfamily member 13b |
| TNK2 | 10188 | tyrosine kinase non receptor 2 |
| TOP1 | 7150 | DNA topoisomerase I |
| TOP2A | 7153 | DNA topoisomerase II alpha |
| TP53 | 7157 | tumor protein p53 |
| TP63 | 8626 | tumor protein p63 |
| TP73 | 7161 | tumor protein p73 |
| TTF1 | 7270 | transcription termination factor 1 |
| TWIST1 | 7291 | twist family bHLH transcription factor 1 |
| TWIST2 | 117581 | twist family bHLH transcription factor 2 |
| TXN | 7295 | thioredoxin |
| TXN2 | 25828 | thioredoxin 2 |
| UBQLN1 | 29979 | ubiquilin 1 |
| UBXN2B | 137886 | UBX domain protein 2B |
| UCA1 | 652995 | urothelial cancer associated 1 (non-protein coding) |
| VCAM1 | 7412 | vascular cell adhesion molecule 1 |
| VDR | 7421 | vitamin D receptor |
| VEGFA | 7422 | vascular endothelial growth factor A |
| VEGFB | 7423 | vascular endothelial growth factor B |
| VEGFC | 7424 | vascular endothelial growth factor C |
| VHL | 7428 | von Hippel-Lindau tumor suppressor |
| VIM | 7431 | vimentin |
| VMP1 | 81671 | vacuole membrane protein 1 |
| VRK1 | 7443 | vaccinia related kinase 1 |
| VTN | 7448 | vitronectin |
| WDR5 | 11091 | WD repeat domain 5 |
| WDR66 | 144406 | WD repeat domain 66 |
| WIF1 | 11197 | WNT inhibitory factor 1 |
| WISP2 | 8839 | WNT1 inducible signaling pathway protein 2 |
| WISP3 | 8838 | WNT1 inducible signaling pathway protein 3 |
| WNK1 | 65125 | WNK lysine deficient protein kinase 1 |
| WNT1 | 7471 | Wnt family member 1 |
| WNT4 | 54361 | Wnt family member 4 |
| WNT5A | 7474 | Wnt family member 5A |
| WT1 | 7490 | Wilms tumor 1 |
| XRCC2 | 7516 | X-ray repair cross complementing 2 |
| XRCC4 | 7518 | X-ray repair cross complementing 4 |
| XRN1 | 54464 | 5'-3' exoribonuclease 1 |
| XRN2 | 22803 | 5'-3' exoribonuclease 2 |
| YAP1 | 10413 | Yes associated protein 1 |
| YY1 | 7528 | YY1 transcription factor |
| ZEB1 | 6935 | zinc finger E-box binding homeobox 1 |
| ZEB2 | 9839 | zinc finger E-box binding homeobox 2 |
