# Supplementary material for: Multi-omics analysis reveals epithelial-mesenchymal transition-related gene FOXM1 as a novel prognostic biomarker in clear cell renal carcinoma
Source: Aging (Albany NY). 2019 Nov 19;11(22):10316–37. doi: 10.18632/aging.102459 (PMC6914426; doi:10.18632/aging.102459)
Supplement: Supplementary Table 4 [file aging-11-102459-s007..docx]

| Supplementary Table 4. The patients and corresponding clinical information of GDC miRNA-Seq dataset (n=615) | | | | | | | | | | | | | |
| --- | --- | --- | --- | --- | --- | --- | --- | --- | --- | --- | --- | --- | --- |
| PATIENT_ID | SAMPLE | CANCER | GENDER | AGE | AJCC_PT | AJCC_PN | AJCC_PM | AJCC_STAGE | GRADE | OS_STATUS | OS_MONTHS | DFS_STATUS | DFS_MONTHS |
| TCGA-CZ-5470 | TCGA-CZ-5470-01A-01R-1502-13 | KIRC | FEMALE | 72 | T2 | N0 | M0 | Stage II | G3 | LIVING | 12.68 | DiseaseFree | 12.68 |
| TCGA-BP-4989 | TCGA-BP-4989-01A-01R-1333-13 | KIRC | MALE | 58 | T3a | N0 | M0 | Stage III | G3 | LIVING | 3.88 | DiseaseFree | 3.88 |
| TCGA-CJ-6027 | TCGA-CJ-6027-01A-11R-1671-13 | KIRC | MALE | 77 | T1a | NX | M0 | Stage I | G4 | DECEASED | 118.76 | Unknown | Unknown |
| TCGA-BP-4998 | TCGA-BP-4998-01A-01R-1333-13 | KIRC | MALE | 49 | T1a | NX | M0 | Stage I | G3 | LIVING | 30.62 | DiseaseFree | 30.62 |
| TCGA-BP-4762 | TCGA-BP-4762-01A-02R-1288-13 | KIRC | MALE | 42 | T1a | NX | M0 | Stage I | G3 | DECEASED | 44.12 | Unknown | Unknown |
| TCGA-B8-A54E | TCGA-B8-A54E-01A-11R-A25Z-13 | KIRC | FEMALE | 62 | T1b | NX | MX | Stage I | G3 | LIVING | 29.86 | DiseaseFree | 29.86 |
| TCGA-B8-4619 | TCGA-B8-4619-01A-01R-1187-13 | KIRC | MALE | 58 | T1a | N0 | M0 | Stage I | G2 | LIVING | 17.18 | DiseaseFree | 17.18 |
| TCGA-CZ-5462 | TCGA-CZ-5462-01A-01R-1502-13 | KIRC | MALE | 83 | T1b | NX | M1 | Stage IV | G3 | DECEASED | 10.22 | Unknown | Unknown |
| TCGA-BP-4801 | TCGA-BP-4801-01A-02R-1419-13 | KIRC | MALE | 57 | T1a | NX | M0 | Stage I | G2 | LIVING | 36.93 | DiseaseFree | 36.93 |
| TCGA-BP-4347 | TCGA-BP-4347-01A-01R-1288-13 | KIRC | MALE | 74 | T3b | NX | M0 | Stage III | G2 | LIVING | 44.91 | DiseaseFree | 44.91 |
| TCGA-B0-4699 | TCGA-B0-4699-01A-01R-1276-13 | KIRC | MALE | 74 | T4 | N0 | M1 | Stage IV | G4 | DECEASED | 3.61 | Unknown | Unknown |
| TCGA-BP-5007 | TCGA-BP-5007-01A-01R-1762-13 | KIRC | MALE | 45 | T2 | N0 | M0 | Stage II | G2 | LIVING | 37.45 | DiseaseFree | 37.45 |
| TCGA-A3-3376 | TCGA-A3-3376-01A-02R-1419-13 | KIRC | MALE | 51 | T1a | N0 | M0 | Stage I | G2 | DECEASED | 55.72 | Unknown | Unknown |
| TCGA-A3-3365 | TCGA-A3-3365-01A-01T-0860-13 | KIRC | MALE | 46 | T1a | NX | M0 | Stage I | G2 | LIVING | 28.68 | DiseaseFree | 28.68 |
| TCGA-6D-AA2E | TCGA-6D-AA2E-01A-11R-A37P-13 | KIRC | FEMALE | 68 | T1b | NX | MX | Stage I | G2 | LIVING | 11.89 | DiseaseFree | 11.89 |
| TCGA-DV-5575 | TCGA-DV-5575-01A-01R-1540-13 | KIRC | FEMALE | 52 | T1a | NX | M0 | Stage I | G2 | LIVING | 56.8 | DiseaseFree | 56.8 |
| TCGA-B0-4815 | TCGA-B0-4815-01A-01R-1502-13 | KIRC | MALE | 65 | T3a | NX | M0 | Stage III | G4 | DECEASED | 52.17 | Unknown | Unknown |
| TCGA-CJ-4894 | TCGA-CJ-4894-01A-01R-1304-13 | KIRC | MALE | 58 | T3a | N0 | M0 | Stage III | G3 | DECEASED | 27.63 | Recurred/Progressed | 5.19 |
| TCGA-MM-A84U | TCGA-MM-A84U-01A-11R-A37P-13 | KIRC | FEMALE | 58 | T1a | NX | MX | Stage I | G2 | LIVING | 23 | DiseaseFree | 23 |
| TCGA-CJ-4885 | TCGA-CJ-4885-01A-01R-1304-13 | KIRC | MALE | 64 | T3a | NX | M1 | Stage IV | G3 | LIVING | 113.37 | Recurred/Progressed | 67.05 |
| TCGA-CZ-5465 | TCGA-CZ-5465-01A-01R-1502-13 | KIRC | FEMALE | 76 | T3b | NX | M0 | Stage III | G2 | DECEASED | 84.23 | Unknown | Unknown |
| TCGA-BP-4353 | TCGA-BP-4353-01A-02R-1288-13 | KIRC | MALE | 61 | T1 | N0 | M0 | Stage I | G2 | DECEASED | 12.32 | Unknown | Unknown |
| TCGA-CW-5589 | TCGA-CW-5589-01A-01R-1540-13 | KIRC | MALE | 52 | T1a | NX | M0 | Stage I | G2 | LIVING | 78.12 | DiseaseFree | 78.12 |
| TCGA-B8-A54D | TCGA-B8-A54D-01A-21R-A25Z-13 | KIRC | MALE | 69 | T3a | NX | MX | Stage III | G2 | LIVING | 27.27 | DiseaseFree | 27.27 |
| TCGA-B8-4621 | TCGA-B8-4621-01A-01R-1502-13 | KIRC | MALE | 63 | T1b | N0 | M0 | Stage I | G3 | LIVING | 25.89 | DiseaseFree | 25.89 |
| TCGA-B0-4701 | TCGA-B0-4701-01A-01R-1276-13 | KIRC | FEMALE | 66 | T3a | N0 | M1 | Stage IV | G3 | DECEASED | 7.82 | Unknown | Unknown |
| TCGA-B8-A54K | TCGA-B8-A54K-01A-11R-A33A-13 | KIRC | MALE | 61 | T1a | NX | MX | Stage I | G1 | LIVING | 15.41 | DiseaseFree | 15.41 |
| TCGA-CJ-6030 | TCGA-CJ-6030-01A-11R-1671-13 | KIRC | MALE | 65 | T1a | N0 | M0 | Stage I | G3 | DECEASED | 75.53 | Unknown | Unknown |
| TCGA-BP-5009 | TCGA-BP-5009-01A-01R-1333-13 | KIRC | MALE | 52 | T1b | NX | M0 | Stage I | G3 | DECEASED | 35.87 | Recurred/Progressed | 16.16 |
| TCGA-BP-4795 | TCGA-BP-4795-01A-02R-1419-13 | KIRC | FEMALE | 74 | T1a | N0 | M0 | Stage I | G2 | LIVING | 20.37 | DiseaseFree | 20.37 |
| TCGA-DV-A4W0 | TCGA-DV-A4W0-01A-11R-A25Z-13 | KIRC | MALE | 55 | T1b | NX | MX | Stage I | G3 | LIVING | 81.14 | Recurred/Progressed | 65.97 |
| TCGA-A3-3311 | TCGA-A3-3311-01A-02R-1324-13 | KIRC | MALE | 57 | T1 | NX | M0 | Stage I | G2 | DECEASED | 39.13 | Unknown | Unknown |
| TCGA-AK-3465 | TCGA-AK-3465-01A-02R-1324-13 | KIRC | FEMALE | 71 | T1b | NX | M0 | Stage I | GX | LIVING | 12.12 | DiseaseFree | 12.12 |
| TCGA-CJ-4905 | TCGA-CJ-4905-01A-02R-1425-13 | KIRC | FEMALE | 62 | T1a | NX | M0 | Stage I | G2 | LIVING | 49.15 | DiseaseFree | 49.15 |
| TCGA-CW-6087 | TCGA-CW-6087-01A-11R-1671-13 | KIRC | MALE | 61 | T3a | N1 | M1 | Stage IV | G4 | DECEASED | 1.35 | Unknown | Unknown |
| TCGA-BP-5190 | TCGA-BP-5190-01A-01R-1425-13 | KIRC | MALE | 61 | T1a | NX | M0 | Stage I | G3 | LIVING | 33.21 | DiseaseFree | 33.21 |
| TCGA-CJ-4638 | TCGA-CJ-4638-01A-01R-1187-13 | KIRC | FEMALE | 46 | T3a | N1 | M1 | Stage IV | G4 | DECEASED | 14.16 | Recurred/Progressed | 2.3 |
| TCGA-B0-5077 | TCGA-B0-5077-01A-01R-1333-13 | KIRC | MALE | 77 | T1a | N0 | M0 | Stage I | G3 | DECEASED | 43.27 | Unknown | Unknown |
| TCGA-CJ-4878 | TCGA-CJ-4878-01A-01R-1304-13 | KIRC | FEMALE | 71 | T3a | NX | M0 | Stage III | G2 | LIVING | 71.81 | DiseaseFree | 71.81 |
| TCGA-BP-4965 | TCGA-BP-4965-01A-01R-1762-13 | KIRC | MALE | 46 | T1a | NX | M0 | Stage I | G2 | LIVING | 61.47 | DiseaseFree | 61.47 |
| TCGA-B0-5094 | TCGA-B0-5094-01A-01R-1419-13 | KIRC | MALE | 62 | T3b | N0 | M1 | Stage IV | G2 | DECEASED | 10.94 | Recurred/Progressed | 2.46 |
| TCGA-CZ-5985 | TCGA-CZ-5985-01A-11R-1671-13 | KIRC | MALE | 58 | T2 | N0 | M0 | Stage II | G2 | LIVING | 65.6 | DiseaseFree | 65.6 |
| TCGA-A3-3374 | TCGA-A3-3374-01A-02R-1324-13 | KIRC | FEMALE | 51 | T1b | N0 | M0 | Stage I | G2 | LIVING | 43.17 | DiseaseFree | 43.17 |
| TCGA-EU-5907 | TCGA-EU-5907-01A-11R-1671-13 | KIRC | MALE | 81 | T3a | NX | M0 | Stage III | G3 | LIVING | 4.17 | DiseaseFree | 4.17 |
| TCGA-B0-5693 | TCGA-B0-5693-01A-11R-1540-13 | KIRC | FEMALE | 47 | T1b | NX | M0 | Stage I | G2 | LIVING | 133.84 | DiseaseFree | 133.84 |
| TCGA-CZ-5460 | TCGA-CZ-5460-01A-01R-1502-13 | KIRC | MALE | 55 | T3b | NX | M1 | Stage IV | G2 | LIVING | 94.38 | DiseaseFree | 94.38 |
| TCGA-A3-3307 | TCGA-A3-3307-01A-01T-0860-13 | KIRC | MALE | 66 | T3b | N0 | M0 | Stage III | G3 | LIVING | 47.17 | DiseaseFree | 47.17 |
| TCGA-CZ-5463 | TCGA-CZ-5463-01A-01R-1502-13 | KIRC | MALE | 76 | T2 | NX | M0 | Stage II | G2 | LIVING | 21.75 | DiseaseFree | 21.75 |
| TCGA-AK-3427 | TCGA-AK-3427-01A-01T-0860-13 | KIRC | MALE | 65 | T1a | N0 | M0 | Stage I | GX | LIVING | 117.71 | DiseaseFree | 117.71 |
| TCGA-CJ-6028 | TCGA-CJ-6028-01A-11R-1671-13 | KIRC | MALE | 58 | T3a | NX | M1 | Stage IV | G4 | DECEASED | 53.38 | Recurred/Progressed | 4.5 |
| TCGA-A3-3372 | TCGA-A3-3372-01A-02R-1324-13 | KIRC | MALE | 64 | T3 | NX | M0 | Stage III | G2 | LIVING | 24.15 | DiseaseFree | 24.15 |
| TCGA-B8-5165 | TCGA-B8-5165-01A-01R-1419-13 | KIRC | MALE | 43 | T1a | N0 | M0 | Stage I | G2 | LIVING | 24.21 | DiseaseFree | 24.21 |
| TCGA-DV-5574 | TCGA-DV-5574-01A-01R-1540-13 | KIRC | MALE | 37 | T1a | NX | M0 | Stage I | G2 | LIVING | 66.23 | Recurred/Progressed | 11.73 |
| TCGA-BP-5178 | TCGA-BP-5178-01A-01R-1425-13 | KIRC | MALE | 71 | T3a | NX | M1 | Stage IV | G4 | DECEASED | 62.81 | Recurred/Progressed | 56.14 |
| TCGA-B4-5832 | TCGA-B4-5832-01A-11R-1671-13 | KIRC | MALE | 65 | T3b | N0 | M0 | Stage III | G2 | LIVING | 5.09 | DiseaseFree | 5.09 |
| TCGA-B0-4838 | TCGA-B0-4838-01A-01R-1304-13 | KIRC | FEMALE | 69 | T1b | N0 | M0 | Stage I | G3 | DECEASED | 27.4 | Unknown | Unknown |
| TCGA-CJ-5672 | TCGA-CJ-5672-01A-11R-1540-13 | KIRC | MALE | 84 | T1a | NX | M0 | Stage I | G3 | DECEASED | 71.94 | Unknown | Unknown |
| TCGA-A3-A8OX | TCGA-A3-A8OX-01A-11R-A37P-13 | KIRC | FEMALE | 65 | T1a | NX | MX | Stage I | G3 | LIVING | 0 | DiseaseFree | 0 |
| TCGA-BP-4338 | TCGA-BP-4338-01A-01R-1288-13 | KIRC | MALE | 43 | T1b | N0 | M0 | Stage I | G3 | LIVING | 93.92 | Recurred/Progressed | 84.49 |
| TCGA-BP-4983 | TCGA-BP-4983-01A-01R-1333-13 | KIRC | FEMALE | 67 | T3a | NX | M0 | Stage III | G4 | LIVING | 46.42 | DiseaseFree | 46.42 |
| TCGA-B8-5549 | TCGA-B8-5549-01A-01R-1540-13 | KIRC | MALE | 53 | T1b | N0 | M0 | Stage I | G3 | LIVING | 6.37 | DiseaseFree | 6.37 |
| TCGA-A3-3373 | TCGA-A3-3373-01A-02R-1419-13 | KIRC | FEMALE | 54 | T1b | N0 | M0 | Stage I | G3 | LIVING | 53.25 | DiseaseFree | 53.25 |
| TCGA-A3-A6NL | TCGA-A3-A6NL-01A-11R-A33A-13 | KIRC | FEMALE | 49 | T1b | NX | MX | Stage I | G2 | LIVING | 22.63 | DiseaseFree | 22.63 |
| TCGA-CJ-6031 | TCGA-CJ-6031-01A-11R-1671-13 | KIRC | MALE | 54 | T1b | NX | M0 | Stage I | G3 | LIVING | 62.61 | DiseaseFree | 62.61 |
| TCGA-BP-4768 | TCGA-BP-4768-01A-01R-1288-13 | KIRC | FEMALE | 72 | T1a | N0 | M0 | Stage I | G2 | LIVING | 13.14 | DiseaseFree | 13.14 |
| TCGA-CJ-4900 | TCGA-CJ-4900-01A-01R-1762-13 | KIRC | FEMALE | 69 | T4 | N1 | M1 | Stage IV | G4 | DECEASED | 56.31 | Unknown | Unknown |
| TCGA-DV-5576 | TCGA-DV-5576-01A-01R-1540-13 | KIRC | FEMALE | 55 | T1a | NX | M0 | Stage I | G2 | DECEASED | 23.88 | Unknown | Unknown |
| TCGA-AK-3434 | TCGA-AK-3434-01A-02R-1276-13 | KIRC | MALE | 72 | T1b | NX | M0 | Stage I | G2 | LIVING | 68.56 | Recurred/Progressed | 53.88 |
| TCGA-BP-4341 | TCGA-BP-4341-01A-01R-1288-13 | KIRC | MALE | 67 | T3a | NX | M0 | Stage III | G2 | DECEASED | 52.2 | Unknown | Unknown |
| TCGA-A3-3320 | TCGA-A3-3320-01A-01T-0860-13 | KIRC | FEMALE | 52 | T1b | NX | M0 | Stage I | G1 | LIVING | 49.54 | DiseaseFree | 49.54 |
| TCGA-BP-4790 | TCGA-BP-4790-01A-01R-1762-13 | KIRC | MALE | 76 | T1a | NX | M0 | Stage I | G2 | DECEASED | 36.5 | Unknown | Unknown |
| TCGA-DV-5573 | TCGA-DV-5573-01A-01R-1540-13 | KIRC | MALE | 41 | T1a | NX | M0 | Stage I | G2 | LIVING | 37.12 | DiseaseFree | 37.12 |
| TCGA-B8-A54I | TCGA-B8-A54I-01A-21R-A33A-13 | KIRC | MALE | 48 | T1b | NX | MX | Stage I | G3 | LIVING | 4.93 | DiseaseFree | 4.93 |
| TCGA-CZ-5461 | TCGA-CZ-5461-01A-01R-1502-13 | KIRC | MALE | 52 | T1b | NX | M1 | Stage IV | G4 | DECEASED | 10.84 | Recurred/Progressed | 1.58 |
| TCGA-B8-5164 | TCGA-B8-5164-01A-01R-1419-13 | KIRC | MALE | 65 | T3a | N0 | M0 | Stage III | G3 | LIVING | 0.85 | DiseaseFree | 0.85 |
| TCGA-AK-3428 | TCGA-AK-3428-01A-02R-1276-13 | KIRC | MALE | 62 | T3b | N0 | M0 | Stage III | G2 | LIVING | 122.47 | DiseaseFree | 122.47 |
| TCGA-CJ-4904 | TCGA-CJ-4904-01A-02R-1425-13 | KIRC | FEMALE | 60 | T3a | N0 | M1 | Stage IV | G3 | LIVING | 108.48 | Recurred/Progressed | 9.13 |
| TCGA-BP-5010 | TCGA-BP-5010-01A-02R-1419-13 | KIRC | MALE | 63 | T3a | N0 | M0 | Stage III | G4 | DECEASED | 28.84 | Recurred/Progressed | 25.66 |
| TCGA-CJ-4912 | TCGA-CJ-4912-01A-01R-1425-13 | KIRC | MALE | 61 | T2 | NX | M0 | Stage II | G3 | LIVING | 54.43 | DiseaseFree | 54.43 |
| TCGA-AS-3777 | TCGA-AS-3777-01A-01T-0860-13 | KIRC | MALE | 63 | T1a | NX | M0 | Stage I | Unknown | LIVING | 40.67 | DiseaseFree | 40.67 |
| TCGA-B0-4844 | TCGA-B0-4844-01A-01R-1276-13 | KIRC | MALE | 60 | T3a | NX | M1 | Stage IV | G3 | DECEASED | 10.28 | Recurred/Progressed | 2.04 |
| TCGA-BP-4766 | TCGA-BP-4766-01A-01R-1288-13 | KIRC | FEMALE | 43 | T1a | NX | M0 | Stage I | G3 | LIVING | 48.03 | DiseaseFree | 48.03 |
| TCGA-A3-3308 | TCGA-A3-3308-01A-02R-1324-13 | KIRC | FEMALE | 77 | T3b | N0 | M0 | Stage III | G2 | LIVING | 0.53 | DiseaseFree | 0.53 |
| TCGA-BP-5177 | TCGA-BP-5177-01A-01R-1425-13 | KIRC | FEMALE | 46 | T1a | NX | M0 | Stage I | G3 | LIVING | 9.63 | DiseaseFree | 9.63 |
| TCGA-CZ-5988 | TCGA-CZ-5988-01A-11R-1671-13 | KIRC | MALE | 38 | T1b | N0 | M0 | Stage I | G2 | LIVING | 22.77 | DiseaseFree | 22.77 |
| TCGA-B2-5635 | TCGA-B2-5635-01A-01R-A27D-13 | KIRC | MALE | 74 | T1a | NX | M0 | Stage I | G2 | LIVING | 24.8 | DiseaseFree | 24.8 |
| TCGA-B0-5075 | TCGA-B0-5075-01A-01R-1762-13 | KIRC | FEMALE | 77 | T3a | N0 | M0 | Stage III | G2 | DECEASED | 20.93 | Unknown | Unknown |
| TCGA-AK-3443 | TCGA-AK-3443-01A-02R-1324-13 | KIRC | MALE | 45 | T2 | N0 | M0 | Stage II | GX | LIVING | 46.75 | DiseaseFree | 46.75 |
| TCGA-CZ-4865 | TCGA-CZ-4865-01A-02R-1502-13 | KIRC | FEMALE | 70 | T1a | NX | M0 | Stage I | G2 | DECEASED | 5.45 | Unknown | Unknown |
| TCGA-BP-4325 | TCGA-BP-4325-01A-02R-1288-13 | KIRC | FEMALE | 64 | T1b | N0 | M0 | Stage I | G2 | LIVING | 97.37 | DiseaseFree | 97.37 |
| TCGA-CJ-5676 | TCGA-CJ-5676-01A-11R-1540-13 | KIRC | MALE | 47 | T3b | NX | M0 | Stage III | G3 | LIVING | 133.61 | Recurred/Progressed | 72.9 |
| TCGA-BP-5187 | TCGA-BP-5187-01A-01R-1425-13 | KIRC | MALE | 54 | T1a | NX | M0 | Stage I | G2 | LIVING | 13.34 | DiseaseFree | 13.34 |
| TCGA-BP-4803 | TCGA-BP-4803-01A-01R-1304-13 | KIRC | MALE | 79 | T3a | NX | M0 | Stage III | G3 | LIVING | 6.7 | DiseaseFree | 6.7 |
| TCGA-BP-4962 | TCGA-BP-4962-01A-01R-1333-13 | KIRC | MALE | 58 | T2 | NX | M0 | Stage II | G2 | LIVING | 58.64 | DiseaseFree | 58.64 |
| TCGA-A3-3331 | TCGA-A3-3331-01A-02R-1324-13 | KIRC | FEMALE | 86 | T1 | N0 | M0 | Stage I | G2 | LIVING | 48.78 | DiseaseFree | 48.78 |
| TCGA-BP-4992 | TCGA-BP-4992-01A-01R-1762-13 | KIRC | MALE | 66 | T1b | NX | M0 | Stage I | G4 | LIVING | 16.46 | DiseaseFree | 16.46 |
| TCGA-A3-3362 | TCGA-A3-3362-01A-02R-1324-13 | KIRC | FEMALE | 60 | T1a | N0 | M0 | Stage I | G2 | LIVING | 51.22 | DiseaseFree | 51.22 |
| TCGA-BP-4340 | TCGA-BP-4340-01A-01R-1762-13 | KIRC | FEMALE | 70 | T1b | N0 | M0 | Stage I | G2 | DECEASED | 18.46 | Unknown | Unknown |
| TCGA-CZ-4866 | TCGA-CZ-4866-01A-01R-1502-13 | KIRC | FEMALE | 79 | T1 | NX | M0 | Stage I | G3 | LIVING | 107.33 | DiseaseFree | 107.33 |
| TCGA-BP-4344 | TCGA-BP-4344-01A-01R-1288-13 | KIRC | FEMALE | 75 | T1a | NX | M0 | Stage I | G2 | LIVING | 54.73 | DiseaseFree | 54.73 |
| TCGA-B0-5098 | TCGA-B0-5098-01A-01R-1419-13 | KIRC | FEMALE | 53 | T1 | NX | M0 | Stage I | G3 | DECEASED | 52.04 | Unknown | Unknown |
| TCGA-A3-3313 | TCGA-A3-3313-01A-01T-0860-13 | KIRC | MALE | 59 | T1b | N0 | M0 | Stage I | G3 | DECEASED | 24.15 | Unknown | Unknown |
| TCGA-BP-4967 | TCGA-BP-4967-01A-01R-1333-13 | KIRC | MALE | 76 | T3a | N0 | M0 | Stage III | G2 | LIVING | 6.73 | DiseaseFree | 6.73 |
| TCGA-A3-3319 | TCGA-A3-3319-01A-01T-0860-13 | KIRC | MALE | 70 | T1b | NX | M0 | Stage I | G2 | LIVING | 37.12 | DiseaseFree | 37.12 |
| TCGA-CZ-5457 | TCGA-CZ-5457-01A-01R-1502-13 | KIRC | MALE | 62 | T3a | NX | M0 | Stage III | G4 | LIVING | 90.47 | Recurred/Progressed | 5.72 |
| TCGA-CW-6097 | TCGA-CW-6097-01A-11R-1671-13 | KIRC | MALE | 32 | T3a | NX | M0 | Stage III | G4 | DECEASED | 18.76 | Recurred/Progressed | 15.01 |
| TCGA-B0-5705 | TCGA-B0-5705-01A-11R-1540-13 | KIRC | FEMALE | 65 | T1 | N0 | M0 | Stage I | G2 | LIVING | 149.05 | Recurred/Progressed | 27.66 |
| TCGA-CZ-5468 | TCGA-CZ-5468-01A-01R-1502-13 | KIRC | MALE | 84 | T3b | NX | M1 | Stage IV | G4 | DECEASED | 1.94 | Unknown | Unknown |
| TCGA-BP-5198 | TCGA-BP-5198-01A-01R-1425-13 | KIRC | MALE | 72 | T3b | N0 | M0 | Stage III | G3 | LIVING | 19.81 | DiseaseFree | 19.81 |
| TCGA-BP-5182 | TCGA-BP-5182-01A-01R-1425-13 | KIRC | MALE | 56 | T1a | N0 | M0 | Stage I | G3 | LIVING | 38.27 | DiseaseFree | 38.27 |
| TCGA-AK-3425 | TCGA-AK-3425-01A-02R-1276-13 | KIRC | MALE | 68 | T1 | N0 | M0 | Stage I | G2 | LIVING | 109.82 | DiseaseFree | 109.82 |
| TCGA-B2-5633 | TCGA-B2-5633-01A-01R-1540-13 | KIRC | MALE | 56 | T1b | N0 | M0 | Stage I | G2 | LIVING | 31.64 | DiseaseFree | 31.64 |
| TCGA-B2-4102 | TCGA-B2-4102-01A-02R-1324-13 | KIRC | MALE | 61 | T1b | NX | M0 | Stage I | G2 | LIVING | 31.27 | DiseaseFree | 31.27 |
| TCGA-CW-6090 | TCGA-CW-6090-01A-11R-1671-13 | KIRC | MALE | 68 | T1b | NX | M0 | Stage I | G3 | LIVING | 83.84 | Recurred/Progressed | 91.33 |
| TCGA-CJ-4873 | TCGA-CJ-4873-01A-01R-1304-13 | KIRC | FEMALE | 85 | T3a | N0 | M0 | Stage III | G3 | LIVING | 74.21 | DiseaseFree | 74.21 |
| TCGA-BP-4342 | TCGA-BP-4342-01A-01R-1288-13 | KIRC | MALE | 79 | T2 | N0 | M0 | Stage II | G3 | DECEASED | 74.11 | Recurred/Progressed | 7.88 |
| TCGA-B0-4712 | TCGA-B0-4712-01A-01R-1502-13 | KIRC | MALE | 76 | T3a | NX | M1 | Stage IV | G3 | DECEASED | 43.92 | Unknown | Unknown |
| TCGA-B0-5081 | TCGA-B0-5081-01A-01R-1333-13 | KIRC | FEMALE | 79 | T3b | N0 | M0 | Stage III | G2 | DECEASED | 11.89 | Recurred/Progressed | 7 |
| TCGA-BP-5000 | TCGA-BP-5000-01A-01R-1762-13 | KIRC | MALE | 40 | T1b | NX | M0 | Stage I | G3 | LIVING | 18.5 | DiseaseFree | 18.5 |
| TCGA-CZ-5459 | TCGA-CZ-5459-01A-01R-1502-13 | KIRC | MALE | 63 | T3b | NX | M0 | Stage III | G3 | LIVING | 55.29 | DiseaseFree | 55.29 |
| TCGA-B0-5115 | TCGA-B0-5115-01A-01R-1419-13 | KIRC | MALE | 43 | T2 | N0 | M1 | Stage IV | G3 | LIVING | 52.69 | Recurred/Progressed | 2.46 |
| TCGA-EU-5906 | TCGA-EU-5906-01A-11R-1671-13 | KIRC | MALE | 55 | T1b | NX | M0 | Stage I | G2 | LIVING | 6.77 | DiseaseFree | 6.77 |
| TCGA-BP-4804 | TCGA-BP-4804-01A-02R-1762-13 | KIRC | MALE | 59 | T1b | NX | M0 | Stage I | G2 | LIVING | 47.93 | Recurred/Progressed | 41.72 |
| TCGA-A3-3382 | TCGA-A3-3382-01A-02R-1324-13 | KIRC | MALE | 69 | T1b | NX | M0 | Stage I | G3 | LIVING | 18.86 | Recurred/Progressed | 16.89 |
| TCGA-B0-5697 | TCGA-B0-5697-01A-11R-1540-13 | KIRC | MALE | 50 | T1a | N0 | M0 | Stage I | G2 | LIVING | 86.4 | DiseaseFree | 86.4 |
| TCGA-B0-4828 | TCGA-B0-4828-01A-01R-1276-13 | KIRC | MALE | 79 | T2 | NX | M1 | Stage IV | G3 | DECEASED | 10.09 | Unknown | Unknown |
| TCGA-A3-3320 | TCGA-A3-3320-01A-02R-1324-13 | KIRC | FEMALE | 52 | T1b | NX | M0 | Stage I | G1 | LIVING | 49.54 | DiseaseFree | 49.54 |
| TCGA-B0-4834 | TCGA-B0-4834-01A-01R-1762-13 | KIRC | MALE | 49 | T1a | N0 | M0 | Stage I | G3 | DECEASED | 68.66 | Unknown | Unknown |
| TCGA-BP-5195 | TCGA-BP-5195-01A-02R-1425-13 | KIRC | MALE | 75 | T1a | NX | M0 | Stage I | G2 | LIVING | 24.61 | DiseaseFree | 24.61 |
| TCGA-BP-4158 | TCGA-BP-4158-01A-02R-1288-13 | KIRC | MALE | 69 | T1b | N0 | M0 | Stage I | G2 | LIVING | 110.94 | DiseaseFree | 110.94 |
| TCGA-GK-A6C7 | TCGA-GK-A6C7-01A-11R-A33A-13 | KIRC | FEMALE | 76 | T1a | NX | MX | Stage I | Unknown | LIVING | 2 | DiseaseFree | 2 |
| TCGA-BP-4343 | TCGA-BP-4343-01A-02R-1288-13 | KIRC | MALE | 64 | T3a | N0 | M0 | Stage III | G3 | DECEASED | 62.81 | Recurred/Progressed | 61.63 |
| TCGA-BP-5191 | TCGA-BP-5191-01A-01R-1425-13 | KIRC | MALE | 79 | T3a | N0 | M0 | Stage III | G2 | LIVING | 31.77 | DiseaseFree | 31.77 |
| TCGA-AK-3431 | TCGA-AK-3431-01A-02R-1276-13 | KIRC | FEMALE | 62 | T2 | NX | M0 | Stage II | G3 | DECEASED | 73.62 | Unknown | Unknown |
| TCGA-BP-4987 | TCGA-BP-4987-01A-01R-1762-13 | KIRC | FEMALE | 41 | T1b | NX | M0 | Stage I | G2 | LIVING | 36.93 | DiseaseFree | 36.93 |
| TCGA-CZ-5466 | TCGA-CZ-5466-01A-01R-1502-13 | KIRC | MALE | 67 | T3a | NX | M0 | Stage III | G2 | LIVING | 22.5 | DiseaseFree | 22.5 |
| TCGA-B0-5095 | TCGA-B0-5095-01A-01R-1419-13 | KIRC | MALE | 81 | T3a | N0 | M0 | Stage III | G3 | DECEASED | 8.05 | Unknown | Unknown |
| TCGA-B0-4824 | TCGA-B0-4824-01A-01R-1276-13 | KIRC | FEMALE | 49 | T1a | N0 | M0 | Stage I | G3 | DECEASED | 54.43 | Unknown | Unknown |
| TCGA-T7-A92I | TCGA-T7-A92I-01A-11R-A37P-13 | KIRC | FEMALE | 47 | T1a | NX | MX | Stage I | G1 | LIVING | 11.7 | DiseaseFree | 11.7 |
| TCGA-CZ-5982 | TCGA-CZ-5982-01A-11R-1671-13 | KIRC | FEMALE | 59 | T1a | NX | M0 | Stage I | G2 | LIVING | 80.12 | DiseaseFree | 80.12 |
| TCGA-CZ-4859 | TCGA-CZ-4859-01A-02R-1425-13 | KIRC | FEMALE | 59 | T1 | N0 | M0 | Stage I | G2 | LIVING | 58.71 | DiseaseFree | 58.71 |
| TCGA-B8-5162 | TCGA-B8-5162-01A-01R-1419-13 | KIRC | MALE | 62 | T2a | NX | M0 | Stage II | G2 | LIVING | 1.18 | DiseaseFree | 1.18 |
| TCGA-B0-4843 | TCGA-B0-4843-01A-01R-1276-13 | KIRC | MALE | 57 | T3a | N0 | M0 | Stage III | G3 | DECEASED | 10.51 | Unknown | Unknown |
| TCGA-BP-4986 | TCGA-BP-4986-01A-01R-1762-13 | KIRC | MALE | 75 | T1a | N0 | M0 | Stage I | G3 | LIVING | 25.79 | DiseaseFree | 25.79 |
| TCGA-CJ-4640 | TCGA-CJ-4640-01A-01R-1187-13 | KIRC | MALE | 49 | T3a | N0 | M0 | Stage III | G4 | LIVING | 114.32 | DiseaseFree | 114.32 |
| TCGA-G6-A8L7 | TCGA-G6-A8L7-01A-11R-A37P-13 | KIRC | FEMALE | 81 | T1b | N0 | MX | Stage I | G3 | LIVING | 70.07 | DiseaseFree | 70.07 |
| TCGA-BP-4770 | TCGA-BP-4770-01A-01R-1502-13 | KIRC | FEMALE | 73 | T4 | N0 | M0 | Stage IV | G4 | DECEASED | 10.81 | Recurred/Progressed | 6.83 |
| TCGA-BP-4975 | TCGA-BP-4975-01A-01R-1762-13 | KIRC | MALE | 40 | T1b | NX | M0 | Stage I | G3 | LIVING | 47.08 | DiseaseFree | 47.08 |
| TCGA-B0-5709 | TCGA-B0-5709-01A-11R-1540-13 | KIRC | FEMALE | 62 | T3a | NX | M0 | Stage III | G3 | LIVING | 130.55 | DiseaseFree | 130.55 |
| TCGA-DV-5569 | TCGA-DV-5569-01A-01R-1540-13 | KIRC | FEMALE | 29 | T1a | NX | M0 | Stage I | G2 | LIVING | 11.66 | DiseaseFree | 11.66 |
| TCGA-BP-4334 | TCGA-BP-4334-01A-01R-1288-13 | KIRC | MALE | 56 | T3a | N0 | M0 | Stage III | G3 | DECEASED | 21.19 | Recurred/Progressed | 14.52 |
| TCGA-BP-4977 | TCGA-BP-4977-01A-01R-1333-13 | KIRC | MALE | 57 | T1b | NX | M0 | Stage I | G3 | LIVING | 14.91 | DiseaseFree | 14.91 |
| TCGA-G6-A8L8 | TCGA-G6-A8L8-01A-21R-A37P-13 | KIRC | FEMALE | 62 | T1b | NX | MX | Stage I | G3 | DECEASED | 35.84 | Unknown | Unknown |
| TCGA-CJ-4892 | TCGA-CJ-4892-01A-01R-1304-13 | KIRC | FEMALE | 65 | T1b | N0 | M0 | Stage I | G2 | LIVING | 49.97 | DiseaseFree | 49.97 |
| TCGA-B0-5711 | TCGA-B0-5711-01A-11R-1671-13 | KIRC | MALE | 50 | T3b | NX | M0 | Stage III | G3 | LIVING | 131.04 | Recurred/Progressed | 123.72 |
| TCGA-CZ-5453 | TCGA-CZ-5453-01A-01R-1502-13 | KIRC | MALE | 67 | T2 | NX | M0 | Stage II | G2 | DECEASED | 79.47 | Unknown | Unknown |
| TCGA-B0-4714 | TCGA-B0-4714-01A-01R-1276-13 | KIRC | MALE | 81 | T3b | NX | M1 | Stage IV | G3 | DECEASED | 3.25 | Unknown | Unknown |
| TCGA-BP-4345 | TCGA-BP-4345-01A-01R-1288-13 | KIRC | MALE | 62 | T3b | N0 | M0 | Stage III | G3 | LIVING | 49.8 | DiseaseFree | 49.8 |
| TCGA-B0-4839 | TCGA-B0-4839-01A-01R-1304-13 | KIRC | FEMALE | 80 | T1b | N0 | M0 | Stage I | G2 | DECEASED | 53.84 | Unknown | Unknown |
| TCGA-A3-3324 | TCGA-A3-3324-01A-02R-1324-13 | KIRC | MALE | 51 | T1b | NX | M0 | Stage I | G3 | LIVING | 38.96 | DiseaseFree | 38.96 |
| TCGA-BP-4161 | TCGA-BP-4161-01A-02R-1324-13 | KIRC | MALE | 74 | T1b | NX | M0 | Stage I | G3 | LIVING | 90.21 | Recurred/Progressed | 88.21 |
| TCGA-B0-5702 | TCGA-B0-5702-01A-11R-1540-13 | KIRC | MALE | 71 | T1b | N0 | M0 | Stage I | G2 | LIVING | 71.35 | DiseaseFree | 71.35 |
| TCGA-BP-4163 | TCGA-BP-4163-01A-02R-1324-13 | KIRC | FEMALE | 60 | T3a | N0 | M0 | Stage III | G3 | LIVING | 93.27 | DiseaseFree | 93.27 |
| TCGA-A3-A8CQ | TCGA-A3-A8CQ-01A-11R-A37P-13 | KIRC | FEMALE | 59 | T1a | NX | MX | Stage I | G2 | LIVING | 0.1 | DiseaseFree | 0.1 |
| TCGA-B0-4847 | TCGA-B0-4847-01A-01R-1276-13 | KIRC | MALE | 60 | T3a | NX | M1 | Stage IV | G3 | DECEASED | 26.05 | Unknown | Unknown |
| TCGA-B8-A54F | TCGA-B8-A54F-01A-11R-A25Z-13 | KIRC | FEMALE | 49 | T1a | NX | MX | Stage I | G2 | LIVING | 17.05 | DiseaseFree | 17.05 |
| TCGA-A3-3349 | TCGA-A3-3349-01A-01T-1187-13 | KIRC | FEMALE | 34 | T1b | N0 | M0 | Stage I | G2 | LIVING | 45.5 | DiseaseFree | 45.5 |
| TCGA-CZ-4856 | TCGA-CZ-4856-01A-02R-1425-13 | KIRC | FEMALE | 62 | T1b | N0 | M0 | Stage I | G2 | LIVING | 0.59 | DiseaseFree | 0.59 |
| TCGA-B8-A7U6 | TCGA-B8-A7U6-01A-12R-A37P-13 | KIRC | FEMALE | 54 | T1a | NX | Unknown | Stage I | G3 | LIVING | 16.26 | DiseaseFree | 16.26 |
| TCGA-CW-5590 | TCGA-CW-5590-01A-01R-1540-13 | KIRC | MALE | 51 | T3a | NX | M1 | Stage IV | G3 | DECEASED | 35.32 | Recurred/Progressed | 22.08 |
| TCGA-CJ-4893 | TCGA-CJ-4893-01A-01R-1304-13 | KIRC | FEMALE | 76 | T1b | NX | M0 | Stage I | G3 | LIVING | 24.64 | DiseaseFree | 24.64 |
| TCGA-A3-3378 | TCGA-A3-3378-01A-01T-0860-13 | KIRC | MALE | 60 | T1 | N0 | M0 | Stage I | G3 | LIVING | 20.7 | DiseaseFree | 20.7 |
| TCGA-B8-5546 | TCGA-B8-5546-01A-01R-1540-13 | KIRC | FEMALE | 38 | T1b | N0 | M0 | Stage I | G2 | LIVING | 16.59 | DiseaseFree | 16.59 |
| TCGA-BP-5001 | TCGA-BP-5001-01A-01R-1333-13 | KIRC | FEMALE | 43 | T1b | NX | M0 | Stage I | G2 | LIVING | 38.67 | DiseaseFree | 38.67 |
| TCGA-CJ-4890 | TCGA-CJ-4890-01A-01R-1304-13 | KIRC | MALE | 72 | T3a | N0 | M1 | Stage IV | G4 | LIVING | 115.6 | Recurred/Progressed | 48.52 |
| TCGA-B8-A54J | TCGA-B8-A54J-01A-11R-A33A-13 | KIRC | MALE | 60 | T2a | NX | MX | Stage II | G2 | LIVING | 17.35 | DiseaseFree | 17.35 |
| TCGA-BP-4160 | TCGA-BP-4160-01A-02R-1288-13 | KIRC | MALE | 67 | T3a | N0 | M0 | Stage III | G2 | LIVING | 94.65 | DiseaseFree | 94.65 |
| TCGA-BP-4355 | TCGA-BP-4355-01A-01R-1288-13 | KIRC | FEMALE | 59 | T3a | NX | M0 | Stage III | G4 | DECEASED | 31.31 | Unknown | Unknown |
| TCGA-BP-4176 | TCGA-BP-4176-01A-02R-1288-13 | KIRC | MALE | 64 | T1b | NX | M0 | Stage I | G2 | LIVING | 64.22 | DiseaseFree | 64.22 |
| TCGA-B0-4691 | TCGA-B0-4691-01A-01R-1276-13 | KIRC | MALE | 55 | T2 | N0 | M1 | Stage IV | G3 | DECEASED | 4.57 | Unknown | Unknown |
| TCGA-B0-5085 | TCGA-B0-5085-01A-01R-1762-13 | KIRC | FEMALE | 76 | T3a | N0 | M0 | Stage III | G3 | DECEASED | 25.3 | Unknown | Unknown |
| TCGA-CJ-4891 | TCGA-CJ-4891-01A-01R-1762-13 | KIRC | FEMALE | 57 | T3c | N0 | M0 | Stage III | G4 | DECEASED | 26.91 | Unknown | Unknown |
| TCGA-CJ-5671 | TCGA-CJ-5671-01A-11R-1540-13 | KIRC | MALE | 51 | T1a | NX | M0 | Stage I | G3 | LIVING | 130.98 | DiseaseFree | 130.98 |
| TCGA-BP-4758 | TCGA-BP-4758-01A-01R-1288-13 | KIRC | MALE | 40 | T1a | NX | M0 | Stage I | G2 | LIVING | 72.54 | DiseaseFree | 72.54 |
| TCGA-CJ-4923 | TCGA-CJ-4923-01A-01R-1425-13 | KIRC | FEMALE | 63 | T3a | NX | M1 | Stage IV | G4 | DECEASED | 18.79 | Recurred/Progressed | 0.82 |
| TCGA-DV-5565 | TCGA-DV-5565-01A-01R-1540-13 | KIRC | MALE | 59 | T1a | NX | M0 | Stage I | G2 | LIVING | 43.66 | DiseaseFree | 43.66 |
| TCGA-CJ-4907 | TCGA-CJ-4907-01A-01R-1425-13 | KIRC | MALE | 58 | T3b | NX | M0 | Stage III | G3 | LIVING | 49.24 | DiseaseFree | 49.24 |
| TCGA-B0-4811 | TCGA-B0-4811-01A-01R-1502-13 | KIRC | MALE | 48 | T3a | N0 | M0 | Stage III | G3 | DECEASED | 46.55 | Recurred/Progressed | 15.05 |
| TCGA-AK-3450 | TCGA-AK-3450-01A-02R-1276-13 | KIRC | FEMALE | 85 | T1a | N0 | M0 | Stage I | G2 | LIVING | 58.44 | DiseaseFree | 58.44 |
| TCGA-B0-5712 | TCGA-B0-5712-01A-11R-1671-13 | KIRC | FEMALE | 68 | T2 | N0 | M1 | Stage IV | G3 | LIVING | 89.42 | Recurred/Progressed | 23.82 |
| TCGA-B0-5399 | TCGA-B0-5399-01A-01R-1502-13 | KIRC | MALE | 46 | T1b | N0 | M0 | Stage I | G2 | LIVING | 46.35 | DiseaseFree | 46.35 |
| TCGA-B0-4693 | TCGA-B0-4693-01A-01R-1276-13 | KIRC | FEMALE | 72 | T3a | N0 | M0 | Stage III | G4 | DECEASED | 2.53 | Unknown | Unknown |
| TCGA-B0-4819 | TCGA-B0-4819-01A-01R-1276-13 | KIRC | FEMALE | 60 | T3b | NX | M1 | Stage IV | G4 | DECEASED | 6.01 | Unknown | Unknown |
| TCGA-DV-5567 | TCGA-DV-5567-01A-01R-1540-13 | KIRC | FEMALE | 40 | T1a | NX | M0 | Stage I | G2 | LIVING | 65.83 | Recurred/Progressed | 65.83 |
| TCGA-BP-5183 | TCGA-BP-5183-01A-01R-1425-13 | KIRC | MALE | 57 | T3a | NX | M0 | Stage III | G3 | LIVING | 42.41 | Recurred/Progressed | 15.93 |
| TCGA-A3-A6NI | TCGA-A3-A6NI-01A-11R-A33A-13 | KIRC | MALE | 47 | T1a | NX | MX | Stage I | G3 | LIVING | 33.44 | DiseaseFree | 33.44 |
| TCGA-B0-4842 | TCGA-B0-4842-01A-02R-1419-13 | KIRC | FEMALE | 73 | T3a | N0 | M0 | Stage III | G4 | DECEASED | 56.64 | Recurred/Progressed | 8.21 |
| TCGA-BP-4970 | TCGA-BP-4970-01A-01R-1762-13 | KIRC | MALE | 44 | T1a | N1 | M0 | Stage III | G3 | LIVING | 14.22 | DiseaseFree | 14.22 |
| TCGA-A3-3380 | TCGA-A3-3380-01A-01T-0860-13 | KIRC | MALE | 54 | T1 | N0 | M0 | Stage I | G2 | LIVING | 18.63 | DiseaseFree | 18.63 |
| TCGA-B0-4836 | TCGA-B0-4836-01A-01R-1762-13 | KIRC | MALE | 61 | T3b | NX | M1 | Stage IV | G3 | DECEASED | 40.67 | Unknown | Unknown |
| TCGA-B0-4849 | TCGA-B0-4849-01A-01R-1276-13 | KIRC | MALE | 51 | T3a | NX | M0 | Stage III | G3 | DECEASED | 2.27 | Recurred/Progressed | 0.13 |
| TCGA-B0-4810 | TCGA-B0-4810-01A-01R-1502-13 | KIRC | MALE | 47 | T3a | N1 | M0 | Stage III | G3 | DECEASED | 15.7 | Recurred/Progressed | 6.73 |
| TCGA-CJ-4889 | TCGA-CJ-4889-01A-01R-1304-13 | KIRC | FEMALE | 63 | T1a | NX | M0 | Stage I | G4 | LIVING | 63.93 | DiseaseFree | 63.93 |
| TCGA-B0-4688 | TCGA-B0-4688-01A-01R-1276-13 | KIRC | MALE | 46 | T4 | N0 | M1 | Stage IV | G4 | DECEASED | 3.32 | Unknown | Unknown |
| TCGA-CJ-5681 | TCGA-CJ-5681-01A-11R-1540-13 | KIRC | FEMALE | 44 | T3a | NX | M1 | Stage IV | G3 | DECEASED | 18.13 | Recurred/Progressed | 0.56 |
| TCGA-A3-3374 | TCGA-A3-3374-01A-01T-0860-13 | KIRC | FEMALE | 51 | T1b | N0 | M0 | Stage I | G2 | LIVING | 43.17 | DiseaseFree | 43.17 |
| TCGA-CJ-4637 | TCGA-CJ-4637-01A-01R-1187-13 | KIRC | FEMALE | 52 | T2b | NX | M1 | Stage IV | G4 | DECEASED | 73.16 | Recurred/Progressed | 59.82 |
| TCGA-CJ-4636 | TCGA-CJ-4636-01A-01R-1187-13 | KIRC | MALE | 51 | T3a | N0 | M0 | Stage III | G3 | LIVING | 63.21 | DiseaseFree | 63.21 |
| TCGA-B2-5636 | TCGA-B2-5636-01A-02R-1540-13 | KIRC | MALE | 79 | T1a | NX | M0 | Stage I | G2 | LIVING | 30.19 | DiseaseFree | 30.19 |
| TCGA-B8-A8YJ | TCGA-B8-A8YJ-01A-13R-A39B-13 | KIRC | FEMALE | 60 | T1b | NX | Unknown | Stage I | G2 | LIVING | 14.16 | DiseaseFree | 14.16 |
| TCGA-A3-3387 | TCGA-A3-3387-01A-01R-1540-13 | KIRC | MALE | 49 | T1a | N0 | M0 | Stage I | G2 | LIVING | 20.27 | DiseaseFree | 20.27 |
| TCGA-BP-5175 | TCGA-BP-5175-01A-01R-1425-13 | KIRC | MALE | 60 | T1a | NX | M0 | Stage I | G3 | LIVING | 30.62 | DiseaseFree | 30.62 |
| TCGA-AK-3429 | TCGA-AK-3429-01A-02R-1324-13 | KIRC | FEMALE | 54 | T2 | N0 | M0 | Stage II | G2 | LIVING | 109.33 | DiseaseFree | 109.33 |
| TCGA-BP-5180 | TCGA-BP-5180-01A-01R-1425-13 | KIRC | MALE | 53 | T1a | NX | M0 | Stage I | G2 | LIVING | 74.34 | DiseaseFree | 74.34 |
| TCGA-CJ-4887 | TCGA-CJ-4887-01A-01R-1762-13 | KIRC | MALE | 48 | T3a | NX | M1 | Stage IV | G3 | DECEASED | 30.62 | Recurred/Progressed | 10.58 |
| TCGA-BP-4981 | TCGA-BP-4981-01A-01R-1762-13 | KIRC | FEMALE | 75 | T3a | NX | M0 | Stage III | G3 | DECEASED | 36.04 | Unknown | Unknown |
| TCGA-BP-4763 | TCGA-BP-4763-01A-01R-1288-13 | KIRC | FEMALE | 79 | T1a | NX | M0 | Stage I | G2 | DECEASED | 41.72 | Unknown | Unknown |
| TCGA-BP-5008 | TCGA-BP-5008-01A-01R-1333-13 | KIRC | MALE | 46 | T1a | NX | M0 | Stage I | G2 | LIVING | 35.18 | DiseaseFree | 35.18 |
| TCGA-CJ-4876 | TCGA-CJ-4876-01A-01R-1304-13 | KIRC | MALE | 57 | T2b | N0 | M0 | Stage II | G3 | LIVING | 64.22 | DiseaseFree | 64.22 |
| TCGA-CW-6088 | TCGA-CW-6088-01A-11R-1671-13 | KIRC | MALE | 60 | T1b | N0 | M0 | Stage I | G2 | LIVING | 105.85 | DiseaseFree | 105.85 |
| TCGA-CW-5591 | TCGA-CW-5591-01A-01R-1540-13 | KIRC | MALE | 56 | T3a | N0 | M1 | Stage IV | G2 | LIVING | 74.61 | DiseaseFree | 74.61 |
| TCGA-CJ-4903 | TCGA-CJ-4903-01A-01R-1425-13 | KIRC | MALE | 50 | T1b | NX | M0 | Stage I | G3 | LIVING | 51.25 | DiseaseFree | 51.25 |
| TCGA-CW-5587 | TCGA-CW-5587-01A-01R-1540-13 | KIRC | FEMALE | 62 | T3b | N0 | M0 | Stage III | G2 | LIVING | 73.13 | Recurred/Progressed | 65.8 |
| TCGA-CJ-4881 | TCGA-CJ-4881-01A-01R-1762-13 | KIRC | MALE | 41 | T3a | NX | M0 | Stage III | G3 | LIVING | 66.16 | Recurred/Progressed | 61.79 |
| TCGA-B0-5117 | TCGA-B0-5117-01A-01R-1419-13 | KIRC | MALE | 40 | T1b | NX | M0 | Stage I | G2 | LIVING | 52.83 | DiseaseFree | 52.83 |
| TCGA-CJ-5689 | TCGA-CJ-5689-01A-11R-1540-13 | KIRC | MALE | 90 | T1b | NX | M0 | Stage I | G4 | DECEASED | 53.22 | Unknown | Unknown |
| TCGA-CW-5585 | TCGA-CW-5585-01A-01R-1540-13 | KIRC | MALE | 51 | T3b | N0 | M1 | Stage IV | G2 | LIVING | 85.71 | DiseaseFree | 85.71 |
| TCGA-B4-5843 | TCGA-B4-5843-01A-11R-1671-13 | KIRC | MALE | 45 | T1 | N0 | M0 | Stage I | G2 | LIVING | 0.36 | DiseaseFree | 0.36 |
| TCGA-B0-4710 | TCGA-B0-4710-01A-01R-1502-13 | KIRC | FEMALE | 75 | T3a | N0 | M0 | Stage III | G3 | LIVING | 57.65 | DiseaseFree | 57.65 |
| TCGA-A3-3311 | TCGA-A3-3311-01A-01T-0860-13 | KIRC | MALE | 57 | T1 | NX | M0 | Stage I | G2 | DECEASED | 39.13 | Unknown | Unknown |
| TCGA-EU-5904 | TCGA-EU-5904-01A-11R-1671-13 | KIRC | FEMALE | 47 | T1 | NX | M0 | Stage I | G1 | LIVING | 18.1 | DiseaseFree | 18.1 |
| TCGA-CJ-4920 | TCGA-CJ-4920-01A-01R-1425-13 | KIRC | FEMALE | 64 | T1b | NX | M0 | Stage I | G2 | DECEASED | 4.57 | Unknown | Unknown |
| TCGA-BP-4174 | TCGA-BP-4174-01A-02R-1288-13 | KIRC | MALE | 49 | T2 | N0 | M0 | Stage II | G3 | LIVING | 61.73 | DiseaseFree | 61.73 |
| TCGA-AK-3444 | TCGA-AK-3444-01A-01T-0860-13 | KIRC | FEMALE | 80 | T1b | NX | M0 | Stage I | G2 | LIVING | 48.32 | DiseaseFree | 48.32 |
| TCGA-A3-3317 | TCGA-A3-3317-01A-02R-1324-13 | KIRC | MALE | 67 | T2 | N0 | M0 | Stage II | G2 | LIVING | 48.98 | Recurred/Progressed | 31.27 |
| TCGA-B0-5106 | TCGA-B0-5106-01A-01R-1419-13 | KIRC | MALE | 64 | T1a | N0 | M0 | Stage I | G2 | DECEASED | 52.5 | Unknown | Unknown |
| TCGA-CJ-5682 | TCGA-CJ-5682-01A-11R-1540-13 | KIRC | MALE | 60 | T3a | NX | M1 | Stage IV | G4 | LIVING | 122.73 | Recurred/Progressed | 1.81 |
| TCGA-CZ-5467 | TCGA-CZ-5467-01A-01R-1502-13 | KIRC | FEMALE | 86 | T3a | N0 | M0 | Stage III | G4 | DECEASED | 2.4 | Recurred/Progressed | 2.04 |
| TCGA-B2-3924 | TCGA-B2-3924-01A-02R-A27D-13 | KIRC | MALE | 73 | T1b | NX | M0 | Stage I | G2 | LIVING | 35.87 | DiseaseFree | 35.87 |
| TCGA-AK-3440 | TCGA-AK-3440-01A-01T-0860-13 | KIRC | MALE | 58 | T1a | NX | M0 | Stage I | G3 | LIVING | 94.12 | DiseaseFree | 94.12 |
| TCGA-MW-A4EC | TCGA-MW-A4EC-01A-11R-A25Z-13 | KIRC | FEMALE | 72 | T1a | NX | MX | Stage I | G2 | LIVING | 16.36 | DiseaseFree | 16.36 |
| TCGA-A3-3378 | TCGA-A3-3378-01A-02R-1324-13 | KIRC | MALE | 60 | T1 | N0 | M0 | Stage I | G3 | LIVING | 20.7 | DiseaseFree | 20.7 |
| TCGA-BP-5201 | TCGA-BP-5201-01A-01R-1425-13 | KIRC | MALE | 63 | T3b | N0 | M1 | Stage IV | G4 | LIVING | 31.24 | Recurred/Progressed | -0.62 |
| TCGA-CZ-4860 | TCGA-CZ-4860-01A-01R-1304-13 | KIRC | MALE | 60 | T4 | NX | M1 | Stage IV | G4 | DECEASED | 6.77 | Unknown | Unknown |
| TCGA-BP-4798 | TCGA-BP-4798-01A-01R-1304-13 | KIRC | MALE | 74 | T3b | N0 | M1 | Stage IV | G4 | DECEASED | 10.97 | Unknown | Unknown |
| TCGA-A3-3367 | TCGA-A3-3367-01A-02R-1419-13 | KIRC | MALE | 72 | T1b | N0 | M0 | Stage I | G3 | LIVING | 74.57 | DiseaseFree | 74.57 |
| TCGA-BP-5169 | TCGA-BP-5169-01A-01R-1425-13 | KIRC | MALE | 70 | T1b | N0 | M0 | Stage I | G4 | LIVING | 6.34 | DiseaseFree | 6.34 |
| TCGA-CW-5581 | TCGA-CW-5581-01A-02R-1540-13 | KIRC | MALE | 44 | T1b | NX | M0 | Stage I | G3 | LIVING | 91.95 | DiseaseFree | 91.95 |
| TCGA-CJ-6032 | TCGA-CJ-6032-01A-11R-1671-13 | KIRC | FEMALE | 63 | T2 | NX | M0 | Stage II | G3 | LIVING | 119.55 | DiseaseFree | 119.55 |
| TCGA-B2-3923 | TCGA-B2-3923-01A-02R-1324-13 | KIRC | MALE | 59 | T2 | NX | M0 | Stage II | G2 | LIVING | 32.59 | DiseaseFree | 32.59 |
| TCGA-BP-5196 | TCGA-BP-5196-01A-01R-1425-13 | KIRC | MALE | 53 | T1a | NX | M0 | Stage I | G2 | LIVING | 33.44 | DiseaseFree | 33.44 |
| TCGA-CJ-6033 | TCGA-CJ-6033-01A-11R-1671-13 | KIRC | FEMALE | 54 | T3a | N0 | M1 | Stage IV | G4 | DECEASED | 7.36 | Recurred/Progressed | 4.73 |
| TCGA-B0-5699 | TCGA-B0-5699-01A-11R-1540-13 | KIRC | MALE | 53 | T1 | N0 | M0 | Stage I | G2 | LIVING | 126.18 | Recurred/Progressed | 3.09 |
| TCGA-B4-5377 | TCGA-B4-5377-01A-01R-1502-13 | KIRC | FEMALE | 68 | T3 | N0 | M1 | Stage IV | G3 | LIVING | 11.99 | DiseaseFree | 11.99 |
| TCGA-B0-5083 | TCGA-B0-5083-01A-02R-1419-13 | KIRC | MALE | 63 | T1a | N0 | M0 | Stage I | G3 | DECEASED | 34.33 | Unknown | Unknown |
| TCGA-CJ-4872 | TCGA-CJ-4872-01A-01R-1304-13 | KIRC | MALE | 51 | T1b | N0 | M0 | Stage I | G4 | LIVING | 47.14 | DiseaseFree | 47.14 |
| TCGA-B2-4098 | TCGA-B2-4098-01A-02R-1324-13 | KIRC | FEMALE | 72 | T1b | NX | M0 | Stage I | G2 | DECEASED | 1.68 | Unknown | Unknown |
| TCGA-BP-5189 | TCGA-BP-5189-01A-02R-1425-13 | KIRC | MALE | 60 | T1b | NX | M0 | Stage I | G4 | DECEASED | 27 | Recurred/Progressed | 25.66 |
| TCGA-BP-4327 | TCGA-BP-4327-01A-01R-1288-13 | KIRC | FEMALE | 75 | T2 | N0 | M0 | Stage II | G2 | DECEASED | 3.58 | Unknown | Unknown |
| TCGA-B0-5703 | TCGA-B0-5703-01A-11R-1540-13 | KIRC | MALE | 73 | T1b | N0 | M0 | Stage I | G3 | LIVING | 73.78 | DiseaseFree | 73.78 |
| TCGA-A3-3324 | TCGA-A3-3324-01A-01T-0860-13 | KIRC | MALE | 51 | T1b | NX | M0 | Stage I | G3 | LIVING | 38.96 | DiseaseFree | 38.96 |
| TCGA-B0-4833 | TCGA-B0-4833-01A-01R-1304-13 | KIRC | FEMALE | 82 | T1b | N0 | M0 | Stage I | G2 | DECEASED | 78.38 | Unknown | Unknown |
| TCGA-B0-5104 | TCGA-B0-5104-01A-01R-1419-13 | KIRC | FEMALE | 90 | T1 | N0 | M0 | Stage I | G2 | DECEASED | 90.41 | Unknown | Unknown |
| TCGA-DV-A4VX | TCGA-DV-A4VX-01A-11R-A25Z-13 | KIRC | MALE | 59 | T3b | N0 | MX | Stage IV | G4 | DECEASED | 53.42 | Unknown | Unknown |
| TCGA-BP-4993 | TCGA-BP-4993-01A-02R-1419-13 | KIRC | MALE | 58 | T1a | NX | M0 | Stage I | G3 | LIVING | 5.81 | DiseaseFree | 5.81 |
| TCGA-AK-3451 | TCGA-AK-3451-01A-02T-1187-13 | KIRC | MALE | 48 | T2 | N0 | M0 | Stage II | G3 | LIVING | 94.22 | DiseaseFree | 94.22 |
| TCGA-A3-3347 | TCGA-A3-3347-01A-02R-1324-13 | KIRC | FEMALE | 76 | T1b | N1 | M0 | Stage III | G2 | DECEASED | 52.89 | Recurred/Progressed | 20.04 |
| TCGA-CW-5580 | TCGA-CW-5580-01A-01R-1671-13 | KIRC | FEMALE | 73 | T3a | NX | M1 | Stage IV | G3 | DECEASED | 64.52 | Unknown | Unknown |
| TCGA-CJ-4871 | TCGA-CJ-4871-01A-01R-1304-13 | KIRC | MALE | 63 | T3a | NX | M1 | Stage IV | G4 | LIVING | 79.6 | Recurred/Progressed | 5.55 |
| TCGA-AK-3436 | TCGA-AK-3436-01A-02R-1324-13 | KIRC | MALE | 40 | T2 | N0 | M1 | Stage IV | G2 | LIVING | 109.43 | Recurred/Progressed | 39.06 |
| TCGA-A3-A6NJ | TCGA-A3-A6NJ-01A-12R-A33A-13 | KIRC | FEMALE | 57 | T1a | NX | MX | Stage I | G1 | LIVING | 15.37 | DiseaseFree | 15.37 |
| TCGA-BP-4162 | TCGA-BP-4162-01A-02R-1324-13 | KIRC | FEMALE | 65 | T1b | N0 | M0 | Stage I | G2 | LIVING | 100.99 | DiseaseFree | 100.99 |
| TCGA-CZ-5989 | TCGA-CZ-5989-01A-11R-1671-13 | KIRC | MALE | 60 | T2 | N0 | M0 | Stage II | G2 | LIVING | 62.58 | DiseaseFree | 62.58 |
| TCGA-BP-4351 | TCGA-BP-4351-01A-01R-1288-13 | KIRC | FEMALE | 51 | T3a | N0 | M0 | Stage III | G2 | LIVING | 31.87 | Recurred/Progressed | 28.68 |
| TCGA-B2-5633 | TCGA-B2-5633-01A-01R-A27D-13 | KIRC | MALE | 56 | T1b | N0 | M0 | Stage I | G2 | LIVING | 31.64 | DiseaseFree | 31.64 |
| TCGA-B0-5108 | TCGA-B0-5108-01A-01R-1419-13 | KIRC | MALE | 54 | T3a | N0 | M0 | Stage III | G2 | LIVING | 58.54 | DiseaseFree | 58.54 |
| TCGA-BP-4776 | TCGA-BP-4776-01A-01R-1288-13 | KIRC | MALE | 52 | T1a | NX | M0 | Stage I | G2 | LIVING | 13.5 | DiseaseFree | 13.5 |
| TCGA-CJ-5686 | TCGA-CJ-5686-01A-11R-1671-13 | KIRC | FEMALE | 59 | T1b | NX | M0 | Stage I | G3 | LIVING | 66.95 | DiseaseFree | 66.95 |
| TCGA-BP-4974 | TCGA-BP-4974-01A-01R-1333-13 | KIRC | MALE | 58 | T3a | N0 | M1 | Stage IV | G4 | DECEASED | 6.93 | Recurred/Progressed | 1.15 |
| TCGA-B8-5550 | TCGA-B8-5550-01A-01R-1540-13 | KIRC | MALE | 71 | T3a | N0 | M0 | Stage III | G3 | LIVING | 48.49 | Recurred/Progressed | 22.8 |
| TCGA-BP-4787 | TCGA-BP-4787-01A-01R-1304-13 | KIRC | FEMALE | 59 | T3a | N0 | M1 | Stage IV | G4 | DECEASED | 15.77 | Recurred/Progressed | 14.49 |
| TCGA-BP-4164 | TCGA-BP-4164-01A-02R-1324-13 | KIRC | FEMALE | 51 | T3a | NX | M0 | Stage III | G2 | DECEASED | 32.59 | Unknown | Unknown |
| TCGA-B2-3923 | TCGA-B2-3923-01B-10R-A27D-13 | KIRC | MALE | 59 | T2 | NX | M0 | Stage II | G2 | LIVING | 32.59 | DiseaseFree | 32.59 |
| TCGA-B0-4694 | TCGA-B0-4694-01A-01R-1276-13 | KIRC | MALE | 72 | T3b | NX | M0 | Stage III | G4 | DECEASED | 3.48 | Unknown | Unknown |
| TCGA-B0-5690 | TCGA-B0-5690-01A-11R-1540-13 | KIRC | FEMALE | 53 | T1b | NX | M0 | Stage I | G1 | LIVING | 111.43 | DiseaseFree | 111.43 |
| TCGA-CJ-4875 | TCGA-CJ-4875-01A-01R-1762-13 | KIRC | MALE | 67 | T3a | NX | M1 | Stage IV | G3 | DECEASED | 116.75 | Recurred/Progressed | 17.44 |
| TCGA-CJ-4888 | TCGA-CJ-4888-01A-01R-1762-13 | KIRC | MALE | 59 | T3a | NX | M1 | Stage IV | G4 | DECEASED | 51.48 | Recurred/Progressed | 19.97 |
| TCGA-B0-4845 | TCGA-B0-4845-01A-01R-1276-13 | KIRC | MALE | 70 | T3a | NX | M1 | Stage IV | G2 | DECEASED | 65.24 | Recurred/Progressed | 24.57 |
| TCGA-CJ-4634 | TCGA-CJ-4634-01A-01R-1187-13 | KIRC | FEMALE | 60 | T1b | NX | M0 | Stage I | G2 | LIVING | 114.91 | DiseaseFree | 114.91 |
| TCGA-BP-4354 | TCGA-BP-4354-01A-02R-1288-13 | KIRC | MALE | 40 | T4 | N1 | M1 | Stage IV | G4 | DECEASED | 33.97 | Recurred/Progressed | 22.5 |
| TCGA-B0-4718 | TCGA-B0-4718-01A-01R-1276-13 | KIRC | MALE | 57 | T3a | NX | M0 | Stage III | G2 | LIVING | 58.41 | Recurred/Progressed | 25.23 |
| TCGA-BP-4166 | TCGA-BP-4166-01A-02R-1288-13 | KIRC | MALE | 69 | T3a | N0 | M0 | Stage III | G3 | LIVING | 0.43 | DiseaseFree | 0.43 |
| TCGA-B0-5121 | TCGA-B0-5121-01A-02R-1419-13 | KIRC | MALE | 56 | T1b | N0 | M0 | Stage I | G2 | LIVING | 48.78 | DiseaseFree | 48.78 |
| TCGA-BP-4761 | TCGA-BP-4761-01A-01R-1762-13 | KIRC | MALE | 57 | T3a | N1 | M0 | Stage III | G4 | LIVING | 5.98 | DiseaseFree | 5.98 |
| TCGA-CZ-4861 | TCGA-CZ-4861-01A-01R-1304-13 | KIRC | MALE | 63 | T2 | NX | M0 | Stage II | G2 | DECEASED | 14.65 | Unknown | Unknown |
| TCGA-A3-3372 | TCGA-A3-3372-01A-01T-0860-13 | KIRC | MALE | 64 | T3 | NX | M0 | Stage III | G2 | LIVING | 24.15 | DiseaseFree | 24.15 |
| TCGA-B2-A4SR | TCGA-B2-A4SR-01A-11R-A25Z-13 | KIRC | MALE | 61 | T2a | NX | M0 | Stage II | Unknown | LIVING | 16.66 | Recurred/Progressed | 8.74 |
| TCGA-AK-3455 | TCGA-AK-3455-01A-01T-0860-13 | KIRC | FEMALE | 71 | T3b | NX | M0 | Stage III | G3 | DECEASED | 22.44 | Unknown | Unknown |
| TCGA-CZ-4863 | TCGA-CZ-4863-01A-01R-1502-13 | KIRC | FEMALE | 51 | T3b | N0 | M0 | Stage III | G3 | LIVING | 63.34 | DiseaseFree | 63.34 |
| TCGA-B8-5158 | TCGA-B8-5158-01A-01R-1419-13 | KIRC | MALE | 56 | T3a | N1 | M0 | Stage III | G4 | LIVING | 40.01 | DiseaseFree | 40.01 |
| TCGA-BP-4799 | TCGA-BP-4799-01A-01R-1304-13 | KIRC | MALE | 70 | T3b | N0 | M0 | Stage III | G3 | DECEASED | 37.22 | Recurred/Progressed | 11.56 |
| TCGA-B4-5834 | TCGA-B4-5834-01A-11R-1671-13 | KIRC | MALE | 59 | T1 | N0 | M0 | Stage I | G1 | LIVING | 1.25 | DiseaseFree | 1.25 |
| TCGA-B2-5639 | TCGA-B2-5639-01A-01R-1540-13 | KIRC | MALE | 46 | T3 | NX | M1 | Stage IV | G3 | DECEASED | 32.95 | Recurred/Progressed | 27.96 |
| TCGA-B0-4707 | TCGA-B0-4707-01A-01R-1276-13 | KIRC | MALE | 63 | T3a | NX | M0 | Stage III | G4 | DECEASED | 19.71 | Unknown | Unknown |
| TCGA-BP-5185 | TCGA-BP-5185-01A-01R-1425-13 | KIRC | MALE | 56 | T1a | NX | M0 | Stage I | G3 | LIVING | 37.19 | Recurred/Progressed | 24.18 |
| TCGA-BP-4331 | TCGA-BP-4331-01A-01R-1762-13 | KIRC | MALE | 52 | T1a | N0 | M0 | Stage I | G2 | DECEASED | 80.62 | Unknown | Unknown |
| TCGA-CJ-4642 | TCGA-CJ-4642-01B-02R-1409-13 | KIRC | MALE | 47 | T2 | NX | M0 | Stage II | G2 | LIVING | 105.29 | DiseaseFree | 105.29 |
| TCGA-B0-5698 | TCGA-B0-5698-01A-11R-1671-13 | KIRC | MALE | 77 | T1b | N0 | M0 | Stage I | G3 | LIVING | 119.28 | DiseaseFree | 119.28 |
| TCGA-B0-5097 | TCGA-B0-5097-01A-01R-1419-13 | KIRC | FEMALE | 59 | T3b | N0 | M0 | Stage III | G2 | LIVING | 21.85 | Recurred/Progressed | 11.79 |
| TCGA-B8-5552 | TCGA-B8-5552-01B-11R-1671-13 | KIRC | FEMALE | 41 | T1b | NX | M0 | Stage I | G2 | LIVING | 34.36 | DiseaseFree | 34.36 |
| TCGA-CJ-4882 | TCGA-CJ-4882-01A-02R-1425-13 | KIRC | MALE | 57 | T3a | NX | M0 | Stage III | G3 | LIVING | 61.86 | DiseaseFree | 61.86 |
| TCGA-B0-4945 | TCGA-B0-4945-01A-01R-1419-13 | KIRC | FEMALE | 75 | T1a | N0 | M0 | Stage I | G2 | DECEASED | 70.47 | Unknown | Unknown |
| TCGA-B0-4837 | TCGA-B0-4837-01A-01R-1304-13 | KIRC | MALE | 63 | T1b | N0 | M0 | Stage I | G3 | DECEASED | 45.27 | Recurred/Progressed | 39.68 |
| TCGA-AK-3447 | TCGA-AK-3447-01A-01T-0860-13 | KIRC | MALE | 83 | T2 | NX | M0 | Stage II | G2 | LIVING | 39.98 | DiseaseFree | 39.98 |
| TCGA-B8-5553 | TCGA-B8-5553-01A-01R-1540-13 | KIRC | FEMALE | 67 | T1b | N0 | M0 | Stage I | G2 | LIVING | 14.29 | DiseaseFree | 14.29 |
| TCGA-B8-4151 | TCGA-B8-4151-01A-01T-1187-13 | KIRC | FEMALE | 51 | T3a | N0 | M0 | Stage III | G2 | LIVING | 42.67 | DiseaseFree | 42.67 |
| TCGA-CZ-4858 | TCGA-CZ-4858-01A-01R-1304-13 | KIRC | MALE | 39 | T2 | NX | M0 | Stage II | G4 | DECEASED | 69.15 | Recurred/Progressed | 62.81 |
| TCGA-B2-3924 | TCGA-B2-3924-01B-03R-A27D-13 | KIRC | MALE | 73 | T1b | NX | M0 | Stage I | G2 | LIVING | 35.87 | DiseaseFree | 35.87 |
| TCGA-B8-4622 | TCGA-B8-4622-01A-02R-1276-13 | KIRC | MALE | 57 | T3a | N0 | M1 | Stage IV | G3 | LIVING | 50.1 | Recurred/Progressed | 40.31 |
| TCGA-BP-5176 | TCGA-BP-5176-01A-01R-1425-13 | KIRC | FEMALE | 78 | T1a | NX | M0 | Stage I | G2 | DECEASED | 52.23 | Unknown | Unknown |
| TCGA-CW-5584 | TCGA-CW-5584-01A-01R-1540-13 | KIRC | MALE | 74 | T3b | N1 | M0 | Stage III | G3 | DECEASED | 5.39 | Recurred/Progressed | 3.35 |
| TCGA-A3-3323 | TCGA-A3-3323-01A-01T-0860-13 | KIRC | MALE | 53 | T1b | NX | M0 | Stage I | G1 | LIVING | 36.33 | DiseaseFree | 36.33 |
| TCGA-CZ-4853 | TCGA-CZ-4853-01A-01R-1425-13 | KIRC | MALE | 82 | T1a | NX | M0 | Stage I | G2 | LIVING | 25.43 | DiseaseFree | 25.43 |
| TCGA-CZ-4854 | TCGA-CZ-4854-01A-01R-1762-13 | KIRC | MALE | 68 | T1b | N0 | M0 | Stage I | G2 | DECEASED | 46.12 | Unknown | Unknown |
| TCGA-BP-4985 | TCGA-BP-4985-01A-01R-1333-13 | KIRC | MALE | 72 | T3a | N0 | M0 | Stage III | G4 | DECEASED | 31.27 | Recurred/Progressed | 21.58 |
| TCGA-CJ-4918 | TCGA-CJ-4918-01A-01R-1425-13 | KIRC | MALE | 64 | T3a | N0 | M1 | Stage IV | G4 | DECEASED | 3.06 | Recurred/Progressed | 1.08 |
| TCGA-B0-4818 | TCGA-B0-4818-01A-01R-1502-13 | KIRC | FEMALE | 68 | T2 | NX | M0 | Stage II | G3 | DECEASED | 16.75 | Recurred/Progressed | 4.17 |
| TCGA-B2-3924 | TCGA-B2-3924-01A-02R-1324-13 | KIRC | MALE | 73 | T1b | NX | M0 | Stage I | G2 | LIVING | 35.87 | DiseaseFree | 35.87 |
| TCGA-BP-4959 | TCGA-BP-4959-01A-01R-1333-13 | KIRC | MALE | 49 | T1b | NX | M0 | Stage I | G3 | LIVING | 87.39 | DiseaseFree | 87.39 |
| TCGA-B0-4848 | TCGA-B0-4848-01A-01R-1276-13 | KIRC | MALE | 54 | T3b | NX | M0 | Stage III | G3 | DECEASED | 29.01 | Recurred/Progressed | 16.23 |
| TCGA-B0-4813 | TCGA-B0-4813-01A-01R-1276-13 | KIRC | MALE | 68 | T3b | NX | M0 | Stage III | G3 | DECEASED | 0.59 | Unknown | Unknown |
| TCGA-BP-4756 | TCGA-BP-4756-01A-01R-1288-13 | KIRC | FEMALE | 62 | T1b | N0 | M0 | Stage I | G2 | LIVING | 12.29 | DiseaseFree | 12.29 |
| TCGA-B0-4700 | TCGA-B0-4700-01A-02R-1540-13 | KIRC | MALE | 60 | T4 | NX | M1 | Stage IV | G4 | DECEASED | 65.05 | Unknown | Unknown |
| TCGA-BP-4969 | TCGA-BP-4969-01A-01R-1762-13 | KIRC | FEMALE | 63 | T1a | NX | M0 | Stage I | G2 | LIVING | 58.94 | DiseaseFree | 58.94 |
| TCGA-BP-4173 | TCGA-BP-4173-01A-02R-1762-13 | KIRC | MALE | 47 | T2 | N0 | M0 | Stage II | G3 | LIVING | 62.19 | DiseaseFree | 62.19 |
| TCGA-B0-5402 | TCGA-B0-5402-01A-01R-1502-13 | KIRC | MALE | 64 | T4 | NX | M0 | Stage IV | G4 | LIVING | 42.38 | Recurred/Progressed | 14.75 |
| TCGA-BP-4797 | TCGA-BP-4797-01A-01R-1762-13 | KIRC | MALE | 34 | T3b | N0 | M0 | Stage III | G3 | LIVING | 36.37 | DiseaseFree | 36.37 |
| TCGA-AK-3433 | TCGA-AK-3433-01A-02R-1276-13 | KIRC | FEMALE | 48 | T2 | N0 | M0 | Stage II | GX | LIVING | 111.99 | DiseaseFree | 111.99 |
| TCGA-BP-5174 | TCGA-BP-5174-01A-01R-1425-13 | KIRC | FEMALE | 45 | T1a | NX | M0 | Stage I | G2 | LIVING | 74.15 | DiseaseFree | 74.15 |
| TCGA-B0-5099 | TCGA-B0-5099-01A-01R-1419-13 | KIRC | FEMALE | 88 | T3b | NX | M0 | Stage III | G3 | DECEASED | 15.93 | Unknown | Unknown |
| TCGA-AK-3430 | TCGA-AK-3430-01A-01T-1187-13 | KIRC | MALE | 61 | T3b | N1 | M0 | Stage III | G3 | DECEASED | 15.77 | Unknown | Unknown |
| TCGA-B0-5812 | TCGA-B0-5812-01A-11R-1671-13 | KIRC | MALE | 53 | T1b | NX | M0 | Stage I | G3 | LIVING | 125.95 | DiseaseFree | 125.95 |
| TCGA-B0-5109 | TCGA-B0-5109-01A-02R-1419-13 | KIRC | MALE | 69 | T3b | N1 | M0 | Stage III | G4 | DECEASED | 19.28 | Recurred/Progressed | 2.3 |
| TCGA-BP-4781 | TCGA-BP-4781-01A-01R-1304-13 | KIRC | MALE | 78 | T1a | NX | M0 | Stage I | G3 | LIVING | 68.33 | DiseaseFree | 68.33 |
| TCGA-B0-4713 | TCGA-B0-4713-01A-01R-1276-13 | KIRC | FEMALE | 76 | T3b | NX | M0 | Stage III | G2 | DECEASED | 6.64 | Unknown | Unknown |
| TCGA-BP-4326 | TCGA-BP-4326-01A-01R-1288-13 | KIRC | FEMALE | 53 | T1b | N0 | M0 | Stage I | G2 | DECEASED | 53.38 | Recurred/Progressed | 31.47 |
| TCGA-BP-5194 | TCGA-BP-5194-01A-02R-1425-13 | KIRC | MALE | 39 | T1a | NX | M0 | Stage I | G2 | LIVING | 13.4 | DiseaseFree | 13.4 |
| TCGA-B0-5088 | TCGA-B0-5088-01A-01R-1333-13 | KIRC | MALE | 53 | T1b | N0 | M0 | Stage I | G3 | DECEASED | 18.5 | Unknown | Unknown |
| TCGA-BP-4973 | TCGA-BP-4973-01A-01R-1333-13 | KIRC | MALE | 47 | T3a | NX | M0 | Stage III | G3 | LIVING | 45.47 | DiseaseFree | 45.47 |
| TCGA-CW-6093 | TCGA-CW-6093-01A-11R-1671-13 | KIRC | MALE | 73 | T1a | NX | M0 | Stage I | G1 | LIVING | 103.35 | DiseaseFree | 103.35 |
| TCGA-A3-3383 | TCGA-A3-3383-01A-02R-1324-13 | KIRC | MALE | 52 | T1 | NX | M0 | Stage I | G2 | LIVING | 28.29 | DiseaseFree | 28.29 |
| TCGA-BP-4352 | TCGA-BP-4352-01A-01R-1288-13 | KIRC | FEMALE | 74 | T3b | N0 | M1 | Stage IV | G4 | DECEASED | 11.3 | Recurred/Progressed | 8.71 |
| TCGA-CZ-4864 | TCGA-CZ-4864-01A-01R-1502-13 | KIRC | MALE | 86 | T2 | N0 | M0 | Stage II | G3 | DECEASED | 92.97 | Unknown | Unknown |
| TCGA-CZ-5987 | TCGA-CZ-5987-01A-11R-1671-13 | KIRC | MALE | 60 | T3b | NX | M1 | Stage IV | G2 | DECEASED | 14.62 | Recurred/Progressed | 12.39 |
| TCGA-AK-3445 | TCGA-AK-3445-01A-02R-1276-13 | KIRC | MALE | 69 | T3a | NX | M0 | Stage III | G3 | LIVING | 78.58 | DiseaseFree | 78.58 |
| TCGA-B0-4817 | TCGA-B0-4817-01A-01R-1276-13 | KIRC | MALE | 81 | T3c | N0 | M0 | Stage III | G3 | DECEASED | 33.48 | Unknown | Unknown |
| TCGA-BP-5004 | TCGA-BP-5004-01A-01R-1333-13 | KIRC | MALE | 53 | T1a | NX | M0 | Stage I | G3 | LIVING | 36.99 | DiseaseFree | 36.99 |
| TCGA-B8-4153 | TCGA-B8-4153-01B-11R-1671-13 | KIRC | MALE | 74 | T3a | NX | M0 | Stage III | G3 | LIVING | 25.03 | Recurred/Progressed | 7.33 |
| TCGA-B0-4698 | TCGA-B0-4698-01A-01R-1502-13 | KIRC | MALE | 75 | T4 | NX | M0 | Stage IV | G4 | DECEASED | 1.38 | Unknown | Unknown |
| TCGA-A3-3382 | TCGA-A3-3382-01A-01T-0860-13 | KIRC | MALE | 69 | T1b | NX | M0 | Stage I | G3 | LIVING | 18.86 | Recurred/Progressed | 16.89 |
| TCGA-A3-3319 | TCGA-A3-3319-01A-02R-1324-13 | KIRC | MALE | 70 | T1b | NX | M0 | Stage I | G2 | LIVING | 37.12 | DiseaseFree | 37.12 |
| TCGA-AK-3454 | TCGA-AK-3454-01A-02R-1276-13 | KIRC | MALE | 84 | T1b | NX | M0 | Stage I | G3 | LIVING | 28.71 | DiseaseFree | 28.71 |
| TCGA-B8-5163 | TCGA-B8-5163-01A-01R-1419-13 | KIRC | FEMALE | 63 | T3a | N0 | M0 | Stage III | G3 | LIVING | 27 | DiseaseFree | 27 |
| TCGA-CZ-5451 | TCGA-CZ-5451-01A-01R-1502-13 | KIRC | MALE | 74 | T2 | N0 | M0 | Stage II | G3 | LIVING | 63.37 | DiseaseFree | 63.37 |
| TCGA-CJ-4886 | TCGA-CJ-4886-01A-01R-1304-13 | KIRC | FEMALE | 42 | T1a | NX | M0 | Stage I | G3 | LIVING | 64.13 | DiseaseFree | 64.13 |
| TCGA-BP-4972 | TCGA-BP-4972-01A-01R-1333-13 | KIRC | FEMALE | 43 | T3a | NX | M0 | Stage III | G3 | LIVING | 49.34 | DiseaseFree | 49.34 |
| TCGA-B8-4143 | TCGA-B8-4143-01A-01T-1187-13 | KIRC | FEMALE | 66 | T3a | N0 | M1 | Stage IV | G3 | DECEASED | 23.29 | Unknown | Unknown |
| TCGA-B0-5706 | TCGA-B0-5706-01A-11R-1540-13 | KIRC | MALE | 45 | T2 | N0 | M0 | Stage II | G2 | LIVING | 105.29 | DiseaseFree | 105.29 |
| TCGA-CJ-4639 | TCGA-CJ-4639-01A-01R-1187-13 | KIRC | FEMALE | 49 | T2 | N0 | M0 | Stage II | G2 | LIVING | 106.08 | DiseaseFree | 106.08 |
| TCGA-BP-4332 | TCGA-BP-4332-01A-01R-1288-13 | KIRC | MALE | 36 | T3a | N0 | M0 | Stage III | G2 | LIVING | 37.22 | DiseaseFree | 37.22 |
| TCGA-G6-A8L6 | TCGA-G6-A8L6-01A-11R-A37P-13 | KIRC | MALE | 55 | T2a | NX | MX | Stage IV | G3 | DECEASED | 10.28 | Recurred/Progressed | 9.99 |
| TCGA-BP-5168 | TCGA-BP-5168-01A-01R-1419-13 | KIRC | MALE | 75 | T1a | NX | M0 | Stage I | G2 | DECEASED | 48.06 | Unknown | Unknown |
| TCGA-BP-4169 | TCGA-BP-4169-01A-02R-1288-13 | KIRC | FEMALE | 76 | T2 | N0 | M0 | Stage II | G2 | DECEASED | 23.03 | Recurred/Progressed | 2.56 |
| TCGA-B2-5641 | TCGA-B2-5641-01A-01R-1540-13 | KIRC | MALE | 79 | T1a | N0 | M0 | Stage I | G3 | LIVING | 21.55 | DiseaseFree | 21.55 |
| TCGA-CZ-4862 | TCGA-CZ-4862-01A-01R-1304-13 | KIRC | MALE | 46 | T1b | NX | M0 | Stage I | G2 | LIVING | 107.46 | DiseaseFree | 107.46 |
| TCGA-B0-5710 | TCGA-B0-5710-01A-11R-1671-13 | KIRC | MALE | 57 | T1b | N0 | M0 | Stage I | G2 | LIVING | 79.83 | Recurred/Progressed | 47.54 |
| TCGA-B2-3923 | TCGA-B2-3923-01A-02R-A27D-13 | KIRC | MALE | 59 | T2 | NX | M0 | Stage II | G2 | LIVING | 32.59 | DiseaseFree | 32.59 |
| TCGA-B4-5844 | TCGA-B4-5844-01A-11R-1671-13 | KIRC | FEMALE | 61 | T2 | N0 | M0 | Stage II | G1 | LIVING | 0.23 | DiseaseFree | 0.23 |
| TCGA-BP-5200 | TCGA-BP-5200-01A-01R-1425-13 | KIRC | MALE | 44 | T2 | NX | M0 | Stage II | G4 | LIVING | 34.92 | DiseaseFree | 34.92 |
| TCGA-CJ-5677 | TCGA-CJ-5677-01A-11R-1540-13 | KIRC | FEMALE | 54 | T3a | NX | M1 | Stage IV | G4 | DECEASED | 25.69 | Recurred/Progressed | 9.46 |
| TCGA-BP-4988 | TCGA-BP-4988-01A-01R-1333-13 | KIRC | MALE | 72 | T1a | N0 | M0 | Stage I | G2 | DECEASED | 27.2 | Unknown | Unknown |
| TCGA-B4-5835 | TCGA-B4-5835-01A-11R-1671-13 | KIRC | FEMALE | 64 | T1 | N0 | M0 | Stage I | G2 | LIVING | 0.53 | DiseaseFree | 0.53 |
| TCGA-BP-5186 | TCGA-BP-5186-01A-01R-1425-13 | KIRC | FEMALE | 50 | T1a | N0 | M0 | Stage I | G2 | LIVING | 22.77 | DiseaseFree | 22.77 |
| TCGA-BP-4961 | TCGA-BP-4961-01A-01R-1762-13 | KIRC | MALE | 47 | T1a | NX | M0 | Stage I | G2 | LIVING | 63.57 | DiseaseFree | 63.57 |
| TCGA-CJ-4895 | TCGA-CJ-4895-01A-01R-1304-13 | KIRC | MALE | 62 | T3a | NX | M1 | Stage IV | G4 | DECEASED | 39.42 | Recurred/Progressed | 2 |
| TCGA-CJ-4899 | TCGA-CJ-4899-01A-01R-1762-13 | KIRC | MALE | 42 | T1b | NX | M0 | Stage I | G2 | LIVING | 50.2 | DiseaseFree | 50.2 |
| TCGA-B0-5092 | TCGA-B0-5092-01A-01R-1419-13 | KIRC | FEMALE | 53 | T1a | N0 | M1 | Stage IV | G3 | DECEASED | 15.08 | Unknown | Unknown |
| TCGA-CZ-5452 | TCGA-CZ-5452-01A-01R-1502-13 | KIRC | MALE | 69 | T2 | N0 | M0 | Stage II | G2 | LIVING | 58.77 | DiseaseFree | 58.77 |
| TCGA-B0-4814 | TCGA-B0-4814-01A-01R-1276-13 | KIRC | MALE | 58 | T4 | N0 | M1 | Stage IV | G3 | DECEASED | 5.52 | Unknown | Unknown |
| TCGA-AK-3460 | TCGA-AK-3460-01A-02R-1276-13 | KIRC | MALE | 58 | T1a | NX | M0 | Stage I | G2 | LIVING | 82.39 | DiseaseFree | 82.39 |
| TCGA-BP-4329 | TCGA-BP-4329-01A-02R-1288-13 | KIRC | MALE | 75 | T3a | N0 | M0 | Stage III | G2 | DECEASED | 27.76 | Recurred/Progressed | 23 |
| TCGA-B8-A54G | TCGA-B8-A54G-01A-11R-A25Z-13 | KIRC | MALE | 50 | T1a | NX | MX | Stage I | G3 | LIVING | 1.74 | DiseaseFree | 1.74 |
| TCGA-BP-4777 | TCGA-BP-4777-01A-01R-1288-13 | KIRC | MALE | 46 | T1a | NX | M0 | Stage I | G3 | LIVING | 56.87 | DiseaseFree | 56.87 |
| TCGA-A3-3370 | TCGA-A3-3370-01A-02R-1419-13 | KIRC | FEMALE | 48 | T1b | N0 | M0 | Stage I | G2 | LIVING | 74.7 | DiseaseFree | 74.7 |
| TCGA-BP-4782 | TCGA-BP-4782-01A-02R-1419-13 | KIRC | FEMALE | 55 | T1a | NX | M0 | Stage I | G2 | LIVING | 11.63 | DiseaseFree | 11.63 |
| TCGA-A3-A8OW | TCGA-A3-A8OW-01A-11R-A37P-13 | KIRC | MALE | 37 | T3a | NX | MX | Stage III | G2 | LIVING | 10.61 | DiseaseFree | 10.61 |
| TCGA-CJ-4916 | TCGA-CJ-4916-01A-01R-1425-13 | KIRC | FEMALE | 69 | T3a | NX | M0 | Stage III | G3 | LIVING | 45.11 | DiseaseFree | 45.11 |
| TCGA-B0-5102 | TCGA-B0-5102-01A-01R-1419-13 | KIRC | FEMALE | 74 | T1 | NX | M0 | Stage I | G3 | DECEASED | 90.8 | Recurred/Progressed | 89.82 |
| TCGA-B0-5695 | TCGA-B0-5695-01A-11R-1540-13 | KIRC | FEMALE | 61 | T1b | N0 | M0 | Stage I | G2 | LIVING | 70.63 | DiseaseFree | 70.63 |
| TCGA-BP-4789 | TCGA-BP-4789-01A-01R-1304-13 | KIRC | MALE | 48 | T1a | NX | M0 | Stage I | G2 | LIVING | 48.92 | DiseaseFree | 48.92 |
| TCGA-A3-3323 | TCGA-A3-3323-01A-02R-1324-13 | KIRC | MALE | 53 | T1b | NX | M0 | Stage I | G1 | LIVING | 36.33 | DiseaseFree | 36.33 |
| TCGA-BP-4775 | TCGA-BP-4775-01A-01R-1288-13 | KIRC | FEMALE | 55 | T1a | NX | M0 | Stage I | G2 | LIVING | 60.55 | DiseaseFree | 60.55 |
| TCGA-B8-A54H | TCGA-B8-A54H-01A-11R-A33A-13 | KIRC | FEMALE | 69 | T2a | N0 | MX | Stage II | G3 | LIVING | 8.41 | DiseaseFree | 8.41 |
| TCGA-B0-4827 | TCGA-B0-4827-01A-02R-1419-13 | KIRC | FEMALE | 77 | T3b | N0 | M0 | Stage III | G4 | DECEASED | 29.07 | Recurred/Progressed | 21.94 |
| TCGA-A3-3385 | TCGA-A3-3385-01A-02R-1419-13 | KIRC | FEMALE | 46 | T1a | N0 | M0 | Stage I | G2 | LIVING | 65.47 | DiseaseFree | 65.47 |
| TCGA-B0-5080 | TCGA-B0-5080-01A-01R-1502-13 | KIRC | MALE | 63 | T3a | N0 | M1 | Stage IV | G3 | DECEASED | 11.24 | Recurred/Progressed | 3.38 |
| TCGA-A3-3357 | TCGA-A3-3357-01A-02R-1419-13 | KIRC | MALE | 62 | T2 | N0 | M0 | Stage II | G3 | LIVING | 88.3 | DiseaseFree | 88.3 |
| TCGA-AK-3440 | TCGA-AK-3440-01A-02R-1276-13 | KIRC | MALE | 58 | T1a | NX | M0 | Stage I | G3 | LIVING | 94.12 | DiseaseFree | 94.12 |
| TCGA-CZ-5986 | TCGA-CZ-5986-01A-11R-1671-13 | KIRC | MALE | 61 | T1 | N0 | M0 | Stage I | G3 | LIVING | 12.25 | DiseaseFree | 12.25 |
| TCGA-A3-A8OV | TCGA-A3-A8OV-01A-11R-A37P-13 | KIRC | MALE | 75 | T1a | NX | MX | Stage I | G2 | LIVING | 11.17 | DiseaseFree | 11.17 |
| TCGA-B4-5838 | TCGA-B4-5838-01A-11R-1671-13 | KIRC | MALE | 52 | T3 | N1 | M0 | Stage III | G2 | LIVING | 5.45 | DiseaseFree | 5.45 |
| TCGA-CZ-5455 | TCGA-CZ-5455-01A-01R-1502-13 | KIRC | MALE | 63 | T3b | NX | M1 | Stage IV | G4 | DECEASED | 18.43 | Unknown | Unknown |
| TCGA-B0-5100 | TCGA-B0-5100-01A-01R-1419-13 | KIRC | MALE | 72 | T3a | NX | M0 | Stage III | G3 | DECEASED | 62.84 | Recurred/Progressed | 46.85 |
| TCGA-BP-5199 | TCGA-BP-5199-01A-01R-1425-13 | KIRC | MALE | 58 | T2 | N0 | M0 | Stage II | G4 | LIVING | 44.51 | DiseaseFree | 44.51 |
| TCGA-EU-5905 | TCGA-EU-5905-01A-11R-1671-13 | KIRC | FEMALE | 67 | T1 | NX | M0 | Stage I | G3 | LIVING | 3.91 | DiseaseFree | 3.91 |
| TCGA-B0-5096 | TCGA-B0-5096-01A-01R-1419-13 | KIRC | FEMALE | 72 | T3a | N1 | M0 | Stage III | GX | DECEASED | 2.23 | Unknown | Unknown |
| TCGA-B8-4154 | TCGA-B8-4154-01A-01T-1187-13 | KIRC | FEMALE | 73 | T1a | N0 | M0 | Stage I | G2 | LIVING | 45.34 | DiseaseFree | 45.34 |
| TCGA-B0-5113 | TCGA-B0-5113-01A-01R-1419-13 | KIRC | FEMALE | 69 | T3a | N0 | M0 | Stage III | G2 | LIVING | 38.6 | DiseaseFree | 38.6 |
| TCGA-CZ-5458 | TCGA-CZ-5458-01A-01R-1502-13 | KIRC | MALE | 43 | T3a | NX | M0 | Stage III | G3 | LIVING | 91.62 | DiseaseFree | 91.62 |
| TCGA-B0-4696 | TCGA-B0-4696-01A-01R-1276-13 | KIRC | MALE | 58 | T3a | N0 | M0 | Stage III | G3 | DECEASED | 28.45 | Recurred/Progressed | 26.08 |
| TCGA-AK-3453 | TCGA-AK-3453-01A-02R-1276-13 | KIRC | FEMALE | 58 | T2 | NX | M0 | Stage II | G2 | LIVING | 83.15 | DiseaseFree | 83.15 |
| TCGA-B0-5119 | TCGA-B0-5119-01A-02R-1419-13 | KIRC | FEMALE | 61 | T1b | N0 | M0 | Stage I | G2 | LIVING | 50.99 | DiseaseFree | 50.99 |
| TCGA-B0-5700 | TCGA-B0-5700-01A-11R-1540-13 | KIRC | MALE | 77 | T1a | N0 | M0 | Stage I | G2 | LIVING | 58.8 | DiseaseFree | 58.8 |
| TCGA-B0-4822 | TCGA-B0-4822-01A-01R-1276-13 | KIRC | MALE | 78 | T2 | NX | M0 | Stage II | G4 | DECEASED | 36.5 | Unknown | Unknown |
| TCGA-B4-5836 | TCGA-B4-5836-01A-11R-1671-13 | KIRC | FEMALE | 61 | T1b | N0 | M0 | Stage I | G2 | LIVING | 4.63 | DiseaseFree | 4.63 |
| TCGA-B0-5713 | TCGA-B0-5713-01A-11R-1671-13 | KIRC | FEMALE | 75 | T3b | N0 | M0 | Stage III | G3 | LIVING | 91.39 | DiseaseFree | 91.39 |
| TCGA-B0-4846 | TCGA-B0-4846-01A-01R-1276-13 | KIRC | MALE | 52 | T3a | N0 | M1 | Stage IV | G2 | DECEASED | 39.42 | Recurred/Progressed | 5.35 |
| TCGA-CJ-4643 | TCGA-CJ-4643-01A-01R-1187-13 | KIRC | FEMALE | 67 | T2b | N0 | M0 | Stage II | G3 | LIVING | 58.9 | DiseaseFree | 58.9 |
| TCGA-B8-5159 | TCGA-B8-5159-01A-01R-1419-13 | KIRC | FEMALE | 61 | T1a | N0 | M0 | Stage I | G3 | LIVING | 23.72 | DiseaseFree | 23.72 |
| TCGA-AK-3426 | TCGA-AK-3426-01A-02R-1324-13 | KIRC | MALE | 37 | T3a | N1 | M0 | Stage III | G3 | DECEASED | 29.07 | Recurred/Progressed | 8.41 |
| TCGA-CJ-4641 | TCGA-CJ-4641-01A-01R-1187-13 | KIRC | FEMALE | 55 | T3a | NX | M1 | Stage IV | G4 | DECEASED | 54.57 | Recurred/Progressed | -11.79 |
| TCGA-BP-5181 | TCGA-BP-5181-01A-01R-1425-13 | KIRC | FEMALE | 58 | T1b | NX | M0 | Stage I | G2 | LIVING | 49.11 | DiseaseFree | 49.11 |
| TCGA-BP-4177 | TCGA-BP-4177-01A-02R-1419-13 | KIRC | MALE | 65 | T1a | NX | M0 | Stage I | G2 | LIVING | 54.86 | DiseaseFree | 54.86 |
| TCGA-A3-3322 | TCGA-A3-3322-01A-02R-1324-13 | KIRC | MALE | 51 | T1a | NX | M0 | Stage I | G2 | LIVING | 48.55 | DiseaseFree | 48.55 |
| TCGA-A3-3317 | TCGA-A3-3317-01A-01T-0860-13 | KIRC | MALE | 67 | T2 | N0 | M0 | Stage II | G2 | LIVING | 48.98 | Recurred/Progressed | 31.27 |
| TCGA-B2-4099 | TCGA-B2-4099-01A-02T-1187-13 | KIRC | MALE | 83 | T1a | NX | M0 | Stage I | G3 | LIVING | 31.93 | DiseaseFree | 31.93 |
| TCGA-BP-4167 | TCGA-BP-4167-01A-02R-1324-13 | KIRC | MALE | 59 | T3a | NX | M0 | Stage III | G2 | LIVING | 89.29 | DiseaseFree | 89.29 |
| TCGA-A3-A8OU | TCGA-A3-A8OU-01A-11R-A37P-13 | KIRC | FEMALE | 74 | T1a | NX | MX | Stage I | G1 | LIVING | 0 | DiseaseFree | 0 |
| TCGA-BP-4760 | TCGA-BP-4760-01A-02R-1419-13 | KIRC | MALE | 69 | T1a | NX | M0 | Stage I | G2 | LIVING | 77.56 | Recurred/Progressed | 47.86 |
| TCGA-A3-3322 | TCGA-A3-3322-01A-01T-0860-13 | KIRC | MALE | 51 | T1a | NX | M0 | Stage I | G2 | LIVING | 48.55 | DiseaseFree | 48.55 |
| TCGA-AK-3458 | TCGA-AK-3458-01A-01R-1502-13 | KIRC | MALE | 48 | T1b | NX | M0 | Stage I | G3 | LIVING | 38.37 | DiseaseFree | 38.37 |
| TCGA-BP-4960 | TCGA-BP-4960-01A-01R-1762-13 | KIRC | MALE | 46 | T2 | N0 | M0 | Stage II | G3 | LIVING | 71.35 | DiseaseFree | 71.35 |
| TCGA-MM-A563 | TCGA-MM-A563-01A-11R-A25Z-13 | KIRC | MALE | 41 | T3 | NX | MX | Unknown | G2 | LIVING | 19.42 | DiseaseFree | 19.42 |
| TCGA-CW-5583 | TCGA-CW-5583-01A-02R-1540-13 | KIRC | FEMALE | 51 | T1a | NX | M0 | Stage I | G2 | LIVING | 81.77 | DiseaseFree | 81.77 |
| TCGA-B0-4823 | TCGA-B0-4823-01A-02R-1419-13 | KIRC | MALE | 88 | T1a | N0 | M0 | Stage I | G2 | DECEASED | 14.91 | Unknown | Unknown |
| TCGA-A3-3358 | TCGA-A3-3358-01A-01R-1540-13 | KIRC | FEMALE | 57 | T1a | N0 | M0 | Stage I | G2 | LIVING | 42.94 | DiseaseFree | 42.94 |
| TCGA-B0-5707 | TCGA-B0-5707-01A-11R-1540-13 | KIRC | FEMALE | 39 | T1a | N0 | M0 | Stage I | G3 | LIVING | 123 | DiseaseFree | 123 |
| TCGA-B0-5694 | TCGA-B0-5694-01A-11R-1540-13 | KIRC | MALE | 71 | T3a | N0 | M0 | Stage III | G3 | DECEASED | 15.77 | Recurred/Progressed | 9.46 |
| TCGA-BP-4159 | TCGA-BP-4159-01A-02R-1288-13 | KIRC | MALE | 70 | T1b | N0 | M0 | Stage I | G2 | DECEASED | 85.45 | Recurred/Progressed | 77.27 |
| TCGA-BP-4330 | TCGA-BP-4330-01A-01R-1288-13 | KIRC | FEMALE | 60 | T3a | N0 | M0 | Stage III | G2 | LIVING | 62.02 | DiseaseFree | 62.02 |
| TCGA-AK-3456 | TCGA-AK-3456-01A-02R-1324-13 | KIRC | MALE | 48 | T2 | N0 | M0 | Stage II | G3 | LIVING | 37.55 | DiseaseFree | 37.55 |
| TCGA-BP-4759 | TCGA-BP-4759-01A-01R-1762-13 | KIRC | MALE | 50 | T1a | NX | M0 | Stage I | G2 | LIVING | 77.92 | DiseaseFree | 77.92 |
| TCGA-B0-4816 | TCGA-B0-4816-01A-01R-1502-13 | KIRC | MALE | 49 | T2 | N0 | M0 | Stage II | G3 | DECEASED | 45.04 | Unknown | Unknown |
| TCGA-A3-3326 | TCGA-A3-3326-01A-01T-0860-13 | KIRC | MALE | 47 | T1a | NX | M0 | Stage I | G1 | LIVING | 37.35 | DiseaseFree | 37.35 |
| TCGA-CJ-4902 | TCGA-CJ-4902-01A-01R-1425-13 | KIRC | MALE | 61 | T3a | NX | M0 | Stage III | G3 | LIVING | 49.93 | DiseaseFree | 49.93 |
| TCGA-CJ-4897 | TCGA-CJ-4897-01A-03R-1425-13 | KIRC | FEMALE | 79 | T3a | NX | M0 | Stage III | G3 | LIVING | 109.76 | Recurred/Progressed | 46.75 |
| TCGA-CJ-5678 | TCGA-CJ-5678-01A-11R-1540-13 | KIRC | MALE | 62 | T2b | N0 | M1 | Stage IV | G3 | DECEASED | 18.86 | Recurred/Progressed | 13.11 |
| TCGA-A3-3363 | TCGA-A3-3363-01A-01T-0860-13 | KIRC | MALE | 50 | T2 | N0 | M0 | Stage II | G2 | LIVING | 10.48 | DiseaseFree | 10.48 |
| TCGA-DV-5566 | TCGA-DV-5566-01A-01R-1540-13 | KIRC | FEMALE | 67 | T1a | NX | M0 | Stage I | G2 | LIVING | 45.93 | DiseaseFree | 45.93 |
| TCGA-BP-4995 | TCGA-BP-4995-01A-01R-1333-13 | KIRC | MALE | 68 | T1b | N0 | M0 | Stage I | G3 | LIVING | 45.04 | DiseaseFree | 45.04 |
| TCGA-B2-5635 | TCGA-B2-5635-01A-01R-1540-13 | KIRC | MALE | 74 | T1a | NX | M0 | Stage I | G2 | LIVING | 24.8 | DiseaseFree | 24.8 |
| TCGA-B0-5692 | TCGA-B0-5692-01A-11R-1540-13 | KIRC | FEMALE | 66 | T3b | N0 | M0 | Stage III | G3 | LIVING | 129.57 | DiseaseFree | 129.57 |
| TCGA-BP-4337 | TCGA-BP-4337-01A-01R-1288-13 | KIRC | FEMALE | 76 | T3b | N0 | M0 | Stage III | G4 | DECEASED | 0.07 | Unknown | Unknown |
| TCGA-B4-5378 | TCGA-B4-5378-01A-01R-1502-13 | KIRC | MALE | 62 | T1 | N0 | M0 | Stage I | G2 | LIVING | 5.75 | DiseaseFree | 5.75 |
| TCGA-B0-4703 | TCGA-B0-4703-01A-01R-1276-13 | KIRC | MALE | 51 | T3a | N0 | M1 | Stage IV | G4 | DECEASED | 5.98 | Unknown | Unknown |
| TCGA-A3-A6NN | TCGA-A3-A6NN-01A-12R-A33A-13 | KIRC | MALE | 78 | T1a | NX | MX | Stage I | G2 | LIVING | 0.1 | DiseaseFree | 0.1 |
| TCGA-B8-4146 | TCGA-B8-4146-01B-11R-1671-13 | KIRC | FEMALE | 41 | T1b | NX | M0 | Stage I | G2 | LIVING | 16.79 | DiseaseFree | 16.79 |
| TCGA-MM-A564 | TCGA-MM-A564-01A-11R-A25Z-13 | KIRC | MALE | 68 | T2a | NX | MX | Stage II | G2 | LIVING | 19.94 | DiseaseFree | 19.94 |
| TCGA-B8-5551 | TCGA-B8-5551-01A-01R-1540-13 | KIRC | FEMALE | 65 | T1b | N0 | M0 | Stage I | G3 | LIVING | 0.53 | DiseaseFree | 0.53 |
| TCGA-B0-5107 | TCGA-B0-5107-01A-01R-1419-13 | KIRC | FEMALE | 65 | T2 | N0 | M1 | Stage IV | G4 | DECEASED | 30.45 | Recurred/Progressed | 18.04 |
| TCGA-B0-4697 | TCGA-B0-4697-01A-01R-1276-13 | KIRC | FEMALE | 46 | T3b | NX | M1 | Stage IV | G4 | DECEASED | 18.99 | Unknown | Unknown |
| TCGA-A3-3383 | TCGA-A3-3383-01A-01T-0860-13 | KIRC | MALE | 52 | T1 | NX | M0 | Stage I | G2 | LIVING | 28.29 | DiseaseFree | 28.29 |
| TCGA-BP-4968 | TCGA-BP-4968-01A-01R-1333-13 | KIRC | MALE | 40 | T1b | N0 | M0 | Stage I | G3 | LIVING | 57.36 | DiseaseFree | 57.36 |
| TCGA-AK-3444 | TCGA-AK-3444-01A-02R-1324-13 | KIRC | FEMALE | 80 | T1b | NX | M0 | Stage I | G2 | LIVING | 48.32 | DiseaseFree | 48.32 |
| TCGA-CJ-5675 | TCGA-CJ-5675-01A-11R-1540-13 | KIRC | MALE | 70 | T2a | NX | M0 | Stage II | G3 | LIVING | 129.3 | Recurred/Progressed | 29.57 |
| TCGA-B0-5120 | TCGA-B0-5120-01A-01R-1419-13 | KIRC | FEMALE | 72 | T1a | N0 | M0 | Stage I | G2 | LIVING | 38.4 | DiseaseFree | 38.4 |
| TCGA-A3-3316 | TCGA-A3-3316-01A-01T-0860-13 | KIRC | MALE | 57 | T2 | NX | M0 | Stage II | G3 | LIVING | 49.05 | DiseaseFree | 49.05 |
| TCGA-BP-4991 | TCGA-BP-4991-01A-01R-1333-13 | KIRC | MALE | 54 | T1a | NX | M0 | Stage I | G2 | LIVING | 46.42 | DiseaseFree | 46.42 |
| TCGA-CJ-4908 | TCGA-CJ-4908-01A-01R-1425-13 | KIRC | MALE | 38 | T1a | NX | M0 | Stage I | G2 | LIVING | 50.3 | DiseaseFree | 50.3 |
| TCGA-B2-5633 | TCGA-B2-5633-01B-04R-A27D-13 | KIRC | MALE | 56 | T1b | N0 | M0 | Stage I | G2 | LIVING | 31.64 | DiseaseFree | 31.64 |
| TCGA-CJ-4869 | TCGA-CJ-4869-01A-02R-1425-13 | KIRC | MALE | 49 | T2 | N1 | M0 | Stage III | G2 | LIVING | 83.9 | Recurred/Progressed | 57.62 |
| TCGA-A3-3346 | TCGA-A3-3346-01A-01T-1192-13 | KIRC | MALE | 68 | T1b | NX | M0 | Stage I | G3 | DECEASED | 4.5 | Recurred/Progressed | 3.75 |
| TCGA-B0-4852 | TCGA-B0-4852-01A-01R-1502-13 | KIRC | FEMALE | 78 | T2 | N0 | M0 | Stage II | G2 | DECEASED | 36.83 | Recurred/Progressed | 28.25 |
| TCGA-CJ-5684 | TCGA-CJ-5684-01A-11R-1540-13 | KIRC | MALE | 61 | T3a | NX | M0 | Stage III | G2 | LIVING | 73.29 | DiseaseFree | 73.29 |
| TCGA-CZ-5984 | TCGA-CZ-5984-01A-11R-1671-13 | KIRC | MALE | 51 | T1b | N0 | M0 | Stage I | G3 | LIVING | 67.9 | DiseaseFree | 67.9 |
| TCGA-A3-3351 | TCGA-A3-3351-01A-02R-1324-13 | KIRC | MALE | 42 | T2a | N0 | M0 | Stage II | G2 | LIVING | 29.89 | DiseaseFree | 29.89 |
| TCGA-BP-4976 | TCGA-BP-4976-01A-01R-1333-13 | KIRC | MALE | 77 | T1a | NX | M0 | Stage I | G3 | LIVING | 53.61 | DiseaseFree | 53.61 |
| TCGA-BP-4963 | TCGA-BP-4963-01A-01R-1333-13 | KIRC | MALE | 63 | T1b | NX | M0 | Stage I | G3 | LIVING | 60.25 | DiseaseFree | 60.25 |
| TCGA-BP-4335 | TCGA-BP-4335-01A-01R-1288-13 | KIRC | FEMALE | 65 | T3a | N0 | M1 | Stage IV | G3 | DECEASED | 15.6 | Recurred/Progressed | 14.91 |
| TCGA-B0-5110 | TCGA-B0-5110-01A-01R-1419-13 | KIRC | FEMALE | 71 | T1a | N0 | M0 | Stage I | G2 | LIVING | 66 | DiseaseFree | 66 |
| TCGA-B0-5701 | TCGA-B0-5701-01A-11R-1540-13 | KIRC | MALE | 65 | T3b | N0 | M0 | Stage III | G4 | LIVING | 80.85 | Recurred/Progressed | 7.36 |
| TCGA-DV-A4VZ | TCGA-DV-A4VZ-01A-11R-A25Z-13 | KIRC | MALE | 53 | T1a | NX | MX | Stage I | G2 | LIVING | 11.99 | DiseaseFree | 11.99 |
| TCGA-AK-3453 | TCGA-AK-3453-01A-01T-0860-13 | KIRC | FEMALE | 58 | T2 | NX | M0 | Stage II | G2 | LIVING | 83.15 | DiseaseFree | 83.15 |
| TCGA-B0-5696 | TCGA-B0-5696-01A-11R-1540-13 | KIRC | MALE | 69 | T3a | N0 | M0 | Stage III | G4 | LIVING | 85.71 | Recurred/Progressed | 56.73 |
| TCGA-BP-5202 | TCGA-BP-5202-01A-02R-1425-13 | KIRC | MALE | 75 | T3a | NX | M0 | Stage III | G2 | LIVING | 0.95 | DiseaseFree | 0.95 |
| TCGA-CJ-4884 | TCGA-CJ-4884-01A-01R-1304-13 | KIRC | FEMALE | 72 | T3a | NX | M0 | Stage III | G3 | LIVING | 57.79 | DiseaseFree | 57.79 |
| TCGA-CZ-5454 | TCGA-CZ-5454-01A-01R-1502-13 | KIRC | MALE | 63 | T2 | N0 | M1 | Stage IV | G2 | DECEASED | 23.72 | Recurred/Progressed | 13.9 |
| TCGA-BP-4807 | TCGA-BP-4807-01A-01R-1304-13 | KIRC | MALE | 42 | T1a | NX | M0 | Stage I | G3 | LIVING | 6.93 | DiseaseFree | 6.93 |
| TCGA-BP-5184 | TCGA-BP-5184-01A-01R-1425-13 | KIRC | MALE | 54 | T1a | NX | M0 | Stage I | G3 | LIVING | 37.22 | DiseaseFree | 37.22 |
| TCGA-DV-A4W0 | TCGA-DV-A4W0-05A-11R-A25Z-13 | KIRC | MALE | 55 | T1b | NX | MX | Stage I | G3 | LIVING | 81.14 | Recurred/Progressed | 65.97 |
| TCGA-CJ-4644 | TCGA-CJ-4644-01A-01R-1187-13 | KIRC | FEMALE | 48 | T3a | N0 | M1 | Stage IV | G3 | DECEASED | 11.04 | Recurred/Progressed | 3.06 |
| TCGA-CJ-5679 | TCGA-CJ-5679-01A-11R-1540-13 | KIRC | MALE | 73 | T3b | NX | M0 | Stage III | G4 | DECEASED | 22.31 | Recurred/Progressed | 19.55 |
| TCGA-B8-5545 | TCGA-B8-5545-01A-01R-1671-13 | KIRC | MALE | 42 | T1a | N0 | M0 | Stage I | G2 | LIVING | 50.1 | DiseaseFree | 50.1 |
| TCGA-A3-3313 | TCGA-A3-3313-01A-02R-1324-13 | KIRC | MALE | 59 | T1b | N0 | M0 | Stage I | G3 | DECEASED | 24.15 | Unknown | Unknown |
| TCGA-AK-3465 | TCGA-AK-3465-01A-01T-0860-13 | KIRC | FEMALE | 71 | T1b | NX | M0 | Stage I | GX | LIVING | 12.12 | DiseaseFree | 12.12 |
| TCGA-BP-4165 | TCGA-BP-4165-01A-02R-1288-13 | KIRC | FEMALE | 64 | T1b | N0 | M0 | Stage I | G1 | LIVING | 99.77 | Recurred/Progressed | 60.38 |
| TCGA-B8-4620 | TCGA-B8-4620-01A-01R-1187-13 | KIRC | FEMALE | 70 | T3a | N0 | M0 | Stage III | G2 | LIVING | 25.53 | Recurred/Progressed | 19.84 |
| TCGA-B0-4821 | TCGA-B0-4821-01A-01R-1502-13 | KIRC | FEMALE | 68 | T3b | N0 | M0 | Stage III | G3 | DECEASED | 40.41 | Unknown | Unknown |
| TCGA-CJ-4901 | TCGA-CJ-4901-01A-01R-1425-13 | KIRC | MALE | 47 | T3b | NX | M0 | Stage III | G3 | LIVING | 47.63 | DiseaseFree | 47.63 |
| TCGA-BP-5173 | TCGA-BP-5173-01A-01R-1425-13 | KIRC | MALE | 75 | T1a | NX | M0 | Stage I | G2 | DECEASED | 2.04 | Unknown | Unknown |
| TCGA-CW-5588 | TCGA-CW-5588-01A-01R-1540-13 | KIRC | FEMALE | 78 | T1a | NX | M0 | Stage I | G2 | LIVING | 66.26 | DiseaseFree | 66.26 |
| TCGA-CJ-4870 | TCGA-CJ-4870-01A-01R-1762-13 | KIRC | FEMALE | 58 | T3a | NX | M0 | Stage III | G2 | LIVING | 49.21 | DiseaseFree | 49.21 |
| TCGA-BP-5192 | TCGA-BP-5192-01A-01R-1425-13 | KIRC | MALE | 59 | T1a | NX | M0 | Stage I | G2 | LIVING | 23.46 | DiseaseFree | 23.46 |
| TCGA-CJ-5683 | TCGA-CJ-5683-01A-11R-1540-13 | KIRC | MALE | 78 | T1b | NX | M0 | Stage I | G3 | LIVING | 62.06 | DiseaseFree | 62.06 |
| TCGA-CZ-5469 | TCGA-CZ-5469-01A-01R-1502-13 | KIRC | MALE | 41 | T2 | N0 | M0 | Stage II | G2 | DECEASED | 31.08 | Recurred/Progressed | 3.61 |
| TCGA-B0-5116 | TCGA-B0-5116-01A-02R-1419-13 | KIRC | MALE | 52 | T3b | N0 | M0 | Stage III | G3 | LIVING | 41.85 | Recurred/Progressed | 37.09 |
| TCGA-BP-4771 | TCGA-BP-4771-01A-01R-1762-13 | KIRC | MALE | 62 | T3a | N0 | M1 | Stage IV | G4 | DECEASED | 5.32 | Recurred/Progressed | 3.61 |
| TCGA-B2-5635 | TCGA-B2-5635-01B-04R-A27D-13 | KIRC | MALE | 74 | T1a | NX | M0 | Stage I | G2 | LIVING | 24.8 | DiseaseFree | 24.8 |
| TCGA-B8-4148 | TCGA-B8-4148-01A-02R-1324-13 | KIRC | FEMALE | 63 | T1a | N0 | M0 | Stage I | G3 | LIVING | 49.93 | DiseaseFree | 49.93 |
| TCGA-CZ-5464 | TCGA-CZ-5464-01A-01R-1502-13 | KIRC | MALE | 69 | T3b | NX | M1 | Stage IV | G2 | LIVING | 69.91 | DiseaseFree | 69.91 |
| TCGA-CZ-5456 | TCGA-CZ-5456-01A-01R-1502-13 | KIRC | MALE | 57 | T2 | N0 | M0 | Stage II | G3 | LIVING | 79.57 | Recurred/Progressed | 31.24 |
| TCGA-B0-5084 | TCGA-B0-5084-01A-01R-1762-13 | KIRC | MALE | 33 | T3a | N1 | M1 | Stage IV | G3 | DECEASED | 7.29 | Recurred/Progressed | 2.14 |
| TCGA-B0-4841 | TCGA-B0-4841-01A-01R-1276-13 | KIRC | MALE | 63 | T2 | NX | M1 | Stage IV | G3 | DECEASED | 6.7 | Recurred/Progressed | 3.55 |
| TCGA-AK-3443 | TCGA-AK-3443-01A-01T-0860-13 | KIRC | MALE | 45 | T2 | N0 | M0 | Stage II | GX | LIVING | 46.75 | DiseaseFree | 46.75 |
| TCGA-G6-A5PC | TCGA-G6-A5PC-01A-11R-A33A-13 | KIRC | FEMALE | 54 | T1b | N0 | M1 | Stage IV | G4 | DECEASED | 7.95 | Unknown | Unknown |
| TCGA-B2-4101 | TCGA-B2-4101-01A-02R-1276-13 | KIRC | MALE | 52 | T2a | NX | M0 | Stage II | G3 | LIVING | 21.29 | DiseaseFree | 21.29 |
| TCGA-A3-3331 | TCGA-A3-3331-01A-01T-0860-13 | KIRC | FEMALE | 86 | T1 | N0 | M0 | Stage I | G2 | LIVING | 48.78 | DiseaseFree | 48.78 |
| TCGA-CJ-5680 | TCGA-CJ-5680-01A-11R-1540-13 | KIRC | FEMALE | 65 | T3a | NX | M1 | Stage IV | G4 | DECEASED | 25.23 | Recurred/Progressed | 5.03 |
| TCGA-B0-5400 | TCGA-B0-5400-01A-01R-1502-13 | KIRC | FEMALE | 59 | T3b | N0 | M0 | Stage III | G4 | LIVING | 56.93 | DiseaseFree | 56.93 |
| TCGA-B0-4706 | TCGA-B0-4706-01A-01R-1502-13 | KIRC | MALE | 61 | T3a | NX | M0 | Stage III | G4 | DECEASED | 2.14 | Unknown | Unknown |
| TCGA-CJ-4635 | TCGA-CJ-4635-01A-02R-1304-13 | KIRC | MALE | 48 | T1b | NX | M0 | Stage I | G3 | LIVING | 46.52 | DiseaseFree | 46.52 |
| TCGA-B0-5691 | TCGA-B0-5691-01A-11R-1540-13 | KIRC | FEMALE | 66 | T1a | N0 | M0 | Stage I | G3 | LIVING | 112.71 | Recurred/Progressed | 106.77 |
| TCGA-BP-4769 | TCGA-BP-4769-01A-01R-1288-13 | KIRC | MALE | 63 | T1a | NX | M0 | Stage I | G2 | LIVING | 61.63 | DiseaseFree | 61.63 |
| TCGA-BP-4971 | TCGA-BP-4971-01A-01R-1762-13 | KIRC | MALE | 40 | T3a | N0 | M0 | Stage III | G3 | LIVING | 48.85 | DiseaseFree | 48.85 |
| TCGA-BP-4346 | TCGA-BP-4346-01A-01R-1288-13 | KIRC | MALE | 57 | T3b | N0 | M0 | Stage III | G3 | DECEASED | 49.05 | Unknown | Unknown |
| TCGA-CZ-4857 | TCGA-CZ-4857-01A-01R-1762-13 | KIRC | MALE | 56 | T3a | N0 | M1 | Stage IV | G3 | DECEASED | 47.04 | Recurred/Progressed | 32.52 |
| TCGA-BP-5170 | TCGA-BP-5170-01A-01R-1425-13 | KIRC | MALE | 55 | T1a | NX | M0 | Stage I | G2 | LIVING | 79.24 | DiseaseFree | 79.24 |
| TCGA-AK-3461 | TCGA-AK-3461-01A-02R-1276-13 | KIRC | MALE | 72 | T1a | NX | M0 | Stage I | G2 | LIVING | 72.83 | DiseaseFree | 72.83 |
| TCGA-B0-4690 | TCGA-B0-4690-01A-01R-1276-13 | KIRC | MALE | 65 | T4 | N0 | M1 | Stage IV | G3 | DECEASED | 1.41 | Unknown | Unknown |
| TCGA-BP-4765 | TCGA-BP-4765-01A-01R-1288-13 | KIRC | MALE | 43 | T1a | NX | M0 | Stage I | G2 | LIVING | 71.75 | DiseaseFree | 71.75 |
| TCGA-A3-3308 | TCGA-A3-3308-01A-01T-0860-13 | KIRC | FEMALE | 77 | T3b | N0 | M0 | Stage III | G2 | LIVING | 0.53 | DiseaseFree | 0.53 |
| TCGA-DV-5568 | TCGA-DV-5568-01A-01R-1540-13 | KIRC | MALE | 26 | T1a | NX | M0 | Stage I | G2 | LIVING | 12.16 | DiseaseFree | 12.16 |
| TCGA-BP-4994 | TCGA-BP-4994-01A-01R-1762-13 | KIRC | MALE | 54 | T1a | NX | M0 | Stage I | G3 | LIVING | 42.97 | DiseaseFree | 42.97 |
| TCGA-BP-4982 | TCGA-BP-4982-01A-01R-1762-13 | KIRC | MALE | 42 | T1b | NX | M0 | Stage I | G3 | LIVING | 33.31 | DiseaseFree | 33.31 |
| TCGA-B2-5641 | TCGA-B2-5641-11A-01R-1540-13 | KIRC | MALE | 79 | - | - | - | - | - | - | - | - | - |
| TCGA-CJ-5689 | TCGA-CJ-5689-11A-01R-1540-13 | KIRC | MALE | 90 | - | - | - | - | - | - | - | - | - |
| TCGA-B0-5703 | TCGA-B0-5703-11A-01R-1540-13 | KIRC | MALE | 73 | - | - | - | - | - | - | - | - | - |
| TCGA-CZ-5986 | TCGA-CZ-5986-11A-01R-1671-13 | KIRC | MALE | 61 | - | - | - | - | - | - | - | - | - |
| TCGA-B2-5636 | TCGA-B2-5636-11A-01R-1540-13 | KIRC | MALE | 79 | - | - | - | - | - | - | - | - | - |
| TCGA-CZ-5989 | TCGA-CZ-5989-11A-01R-1671-13 | KIRC | MALE | 60 | - | - | - | - | - | - | - | - | - |
| TCGA-B0-5402 | TCGA-B0-5402-11A-01R-1502-13 | KIRC | MALE | 64 | - | - | - | - | - | - | - | - | - |
| TCGA-CZ-4864 | TCGA-CZ-4864-11A-01R-1502-13 | KIRC | MALE | 86 | - | - | - | - | - | - | - | - | - |
| TCGA-CJ-5679 | TCGA-CJ-5679-11A-01R-1540-13 | KIRC | MALE | 73 | - | - | - | - | - | - | - | - | - |
| TCGA-CZ-5453 | TCGA-CZ-5453-11A-01R-1502-13 | KIRC | MALE | 67 | - | - | - | - | - | - | - | - | - |
| TCGA-B8-4622 | TCGA-B8-4622-11A-01R-1757-13 | KIRC | MALE | 57 | - | - | - | - | - | - | - | - | - |
| TCGA-B0-4700 | TCGA-B0-4700-11A-01R-1540-13 | KIRC | MALE | 60 | - | - | - | - | - | - | - | - | - |
| TCGA-B0-5711 | TCGA-B0-5711-11A-01R-1671-13 | KIRC | MALE | 50 | - | - | - | - | - | - | - | - | - |
| TCGA-CZ-5982 | TCGA-CZ-5982-11A-01R-1671-13 | KIRC | FEMALE | 59 | - | - | - | - | - | - | - | - | - |
| TCGA-B0-5712 | TCGA-B0-5712-11A-01R-1671-13 | KIRC | FEMALE | 68 | - | - | - | - | - | - | - | - | - |
| TCGA-CJ-5680 | TCGA-CJ-5680-11A-01R-1540-13 | KIRC | FEMALE | 65 | - | - | - | - | - | - | - | - | - |
| TCGA-B0-5694 | TCGA-B0-5694-11A-01R-1540-13 | KIRC | MALE | 71 | - | - | - | - | - | - | - | - | - |
| TCGA-CJ-5676 | TCGA-CJ-5676-11A-01R-1540-13 | KIRC | MALE | 47 | - | - | - | - | - | - | - | - | - |
| TCGA-CJ-5672 | TCGA-CJ-5672-11A-01R-1540-13 | KIRC | MALE | 84 | - | - | - | - | - | - | - | - | - |
| TCGA-B0-5697 | TCGA-B0-5697-11A-01R-1540-13 | KIRC | MALE | 50 | - | - | - | - | - | - | - | - | - |
| TCGA-CZ-5457 | TCGA-CZ-5457-11A-01R-1502-13 | KIRC | MALE | 62 | - | - | - | - | - | - | - | - | - |
| TCGA-CZ-5456 | TCGA-CZ-5456-11A-02R-1502-13 | KIRC | MALE | 57 | - | - | - | - | - | - | - | - | - |
| TCGA-B0-5699 | TCGA-B0-5699-11A-01R-1540-13 | KIRC | MALE | 53 | - | - | - | - | - | - | - | - | - |
| TCGA-CZ-5455 | TCGA-CZ-5455-11A-01R-1502-13 | KIRC | MALE | 63 | - | - | - | - | - | - | - | - | - |
| TCGA-CW-5587 | TCGA-CW-5587-11A-01R-1540-13 | KIRC | FEMALE | 62 | - | - | - | - | - | - | - | - | - |
| TCGA-B8-4620 | TCGA-B8-4620-11A-01R-1757-13 | KIRC | FEMALE | 70 | - | - | - | - | - | - | - | - | - |
| TCGA-CW-5589 | TCGA-CW-5589-11A-01R-1540-13 | KIRC | MALE | 52 | - | - | - | - | - | - | - | - | - |
| TCGA-B0-5696 | TCGA-B0-5696-11A-01R-1540-13 | KIRC | MALE | 69 | - | - | - | - | - | - | - | - | - |
| TCGA-CZ-5466 | TCGA-CZ-5466-11A-01R-1502-13 | KIRC | MALE | 67 | - | - | - | - | - | - | - | - | - |
| TCGA-CZ-5454 | TCGA-CZ-5454-11A-01R-1502-13 | KIRC | MALE | 63 | - | - | - | - | - | - | - | - | - |
| TCGA-B0-5709 | TCGA-B0-5709-11A-01R-1540-13 | KIRC | FEMALE | 62 | - | - | - | - | - | - | - | - | - |
| TCGA-CJ-5677 | TCGA-CJ-5677-11A-01R-1540-13 | KIRC | FEMALE | 54 | - | - | - | - | - | - | - | - | - |
| TCGA-CZ-5985 | TCGA-CZ-5985-11A-01R-1671-13 | KIRC | MALE | 58 | - | - | - | - | - | - | - | - | - |
| TCGA-CW-5581 | TCGA-CW-5581-11A-01R-1540-13 | KIRC | MALE | 44 | - | - | - | - | - | - | - | - | - |
| TCGA-CZ-5470 | TCGA-CZ-5470-11A-01R-1502-13 | KIRC | FEMALE | 72 | - | - | - | - | - | - | - | - | - |
| TCGA-B0-4712 | TCGA-B0-4712-11A-02R-1502-13 | KIRC | MALE | 76 | - | - | - | - | - | - | - | - | - |
| TCGA-CW-5584 | TCGA-CW-5584-11A-01R-1540-13 | KIRC | MALE | 74 | - | - | - | - | - | - | - | - | - |
| TCGA-CZ-5458 | TCGA-CZ-5458-11A-01R-1502-13 | KIRC | MALE | 43 | - | - | - | - | - | - | - | - | - |
| TCGA-CZ-5463 | TCGA-CZ-5463-11A-01R-1502-13 | KIRC | MALE | 76 | - | - | - | - | - | - | - | - | - |
| TCGA-CW-5580 | TCGA-CW-5580-11A-02R-1671-13 | KIRC | FEMALE | 73 | - | - | - | - | - | - | - | - | - |
| TCGA-B0-5705 | TCGA-B0-5705-11A-01R-1540-13 | KIRC | FEMALE | 65 | - | - | - | - | - | - | - | - | - |
| TCGA-A3-3358 | TCGA-A3-3358-11A-01R-1540-13 | KIRC | FEMALE | 57 | - | - | - | - | - | - | - | - | - |
| TCGA-CJ-6030 | TCGA-CJ-6030-11A-01R-1671-13 | KIRC | MALE | 65 | - | - | - | - | - | - | - | - | - |
| TCGA-CZ-5452 | TCGA-CZ-5452-11A-01R-1502-13 | KIRC | MALE | 69 | - | - | - | - | - | - | - | - | - |
| TCGA-B0-5706 | TCGA-B0-5706-11A-01R-1540-13 | KIRC | MALE | 45 | - | - | - | - | - | - | - | - | - |
| TCGA-CZ-5465 | TCGA-CZ-5465-11A-01R-1502-13 | KIRC | FEMALE | 76 | - | - | - | - | - | - | - | - | - |
| TCGA-CZ-5467 | TCGA-CZ-5467-11A-01R-1502-13 | KIRC | FEMALE | 86 | - | - | - | - | - | - | - | - | - |
| TCGA-B8-4619 | TCGA-B8-4619-11A-01R-1757-13 | KIRC | MALE | 58 | - | - | - | - | - | - | - | - | - |
| TCGA-B0-5691 | TCGA-B0-5691-11A-01R-1540-13 | KIRC | FEMALE | 66 | - | - | - | - | - | - | - | - | - |
| TCGA-CZ-5984 | TCGA-CZ-5984-11A-01R-1671-13 | KIRC | MALE | 51 | - | - | - | - | - | - | - | - | - |
| TCGA-CW-5591 | TCGA-CW-5591-11A-01R-1540-13 | KIRC | MALE | 56 | - | - | - | - | - | - | - | - | - |
| TCGA-CW-6090 | TCGA-CW-6090-11A-01R-1671-13 | KIRC | MALE | 68 | - | - | - | - | - | - | - | - | - |
| TCGA-CZ-5988 | TCGA-CZ-5988-11A-01R-1671-13 | KIRC | MALE | 38 | - | - | - | - | - | - | - | - | - |
| TCGA-CJ-5681 | TCGA-CJ-5681-11A-01R-1540-13 | KIRC | FEMALE | 44 | - | - | - | - | - | - | - | - | - |
| TCGA-B8-5549 | TCGA-B8-5549-11A-01R-1540-13 | KIRC | MALE | 53 | - | - | - | - | - | - | - | - | - |
| TCGA-CW-5585 | TCGA-CW-5585-11A-01R-1540-13 | KIRC | MALE | 51 | - | - | - | - | - | - | - | - | - |
| TCGA-CZ-5451 | TCGA-CZ-5451-11A-01R-1502-13 | KIRC | MALE | 74 | - | - | - | - | - | - | - | - | - |
| TCGA-B8-5552 | TCGA-B8-5552-11A-01R-1671-13 | KIRC | FEMALE | 41 | - | - | - | - | - | - | - | - | - |
| TCGA-CZ-4865 | TCGA-CZ-4865-11A-01R-1502-13 | KIRC | FEMALE | 70 | - | - | - | - | - | - | - | - | - |
| TCGA-CW-6087 | TCGA-CW-6087-11A-01R-1671-13 | KIRC | MALE | 61 | - | - | - | - | - | - | - | - | - |
| TCGA-CW-6088 | TCGA-CW-6088-11A-01R-1671-13 | KIRC | MALE | 60 | - | - | - | - | - | - | - | - | - |
| TCGA-CZ-5469 | TCGA-CZ-5469-11A-01R-1502-13 | KIRC | MALE | 41 | - | - | - | - | - | - | - | - | - |
| TCGA-CZ-4863 | TCGA-CZ-4863-11A-01R-1502-13 | KIRC | FEMALE | 51 | - | - | - | - | - | - | - | - | - |
| TCGA-CZ-5461 | TCGA-CZ-5461-11A-01R-1502-13 | KIRC | MALE | 52 | - | - | - | - | - | - | - | - | - |
| TCGA-CZ-5987 | TCGA-CZ-5987-11A-01R-1671-13 | KIRC | MALE | 60 | - | - | - | - | - | - | - | - | - |
| TCGA-CJ-5678 | TCGA-CJ-5678-11A-01R-1540-13 | KIRC | MALE | 62 | - | - | - | - | - | - | - | - | - |
| TCGA-B0-5701 | TCGA-B0-5701-11A-01R-1540-13 | KIRC | MALE | 65 | - | - | - | - | - | - | - | - | - |
| TCGA-B0-5690 | TCGA-B0-5690-11A-01R-1540-13 | KIRC | FEMALE | 53 | - | - | - | - | - | - | - | - | - |
| TCGA-A3-3387 | TCGA-A3-3387-11A-01R-1540-13 | KIRC | MALE | 49 | - | - | - | - | - | - | - | - | - |
| TCGA-CZ-5468 | TCGA-CZ-5468-11A-01R-1502-13 | KIRC | MALE | 84 | - | - | - | - | - | - | - | - | - |
| TCGA-CZ-5462 | TCGA-CZ-5462-11A-01R-1502-13 | KIRC | MALE | 83 | - | - | - | - | - | - | - | - | - |
