# Supplementary material for: Multi-omics analysis reveals epithelial-mesenchymal transition-related gene FOXM1 as a novel prognostic biomarker in clear cell renal carcinoma
Source: Aging (Albany NY). 2019 Nov 19;11(22):10316–37. doi: 10.18632/aging.102459 (PMC6914426; doi:10.18632/aging.102459)
Supplement: Supplementary Table 6 [file aging-11-102459-s001..docx]

| Supplementary Table 6. The patients and corresponding clinical information of GEO expression dataset (n=440) | | | | | | | | |
| --- | --- | --- | --- | --- | --- | --- | --- | --- |
| GSM_id | Platform | Series | Group | AnalyteGroup | Gender | Clin_PrimarySite | Stage | Grade |
| GSM347652 | GPL570 | GSE13818 | Cancer | KIRC | Unknown | Unknown | Unknown | Unknown |
| GSM1009107 | GPL570 | GSE41137 | Cancer | KIRC | Unknown | VHL Clear Cell Renal Cell Carcinoma | Unknown | Unknown |
| GSM198786 | GPL570 | GSE8050 | Cancer | KIRC | Unknown | Unknown | Unknown | Unknown |
| GSM315857 | GPL570 | GSE12606 | Cancer | KIRC | MALE | Unknown | Unknown | Unknown |
| GSM1009179 | GPL570 | GSE41137 | Cancer | KIRC | Unknown | VHL Clear Cell Renal Cell Carcinoma | Unknown | Unknown |
| GSM559577 | GPL570 | GSE22541 | Cancer | KIRC | MALE | primary clear-cell renal cell carcinoma | Stage I | G2 |
| GSM1009185 | GPL570 | GSE41137 | Cancer | KIRC | Unknown | VHL Clear Cell Renal Cell Carcinoma | Unknown | Unknown |
| GSM1009195 | GPL570 | GSE41137 | Cancer | KIRC | Unknown | VHL Clear Cell Renal Cell Carcinoma | Unknown | Unknown |
| GSM1009124 | GPL570 | GSE41137 | Cancer | KIRC | Unknown | VHL Clear Cell Renal Cell Carcinoma | Unknown | Unknown |
| GSM1009122 | GPL570 | GSE41137 | Cancer | KIRC | Unknown | VHL Clear Cell Renal Cell Carcinoma | Unknown | Unknown |
| GSM1009140 | GPL570 | GSE41137 | Cancer | KIRC | Unknown | VHL Clear Cell Renal Cell Carcinoma | Unknown | Unknown |
| GSM1009087 | GPL570 | GSE41137 | Cancer | KIRC | Unknown | VHL Clear Cell Renal Cell Carcinoma | Unknown | Unknown |
| GSM1009074 | GPL570 | GSE41137 | Cancer | KIRC | Unknown | VHL Clear Cell Renal Cell Carcinoma | Unknown | Unknown |
| GSM1009084 | GPL570 | GSE41137 | Cancer | KIRC | Unknown | VHL Clear Cell Renal Cell Carcinoma | Unknown | Unknown |
| GSM559580 | GPL570 | GSE22541 | Cancer | KIRC | FEMALE | primary clear-cell renal cell carcinoma | Stage II | G2 |
| GSM1009064 | GPL570 | GSE41137 | Cancer | KIRC | Unknown | VHL Clear Cell Renal Cell Carcinoma | Unknown | Unknown |
| GSM1009189 | GPL570 | GSE41137 | Cancer | KIRC | Unknown | VHL Clear Cell Renal Cell Carcinoma | Unknown | Unknown |
| GSM1009151 | GPL570 | GSE41137 | Cancer | KIRC | Unknown | VHL Clear Cell Renal Cell Carcinoma | Unknown | Unknown |
| GSM1009121 | GPL570 | GSE41137 | Cancer | KIRC | Unknown | VHL Clear Cell Renal Cell Carcinoma | Unknown | Unknown |
| GSM1009194 | GPL570 | GSE41137 | Cancer | KIRC | Unknown | VHL Clear Cell Renal Cell Carcinoma | Unknown | Unknown |
| GSM1009102 | GPL570 | GSE41137 | Cancer | KIRC | Unknown | VHL Clear Cell Renal Cell Carcinoma | Unknown | Unknown |
| GSM1009142 | GPL570 | GSE41137 | Cancer | KIRC | Unknown | VHL Clear Cell Renal Cell Carcinoma | Unknown | Unknown |
| GSM559579 | GPL570 | GSE22541 | Cancer | KIRC | FEMALE | primary clear-cell renal cell carcinoma | Stage I | G2 |
| GSM1009166 | GPL570 | GSE41137 | Cancer | KIRC | Unknown | VHL Clear Cell Renal Cell Carcinoma | Unknown | Unknown |
| GSM1009184 | GPL570 | GSE41137 | Cancer | KIRC | Unknown | VHL Clear Cell Renal Cell Carcinoma | Unknown | Unknown |
| GSM559591 | GPL570 | GSE22541 | Cancer | KIRC | MALE | primary clear-cell renal cell carcinoma | Stage I | G2 |
| GSM1009076 | GPL570 | GSE41137 | Cancer | KIRC | Unknown | VHL Clear Cell Renal Cell Carcinoma | Unknown | Unknown |
| GSM347673 | GPL570 | GSE13818 | Cancer | KIRC | Unknown | Unknown | Unknown | Unknown |
| GSM1009182 | GPL570 | GSE41137 | Cancer | KIRC | Unknown | VHL Clear Cell Renal Cell Carcinoma | Unknown | Unknown |
| GSM1009092 | GPL570 | GSE41137 | Cancer | KIRC | Unknown | VHL Clear Cell Renal Cell Carcinoma | Unknown | Unknown |
| GSM1009178 | GPL570 | GSE41137 | Cancer | KIRC | Unknown | VHL Clear Cell Renal Cell Carcinoma | Unknown | Unknown |
| GSM559587 | GPL570 | GSE22541 | Cancer | KIRC | FEMALE | primary clear-cell renal cell carcinoma | Stage I | G3 |
| GSM1009155 | GPL570 | GSE41137 | Cancer | KIRC | Unknown | VHL Clear Cell Renal Cell Carcinoma | Unknown | Unknown |
| GSM1009080 | GPL570 | GSE41137 | Cancer | KIRC | Unknown | VHL Clear Cell Renal Cell Carcinoma | Unknown | Unknown |
| GSM1009079 | GPL570 | GSE41137 | Cancer | KIRC | Unknown | VHL Clear Cell Renal Cell Carcinoma | Unknown | Unknown |
| GSM198784 | GPL570 | GSE8050 | Cancer | KIRC | Unknown | Unknown | Unknown | Unknown |
| GSM1009091 | GPL570 | GSE41137 | Cancer | KIRC | Unknown | VHL Clear Cell Renal Cell Carcinoma | Unknown | Unknown |
| GSM1009141 | GPL570 | GSE41137 | Cancer | KIRC | Unknown | VHL Clear Cell Renal Cell Carcinoma | Unknown | Unknown |
| GSM559578 | GPL570 | GSE22541 | Cancer | KIRC | MALE | primary clear-cell renal cell carcinoma | Stage I | G2 |
| GSM1009126 | GPL570 | GSE41137 | Cancer | KIRC | Unknown | VHL Clear Cell Renal Cell Carcinoma | Unknown | Unknown |
| GSM825420 | GPL570 | GSE33371 | Cancer | KIRC | MALE | Unknown | Stage II | Unknown |
| GSM825427 | GPL570 | GSE33371 | Cancer | KIRC | FEMALE | Unknown | Stage III | Unknown |
| GSM559595 | GPL570 | GSE22541 | Cancer | KIRC | FEMALE | primary clear-cell renal cell carcinoma | Stage II | G2 |
| GSM559581 | GPL570 | GSE22541 | Cancer | KIRC | FEMALE | primary clear-cell renal cell carcinoma | Stage II | G2 |
| GSM1009127 | GPL570 | GSE41137 | Cancer | KIRC | Unknown | VHL Clear Cell Renal Cell Carcinoma | Unknown | Unknown |
| GSM1009071 | GPL570 | GSE41137 | Cancer | KIRC | Unknown | VHL Clear Cell Renal Cell Carcinoma | Unknown | Unknown |
| GSM1009131 | GPL570 | GSE41137 | Cancer | KIRC | Unknown | VHL Clear Cell Renal Cell Carcinoma | Unknown | Unknown |
| GSM825430 | GPL570 | GSE33371 | Cancer | KIRC | FEMALE | Unknown | Stage I | Unknown |
| GSM1009136 | GPL570 | GSE41137 | Cancer | KIRC | Unknown | VHL Clear Cell Renal Cell Carcinoma | Unknown | Unknown |
| GSM1009093 | GPL570 | GSE41137 | Cancer | KIRC | Unknown | VHL Clear Cell Renal Cell Carcinoma | Unknown | Unknown |
| GSM1009096 | GPL570 | GSE41137 | Cancer | KIRC | Unknown | VHL Clear Cell Renal Cell Carcinoma | Unknown | Unknown |
| GSM347679 | GPL570 | GSE13818 | Cancer | KIRC | Unknown | Unknown | Unknown | Unknown |
| GSM1009146 | GPL570 | GSE41137 | Cancer | KIRC | Unknown | VHL Clear Cell Renal Cell Carcinoma | Unknown | Unknown |
| GSM1009067 | GPL570 | GSE41137 | Cancer | KIRC | Unknown | VHL Clear Cell Renal Cell Carcinoma | Unknown | Unknown |
| GSM1009170 | GPL570 | GSE41137 | Cancer | Unknown | Unknown | Unknown | Unknown | Unknown |
| GSM1009191 | GPL570 | GSE41137 | Cancer | KIRC | Unknown | VHL Clear Cell Renal Cell Carcinoma | Unknown | Unknown |
| GSM559585 | GPL570 | GSE22541 | Cancer | KIRC | FEMALE | primary clear-cell renal cell carcinoma | Stage II | G2 |
| GSM1009082 | GPL570 | GSE41137 | Cancer | KIRC | Unknown | VHL Clear Cell Renal Cell Carcinoma | Unknown | Unknown |
| GSM1009105 | GPL570 | GSE41137 | Cancer | KIRC | Unknown | VHL Clear Cell Renal Cell Carcinoma | Unknown | Unknown |
| GSM1009150 | GPL570 | GSE41137 | Cancer | KIRC | Unknown | VHL Clear Cell Renal Cell Carcinoma | Unknown | Unknown |
| GSM1009075 | GPL570 | GSE41137 | Cancer | KIRC | Unknown | VHL Clear Cell Renal Cell Carcinoma | Unknown | Unknown |
| GSM1009068 | GPL570 | GSE41137 | Cancer | KIRC | Unknown | VHL Clear Cell Renal Cell Carcinoma | Unknown | Unknown |
| GSM347681 | GPL570 | GSE13818 | Cancer | KIRC | Unknown | Unknown | Unknown | Unknown |
| GSM347646 | GPL570 | GSE13818 | Cancer | KIRC | Unknown | Unknown | Unknown | Unknown |
| GSM1009120 | GPL570 | GSE41137 | Cancer | KIRC | Unknown | VHL Clear Cell Renal Cell Carcinoma | Unknown | Unknown |
| GSM1009198 | GPL570 | GSE41137 | Cancer | KIRC | Unknown | VHL Clear Cell Renal Cell Carcinoma | Unknown | Unknown |
| GSM1009162 | GPL570 | GSE41137 | Cancer | KIRC | Unknown | VHL Clear Cell Renal Cell Carcinoma | Unknown | Unknown |
| GSM347658 | GPL570 | GSE13818 | Cancer | KIRC | Unknown | Unknown | Unknown | Unknown |
| GSM1009099 | GPL570 | GSE41137 | Cancer | KIRC | Unknown | VHL Clear Cell Renal Cell Carcinoma | Unknown | Unknown |
| GSM1009101 | GPL570 | GSE41137 | Cancer | KIRC | Unknown | VHL Clear Cell Renal Cell Carcinoma | Unknown | Unknown |
| GSM1009103 | GPL570 | GSE41137 | Cancer | KIRC | Unknown | VHL Clear Cell Renal Cell Carcinoma | Unknown | Unknown |
| GSM1009193 | GPL570 | GSE41137 | Cancer | KIRC | Unknown | VHL Clear Cell Renal Cell Carcinoma | Unknown | Unknown |
| GSM1009100 | GPL570 | GSE41137 | Cancer | KIRC | Unknown | VHL Clear Cell Renal Cell Carcinoma | Unknown | Unknown |
| GSM1009186 | GPL570 | GSE41137 | Cancer | KIRC | Unknown | VHL Clear Cell Renal Cell Carcinoma | Unknown | Unknown |
| GSM1009114 | GPL570 | GSE41137 | Cancer | KIRC | Unknown | VHL Clear Cell Renal Cell Carcinoma | Unknown | Unknown |
| GSM1009109 | GPL570 | GSE41137 | Cancer | KIRC | Unknown | VHL Clear Cell Renal Cell Carcinoma | Unknown | Unknown |
| GSM1009159 | GPL570 | GSE41137 | Cancer | KIRC | Unknown | VHL Clear Cell Renal Cell Carcinoma | Unknown | Unknown |
| GSM1009073 | GPL570 | GSE41137 | Cancer | KIRC | Unknown | VHL Clear Cell Renal Cell Carcinoma | Unknown | Unknown |
| GSM347669 | GPL570 | GSE13818 | Cancer | KIRC | Unknown | Unknown | Unknown | Unknown |
| GSM1009174 | GPL570 | GSE41137 | Cancer | KIRC | Unknown | VHL Clear Cell Renal Cell Carcinoma | Unknown | Unknown |
| GSM1009098 | GPL570 | GSE41137 | Cancer | KIRC | Unknown | VHL Clear Cell Renal Cell Carcinoma | Unknown | Unknown |
| GSM1009157 | GPL570 | GSE41137 | Cancer | KIRC | Unknown | VHL Clear Cell Renal Cell Carcinoma | Unknown | Unknown |
| GSM1009144 | GPL570 | GSE41137 | Cancer | KIRC | Unknown | VHL Clear Cell Renal Cell Carcinoma | Unknown | Unknown |
| GSM1009176 | GPL570 | GSE41137 | Cancer | KIRC | Unknown | VHL Clear Cell Renal Cell Carcinoma | Unknown | Unknown |
| GSM1009192 | GPL570 | GSE41137 | Cancer | KIRC | Unknown | VHL Clear Cell Renal Cell Carcinoma | Unknown | Unknown |
| GSM559592 | GPL570 | GSE22541 | Cancer | KIRC | MALE | primary clear-cell renal cell carcinoma | Stage III | G3 |
| GSM1009123 | GPL570 | GSE41137 | Cancer | KIRC | Unknown | VHL Clear Cell Renal Cell Carcinoma | Unknown | Unknown |
| GSM347676 | GPL570 | GSE13818 | Cancer | KIRC | Unknown | Unknown | Unknown | Unknown |
| GSM1009161 | GPL570 | GSE41137 | Cancer | KIRC | Unknown | VHL Clear Cell Renal Cell Carcinoma | Unknown | Unknown |
| GSM1009199 | GPL570 | GSE41137 | Cancer | KIRC | Unknown | VHL Clear Cell Renal Cell Carcinoma | Unknown | Unknown |
| GSM1009078 | GPL570 | GSE41137 | Cancer | KIRC | Unknown | VHL Clear Cell Renal Cell Carcinoma | Unknown | Unknown |
| GSM347665 | GPL570 | GSE13818 | Cancer | KIRC | Unknown | Unknown | Unknown | Unknown |
| GSM1009130 | GPL570 | GSE41137 | Cancer | KIRC | Unknown | VHL Clear Cell Renal Cell Carcinoma | Unknown | Unknown |
| GSM1009066 | GPL570 | GSE41137 | Cancer | KIRC | Unknown | VHL Clear Cell Renal Cell Carcinoma | Unknown | Unknown |
| GSM559589 | GPL570 | GSE22541 | Cancer | KIRC | MALE | primary clear-cell renal cell carcinoma | Stage I | G2 |
| GSM1009177 | GPL570 | GSE41137 | Cancer | KIRC | Unknown | VHL Clear Cell Renal Cell Carcinoma | Unknown | Unknown |
| GSM347649 | GPL570 | GSE13818 | Cancer | KIRC | Unknown | Unknown | Unknown | Unknown |
| GSM1009165 | GPL570 | GSE41137 | Cancer | KIRC | Unknown | VHL Clear Cell Renal Cell Carcinoma | Unknown | Unknown |
| GSM559596 | GPL570 | GSE22541 | Cancer | KIRC | FEMALE | primary clear-cell renal cell carcinoma | Stage II | G2 |
| GSM1009113 | GPL570 | GSE41137 | Cancer | KIRC | Unknown | VHL Clear Cell Renal Cell Carcinoma | Unknown | Unknown |
| GSM1009171 | GPL570 | GSE41137 | Cancer | KIRC | Unknown | VHL Clear Cell Renal Cell Carcinoma | Unknown | Unknown |
| GSM1009116 | GPL570 | GSE41137 | Cancer | KIRC | Unknown | VHL Clear Cell Renal Cell Carcinoma | Unknown | Unknown |
| GSM1009135 | GPL570 | GSE41137 | Cancer | KIRC | Unknown | VHL Clear Cell Renal Cell Carcinoma | Unknown | Unknown |
| GSM1009188 | GPL570 | GSE41137 | Cancer | KIRC | Unknown | VHL Clear Cell Renal Cell Carcinoma | Unknown | Unknown |
| GSM1009133 | GPL570 | GSE41137 | Cancer | KIRC | Unknown | VHL Clear Cell Renal Cell Carcinoma | Unknown | Unknown |
| GSM1009088 | GPL570 | GSE41137 | Cancer | KIRC | Unknown | VHL Clear Cell Renal Cell Carcinoma | Unknown | Unknown |
| GSM1009156 | GPL570 | GSE41137 | Cancer | KIRC | Unknown | VHL Clear Cell Renal Cell Carcinoma | Unknown | Unknown |
| GSM1009169 | GPL570 | GSE41137 | Cancer | KIRC | Unknown | VHL Clear Cell Renal Cell Carcinoma | Unknown | Unknown |
| GSM1009129 | GPL570 | GSE41137 | Cancer | KIRC | Unknown | VHL Clear Cell Renal Cell Carcinoma | Unknown | Unknown |
| GSM1009062 | GPL570 | GSE41137 | Cancer | KIRC | Unknown | VHL Clear Cell Renal Cell Carcinoma | Unknown | Unknown |
| GSM347647 | GPL570 | GSE13818 | Cancer | KIRC | Unknown | Unknown | Unknown | Unknown |
| GSM347642 | GPL570 | GSE13818 | Cancer | KIRC | Unknown | Unknown | Unknown | Unknown |
| GSM1009138 | GPL570 | GSE41137 | Cancer | KIRC | Unknown | VHL Clear Cell Renal Cell Carcinoma | Unknown | Unknown |
| GSM1009180 | GPL570 | GSE41137 | Cancer | KIRC | Unknown | VHL Clear Cell Renal Cell Carcinoma | Unknown | Unknown |
| GSM1009148 | GPL570 | GSE41137 | Cancer | KIRC | Unknown | VHL Clear Cell Renal Cell Carcinoma | Unknown | Unknown |
| GSM1009137 | GPL570 | GSE41137 | Cancer | KIRC | Unknown | VHL Clear Cell Renal Cell Carcinoma | Unknown | Unknown |
| GSM1009097 | GPL570 | GSE41137 | Cancer | KIRC | Unknown | VHL Clear Cell Renal Cell Carcinoma | Unknown | Unknown |
| GSM1009106 | GPL570 | GSE41137 | Cancer | KIRC | Unknown | VHL Clear Cell Renal Cell Carcinoma | Unknown | Unknown |
| GSM559600 | GPL570 | GSE22541 | Cancer | KIRC | MALE | primary clear-cell renal cell carcinoma | Stage III | G3 |
| GSM2041410 | GPL570 | GSE76948 | Cancer | KIRC | Unknown | Unknown | Unknown | Unknown |
| GSM1009147 | GPL570 | GSE41137 | Cancer | KIRC | Unknown | VHL Clear Cell Renal Cell Carcinoma | Unknown | Unknown |
| GSM825429 | GPL570 | GSE33371 | Cancer | KIRC | FEMALE | Unknown | Stage IV | Unknown |
| GSM1009085 | GPL570 | GSE41137 | Cancer | KIRC | Unknown | VHL Clear Cell Renal Cell Carcinoma | Unknown | Unknown |
| GSM1009077 | GPL570 | GSE41137 | Cancer | KIRC | Unknown | VHL Clear Cell Renal Cell Carcinoma | Unknown | Unknown |
| GSM347657 | GPL570 | GSE13818 | Cancer | KIRC | Unknown | Unknown | Unknown | Unknown |
| GSM1009094 | GPL570 | GSE41137 | Cancer | KIRC | Unknown | VHL Clear Cell Renal Cell Carcinoma | Unknown | Unknown |
| GSM825422 | GPL570 | GSE33371 | Cancer | KIRC | MALE | Unknown | Stage II | Unknown |
| GSM1009117 | GPL570 | GSE41137 | Cancer | KIRC | Unknown | VHL Clear Cell Renal Cell Carcinoma | Unknown | Unknown |
| GSM347650 | GPL570 | GSE13818 | Cancer | KIRC | Unknown | Unknown | Unknown | Unknown |
| GSM1009163 | GPL570 | GSE41137 | Cancer | KIRC | Unknown | VHL Clear Cell Renal Cell Carcinoma | Unknown | Unknown |
| GSM347678 | GPL570 | GSE13818 | Cancer | KIRC | Unknown | Unknown | Unknown | Unknown |
| GSM1009095 | GPL570 | GSE41137 | Cancer | KIRC | Unknown | VHL Clear Cell Renal Cell Carcinoma | Unknown | Unknown |
| GSM1009172 | GPL570 | GSE41137 | Cancer | KIRC | Unknown | VHL Clear Cell Renal Cell Carcinoma | Unknown | Unknown |
| GSM315863 | GPL570 | GSE12606 | Cancer | KIRC | MALE | Unknown | Unknown | Unknown |
| GSM1009108 | GPL570 | GSE41137 | Cancer | KIRC | Unknown | VHL Clear Cell Renal Cell Carcinoma | Unknown | Unknown |
| GSM1009110 | GPL570 | GSE41137 | Cancer | KIRC | Unknown | VHL Clear Cell Renal Cell Carcinoma | Unknown | Unknown |
| GSM1009175 | GPL570 | GSE41137 | Cancer | KIRC | Unknown | VHL Clear Cell Renal Cell Carcinoma | Unknown | Unknown |
| GSM1009086 | GPL570 | GSE41137 | Cancer | KIRC | Unknown | VHL Clear Cell Renal Cell Carcinoma | Unknown | Unknown |
| GSM1009115 | GPL570 | GSE41137 | Cancer | KIRC | Unknown | VHL Clear Cell Renal Cell Carcinoma | Unknown | Unknown |
| GSM1009069 | GPL570 | GSE41137 | Cancer | Unknown | Unknown | Unknown | Unknown | Unknown |
| GSM1009160 | GPL570 | GSE41137 | Cancer | KIRC | Unknown | VHL Clear Cell Renal Cell Carcinoma | Unknown | Unknown |
| GSM461784 | GPL570 | GSE18549 | Cancer | KIRC | Unknown | Unknown | Unknown | Unknown |
| GSM1009201 | GPL570 | GSE41137 | Cancer | KIRC | Unknown | VHL Clear Cell Renal Cell Carcinoma | Unknown | Unknown |
| GSM347644 | GPL570 | GSE13818 | Cancer | KIRC | Unknown | Unknown | Unknown | Unknown |
| GSM1009111 | GPL570 | GSE41137 | Cancer | KIRC | Unknown | VHL Clear Cell Renal Cell Carcinoma | Unknown | Unknown |
| GSM1009200 | GPL570 | GSE41137 | Cancer | KIRC | Unknown | VHL Clear Cell Renal Cell Carcinoma | Unknown | Unknown |
| GSM1009168 | GPL570 | GSE41137 | Cancer | KIRC | Unknown | VHL Clear Cell Renal Cell Carcinoma | Unknown | Unknown |
| GSM2041411 | GPL570 | GSE76948 | Cancer | KIRC | Unknown | Unknown | Unknown | Unknown |
| GSM559598 | GPL570 | GSE22541 | Cancer | KIRC | MALE | primary clear-cell renal cell carcinoma | Stage III | G2 |
| GSM1009181 | GPL570 | GSE41137 | Cancer | KIRC | Unknown | VHL Clear Cell Renal Cell Carcinoma | Unknown | Unknown |
| GSM1009118 | GPL570 | GSE41137 | Cancer | KIRC | Unknown | VHL Clear Cell Renal Cell Carcinoma | Unknown | Unknown |
| GSM1009112 | GPL570 | GSE41137 | Cancer | KIRC | Unknown | VHL Clear Cell Renal Cell Carcinoma | Unknown | Unknown |
| GSM1009158 | GPL570 | GSE41137 | Cancer | KIRC | Unknown | VHL Clear Cell Renal Cell Carcinoma | Unknown | Unknown |
| GSM1009072 | GPL570 | GSE41137 | Cancer | KIRC | Unknown | VHL Clear Cell Renal Cell Carcinoma | Unknown | Unknown |
| GSM347667 | GPL570 | GSE13818 | Cancer | KIRC | Unknown | Unknown | Unknown | Unknown |
| GSM559597 | GPL570 | GSE22541 | Cancer | KIRC | MALE | primary clear-cell renal cell carcinoma | Stage II | G2 |
| GSM1009153 | GPL570 | GSE41137 | Cancer | KIRC | Unknown | VHL Clear Cell Renal Cell Carcinoma | Unknown | Unknown |
| GSM1009119 | GPL570 | GSE41137 | Cancer | KIRC | Unknown | VHL Clear Cell Renal Cell Carcinoma | Unknown | Unknown |
| GSM1009183 | GPL570 | GSE41137 | Cancer | KIRC | Unknown | VHL Clear Cell Renal Cell Carcinoma | Unknown | Unknown |
| GSM559599 | GPL570 | GSE22541 | Cancer | KIRC | MALE | primary clear-cell renal cell carcinoma | Stage III | G2 |
| GSM1009070 | GPL570 | GSE41137 | Cancer | KIRC | Unknown | VHL Clear Cell Renal Cell Carcinoma | Unknown | Unknown |
| GSM1009154 | GPL570 | GSE41137 | Cancer | KIRC | Unknown | VHL Clear Cell Renal Cell Carcinoma | Unknown | Unknown |
| GSM347670 | GPL570 | GSE13818 | Cancer | KIRC | Unknown | Unknown | Unknown | Unknown |
| GSM1009190 | GPL570 | GSE41137 | Cancer | KIRC | Unknown | VHL Clear Cell Renal Cell Carcinoma | Unknown | Unknown |
| GSM1009128 | GPL570 | GSE41137 | Cancer | KIRC | Unknown | VHL Clear Cell Renal Cell Carcinoma | Unknown | Unknown |
| GSM347654 | GPL570 | GSE13818 | Cancer | KIRC | Unknown | Unknown | Unknown | Unknown |
| GSM1009145 | GPL570 | GSE41137 | Cancer | KIRC | Unknown | VHL Clear Cell Renal Cell Carcinoma | Unknown | Unknown |
| GSM347637 | GPL570 | GSE13818 | Cancer | KIRC | Unknown | Unknown | Unknown | Unknown |
| GSM1009164 | GPL570 | GSE41137 | Cancer | KIRC | Unknown | VHL Clear Cell Renal Cell Carcinoma | Unknown | Unknown |
| GSM1009196 | GPL570 | GSE41137 | Cancer | KIRC | Unknown | VHL Clear Cell Renal Cell Carcinoma | Unknown | Unknown |
| GSM1009063 | GPL570 | GSE41137 | Cancer | KIRC | Unknown | VHL Clear Cell Renal Cell Carcinoma | Unknown | Unknown |
| GSM1009083 | GPL570 | GSE41137 | Cancer | KIRC | Unknown | VHL Clear Cell Renal Cell Carcinoma | Unknown | Unknown |
| GSM347699 | GPL570 | GSE13818 | Cancer | KIRC | Unknown | Unknown | Unknown | Unknown |
| GSM1009143 | GPL570 | GSE41137 | Cancer | KIRC | Unknown | VHL Clear Cell Renal Cell Carcinoma | Unknown | Unknown |
| GSM1009197 | GPL570 | GSE41137 | Cancer | KIRC | Unknown | VHL Clear Cell Renal Cell Carcinoma | Unknown | Unknown |
| GSM1009089 | GPL570 | GSE41137 | Cancer | KIRC | Unknown | VHL Clear Cell Renal Cell Carcinoma | Unknown | Unknown |
| GSM1009167 | GPL570 | GSE41137 | Cancer | KIRC | Unknown | VHL Clear Cell Renal Cell Carcinoma | Unknown | Unknown |
| GSM347684 | GPL570 | GSE13818 | Cancer | KIRC | Unknown | Unknown | Unknown | Unknown |
| GSM1009090 | GPL570 | GSE41137 | Cancer | KIRC | Unknown | VHL Clear Cell Renal Cell Carcinoma | Unknown | Unknown |
| GSM315860 | GPL570 | GSE12606 | Cancer | KIRC | MALE | Unknown | Unknown | Unknown |
| GSM559583 | GPL570 | GSE22541 | Cancer | KIRC | MALE | primary clear-cell renal cell carcinoma | Stage I | G3 |
| GSM1009134 | GPL570 | GSE41137 | Cancer | KIRC | Unknown | VHL Clear Cell Renal Cell Carcinoma | Unknown | Unknown |
| GSM1009149 | GPL570 | GSE41137 | Cancer | KIRC | Unknown | VHL Clear Cell Renal Cell Carcinoma | Unknown | Unknown |
| GSM1009132 | GPL570 | GSE41137 | Cancer | KIRC | Unknown | VHL Clear Cell Renal Cell Carcinoma | Unknown | Unknown |
| GSM1009187 | GPL570 | GSE41137 | Cancer | KIRC | Unknown | VHL Clear Cell Renal Cell Carcinoma | Unknown | Unknown |
| GSM347700 | GPL570 | GSE13818 | Cancer | KIRC | Unknown | Unknown | Unknown | Unknown |
| GSM1009104 | GPL570 | GSE41137 | Cancer | KIRC | Unknown | VHL Clear Cell Renal Cell Carcinoma | Unknown | Unknown |
| GSM1009125 | GPL570 | GSE41137 | Cancer | KIRC | Unknown | VHL Clear Cell Renal Cell Carcinoma | Unknown | Unknown |
| GSM1009065 | GPL570 | GSE41137 | Cancer | KIRC | Unknown | VHL Clear Cell Renal Cell Carcinoma | Unknown | Unknown |
| GSM281424 | GPL570 | GSE11166 | Normal | KIRC | Unknown | Unknown | Unknown | Unknown |
| GSM1964045 | GPL570 | GSE75693 | Normal | KIRC | Unknown | kidney | Unknown | Unknown |
| GSM849090 | GPL570 | GSE34437 | Normal | KIRC | Unknown | Unknown | Unknown | Unknown |
| GSM1618388 | GPL570 | GSE66270/GSE66272 | Normal | KIRC | MALE | adj.normal | Unknown | Unknown |
| GSM1964095 | GPL570 | GSE75693 | Normal | KIRC | Unknown | kidney | Unknown | Unknown |
| GSM517901 | GPL570 | GSE20615 | Normal | KIRC | Unknown | Unknown | Unknown | Unknown |
| GSM1618416 | GPL570 | GSE66271/GSE66272 | Normal | KIRC | MALE | adj.normal | Unknown | Unknown |
| GSM281407 | GPL570 | GSE11166 | Normal | KIRC | Unknown | Unknown | Unknown | Unknown |
| GSM176410 | GPL570 | GSE7307 | Normal | KIRC | MALE | adrenal_gland_cortex | Unknown | Unknown |
| GSM1964050 | GPL570 | GSE75693 | Normal | KIRC | Unknown | kidney | Unknown | Unknown |
| GSM849092 | GPL570 | GSE34437 | Normal | KIRC | Unknown | Unknown | Unknown | Unknown |
| GSM1134873 | GPL570 | GSE46699 | Normal | KIRC | Unknown | normal | Unknown | Unknown |
| GSM1618410 | GPL570 | GSE66270/GSE66272 | Normal | KIRC | MALE | adj.normal | Unknown | Unknown |
| GSM1964096 | GPL570 | GSE75693 | Normal | KIRC | Unknown | kidney | Unknown | Unknown |
| GSM477794 | GPL570 | GSE19249 | Normal | KIRC | Unknown | kidney | Unknown | Unknown |
| GSM1618432 | GPL570 | GSE66271/GSE66272 | Normal | KIRC | FEMALE | adj.normal | Unknown | Unknown |
| GSM1039977 | GPL570 | GSE20615 | Normal | KIRC | Unknown | Unknown | Unknown | Unknown |
| GSM1964101 | GPL570 | GSE75693 | Normal | KIRC | Unknown | kidney | Unknown | Unknown |
| GSM281311 | GPL570 | GSE11151 | Normal | KIRC | Unknown | Unknown | Unknown | Unknown |
| GSM849069 | GPL570 | GSE34437 | Normal | KIRC | Unknown | Unknown | Unknown | Unknown |
| GSM198785 | GPL570 | GSE8050 | Normal | KIRC | Unknown | Unknown | Unknown | Unknown |
| GSM1964075 | GPL570 | GSE75693 | Normal | KIRC | Unknown | kidney | Unknown | Unknown |
| GSM1964100 | GPL570 | GSE75693 | Normal | KIRC | Unknown | kidney | Unknown | Unknown |
| GSM281400 | GPL570 | GSE11166 | Normal | KIRC | Unknown | Unknown | Unknown | Unknown |
| GSM1964081 | GPL570 | GSE75693 | Normal | KIRC | Unknown | kidney | Unknown | Unknown |
| GSM281402 | GPL570 | GSE11166 | Normal | KIRC | Unknown | Unknown | Unknown | Unknown |
| GSM1964064 | GPL570 | GSE75693 | Normal | KIRC | Unknown | kidney | Unknown | Unknown |
| GSM1964079 | GPL570 | GSE75693 | Normal | KIRC | Unknown | kidney | Unknown | Unknown |
| GSM1964091 | GPL570 | GSE75693 | Normal | KIRC | Unknown | kidney | Unknown | Unknown |
| GSM477789 | GPL570 | GSE19249 | Normal | KIRC | Unknown | kidney | Unknown | Unknown |
| GSM281413 | GPL570 | GSE11166 | Normal | KIRC | Unknown | Unknown | Unknown | Unknown |
| GSM849108 | GPL570 | GSE34437 | Normal | KIRC | Unknown | Unknown | Unknown | Unknown |
| GSM1964094 | GPL570 | GSE75693 | Normal | KIRC | Unknown | kidney | Unknown | Unknown |
| GSM693937 | GPL570 | GSE28050/GSE28053 | Normal | KIRC | Unknown | Unknown | Unknown | Unknown |
| GSM477795 | GPL570 | GSE19249 | Normal | KIRC | Unknown | kidney | Unknown | Unknown |
| GSM1964071 | GPL570 | GSE75693 | Normal | KIRC | Unknown | kidney | Unknown | Unknown |
| GSM1618420 | GPL570 | GSE66271/GSE66272 | Normal | KIRC | FEMALE | adj.normal | Unknown | Unknown |
| GSM849065 | GPL570 | GSE34437 | Normal | KIRC | Unknown | Unknown | Unknown | Unknown |
| GSM849050 | GPL570 | GSE34437 | Normal | KIRC | Unknown | Unknown | Unknown | Unknown |
| GSM1964085 | GPL570 | GSE75693 | Normal | KIRC | Unknown | kidney | Unknown | Unknown |
| GSM1618430 | GPL570 | GSE66271/GSE66272 | Normal | KIRC | MALE | adj.normal | Unknown | Unknown |
| GSM1618398 | GPL570 | GSE66270/GSE66272 | Normal | KIRC | FEMALE | adj.normal | Unknown | Unknown |
| GSM849079 | GPL570 | GSE34437 | Normal | KIRC | Unknown | Unknown | Unknown | Unknown |
| GSM281405 | GPL570 | GSE11166 | Normal | KIRC | Unknown | Unknown | Unknown | Unknown |
| GSM849067 | GPL570 | GSE34437 | Normal | KIRC | Unknown | Unknown | Unknown | Unknown |
| GSM849089 | GPL570 | GSE34437 | Normal | KIRC | Unknown | Unknown | Unknown | Unknown |
| GSM1134877 | GPL570 | GSE46699 | Normal | KIRC | Unknown | normal | Unknown | Unknown |
| GSM1618434 | GPL570 | GSE66271/GSE66272 | Normal | KIRC | FEMALE | adj.normal | Unknown | Unknown |
| GSM281316 | GPL570 | GSE11151 | Normal | KIRC | Unknown | Unknown | Unknown | Unknown |
| GSM46955 | GPL570 | GSE2109 | Normal | BRCA | Unknown | Unknown | Unknown | Unknown |
| GSM176322 | GPL570 | GSE7307 | Normal | KIRC | MALE | kidney_cortex | Unknown | Unknown |
| GSM281351 | GPL570 | GSE11166 | Normal | KIRC | Unknown | Unknown | Unknown | Unknown |
| GSM849068 | GPL570 | GSE34437 | Normal | KIRC | Unknown | Unknown | Unknown | Unknown |
| GSM281346 | GPL570 | GSE11166 | Normal | KIRC | Unknown | Unknown | Unknown | Unknown |
| GSM281314 | GPL570 | GSE11151 | Normal | KIRC | Unknown | Unknown | Unknown | Unknown |
| GSM849049 | GPL570 | GSE34437 | Normal | KIRC | Unknown | Unknown | Unknown | Unknown |
| GSM2143914 | GPL570 | GSE81156 | Normal | KIRC | FEMALE | Unknown | Unknown | Unknown |
| GSM1304421 | GPL570 | GSE46699 | Normal | KIRC | Unknown | normal | Unknown | Unknown |
| GSM1618406 | GPL570 | GSE66270/GSE66272 | Normal | KIRC | MALE | adj.normal | Unknown | Unknown |
| GSM849075 | GPL570 | GSE34437 | Normal | KIRC | Unknown | Unknown | Unknown | Unknown |
| GSM315855 | GPL570 | GSE12606 | Normal | KIRC | Unknown | Unknown | Unknown | Unknown |
| GSM1618418 | GPL570 | GSE66271/GSE66272 | Normal | KIRC | MALE | adj.normal | Unknown | Unknown |
| GSM1964063 | GPL570 | GSE75693 | Normal | KIRC | Unknown | kidney | Unknown | Unknown |
| GSM1964084 | GPL570 | GSE75693 | Normal | KIRC | Unknown | kidney | Unknown | Unknown |
| GSM1618390 | GPL570 | GSE66270/GSE66272 | Normal | KIRC | MALE | adj.normal | Unknown | Unknown |
| GSM281422 | GPL570 | GSE11166 | Normal | KIRC | Unknown | Unknown | Unknown | Unknown |
| GSM176424 | GPL570 | GSE7307 | Normal | KIRC | MALE | kidney_medulla | Unknown | Unknown |
| GSM517903 | GPL570 | GSE20615 | Normal | KIRC | Unknown | Unknown | Unknown | Unknown |
| GSM1964066 | GPL570 | GSE75693 | Normal | KIRC | Unknown | kidney | Unknown | Unknown |
| GSM176409 | GPL570 | GSE7307 | Normal | KIRC | FEMALE | adrenal_gland_cortex | Unknown | Unknown |
| GSM1618414 | GPL570 | GSE66270/GSE66272 | Normal | KIRC | MALE | adj.normal | Unknown | Unknown |
| GSM1964098 | GPL570 | GSE75693 | Normal | KIRC | Unknown | kidney | Unknown | Unknown |
| GSM493253 | GPL570 | GSE19750/GSE19776 | Normal | KIRC | Unknown | Unknown | Unknown | Unknown |
| GSM1964086 | GPL570 | GSE75693 | Normal | KIRC | Unknown | kidney | Unknown | Unknown |
| GSM281352 | GPL570 | GSE11166 | Normal | KIRC | Unknown | Unknown | Unknown | Unknown |
| GSM849071 | GPL570 | GSE34437 | Normal | KIRC | Unknown | Unknown | Unknown | Unknown |
| GSM1964072 | GPL570 | GSE75693 | Normal | KIRC | Unknown | kidney | Unknown | Unknown |
| GSM1618396 | GPL570 | GSE66270/GSE66272 | Normal | KIRC | MALE | adj.normal | Unknown | Unknown |
| GSM849062 | GPL570 | GSE34437 | Normal | KIRC | Unknown | Unknown | Unknown | Unknown |
| GSM46955 | GPL570 | GSE2109 | Normal | BRCA | Unknown | Unknown | Unknown | Unknown |
| GSM1964058 | GPL570 | GSE75693 | Normal | KIRC | Unknown | kidney | Unknown | Unknown |
| GSM1964074 | GPL570 | GSE75693 | Normal | KIRC | Unknown | kidney | Unknown | Unknown |
| GSM1134861 | GPL570 | GSE46699 | Normal | KIRC | Unknown | normal | Unknown | Unknown |
| GSM1304423 | GPL570 | GSE46699 | Normal | KIRC | Unknown | normal | Unknown | Unknown |
| GSM477797 | GPL570 | GSE19249 | Normal | KIRC | Unknown | kidney | Unknown | Unknown |
| GSM1964073 | GPL570 | GSE75693 | Normal | KIRC | Unknown | kidney | Unknown | Unknown |
| GSM849072 | GPL570 | GSE34437 | Normal | KIRC | Unknown | Unknown | Unknown | Unknown |
| GSM1964044 | GPL570 | GSE75693 | Normal | KIRC | Unknown | kidney | Unknown | Unknown |
| GSM1618408 | GPL570 | GSE66270/GSE66272 | Normal | KIRC | MALE | adj.normal | Unknown | Unknown |
| GSM625518 | GPL570 | GSE25471 | Normal | KIRC | Unknown | Unknown | Unknown | Unknown |
| GSM1964051 | GPL570 | GSE75693 | Normal | KIRC | Unknown | kidney | Unknown | Unknown |
| GSM849091 | GPL570 | GSE34437 | Normal | KIRC | Unknown | Unknown | Unknown | Unknown |
| GSM1618402 | GPL570 | GSE66270/GSE66272 | Normal | KIRC | MALE | adj.normal | Unknown | Unknown |
| GSM315856 | GPL570 | GSE12606 | Normal | KIRC | MALE | Unknown | Unknown | Unknown |
| GSM849076 | GPL570 | GSE34437 | Normal | KIRC | Unknown | Unknown | Unknown | Unknown |
| GSM1134879 | GPL570 | GSE46699 | Normal | KIRC | Unknown | normal | Unknown | Unknown |
| GSM849099 | GPL570 | GSE34437 | Normal | KIRC | Unknown | Unknown | Unknown | Unknown |
| GSM849053 | GPL570 | GSE34437 | Normal | KIRC | Unknown | Unknown | Unknown | Unknown |
| GSM176324 | GPL570 | GSE7307 | Normal | KIRC | MALE | kidney_cortex | Unknown | Unknown |
| GSM281276 | GPL570 | GSE11166 | Normal | KIRC | Unknown | Unknown | Unknown | Unknown |
| GSM281356 | GPL570 | GSE11166 | Normal | KIRC | Unknown | Unknown | Unknown | Unknown |
| GSM1964046 | GPL570 | GSE75693 | Normal | KIRC | Unknown | kidney | Unknown | Unknown |
| GSM1964070 | GPL570 | GSE75693 | Normal | KIRC | Unknown | kidney | Unknown | Unknown |
| GSM849057 | GPL570 | GSE34437 | Normal | KIRC | Unknown | Unknown | Unknown | Unknown |
| GSM281416 | GPL570 | GSE11166 | Normal | KIRC | Unknown | Unknown | Unknown | Unknown |
| GSM477786 | GPL570 | GSE19249 | Normal | KIRC | Unknown | kidney | Unknown | Unknown |
| GSM281410 | GPL570 | GSE11166 | Normal | KIRC | Unknown | Unknown | Unknown | Unknown |
| GSM1964088 | GPL570 | GSE75693 | Normal | KIRC | Unknown | kidney | Unknown | Unknown |
| GSM1618438 | GPL570 | GSE66271/GSE66272 | Normal | KIRC | FEMALE | adj.normal | Unknown | Unknown |
| GSM493251 | GPL570 | GSE19750/GSE19776 | Normal | KIRC | Unknown | Unknown | Unknown | Unknown |
| GSM849086 | GPL570 | GSE34437 | Normal | KIRC | Unknown | Unknown | Unknown | Unknown |
| GSM518606 | GPL570 | GSE20677 | Normal | KIRC | Unknown | Unknown | Unknown | Unknown |
| GSM1964103 | GPL570 | GSE75693 | Normal | KIRC | Unknown | kidney | Unknown | Unknown |
| GSM849088 | GPL570 | GSE34437 | Normal | KIRC | Unknown | Unknown | Unknown | Unknown |
| GSM1964057 | GPL570 | GSE75693 | Normal | KIRC | Unknown | kidney | Unknown | Unknown |
| GSM1964111 | GPL570 | GSE75693 | Normal | KIRC | Unknown | kidney | Unknown | Unknown |
| GSM849078 | GPL570 | GSE34437 | Normal | KIRC | Unknown | Unknown | Unknown | Unknown |
| GSM1964069 | GPL570 | GSE75693 | Normal | KIRC | Unknown | kidney | Unknown | Unknown |
| GSM849087 | GPL570 | GSE34437 | Normal | KIRC | Unknown | Unknown | Unknown | Unknown |
| GSM1964108 | GPL570 | GSE75693 | Normal | KIRC | Unknown | kidney | Unknown | Unknown |
| GSM849073 | GPL570 | GSE34437 | Normal | Unknown | Unknown | Unknown | Unknown | Unknown |
| GSM1964082 | GPL570 | GSE75693 | Normal | KIRC | Unknown | kidney | Unknown | Unknown |
| GSM281357 | GPL570 | GSE11166 | Normal | KIRC | Unknown | Unknown | Unknown | Unknown |
| GSM849082 | GPL570 | GSE34437 | Normal | KIRC | Unknown | Unknown | Unknown | Unknown |
| GSM493252 | GPL570 | GSE19750/GSE19776 | Normal | KIRC | Unknown | Unknown | Unknown | Unknown |
| GSM1039978 | GPL570 | GSE20615 | Normal | KIRC | Unknown | Unknown | Unknown | Unknown |
| GSM1964039 | GPL570 | GSE75693 | Normal | KIRC | Unknown | kidney | Unknown | Unknown |
| GSM1134857 | GPL570 | GSE46699 | Normal | KIRC | Unknown | normal | Unknown | Unknown |
| GSM1964080 | GPL570 | GSE75693 | Normal | KIRC | Unknown | kidney | Unknown | Unknown |
| GSM849106 | GPL570 | GSE34437 | Normal | KIRC | Unknown | Unknown | Unknown | Unknown |
| GSM693939 | GPL570 | GSE28050/GSE28053 | Normal | KIRC | Unknown | Unknown | Unknown | Unknown |
| GSM1964104 | GPL570 | GSE75693 | Normal | KIRC | Unknown | kidney | Unknown | Unknown |
| GSM315862 | GPL570 | GSE12606 | Normal | KIRC | MALE | Unknown | Unknown | Unknown |
| GSM849085 | GPL570 | GSE34437 | Normal | KIRC | Unknown | Unknown | Unknown | Unknown |
| GSM849063 | GPL570 | GSE34437 | Normal | KIRC | Unknown | Unknown | Unknown | Unknown |
| GSM681102 | GPL570 | GSE27556 | Normal | KIRC | Unknown | normal kidney tissue | Unknown | Unknown |
| GSM1618426 | GPL570 | GSE66271/GSE66272 | Normal | KIRC | MALE | adj.normal | Unknown | Unknown |
| GSM849058 | GPL570 | GSE34437 | Normal | KIRC | Unknown | Unknown | Unknown | Unknown |
| GSM46955 | GPL570 | GSE2109 | Normal | BRCA | Unknown | Unknown | Unknown | Unknown |
| GSM1964089 | GPL570 | GSE75693 | Normal | KIRC | Unknown | kidney | Unknown | Unknown |
| GSM176321 | GPL570 | GSE7307 | Normal | KIRC | MALE | kidney_cortex | Unknown | Unknown |
| GSM1134851 | GPL570 | GSE46699 | Normal | KIRC | Unknown | normal | Unknown | Unknown |
| GSM1039979 | GPL570 | GSE20615 | Normal | KIRC | Unknown | Unknown | Unknown | Unknown |
| GSM849051 | GPL570 | GSE34437 | Normal | KIRC | Unknown | Unknown | Unknown | Unknown |
| GSM1964042 | GPL570 | GSE75693 | Normal | KIRC | Unknown | kidney | Unknown | Unknown |
| GSM517902 | GPL570 | GSE20615 | Normal | KIRC | Unknown | Unknown | Unknown | Unknown |
| GSM1964056 | GPL570 | GSE75693 | Normal | KIRC | Unknown | kidney | Unknown | Unknown |
| GSM1964087 | GPL570 | GSE75693 | Normal | KIRC | Unknown | kidney | Unknown | Unknown |
| GSM1134853 | GPL570 | GSE46699 | Normal | KIRC | Unknown | normal | Unknown | Unknown |
| GSM477791 | GPL570 | GSE19249 | Normal | KIRC | Unknown | kidney | Unknown | Unknown |
| GSM1618436 | GPL570 | GSE66271/GSE66272 | Normal | KIRC | FEMALE | adj.normal | Unknown | Unknown |
| GSM176427 | GPL570 | GSE7307 | Normal | KIRC | MALE | kidney_medulla | Unknown | Unknown |
| GSM1964062 | GPL570 | GSE75693 | Normal | KIRC | Unknown | kidney | Unknown | Unknown |
| GSM1964041 | GPL570 | GSE75693 | Normal | KIRC | Unknown | kidney | Unknown | Unknown |
| GSM1964067 | GPL570 | GSE75693 | Normal | KIRC | Unknown | kidney | Unknown | Unknown |
| GSM1964112 | GPL570 | GSE75693 | Normal | KIRC | Unknown | kidney | Unknown | Unknown |
| GSM1964059 | GPL570 | GSE75693 | Normal | KIRC | Unknown | kidney | Unknown | Unknown |
| GSM693950 | GPL570 | GSE28050/GSE28053 | Normal | Unknown | Unknown | Unknown | Unknown | Unknown |
| GSM281414 | GPL570 | GSE11166 | Normal | KIRC | Unknown | Unknown | Unknown | Unknown |
| GSM1134859 | GPL570 | GSE46699 | Normal | KIRC | Unknown | normal | Unknown | Unknown |
| GSM693938 | GPL570 | GSE28050/GSE28053 | Normal | KIRC | Unknown | Unknown | Unknown | Unknown |
| GSM1964049 | GPL570 | GSE75693 | Normal | KIRC | Unknown | kidney | Unknown | Unknown |
| GSM281403 | GPL570 | GSE11166 | Normal | KIRC | Unknown | Unknown | Unknown | Unknown |
| GSM281084 | GPL570 | GSE11166 | Normal | KIRC | Unknown | Unknown | Unknown | Unknown |
| GSM1134871 | GPL570 | GSE46699 | Normal | KIRC | Unknown | normal | Unknown | Unknown |
| GSM176408 | GPL570 | GSE7307 | Normal | KIRC | MALE | adrenal_gland_cortex | Unknown | Unknown |
| GSM849054 | GPL570 | GSE34437 | Normal | KIRC | Unknown | Unknown | Unknown | Unknown |
| GSM849074 | GPL570 | GSE34437 | Normal | KIRC | Unknown | Unknown | Unknown | Unknown |
| GSM281421 | GPL570 | GSE11166 | Normal | KIRC | Unknown | Unknown | Unknown | Unknown |
| GSM1964117 | GPL570 | GSE75693 | Normal | KIRC | Unknown | kidney | Unknown | Unknown |
| GSM1134869 | GPL570 | GSE46699 | Normal | KIRC | Unknown | normal | Unknown | Unknown |
| GSM1964092 | GPL570 | GSE75693 | Normal | KIRC | Unknown | kidney | Unknown | Unknown |
| GSM1964060 | GPL570 | GSE75693 | Normal | KIRC | Unknown | kidney | Unknown | Unknown |
| GSM1618424 | GPL570 | GSE66271/GSE66272 | Normal | KIRC | MALE | adj.normal | Unknown | Unknown |
| GSM281277 | GPL570 | GSE11166 | Normal | KIRC | Unknown | Unknown | Unknown | Unknown |
| GSM1964109 | GPL570 | GSE75693 | Normal | KIRC | Unknown | kidney | Unknown | Unknown |
| GSM1134865 | GPL570 | GSE46699 | Normal | KIRC | Unknown | normal | Unknown | Unknown |
| GSM849083 | GPL570 | GSE34437 | Normal | KIRC | Unknown | Unknown | Unknown | Unknown |
| GSM1964068 | GPL570 | GSE75693 | Normal | KIRC | Unknown | kidney | Unknown | Unknown |
| GSM1964054 | GPL570 | GSE75693 | Normal | KIRC | Unknown | kidney | Unknown | Unknown |
| GSM281406 | GPL570 | GSE11166 | Normal | KIRC | Unknown | Unknown | Unknown | Unknown |
| GSM693951 | GPL570 | GSE28050/GSE28053 | Normal | KIRC | Unknown | Unknown | Unknown | Unknown |
| GSM477787 | GPL570 | GSE19249 | Normal | KIRC | Unknown | kidney | Unknown | Unknown |
| GSM1618428 | GPL570 | GSE66271/GSE66272 | Normal | KIRC | MALE | adj.normal | Unknown | Unknown |
| GSM198783 | GPL570 | GSE8050 | Normal | KIRC | Unknown | Unknown | Unknown | Unknown |
| GSM1134849 | GPL570 | GSE46699 | Normal | KIRC | Unknown | normal | Unknown | Unknown |
| GSM849107 | GPL570 | GSE34437 | Normal | KIRC | Unknown | Unknown | Unknown | Unknown |
| GSM1618394 | GPL570 | GSE66270/GSE66272 | Normal | KIRC | MALE | adj.normal | Unknown | Unknown |
| GSM849059 | GPL570 | GSE34437 | Normal | KIRC | Unknown | Unknown | Unknown | Unknown |
| GSM1964110 | GPL570 | GSE75693 | Normal | KIRC | Unknown | kidney | Unknown | Unknown |
| GSM1964078 | GPL570 | GSE75693 | Normal | KIRC | Unknown | kidney | Unknown | Unknown |
| GSM1964102 | GPL570 | GSE75693 | Normal | KIRC | Unknown | kidney | Unknown | Unknown |
| GSM1964097 | GPL570 | GSE75693 | Normal | KIRC | Unknown | kidney | Unknown | Unknown |
| GSM849077 | GPL570 | GSE34437 | Normal | KIRC | Unknown | Unknown | Unknown | Unknown |
| GSM849070 | GPL570 | GSE34437 | Normal | KIRC | Unknown | Unknown | Unknown | Unknown |
| GSM1618392 | GPL570 | GSE66270/GSE66272 | Normal | KIRC | MALE | adj.normal | Unknown | Unknown |
| GSM1618422 | GPL570 | GSE66271/GSE66272 | Normal | KIRC | MALE | adj.normal | Unknown | Unknown |
| GSM849066 | GPL570 | GSE34437 | Normal | KIRC | Unknown | Unknown | Unknown | Unknown |
| GSM1964077 | GPL570 | GSE75693 | Normal | KIRC | Unknown | kidney | Unknown | Unknown |
| GSM1964061 | GPL570 | GSE75693 | Normal | KIRC | Unknown | kidney | Unknown | Unknown |
| GSM315859 | GPL570 | GSE12606 | Normal | KIRC | MALE | Unknown | Unknown | Unknown |
| GSM176426 | GPL570 | GSE7307 | Normal | KIRC | FEMALE | kidney_medulla | Unknown | Unknown |
| GSM849081 | GPL570 | GSE34437 | Normal | KIRC | Unknown | Unknown | Unknown | Unknown |
| GSM493254 | GPL570 | GSE19750/GSE19776 | Normal | KIRC | Unknown | Unknown | Unknown | Unknown |
| GSM1964076 | GPL570 | GSE75693 | Normal | KIRC | Unknown | kidney | Unknown | Unknown |
| GSM693936 | GPL570 | GSE28050/GSE28053 | Normal | KIRC | Unknown | Unknown | Unknown | Unknown |
| GSM175911 | GPL570 | GSE7307 | Normal | KIRC | MIX | kidney | Unknown | Unknown |
| GSM1964114 | GPL570 | GSE75693 | Normal | KIRC | Unknown | kidney | Unknown | Unknown |
| GSM176407 | GPL570 | GSE7307 | Normal | KIRC | MALE | adrenal_gland_cortex | Unknown | Unknown |
| GSM1134875 | GPL570 | GSE46699 | Normal | KIRC | Unknown | normal | Unknown | Unknown |
| GSM1618440 | GPL570 | GSE66271/GSE66272 | Normal | KIRC | MALE | adj.normal | Unknown | Unknown |
| GSM1964116 | GPL570 | GSE75693 | Normal | KIRC | Unknown | kidney | Unknown | Unknown |
| GSM849056 | GPL570 | GSE34437 | Normal | KIRC | Unknown | Unknown | Unknown | Unknown |
| GSM849105 | GPL570 | GSE34437 | Normal | KIRC | Unknown | Unknown | Unknown | Unknown |
| GSM281312 | GPL570 | GSE11151 | Normal | KIRC | Unknown | Unknown | Unknown | Unknown |
| GSM281347 | GPL570 | GSE11166 | Normal | KIRC | Unknown | Unknown | Unknown | Unknown |
| GSM634979 | GPL570 | GSE25861 | Normal | KIRC | MALE | Unknown | Unknown | Unknown |
| GSM1134863 | GPL570 | GSE46699 | Normal | KIRC | Unknown | normal | Unknown | Unknown |
| GSM1964040 | GPL570 | GSE75693 | Normal | KIRC | Unknown | kidney | Unknown | Unknown |
| GSM1618404 | GPL570 | GSE66270/GSE66272 | Normal | KIRC | MALE | adj.normal | Unknown | Unknown |
| GSM1134855 | GPL570 | GSE46699 | Normal | KIRC | Unknown | normal | Unknown | Unknown |
| GSM1964107 | GPL570 | GSE75693 | Normal | KIRC | Unknown | kidney | Unknown | Unknown |
| GSM281408 | GPL570 | GSE11166 | Normal | KIRC | Unknown | Unknown | Unknown | Unknown |
| GSM1964055 | GPL570 | GSE75693 | Normal | KIRC | Unknown | kidney | Unknown | Unknown |
| GSM849064 | GPL570 | GSE34437 | Normal | KIRC | Unknown | Unknown | Unknown | Unknown |
| GSM1964090 | GPL570 | GSE75693 | Normal | KIRC | Unknown | kidney | Unknown | Unknown |
| GSM849055 | GPL570 | GSE34437 | Normal | KIRC | Unknown | Unknown | Unknown | Unknown |
| GSM477799 | GPL570 | GSE19249 | Normal | KIRC | Unknown | kidney | Unknown | Unknown |
| GSM849052 | GPL570 | GSE34437 | Normal | KIRC | Unknown | Unknown | Unknown | Unknown |
| GSM2143913 | GPL570 | GSE81156 | Normal | KIRC | FEMALE | Unknown | Unknown | Unknown |
| GSM281417 | GPL570 | GSE11166 | Normal | KIRC | Unknown | Unknown | Unknown | Unknown |
| GSM1964099 | GPL570 | GSE75693 | Normal | KIRC | Unknown | kidney | Unknown | Unknown |
| GSM176425 | GPL570 | GSE7307 | Normal | KIRC | MALE | kidney_medulla | Unknown | Unknown |
| GSM849061 | GPL570 | GSE34437 | Normal | KIRC | Unknown | Unknown | Unknown | Unknown |
| GSM281399 | GPL570 | GSE11166 | Normal | KIRC | Unknown | Unknown | Unknown | Unknown |
| GSM281259 | GPL570 | GSE11166 | Normal | KIRC | Unknown | Unknown | Unknown | Unknown |
| GSM1964113 | GPL570 | GSE75693 | Normal | KIRC | Unknown | kidney | Unknown | Unknown |
| GSM849084 | GPL570 | GSE34437 | Normal | KIRC | Unknown | Unknown | Unknown | Unknown |
| GSM1134867 | GPL570 | GSE46699 | Normal | KIRC | Unknown | normal | Unknown | Unknown |
| GSM281315 | GPL570 | GSE11151 | Normal | KIRC | Unknown | Unknown | Unknown | Unknown |
| GSM1618412 | GPL570 | GSE66270/GSE66272 | Normal | KIRC | FEMALE | adj.normal | Unknown | Unknown |
| GSM1618400 | GPL570 | GSE66270/GSE66272 | Normal | KIRC | FEMALE | adj.normal | Unknown | Unknown |
| GSM1964048 | GPL570 | GSE75693 | Normal | KIRC | Unknown | kidney | Unknown | Unknown |
| GSM1964083 | GPL570 | GSE75693 | Normal | KIRC | Unknown | kidney | Unknown | Unknown |
| GSM1964043 | GPL570 | GSE75693 | Normal | KIRC | Unknown | kidney | Unknown | Unknown |
| GSM176323 | GPL570 | GSE7307 | Normal | KIRC | FEMALE | kidney_cortex | Unknown | Unknown |
| GSM849080 | GPL570 | GSE34437 | Normal | KIRC | Unknown | Unknown | Unknown | Unknown |
| GSM281420 | GPL570 | GSE11166 | Normal | KIRC | Unknown | Unknown | Unknown | Unknown |
| GSM1964115 | GPL570 | GSE75693 | Normal | KIRC | Unknown | kidney | Unknown | Unknown |
| GSM849060 | GPL570 | GSE34437 | Normal | KIRC | Unknown | Unknown | Unknown | Unknown |
| GSM1964065 | GPL570 | GSE75693 | Normal | KIRC | Unknown | kidney | Unknown | Unknown |
