# Supplementary material for: Multi-omics analysis reveals epithelial-mesenchymal transition-related gene FOXM1 as a novel prognostic biomarker in clear cell renal carcinoma
Source: Aging (Albany NY). 2019 Nov 19;11(22):10316–37. doi: 10.18632/aging.102459 (PMC6914426; doi:10.18632/aging.102459)
Supplement: Supplementary Table 7 [file aging-11-102459-s006..docx]

| Supplementary Table 7. The patients and corresponding clinical information of DNA methylation dataset (n=577) | | | | | | | | | | | | |
| --- | --- | --- | --- | --- | --- | --- | --- | --- | --- | --- | --- | --- |
| SampleID | Group | Gender | Age | AJCC_PT | AJCC_PN | AJCC_PM | Stage | Grade | OS_status | OS_months | DFS_status | DFS_months |
| TCGA-6D-AA2E-01A-11D-A36Y-05 | Cancer | FEMALE | 68 | T1b | NX | MX | Stage I | G2 | LIVING | 11.89 | DiseaseFree | 11.89 |
| TCGA-A3-3357-01A-02D-1418-05 | Cancer | MALE | 62 | T2 | N0 | M0 | Stage II | G3 | LIVING | 88.3 | DiseaseFree | 88.3 |
| TCGA-A3-3358-01A-01D-1536-05 | Cancer | FEMALE | 57 | T1a | N0 | M0 | Stage I | G2 | LIVING | 42.94 | DiseaseFree | 42.94 |
| TCGA-A3-3367-01A-02D-1418-05 | Cancer | MALE | 72 | T1b | N0 | M0 | Stage I | G3 | LIVING | 74.57 | DiseaseFree | 74.57 |
| TCGA-A3-3370-01A-02D-1418-05 | Cancer | FEMALE | 48 | T1b | N0 | M0 | Stage I | G2 | LIVING | 74.7 | DiseaseFree | 74.7 |
| TCGA-A3-3373-01A-02D-1418-05 | Cancer | FEMALE | 54 | T1b | N0 | M0 | Stage I | G3 | LIVING | 53.25 | DiseaseFree | 53.25 |
| TCGA-A3-3376-01A-02D-1418-05 | Cancer | MALE | 51 | T1a | N0 | M0 | Stage I | G2 | DECEASED | 55.72 | Unknown | Unknown |
| TCGA-A3-3385-01A-02D-1418-05 | Cancer | FEMALE | 46 | T1a | N0 | M0 | Stage I | G2 | LIVING | 65.47 | DiseaseFree | 65.47 |
| TCGA-A3-3387-01A-01D-1536-05 | Cancer | MALE | 49 | T1a | N0 | M0 | Stage I | G2 | LIVING | 20.27 | DiseaseFree | 20.27 |
| TCGA-A3-A6NI-01A-11D-A33L-05 | Cancer | MALE | 47 | T1a | NX | MX | Stage I | G3 | LIVING | 33.44 | DiseaseFree | 33.44 |
| TCGA-A3-A6NJ-01A-12D-A33L-05 | Cancer | FEMALE | 57 | T1a | NX | MX | Stage I | G1 | LIVING | 15.37 | DiseaseFree | 15.37 |
| TCGA-A3-A6NL-01A-11D-A33L-05 | Cancer | FEMALE | 49 | T1b | NX | MX | Stage I | G2 | LIVING | 22.63 | DiseaseFree | 22.63 |
| TCGA-A3-A6NN-01A-12D-A33L-05 | Cancer | MALE | 78 | T1a | NX | MX | Stage I | G2 | LIVING | 0.1 | DiseaseFree | 0.1 |
| TCGA-A3-A8CQ-01A-11D-A36Y-05 | Cancer | FEMALE | 59 | T1a | NX | MX | Stage I | G2 | LIVING | 0.1 | DiseaseFree | 0.1 |
| TCGA-A3-A8OU-01A-11D-A36Y-05 | Cancer | FEMALE | 74 | T1a | NX | MX | Stage I | G1 | LIVING | 0 | DiseaseFree | 0 |
| TCGA-A3-A8OV-01A-11D-A36Y-05 | Cancer | MALE | 75 | T1a | NX | MX | Stage I | G2 | LIVING | 11.17 | DiseaseFree | 11.17 |
| TCGA-A3-A8OW-01A-11D-A36Y-05 | Cancer | MALE | 37 | T3a | NX | MX | Stage III | G2 | LIVING | 10.61 | DiseaseFree | 10.61 |
| TCGA-A3-A8OX-01A-11D-A36Y-05 | Cancer | FEMALE | 65 | T1a | NX | MX | Stage I | G3 | LIVING | 0 | DiseaseFree | 0 |
| TCGA-AK-3425-01A-02D-1275-05 | Cancer | MALE | 68 | T1 | N0 | M0 | Stage I | G2 | LIVING | 109.82 | DiseaseFree | 109.82 |
| TCGA-AK-3428-01A-02D-1275-05 | Cancer | MALE | 62 | T3b | N0 | M0 | Stage III | G2 | LIVING | 122.47 | DiseaseFree | 122.47 |
| TCGA-AK-3431-01A-02D-1275-05 | Cancer | FEMALE | 62 | T2 | NX | M0 | Stage II | G3 | DECEASED | 73.62 | Unknown | Unknown |
| TCGA-AK-3433-01A-02D-1275-05 | Cancer | FEMALE | 48 | T2 | N0 | M0 | Stage II | GX | LIVING | 111.99 | DiseaseFree | 111.99 |
| TCGA-AK-3434-01A-02D-1275-05 | Cancer | MALE | 72 | T1b | NX | M0 | Stage I | G2 | LIVING | 68.56 | Recurred/Progressed | 53.88 |
| TCGA-AK-3440-01A-02D-1275-05 | Cancer | MALE | 58 | T1a | NX | M0 | Stage I | G3 | LIVING | 94.12 | DiseaseFree | 94.12 |
| TCGA-AK-3445-01A-02D-1275-05 | Cancer | MALE | 69 | T3a | NX | M0 | Stage III | G3 | LIVING | 78.58 | DiseaseFree | 78.58 |
| TCGA-AK-3450-01A-02D-1275-05 | Cancer | FEMALE | 85 | T1a | N0 | M0 | Stage I | G2 | LIVING | 58.44 | DiseaseFree | 58.44 |
| TCGA-AK-3453-01A-02D-1275-05 | Cancer | FEMALE | 58 | T2 | NX | M0 | Stage II | G2 | LIVING | 83.15 | DiseaseFree | 83.15 |
| TCGA-AK-3454-01A-02D-1275-05 | Cancer | MALE | 84 | T1b | NX | M0 | Stage I | G3 | LIVING | 28.71 | DiseaseFree | 28.71 |
| TCGA-AK-3458-01A-01D-1500-05 | Cancer | MALE | 48 | T1b | NX | M0 | Stage I | G3 | LIVING | 38.37 | DiseaseFree | 38.37 |
| TCGA-AK-3460-01A-02D-1275-05 | Cancer | MALE | 58 | T1a | NX | M0 | Stage I | G2 | LIVING | 82.39 | DiseaseFree | 82.39 |
| TCGA-AK-3461-01A-02D-1275-05 | Cancer | MALE | 72 | T1a | NX | M0 | Stage I | G2 | LIVING | 72.83 | DiseaseFree | 72.83 |
| TCGA-B0-4688-01A-01D-1275-05 | Cancer | MALE | 46 | T4 | N0 | M1 | Stage IV | G4 | DECEASED | 3.32 | Unknown | Unknown |
| TCGA-B0-4690-01A-01D-1275-05 | Cancer | MALE | 65 | T4 | N0 | M1 | Stage IV | G3 | DECEASED | 1.41 | Unknown | Unknown |
| TCGA-B0-4691-01A-01D-1275-05 | Cancer | MALE | 55 | T2 | N0 | M1 | Stage IV | G3 | DECEASED | 4.57 | Unknown | Unknown |
| TCGA-B0-4693-01A-01D-1275-05 | Cancer | FEMALE | 72 | T3a | N0 | M0 | Stage III | G4 | DECEASED | 2.53 | Unknown | Unknown |
| TCGA-B0-4694-01A-01D-1275-05 | Cancer | MALE | 72 | T3b | NX | M0 | Stage III | G4 | DECEASED | 3.48 | Unknown | Unknown |
| TCGA-B0-4696-01A-01D-1275-05 | Cancer | MALE | 58 | T3a | N0 | M0 | Stage III | G3 | DECEASED | 28.45 | Recurred/Progressed | 26.08 |
| TCGA-B0-4697-01A-01D-1275-05 | Cancer | FEMALE | 46 | T3b | NX | M1 | Stage IV | G4 | DECEASED | 18.99 | Unknown | Unknown |
| TCGA-B0-4698-01A-01D-1500-05 | Cancer | MALE | 75 | T4 | NX | M0 | Stage IV | G4 | DECEASED | 1.38 | Unknown | Unknown |
| TCGA-B0-4699-01A-01D-1275-05 | Cancer | MALE | 74 | T4 | N0 | M1 | Stage IV | G4 | DECEASED | 3.61 | Unknown | Unknown |
| TCGA-B0-4700-01A-02D-1536-05 | Cancer | MALE | 60 | T4 | NX | M1 | Stage IV | G4 | DECEASED | 65.05 | Unknown | Unknown |
| TCGA-B0-4701-01A-01D-1275-05 | Cancer | FEMALE | 66 | T3a | N0 | M1 | Stage IV | G3 | DECEASED | 7.82 | Unknown | Unknown |
| TCGA-B0-4703-01A-01D-1275-05 | Cancer | MALE | 51 | T3a | N0 | M1 | Stage IV | G4 | DECEASED | 5.98 | Unknown | Unknown |
| TCGA-B0-4706-01A-01D-1500-05 | Cancer | MALE | 61 | T3a | NX | M0 | Stage III | G4 | DECEASED | 2.14 | Unknown | Unknown |
| TCGA-B0-4707-01A-01D-1275-05 | Cancer | MALE | 63 | T3a | NX | M0 | Stage III | G4 | DECEASED | 19.71 | Unknown | Unknown |
| TCGA-B0-4710-01A-01D-1500-05 | Cancer | FEMALE | 75 | T3a | N0 | M0 | Stage III | G3 | LIVING | 57.65 | DiseaseFree | 57.65 |
| TCGA-B0-4712-01A-01D-1500-05 | Cancer | MALE | 76 | T3a | NX | M1 | Stage IV | G3 | DECEASED | 43.92 | Unknown | Unknown |
| TCGA-B0-4713-01A-01D-1275-05 | Cancer | FEMALE | 76 | T3b | NX | M0 | Stage III | G2 | DECEASED | 6.64 | Unknown | Unknown |
| TCGA-B0-4714-01A-01D-1275-05 | Cancer | MALE | 81 | T3b | NX | M1 | Stage IV | G3 | DECEASED | 3.25 | Unknown | Unknown |
| TCGA-B0-4718-01A-01D-1275-05 | Cancer | MALE | 57 | T3a | NX | M0 | Stage III | G2 | LIVING | 58.41 | Recurred/Progressed | 25.23 |
| TCGA-B0-4810-01A-01D-1500-05 | Cancer | MALE | 47 | T3a | N1 | M0 | Stage III | G3 | DECEASED | 15.7 | Recurred/Progressed | 6.73 |
| TCGA-B0-4811-01A-01D-1500-05 | Cancer | MALE | 48 | T3a | N0 | M0 | Stage III | G3 | DECEASED | 46.55 | Recurred/Progressed | 15.05 |
| TCGA-B0-4813-01A-01D-1275-05 | Cancer | MALE | 68 | T3b | NX | M0 | Stage III | G3 | DECEASED | 0.59 | Unknown | Unknown |
| TCGA-B0-4814-01A-01D-1275-05 | Cancer | MALE | 58 | T4 | N0 | M1 | Stage IV | G3 | DECEASED | 5.52 | Unknown | Unknown |
| TCGA-B0-4815-01A-01D-1500-05 | Cancer | MALE | 65 | T3a | NX | M0 | Stage III | G4 | DECEASED | 52.17 | Unknown | Unknown |
| TCGA-B0-4816-01A-01D-1500-05 | Cancer | MALE | 49 | T2 | N0 | M0 | Stage II | G3 | DECEASED | 45.04 | Unknown | Unknown |
| TCGA-B0-4817-01A-01D-1275-05 | Cancer | MALE | 81 | T3c | N0 | M0 | Stage III | G3 | DECEASED | 33.48 | Unknown | Unknown |
| TCGA-B0-4818-01A-01D-1500-05 | Cancer | FEMALE | 68 | T2 | NX | M0 | Stage II | G3 | DECEASED | 16.75 | Recurred/Progressed | 4.17 |
| TCGA-B0-4819-01A-01D-1275-05 | Cancer | FEMALE | 60 | T3b | NX | M1 | Stage IV | G4 | DECEASED | 6.01 | Unknown | Unknown |
| TCGA-B0-4821-01A-01D-1500-05 | Cancer | FEMALE | 68 | T3b | N0 | M0 | Stage III | G3 | DECEASED | 40.41 | Unknown | Unknown |
| TCGA-B0-4822-01A-01D-1275-05 | Cancer | MALE | 78 | T2 | NX | M0 | Stage II | G4 | DECEASED | 36.5 | Unknown | Unknown |
| TCGA-B0-4823-01A-02D-1418-05 | Cancer | MALE | 88 | T1a | N0 | M0 | Stage I | G2 | DECEASED | 14.91 | Unknown | Unknown |
| TCGA-B0-4824-01A-01D-1275-05 | Cancer | FEMALE | 49 | T1a | N0 | M0 | Stage I | G3 | DECEASED | 54.43 | Unknown | Unknown |
| TCGA-B0-4827-01A-02D-1418-05 | Cancer | FEMALE | 77 | T3b | N0 | M0 | Stage III | G4 | DECEASED | 29.07 | Recurred/Progressed | 21.94 |
| TCGA-B0-4828-01A-01D-1275-05 | Cancer | MALE | 79 | T2 | NX | M1 | Stage IV | G3 | DECEASED | 10.09 | Unknown | Unknown |
| TCGA-B0-4841-01A-01D-1275-05 | Cancer | MALE | 63 | T2 | NX | M1 | Stage IV | G3 | DECEASED | 6.7 | Recurred/Progressed | 3.55 |
| TCGA-B0-4842-01A-02D-1418-05 | Cancer | FEMALE | 73 | T3a | N0 | M0 | Stage III | G4 | DECEASED | 56.64 | Recurred/Progressed | 8.21 |
| TCGA-B0-4843-01A-01D-1275-05 | Cancer | MALE | 57 | T3a | N0 | M0 | Stage III | G3 | DECEASED | 10.51 | Unknown | Unknown |
| TCGA-B0-4844-01A-01D-1275-05 | Cancer | MALE | 60 | T3a | NX | M1 | Stage IV | G3 | DECEASED | 10.28 | Recurred/Progressed | 2.04 |
| TCGA-B0-4845-01A-01D-1275-05 | Cancer | MALE | 70 | T3a | NX | M1 | Stage IV | G2 | DECEASED | 65.24 | Recurred/Progressed | 24.57 |
| TCGA-B0-4846-01A-01D-1275-05 | Cancer | MALE | 52 | T3a | N0 | M1 | Stage IV | G2 | DECEASED | 39.42 | Recurred/Progressed | 5.35 |
| TCGA-B0-4847-01A-01D-1275-05 | Cancer | MALE | 60 | T3a | NX | M1 | Stage IV | G3 | DECEASED | 26.05 | Unknown | Unknown |
| TCGA-B0-4848-01A-01D-1275-05 | Cancer | MALE | 54 | T3b | NX | M0 | Stage III | G3 | DECEASED | 29.01 | Recurred/Progressed | 16.23 |
| TCGA-B0-4849-01A-01D-1275-05 | Cancer | MALE | 51 | T3a | NX | M0 | Stage III | G3 | DECEASED | 2.27 | Recurred/Progressed | 0.13 |
| TCGA-B0-4852-01A-01D-1500-05 | Cancer | FEMALE | 78 | T2 | N0 | M0 | Stage II | G2 | DECEASED | 36.83 | Recurred/Progressed | 28.25 |
| TCGA-B0-4945-01A-01D-1418-05 | Cancer | FEMALE | 75 | T1a | N0 | M0 | Stage I | G2 | DECEASED | 70.47 | Unknown | Unknown |
| TCGA-B0-5080-01A-01D-1500-05 | Cancer | MALE | 63 | T3a | N0 | M1 | Stage IV | G3 | DECEASED | 11.24 | Recurred/Progressed | 3.38 |
| TCGA-B0-5083-01A-02D-1418-05 | Cancer | MALE | 63 | T1a | N0 | M0 | Stage I | G3 | DECEASED | 34.33 | Unknown | Unknown |
| TCGA-B0-5092-01A-01D-1418-05 | Cancer | FEMALE | 53 | T1a | N0 | M1 | Stage IV | G3 | DECEASED | 15.08 | Unknown | Unknown |
| TCGA-B0-5094-01A-01D-1418-05 | Cancer | MALE | 62 | T3b | N0 | M1 | Stage IV | G2 | DECEASED | 10.94 | Recurred/Progressed | 2.46 |
| TCGA-B0-5095-01A-01D-1418-05 | Cancer | MALE | 81 | T3a | N0 | M0 | Stage III | G3 | DECEASED | 8.05 | Unknown | Unknown |
| TCGA-B0-5097-01A-01D-1418-05 | Cancer | FEMALE | 59 | T3b | N0 | M0 | Stage III | G2 | LIVING | 21.85 | Recurred/Progressed | 11.79 |
| TCGA-B0-5098-01A-01D-1418-05 | Cancer | FEMALE | 53 | T1 | NX | M0 | Stage I | G3 | DECEASED | 52.04 | Unknown | Unknown |
| TCGA-B0-5099-01A-01D-1418-05 | Cancer | FEMALE | 88 | T3b | NX | M0 | Stage III | G3 | DECEASED | 15.93 | Unknown | Unknown |
| TCGA-B0-5100-01A-01D-1418-05 | Cancer | MALE | 72 | T3a | NX | M0 | Stage III | G3 | DECEASED | 62.84 | Recurred/Progressed | 46.85 |
| TCGA-B0-5102-01A-01D-1418-05 | Cancer | FEMALE | 74 | T1 | NX | M0 | Stage I | G3 | DECEASED | 90.8 | Recurred/Progressed | 89.82 |
| TCGA-B0-5104-01A-01D-1418-05 | Cancer | FEMALE | 90 | T1 | N0 | M0 | Stage I | G2 | DECEASED | 90.41 | Unknown | Unknown |
| TCGA-B0-5106-01A-01D-1418-05 | Cancer | MALE | 64 | T1a | N0 | M0 | Stage I | G2 | DECEASED | 52.5 | Unknown | Unknown |
| TCGA-B0-5107-01A-01D-1418-05 | Cancer | FEMALE | 65 | T2 | N0 | M1 | Stage IV | G4 | DECEASED | 30.45 | Recurred/Progressed | 18.04 |
| TCGA-B0-5109-01A-02D-1418-05 | Cancer | MALE | 69 | T3b | N1 | M0 | Stage III | G4 | DECEASED | 19.28 | Recurred/Progressed | 2.3 |
| TCGA-B0-5110-01A-01D-1418-05 | Cancer | FEMALE | 71 | T1a | N0 | M0 | Stage I | G2 | LIVING | 66 | DiseaseFree | 66 |
| TCGA-B0-5113-01A-01D-1418-05 | Cancer | FEMALE | 69 | T3a | N0 | M0 | Stage III | G2 | LIVING | 38.6 | DiseaseFree | 38.6 |
| TCGA-B0-5115-01A-01D-1418-05 | Cancer | MALE | 43 | T2 | N0 | M1 | Stage IV | G3 | LIVING | 52.69 | Recurred/Progressed | 2.46 |
| TCGA-B0-5116-01A-02D-1418-05 | Cancer | MALE | 52 | T3b | N0 | M0 | Stage III | G3 | LIVING | 41.85 | Recurred/Progressed | 37.09 |
| TCGA-B0-5117-01A-01D-1418-05 | Cancer | MALE | 40 | T1b | NX | M0 | Stage I | G2 | LIVING | 52.83 | DiseaseFree | 52.83 |
| TCGA-B0-5119-01A-02D-1418-05 | Cancer | FEMALE | 61 | T1b | N0 | M0 | Stage I | G2 | LIVING | 50.99 | DiseaseFree | 50.99 |
| TCGA-B0-5120-01A-01D-1418-05 | Cancer | FEMALE | 72 | T1a | N0 | M0 | Stage I | G2 | LIVING | 38.4 | DiseaseFree | 38.4 |
| TCGA-B0-5121-01A-02D-1418-05 | Cancer | MALE | 56 | T1b | N0 | M0 | Stage I | G2 | LIVING | 48.78 | DiseaseFree | 48.78 |
| TCGA-B0-5399-01A-01D-1500-05 | Cancer | MALE | 46 | T1b | N0 | M0 | Stage I | G2 | LIVING | 46.35 | DiseaseFree | 46.35 |
| TCGA-B0-5400-01A-01D-1500-05 | Cancer | FEMALE | 59 | T3b | N0 | M0 | Stage III | G4 | LIVING | 56.93 | DiseaseFree | 56.93 |
| TCGA-B0-5402-01A-01D-1500-05 | Cancer | MALE | 64 | T4 | NX | M0 | Stage IV | G4 | LIVING | 42.38 | Recurred/Progressed | 14.75 |
| TCGA-B0-5690-01A-11D-1536-05 | Cancer | FEMALE | 53 | T1b | NX | M0 | Stage I | G1 | LIVING | 111.43 | DiseaseFree | 111.43 |
| TCGA-B0-5691-01A-11D-1536-05 | Cancer | FEMALE | 66 | T1a | N0 | M0 | Stage I | G3 | LIVING | 112.71 | Recurred/Progressed | 106.77 |
| TCGA-B0-5692-01A-11D-1536-05 | Cancer | FEMALE | 66 | T3b | N0 | M0 | Stage III | G3 | LIVING | 129.57 | DiseaseFree | 129.57 |
| TCGA-B0-5693-01A-11D-1536-05 | Cancer | FEMALE | 47 | T1b | NX | M0 | Stage I | G2 | LIVING | 133.84 | DiseaseFree | 133.84 |
| TCGA-B0-5694-01A-11D-1536-05 | Cancer | MALE | 71 | T3a | N0 | M0 | Stage III | G3 | DECEASED | 15.77 | Recurred/Progressed | 9.46 |
| TCGA-B0-5695-01A-11D-1536-05 | Cancer | FEMALE | 61 | T1b | N0 | M0 | Stage I | G2 | LIVING | 70.63 | DiseaseFree | 70.63 |
| TCGA-B0-5696-01A-11D-1536-05 | Cancer | MALE | 69 | T3a | N0 | M0 | Stage III | G4 | LIVING | 85.71 | Recurred/Progressed | 56.73 |
| TCGA-B0-5697-01A-11D-1536-05 | Cancer | MALE | 50 | T1a | N0 | M0 | Stage I | G2 | LIVING | 86.4 | DiseaseFree | 86.4 |
| TCGA-B0-5698-01A-11D-1670-05 | Cancer | MALE | 77 | T1b | N0 | M0 | Stage I | G3 | LIVING | 119.28 | DiseaseFree | 119.28 |
| TCGA-B0-5699-01A-11D-1536-05 | Cancer | MALE | 53 | T1 | N0 | M0 | Stage I | G2 | LIVING | 126.18 | Recurred/Progressed | 3.09 |
| TCGA-B0-5700-01A-11D-1536-05 | Cancer | MALE | 77 | T1a | N0 | M0 | Stage I | G2 | LIVING | 58.8 | DiseaseFree | 58.8 |
| TCGA-B0-5701-01A-11D-1536-05 | Cancer | MALE | 65 | T3b | N0 | M0 | Stage III | G4 | LIVING | 80.85 | Recurred/Progressed | 7.36 |
| TCGA-B0-5703-01A-11D-1536-05 | Cancer | MALE | 73 | T1b | N0 | M0 | Stage I | G3 | LIVING | 73.78 | DiseaseFree | 73.78 |
| TCGA-B0-5706-01A-11D-1536-05 | Cancer | MALE | 45 | T2 | N0 | M0 | Stage II | G2 | LIVING | 105.29 | DiseaseFree | 105.29 |
| TCGA-B0-5707-01A-11D-1536-05 | Cancer | FEMALE | 39 | T1a | N0 | M0 | Stage I | G3 | LIVING | 123 | DiseaseFree | 123 |
| TCGA-B0-5709-01A-11D-1536-05 | Cancer | FEMALE | 62 | T3a | NX | M0 | Stage III | G3 | LIVING | 130.55 | DiseaseFree | 130.55 |
| TCGA-B0-5710-01A-11D-1670-05 | Cancer | MALE | 57 | T1b | N0 | M0 | Stage I | G2 | LIVING | 79.83 | Recurred/Progressed | 47.54 |
| TCGA-B0-5711-01A-11D-1670-05 | Cancer | MALE | 50 | T3b | NX | M0 | Stage III | G3 | LIVING | 131.04 | Recurred/Progressed | 123.72 |
| TCGA-B0-5712-01A-11D-1670-05 | Cancer | FEMALE | 68 | T2 | N0 | M1 | Stage IV | G3 | LIVING | 89.42 | Recurred/Progressed | 23.82 |
| TCGA-B0-5713-01A-11D-1670-05 | Cancer | FEMALE | 75 | T3b | N0 | M0 | Stage III | G3 | LIVING | 91.39 | DiseaseFree | 91.39 |
| TCGA-B0-5812-01A-11D-1670-05 | Cancer | MALE | 53 | T1b | NX | M0 | Stage I | G3 | LIVING | 125.95 | DiseaseFree | 125.95 |
| TCGA-B2-3924-01A-02D-A27A-05 | Cancer | MALE | 73 | T1b | NX | M0 | Stage I | G2 | LIVING | 35.87 | DiseaseFree | 35.87 |
| TCGA-B2-3924-01B-03D-A27A-05 | Cancer | MALE | 73 | T1b | NX | M0 | Stage I | G2 | LIVING | 35.87 | DiseaseFree | 35.87 |
| TCGA-B2-4101-01A-02D-1275-05 | Cancer | MALE | 52 | T2a | NX | M0 | Stage II | G3 | LIVING | 21.29 | DiseaseFree | 21.29 |
| TCGA-B2-5633-01A-01D-1536-05 | Cancer | MALE | 56 | T1b | N0 | M0 | Stage I | G2 | LIVING | 31.64 | DiseaseFree | 31.64 |
| TCGA-B2-5633-01A-01D-A27A-05 | Cancer | MALE | 56 | T1b | N0 | M0 | Stage I | G2 | LIVING | 31.64 | DiseaseFree | 31.64 |
| TCGA-B2-5633-01B-04D-A27A-05 | Cancer | MALE | 56 | T1b | N0 | M0 | Stage I | G2 | LIVING | 31.64 | DiseaseFree | 31.64 |
| TCGA-B2-5635-01A-01D-1536-05 | Cancer | MALE | 74 | T1a | NX | M0 | Stage I | G2 | LIVING | 24.8 | DiseaseFree | 24.8 |
| TCGA-B2-5635-01A-01D-A27A-05 | Cancer | MALE | 74 | T1a | NX | M0 | Stage I | G2 | LIVING | 24.8 | DiseaseFree | 24.8 |
| TCGA-B2-5635-01B-04D-A27A-05 | Cancer | MALE | 74 | T1a | NX | M0 | Stage I | G2 | LIVING | 24.8 | DiseaseFree | 24.8 |
| TCGA-B2-5639-01A-01D-1536-05 | Cancer | MALE | 46 | T3 | NX | M1 | Stage IV | G3 | DECEASED | 32.95 | Recurred/Progressed | 27.96 |
| TCGA-B2-5641-01A-01D-1536-05 | Cancer | MALE | 79 | T1a | N0 | M0 | Stage I | G3 | LIVING | 21.55 | DiseaseFree | 21.55 |
| TCGA-B2-A4SR-01A-11D-A264-05 | Cancer | MALE | 61 | T2a | NX | M0 | Stage II | Unknown | LIVING | 16.66 | Recurred/Progressed | 8.74 |
| TCGA-B4-5377-01A-01D-1500-05 | Cancer | FEMALE | 68 | T3 | N0 | M1 | Stage IV | G3 | LIVING | 11.99 | DiseaseFree | 11.99 |
| TCGA-B4-5378-01A-01D-1500-05 | Cancer | MALE | 62 | T1 | N0 | M0 | Stage I | G2 | LIVING | 5.75 | DiseaseFree | 5.75 |
| TCGA-B4-5832-01A-11D-1670-05 | Cancer | MALE | 65 | T3b | N0 | M0 | Stage III | G2 | LIVING | 5.09 | DiseaseFree | 5.09 |
| TCGA-B4-5834-01A-11D-1670-05 | Cancer | MALE | 59 | T1 | N0 | M0 | Stage I | G1 | LIVING | 1.25 | DiseaseFree | 1.25 |
| TCGA-B4-5835-01A-11D-1670-05 | Cancer | FEMALE | 64 | T1 | N0 | M0 | Stage I | G2 | LIVING | 0.53 | DiseaseFree | 0.53 |
| TCGA-B4-5836-01A-11D-1670-05 | Cancer | FEMALE | 61 | T1b | N0 | M0 | Stage I | G2 | LIVING | 4.63 | DiseaseFree | 4.63 |
| TCGA-B4-5838-01A-11D-1670-05 | Cancer | MALE | 52 | T3 | N1 | M0 | Stage III | G2 | LIVING | 5.45 | DiseaseFree | 5.45 |
| TCGA-B4-5843-01A-11D-1670-05 | Cancer | MALE | 45 | T1 | N0 | M0 | Stage I | G2 | LIVING | 0.36 | DiseaseFree | 0.36 |
| TCGA-B4-5844-01A-11D-1670-05 | Cancer | FEMALE | 61 | T2 | N0 | M0 | Stage II | G1 | LIVING | 0.23 | DiseaseFree | 0.23 |
| TCGA-B8-4146-01B-11D-1670-05 | Cancer | FEMALE | 41 | T1b | NX | M0 | Stage I | G2 | LIVING | 16.79 | DiseaseFree | 16.79 |
| TCGA-B8-4153-01B-11D-1670-05 | Cancer | MALE | 74 | T3a | NX | M0 | Stage III | G3 | LIVING | 25.03 | Recurred/Progressed | 7.33 |
| TCGA-B8-4621-01A-01D-1500-05 | Cancer | MALE | 63 | T1b | N0 | M0 | Stage I | G3 | LIVING | 25.89 | DiseaseFree | 25.89 |
| TCGA-B8-4622-01A-02D-1275-05 | Cancer | MALE | 57 | T3a | N0 | M1 | Stage IV | G3 | LIVING | 50.1 | Recurred/Progressed | 40.31 |
| TCGA-B8-5158-01A-01D-1418-05 | Cancer | MALE | 56 | T3a | N1 | M0 | Stage III | G4 | LIVING | 40.01 | DiseaseFree | 40.01 |
| TCGA-B8-5159-01A-01D-1418-05 | Cancer | FEMALE | 61 | T1a | N0 | M0 | Stage I | G3 | LIVING | 23.72 | DiseaseFree | 23.72 |
| TCGA-B8-5162-01A-01D-1418-05 | Cancer | MALE | 62 | T2a | NX | M0 | Stage II | G2 | LIVING | 1.18 | DiseaseFree | 1.18 |
| TCGA-B8-5163-01A-01D-1418-05 | Cancer | FEMALE | 63 | T3a | N0 | M0 | Stage III | G3 | LIVING | 27 | DiseaseFree | 27 |
| TCGA-B8-5164-01A-01D-1418-05 | Cancer | MALE | 65 | T3a | N0 | M0 | Stage III | G3 | LIVING | 0.85 | DiseaseFree | 0.85 |
| TCGA-B8-5165-01A-01D-1418-05 | Cancer | MALE | 43 | T1a | N0 | M0 | Stage I | G2 | LIVING | 24.21 | DiseaseFree | 24.21 |
| TCGA-B8-5545-01A-01D-1670-05 | Cancer | MALE | 42 | T1a | N0 | M0 | Stage I | G2 | LIVING | 50.1 | DiseaseFree | 50.1 |
| TCGA-B8-5546-01A-01D-1536-05 | Cancer | FEMALE | 38 | T1b | N0 | M0 | Stage I | G2 | LIVING | 16.59 | DiseaseFree | 16.59 |
| TCGA-B8-5549-01A-01D-1536-05 | Cancer | MALE | 53 | T1b | N0 | M0 | Stage I | G3 | LIVING | 6.37 | DiseaseFree | 6.37 |
| TCGA-B8-5550-01A-01D-1536-05 | Cancer | MALE | 71 | T3a | N0 | M0 | Stage III | G3 | LIVING | 48.49 | Recurred/Progressed | 22.8 |
| TCGA-B8-5551-01A-01D-1536-05 | Cancer | FEMALE | 65 | T1b | N0 | M0 | Stage I | G3 | LIVING | 0.53 | DiseaseFree | 0.53 |
| TCGA-B8-5552-01B-11D-1670-05 | Cancer | FEMALE | 41 | T1b | NX | M0 | Stage I | G2 | LIVING | 34.36 | DiseaseFree | 34.36 |
| TCGA-B8-5553-01A-01D-1536-05 | Cancer | FEMALE | 67 | T1b | N0 | M0 | Stage I | G2 | LIVING | 14.29 | DiseaseFree | 14.29 |
| TCGA-B8-A54D-01A-21D-A264-05 | Cancer | MALE | 69 | T3a | NX | MX | Stage III | G2 | LIVING | 27.27 | DiseaseFree | 27.27 |
| TCGA-B8-A54E-01A-11D-A264-05 | Cancer | FEMALE | 62 | T1b | NX | MX | Stage I | G3 | LIVING | 29.86 | DiseaseFree | 29.86 |
| TCGA-B8-A54F-01A-11D-A264-05 | Cancer | FEMALE | 49 | T1a | NX | MX | Stage I | G2 | LIVING | 17.05 | DiseaseFree | 17.05 |
| TCGA-B8-A54G-01A-11D-A264-05 | Cancer | MALE | 50 | T1a | NX | MX | Stage I | G3 | LIVING | 1.74 | DiseaseFree | 1.74 |
| TCGA-B8-A54H-01A-11D-A33L-05 | Cancer | FEMALE | 69 | T2a | N0 | MX | Stage II | G3 | LIVING | 8.41 | DiseaseFree | 8.41 |
| TCGA-B8-A54I-01A-21D-A33L-05 | Cancer | MALE | 48 | T1b | NX | MX | Stage I | G3 | LIVING | 4.93 | DiseaseFree | 4.93 |
| TCGA-B8-A54J-01A-11D-A33L-05 | Cancer | MALE | 60 | T2a | NX | MX | Stage II | G2 | LIVING | 17.35 | DiseaseFree | 17.35 |
| TCGA-B8-A7U6-01A-12D-A36Y-05 | Cancer | FEMALE | 54 | T1a | NX | Unknown | Stage I | G3 | LIVING | 16.26 | DiseaseFree | 16.26 |
| TCGA-B8-A8YJ-01A-13D-A39G-05 | Cancer | FEMALE | 60 | T1b | NX | Unknown | Stage I | G2 | LIVING | 14.16 | DiseaseFree | 14.16 |
| TCGA-BP-4760-01A-02D-1418-05 | Cancer | MALE | 69 | T1a | NX | M0 | Stage I | G2 | LIVING | 77.56 | Recurred/Progressed | 47.86 |
| TCGA-BP-4770-01A-01D-1500-05 | Cancer | FEMALE | 73 | T4 | N0 | M0 | Stage IV | G4 | DECEASED | 10.81 | Recurred/Progressed | 6.83 |
| TCGA-BP-4782-01A-02D-1418-05 | Cancer | FEMALE | 55 | T1a | NX | M0 | Stage I | G2 | LIVING | 11.63 | DiseaseFree | 11.63 |
| TCGA-BP-4801-01A-02D-1418-05 | Cancer | MALE | 57 | T1a | NX | M0 | Stage I | G2 | LIVING | 36.93 | DiseaseFree | 36.93 |
| TCGA-BP-4993-01A-02D-1418-05 | Cancer | MALE | 58 | T1a | NX | M0 | Stage I | G3 | LIVING | 5.81 | DiseaseFree | 5.81 |
| TCGA-BP-5010-01A-02D-1418-05 | Cancer | MALE | 63 | T3a | N0 | M0 | Stage III | G4 | DECEASED | 28.84 | Recurred/Progressed | 25.66 |
| TCGA-BP-5168-01A-01D-1418-05 | Cancer | MALE | 75 | T1a | NX | M0 | Stage I | G2 | DECEASED | 48.06 | Unknown | Unknown |
| TCGA-BP-5169-01A-01D-1424-05 | Cancer | MALE | 70 | T1b | N0 | M0 | Stage I | G4 | LIVING | 6.34 | DiseaseFree | 6.34 |
| TCGA-BP-5170-01A-01D-1424-05 | Cancer | MALE | 55 | T1a | NX | M0 | Stage I | G2 | LIVING | 79.24 | DiseaseFree | 79.24 |
| TCGA-BP-5173-01A-01D-1424-05 | Cancer | MALE | 75 | T1a | NX | M0 | Stage I | G2 | DECEASED | 2.04 | Unknown | Unknown |
| TCGA-BP-5174-01A-01D-1424-05 | Cancer | FEMALE | 45 | T1a | NX | M0 | Stage I | G2 | LIVING | 74.15 | DiseaseFree | 74.15 |
| TCGA-BP-5175-01A-01D-1424-05 | Cancer | MALE | 60 | T1a | NX | M0 | Stage I | G3 | LIVING | 30.62 | DiseaseFree | 30.62 |
| TCGA-BP-5176-01A-01D-1424-05 | Cancer | FEMALE | 78 | T1a | NX | M0 | Stage I | G2 | DECEASED | 52.23 | Unknown | Unknown |
| TCGA-BP-5177-01A-01D-1424-05 | Cancer | FEMALE | 46 | T1a | NX | M0 | Stage I | G3 | LIVING | 9.63 | DiseaseFree | 9.63 |
| TCGA-BP-5178-01A-01D-1424-05 | Cancer | MALE | 71 | T3a | NX | M1 | Stage IV | G4 | DECEASED | 62.81 | Recurred/Progressed | 56.14 |
| TCGA-BP-5180-01A-01D-1424-05 | Cancer | MALE | 53 | T1a | NX | M0 | Stage I | G2 | LIVING | 74.34 | DiseaseFree | 74.34 |
| TCGA-BP-5181-01A-01D-1424-05 | Cancer | FEMALE | 58 | T1b | NX | M0 | Stage I | G2 | LIVING | 49.11 | DiseaseFree | 49.11 |
| TCGA-BP-5182-01A-01D-1424-05 | Cancer | MALE | 56 | T1a | N0 | M0 | Stage I | G3 | LIVING | 38.27 | DiseaseFree | 38.27 |
| TCGA-BP-5183-01A-01D-1424-05 | Cancer | MALE | 57 | T3a | NX | M0 | Stage III | G3 | LIVING | 42.41 | Recurred/Progressed | 15.93 |
| TCGA-BP-5184-01A-01D-1424-05 | Cancer | MALE | 54 | T1a | NX | M0 | Stage I | G3 | LIVING | 37.22 | DiseaseFree | 37.22 |
| TCGA-BP-5185-01A-01D-1424-05 | Cancer | MALE | 56 | T1a | NX | M0 | Stage I | G3 | LIVING | 37.19 | Recurred/Progressed | 24.18 |
| TCGA-BP-5186-01A-01D-1424-05 | Cancer | FEMALE | 50 | T1a | N0 | M0 | Stage I | G2 | LIVING | 22.77 | DiseaseFree | 22.77 |
| TCGA-BP-5187-01A-01D-1424-05 | Cancer | MALE | 54 | T1a | NX | M0 | Stage I | G2 | LIVING | 13.34 | DiseaseFree | 13.34 |
| TCGA-BP-5189-01A-02D-1424-05 | Cancer | MALE | 60 | T1b | NX | M0 | Stage I | G4 | DECEASED | 27 | Recurred/Progressed | 25.66 |
| TCGA-BP-5191-01A-01D-1424-05 | Cancer | MALE | 79 | T3a | N0 | M0 | Stage III | G2 | LIVING | 31.77 | DiseaseFree | 31.77 |
| TCGA-BP-5192-01A-01D-1424-05 | Cancer | MALE | 59 | T1a | NX | M0 | Stage I | G2 | LIVING | 23.46 | DiseaseFree | 23.46 |
| TCGA-BP-5194-01A-02D-1424-05 | Cancer | MALE | 39 | T1a | NX | M0 | Stage I | G2 | LIVING | 13.4 | DiseaseFree | 13.4 |
| TCGA-BP-5195-01A-02D-1424-05 | Cancer | MALE | 75 | T1a | NX | M0 | Stage I | G2 | LIVING | 24.61 | DiseaseFree | 24.61 |
| TCGA-BP-5196-01A-01D-1424-05 | Cancer | MALE | 53 | T1a | NX | M0 | Stage I | G2 | LIVING | 33.44 | DiseaseFree | 33.44 |
| TCGA-BP-5198-01A-01D-1424-05 | Cancer | MALE | 72 | T3b | N0 | M0 | Stage III | G3 | LIVING | 19.81 | DiseaseFree | 19.81 |
| TCGA-BP-5199-01A-01D-1424-05 | Cancer | MALE | 58 | T2 | N0 | M0 | Stage II | G4 | LIVING | 44.51 | DiseaseFree | 44.51 |
| TCGA-BP-5200-01A-01D-1424-05 | Cancer | MALE | 44 | T2 | NX | M0 | Stage II | G4 | LIVING | 34.92 | DiseaseFree | 34.92 |
| TCGA-BP-5201-01A-01D-1424-05 | Cancer | MALE | 63 | T3b | N0 | M1 | Stage IV | G4 | LIVING | 31.24 | Recurred/Progressed | -0.62 |
| TCGA-BP-5202-01A-02D-1424-05 | Cancer | MALE | 75 | T3a | NX | M0 | Stage III | G2 | LIVING | 0.95 | DiseaseFree | 0.95 |
| TCGA-CJ-4869-01A-02D-1424-05 | Cancer | MALE | 49 | T2 | N1 | M0 | Stage III | G2 | LIVING | 83.9 | Recurred/Progressed | 57.62 |
| TCGA-CJ-4882-01A-02D-1424-05 | Cancer | MALE | 57 | T3a | NX | M0 | Stage III | G3 | LIVING | 61.86 | DiseaseFree | 61.86 |
| TCGA-CJ-4897-01A-03D-1424-05 | Cancer | FEMALE | 79 | T3a | NX | M0 | Stage III | G3 | LIVING | 109.76 | Recurred/Progressed | 46.75 |
| TCGA-CJ-4901-01A-01D-1424-05 | Cancer | MALE | 47 | T3b | NX | M0 | Stage III | G3 | LIVING | 47.63 | DiseaseFree | 47.63 |
| TCGA-CJ-4902-01A-01D-1424-05 | Cancer | MALE | 61 | T3a | NX | M0 | Stage III | G3 | LIVING | 49.93 | DiseaseFree | 49.93 |
| TCGA-CJ-4903-01A-01D-1424-05 | Cancer | MALE | 50 | T1b | NX | M0 | Stage I | G3 | LIVING | 51.25 | DiseaseFree | 51.25 |
| TCGA-CJ-4904-01A-02D-1424-05 | Cancer | FEMALE | 60 | T3a | N0 | M1 | Stage IV | G3 | LIVING | 108.48 | Recurred/Progressed | 9.13 |
| TCGA-CJ-4905-01A-02D-1424-05 | Cancer | FEMALE | 62 | T1a | NX | M0 | Stage I | G2 | LIVING | 49.15 | DiseaseFree | 49.15 |
| TCGA-CJ-4907-01A-01D-1424-05 | Cancer | MALE | 58 | T3b | NX | M0 | Stage III | G3 | LIVING | 49.24 | DiseaseFree | 49.24 |
| TCGA-CJ-4908-01A-01D-1424-05 | Cancer | MALE | 38 | T1a | NX | M0 | Stage I | G2 | LIVING | 50.3 | DiseaseFree | 50.3 |
| TCGA-CJ-4913-01A-01D-1424-05 | Cancer | FEMALE | 45 | T3a | NX | M0 | Stage III | G4 | DECEASED | 38.53 | Recurred/Progressed | 17.67 |
| TCGA-CJ-4916-01A-01D-1424-05 | Cancer | FEMALE | 69 | T3a | NX | M0 | Stage III | G3 | LIVING | 45.11 | DiseaseFree | 45.11 |
| TCGA-CJ-4918-01A-01D-1424-05 | Cancer | MALE | 64 | T3a | N0 | M1 | Stage IV | G4 | DECEASED | 3.06 | Recurred/Progressed | 1.08 |
| TCGA-CJ-4920-01A-01D-1424-05 | Cancer | FEMALE | 64 | T1b | NX | M0 | Stage I | G2 | DECEASED | 4.57 | Unknown | Unknown |
| TCGA-CJ-4923-01A-01D-1424-05 | Cancer | FEMALE | 63 | T3a | NX | M1 | Stage IV | G4 | DECEASED | 18.79 | Recurred/Progressed | 0.82 |
| TCGA-CJ-5671-01A-11D-1536-05 | Cancer | MALE | 51 | T1a | NX | M0 | Stage I | G3 | LIVING | 130.98 | DiseaseFree | 130.98 |
| TCGA-CJ-5672-01A-11D-1536-05 | Cancer | MALE | 84 | T1a | NX | M0 | Stage I | G3 | DECEASED | 71.94 | Unknown | Unknown |
| TCGA-CJ-5675-01A-11D-1536-05 | Cancer | MALE | 70 | T2a | NX | M0 | Stage II | G3 | LIVING | 129.3 | Recurred/Progressed | 29.57 |
| TCGA-CJ-5676-01A-11D-1536-05 | Cancer | MALE | 47 | T3b | NX | M0 | Stage III | G3 | LIVING | 133.61 | Recurred/Progressed | 72.9 |
| TCGA-CJ-5677-01A-11D-1536-05 | Cancer | FEMALE | 54 | T3a | NX | M1 | Stage IV | G4 | DECEASED | 25.69 | Recurred/Progressed | 9.46 |
| TCGA-CJ-5678-01A-11D-1536-05 | Cancer | MALE | 62 | T2b | N0 | M1 | Stage IV | G3 | DECEASED | 18.86 | Recurred/Progressed | 13.11 |
| TCGA-CJ-5679-01A-11D-1536-05 | Cancer | MALE | 73 | T3b | NX | M0 | Stage III | G4 | DECEASED | 22.31 | Recurred/Progressed | 19.55 |
| TCGA-CJ-5680-01A-11D-1536-05 | Cancer | FEMALE | 65 | T3a | NX | M1 | Stage IV | G4 | DECEASED | 25.23 | Recurred/Progressed | 5.03 |
| TCGA-CJ-5681-01A-11D-1536-05 | Cancer | FEMALE | 44 | T3a | NX | M1 | Stage IV | G3 | DECEASED | 18.13 | Recurred/Progressed | 0.56 |
| TCGA-CJ-5682-01A-11D-1536-05 | Cancer | MALE | 60 | T3a | NX | M1 | Stage IV | G4 | LIVING | 122.73 | Recurred/Progressed | 1.81 |
| TCGA-CJ-5683-01A-11D-1536-05 | Cancer | MALE | 78 | T1b | NX | M0 | Stage I | G3 | LIVING | 62.06 | DiseaseFree | 62.06 |
| TCGA-CJ-5684-01A-11D-1536-05 | Cancer | MALE | 61 | T3a | NX | M0 | Stage III | G2 | LIVING | 73.29 | DiseaseFree | 73.29 |
| TCGA-CJ-5686-01A-11D-1670-05 | Cancer | FEMALE | 59 | T1b | NX | M0 | Stage I | G3 | LIVING | 66.95 | DiseaseFree | 66.95 |
| TCGA-CJ-5689-01A-11D-1536-05 | Cancer | MALE | 90 | T1b | NX | M0 | Stage I | G4 | DECEASED | 53.22 | Unknown | Unknown |
| TCGA-CJ-6027-01A-11D-1670-05 | Cancer | MALE | 77 | T1a | NX | M0 | Stage I | G4 | DECEASED | 118.76 | Unknown | Unknown |
| TCGA-CJ-6028-01A-11D-1670-05 | Cancer | MALE | 58 | T3a | NX | M1 | Stage IV | G4 | DECEASED | 53.38 | Recurred/Progressed | 4.5 |
| TCGA-CJ-6030-01A-11D-1670-05 | Cancer | MALE | 65 | T1a | N0 | M0 | Stage I | G3 | DECEASED | 75.53 | Unknown | Unknown |
| TCGA-CJ-6031-01A-11D-1670-05 | Cancer | MALE | 54 | T1b | NX | M0 | Stage I | G3 | LIVING | 62.61 | DiseaseFree | 62.61 |
| TCGA-CJ-6032-01A-11D-1670-05 | Cancer | FEMALE | 63 | T2 | NX | M0 | Stage II | G3 | LIVING | 119.55 | DiseaseFree | 119.55 |
| TCGA-CJ-6033-01A-11D-1670-05 | Cancer | FEMALE | 54 | T3a | N0 | M1 | Stage IV | G4 | DECEASED | 7.36 | Recurred/Progressed | 4.73 |
| TCGA-CW-5580-01A-01D-1670-05 | Cancer | FEMALE | 73 | T3a | NX | M1 | Stage IV | G3 | DECEASED | 64.52 | Unknown | Unknown |
| TCGA-CW-5581-01A-02D-1536-05 | Cancer | MALE | 44 | T1b | NX | M0 | Stage I | G3 | LIVING | 91.95 | DiseaseFree | 91.95 |
| TCGA-CW-5583-01A-02D-1536-05 | Cancer | FEMALE | 51 | T1a | NX | M0 | Stage I | G2 | LIVING | 81.77 | DiseaseFree | 81.77 |
| TCGA-CW-5584-01A-01D-1536-05 | Cancer | MALE | 74 | T3b | N1 | M0 | Stage III | G3 | DECEASED | 5.39 | Recurred/Progressed | 3.35 |
| TCGA-CW-5585-01A-01D-1536-05 | Cancer | MALE | 51 | T3b | N0 | M1 | Stage IV | G2 | LIVING | 85.71 | DiseaseFree | 85.71 |
| TCGA-CW-5587-01A-01D-1536-05 | Cancer | FEMALE | 62 | T3b | N0 | M0 | Stage III | G2 | LIVING | 73.13 | Recurred/Progressed | 65.8 |
| TCGA-CW-5588-01A-01D-1536-05 | Cancer | FEMALE | 78 | T1a | NX | M0 | Stage I | G2 | LIVING | 66.26 | DiseaseFree | 66.26 |
| TCGA-CW-5589-01A-01D-1536-05 | Cancer | MALE | 52 | T1a | NX | M0 | Stage I | G2 | LIVING | 78.12 | DiseaseFree | 78.12 |
| TCGA-CW-5590-01A-01D-1536-05 | Cancer | MALE | 51 | T3a | NX | M1 | Stage IV | G3 | DECEASED | 35.32 | Recurred/Progressed | 22.08 |
| TCGA-CW-5591-01A-01D-1536-05 | Cancer | MALE | 56 | T3a | N0 | M1 | Stage IV | G2 | LIVING | 74.61 | DiseaseFree | 74.61 |
| TCGA-CW-6087-01A-11D-1670-05 | Cancer | MALE | 61 | T3a | N1 | M1 | Stage IV | G4 | DECEASED | 1.35 | Unknown | Unknown |
| TCGA-CW-6088-01A-11D-1670-05 | Cancer | MALE | 60 | T1b | N0 | M0 | Stage I | G2 | LIVING | 105.85 | DiseaseFree | 105.85 |
| TCGA-CW-6090-01A-11D-1670-05 | Cancer | MALE | 68 | T1b | NX | M0 | Stage I | G3 | LIVING | 83.84 | Recurred/Progressed | 91.33 |
| TCGA-CW-6093-01A-11D-1670-05 | Cancer | MALE | 73 | T1a | NX | M0 | Stage I | G1 | LIVING | 103.35 | DiseaseFree | 103.35 |
| TCGA-CW-6097-01A-11D-1670-05 | Cancer | MALE | 32 | T3a | NX | M0 | Stage III | G4 | DECEASED | 18.76 | Recurred/Progressed | 15.01 |
| TCGA-CZ-4853-01A-01D-1424-05 | Cancer | MALE | 82 | T1a | NX | M0 | Stage I | G2 | LIVING | 25.43 | DiseaseFree | 25.43 |
| TCGA-CZ-4856-01A-02D-1424-05 | Cancer | FEMALE | 62 | T1b | N0 | M0 | Stage I | G2 | LIVING | 0.59 | DiseaseFree | 0.59 |
| TCGA-CZ-4859-01A-02D-1424-05 | Cancer | FEMALE | 59 | T1 | N0 | M0 | Stage I | G2 | LIVING | 58.71 | DiseaseFree | 58.71 |
| TCGA-CZ-4863-01A-01D-1500-05 | Cancer | FEMALE | 51 | T3b | N0 | M0 | Stage III | G3 | LIVING | 63.34 | DiseaseFree | 63.34 |
| TCGA-CZ-4864-01A-01D-1500-05 | Cancer | MALE | 86 | T2 | N0 | M0 | Stage II | G3 | DECEASED | 92.97 | Unknown | Unknown |
| TCGA-CZ-4865-01A-02D-1500-05 | Cancer | FEMALE | 70 | T1a | NX | M0 | Stage I | G2 | DECEASED | 5.45 | Unknown | Unknown |
| TCGA-CZ-5451-01A-01D-1500-05 | Cancer | MALE | 74 | T2 | N0 | M0 | Stage II | G3 | LIVING | 63.37 | DiseaseFree | 63.37 |
| TCGA-CZ-5452-01A-01D-1500-05 | Cancer | MALE | 69 | T2 | N0 | M0 | Stage II | G2 | LIVING | 58.77 | DiseaseFree | 58.77 |
| TCGA-CZ-5453-01A-01D-1500-05 | Cancer | MALE | 67 | T2 | NX | M0 | Stage II | G2 | DECEASED | 79.47 | Unknown | Unknown |
| TCGA-CZ-5454-01A-01D-1500-05 | Cancer | MALE | 63 | T2 | N0 | M1 | Stage IV | G2 | DECEASED | 23.72 | Recurred/Progressed | 13.9 |
| TCGA-CZ-5455-01A-01D-1500-05 | Cancer | MALE | 63 | T3b | NX | M1 | Stage IV | G4 | DECEASED | 18.43 | Unknown | Unknown |
| TCGA-CZ-5456-01A-01D-1500-05 | Cancer | MALE | 57 | T2 | N0 | M0 | Stage II | G3 | LIVING | 79.57 | Recurred/Progressed | 31.24 |
| TCGA-CZ-5457-01A-01D-1500-05 | Cancer | MALE | 62 | T3a | NX | M0 | Stage III | G4 | LIVING | 90.47 | Recurred/Progressed | 5.72 |
| TCGA-CZ-5458-01A-01D-1500-05 | Cancer | MALE | 43 | T3a | NX | M0 | Stage III | G3 | LIVING | 91.62 | DiseaseFree | 91.62 |
| TCGA-CZ-5459-01A-01D-1500-05 | Cancer | MALE | 63 | T3b | NX | M0 | Stage III | G3 | LIVING | 55.29 | DiseaseFree | 55.29 |
| TCGA-CZ-5460-01A-01D-1500-05 | Cancer | MALE | 55 | T3b | NX | M1 | Stage IV | G2 | LIVING | 94.38 | DiseaseFree | 94.38 |
| TCGA-CZ-5461-01A-01D-1500-05 | Cancer | MALE | 52 | T1b | NX | M1 | Stage IV | G4 | DECEASED | 10.84 | Recurred/Progressed | 1.58 |
| TCGA-CZ-5462-01A-01D-1500-05 | Cancer | MALE | 83 | T1b | NX | M1 | Stage IV | G3 | DECEASED | 10.22 | Unknown | Unknown |
| TCGA-CZ-5463-01A-01D-1500-05 | Cancer | MALE | 76 | T2 | NX | M0 | Stage II | G2 | LIVING | 21.75 | DiseaseFree | 21.75 |
| TCGA-CZ-5464-01A-01D-1500-05 | Cancer | MALE | 69 | T3b | NX | M1 | Stage IV | G2 | LIVING | 69.91 | DiseaseFree | 69.91 |
| TCGA-CZ-5465-01A-01D-1500-05 | Cancer | FEMALE | 76 | T3b | NX | M0 | Stage III | G2 | DECEASED | 84.23 | Unknown | Unknown |
| TCGA-CZ-5466-01A-01D-1500-05 | Cancer | MALE | 67 | T3a | NX | M0 | Stage III | G2 | LIVING | 22.5 | DiseaseFree | 22.5 |
| TCGA-CZ-5467-01A-01D-1500-05 | Cancer | FEMALE | 86 | T3a | N0 | M0 | Stage III | G4 | DECEASED | 2.4 | Recurred/Progressed | 2.04 |
| TCGA-CZ-5469-01A-01D-1500-05 | Cancer | MALE | 41 | T2 | N0 | M0 | Stage II | G2 | DECEASED | 31.08 | Recurred/Progressed | 3.61 |
| TCGA-CZ-5470-01A-01D-1500-05 | Cancer | FEMALE | 72 | T2 | N0 | M0 | Stage II | G3 | LIVING | 12.68 | DiseaseFree | 12.68 |
| TCGA-CZ-5982-01A-11D-1670-05 | Cancer | FEMALE | 59 | T1a | NX | M0 | Stage I | G2 | LIVING | 80.12 | DiseaseFree | 80.12 |
| TCGA-CZ-5984-01A-11D-1670-05 | Cancer | MALE | 51 | T1b | N0 | M0 | Stage I | G3 | LIVING | 67.9 | DiseaseFree | 67.9 |
| TCGA-CZ-5985-01A-11D-1670-05 | Cancer | MALE | 58 | T2 | N0 | M0 | Stage II | G2 | LIVING | 65.6 | DiseaseFree | 65.6 |
| TCGA-CZ-5986-01A-11D-1670-05 | Cancer | MALE | 61 | T1 | N0 | M0 | Stage I | G3 | LIVING | 12.25 | DiseaseFree | 12.25 |
| TCGA-CZ-5987-01A-11D-1670-05 | Cancer | MALE | 60 | T3b | NX | M1 | Stage IV | G2 | DECEASED | 14.62 | Recurred/Progressed | 12.39 |
| TCGA-CZ-5988-01A-11D-1670-05 | Cancer | MALE | 38 | T1b | N0 | M0 | Stage I | G2 | LIVING | 22.77 | DiseaseFree | 22.77 |
| TCGA-CZ-5989-01A-11D-1670-05 | Cancer | MALE | 60 | T2 | N0 | M0 | Stage II | G2 | LIVING | 62.58 | DiseaseFree | 62.58 |
| TCGA-DV-5565-01A-01D-1536-05 | Cancer | MALE | 59 | T1a | NX | M0 | Stage I | G2 | LIVING | 43.66 | DiseaseFree | 43.66 |
| TCGA-DV-5566-01A-01D-1536-05 | Cancer | FEMALE | 67 | T1a | NX | M0 | Stage I | G2 | LIVING | 45.93 | DiseaseFree | 45.93 |
| TCGA-DV-5567-01A-01D-1536-05 | Cancer | FEMALE | 40 | T1a | NX | M0 | Stage I | G2 | LIVING | 65.83 | Recurred/Progressed | 65.83 |
| TCGA-DV-5568-01A-01D-1536-05 | Cancer | MALE | 26 | T1a | NX | M0 | Stage I | G2 | LIVING | 12.16 | DiseaseFree | 12.16 |
| TCGA-DV-5569-01A-01D-1536-05 | Cancer | FEMALE | 29 | T1a | NX | M0 | Stage I | G2 | LIVING | 11.66 | DiseaseFree | 11.66 |
| TCGA-DV-5573-01A-01D-1536-05 | Cancer | MALE | 41 | T1a | NX | M0 | Stage I | G2 | LIVING | 37.12 | DiseaseFree | 37.12 |
| TCGA-DV-5574-01A-01D-1536-05 | Cancer | MALE | 37 | T1a | NX | M0 | Stage I | G2 | LIVING | 66.23 | Recurred/Progressed | 11.73 |
| TCGA-DV-5575-01A-01D-1536-05 | Cancer | FEMALE | 52 | T1a | NX | M0 | Stage I | G2 | LIVING | 56.8 | DiseaseFree | 56.8 |
| TCGA-DV-5576-01A-01D-1536-05 | Cancer | FEMALE | 55 | T1a | NX | M0 | Stage I | G2 | DECEASED | 23.88 | Unknown | Unknown |
| TCGA-DV-A4VX-01A-11D-A264-05 | Cancer | MALE | 59 | T3b | N0 | MX | Stage IV | G4 | DECEASED | 53.42 | Unknown | Unknown |
| TCGA-DV-A4VZ-01A-11D-A264-05 | Cancer | MALE | 53 | T1a | NX | MX | Stage I | G2 | LIVING | 11.99 | DiseaseFree | 11.99 |
| TCGA-DV-A4W0-01A-11D-A264-05 | Cancer | MALE | 55 | T1b | NX | MX | Stage I | G3 | LIVING | 81.14 | Recurred/Progressed | 65.97 |
| TCGA-DV-A4W0-05A-11D-A264-05 | Cancer | MALE | 55 | T1b | NX | MX | Stage I | G3 | LIVING | 81.14 | Recurred/Progressed | 65.97 |
| TCGA-EU-5904-01A-11D-1670-05 | Cancer | FEMALE | 47 | T1 | NX | M0 | Stage I | G1 | LIVING | 18.1 | DiseaseFree | 18.1 |
| TCGA-EU-5905-01A-11D-1670-05 | Cancer | FEMALE | 67 | T1 | NX | M0 | Stage I | G3 | LIVING | 3.91 | DiseaseFree | 3.91 |
| TCGA-EU-5906-01A-11D-1670-05 | Cancer | MALE | 55 | T1b | NX | M0 | Stage I | G2 | LIVING | 6.77 | DiseaseFree | 6.77 |
| TCGA-EU-5907-01A-11D-1670-05 | Cancer | MALE | 81 | T3a | NX | M0 | Stage III | G3 | LIVING | 4.17 | DiseaseFree | 4.17 |
| TCGA-G6-A5PC-01A-11D-A33L-05 | Cancer | FEMALE | 54 | T1b | N0 | M1 | Stage IV | G4 | DECEASED | 7.95 | Unknown | Unknown |
| TCGA-G6-A8L6-01A-11D-A36Y-05 | Cancer | MALE | 55 | T2a | NX | MX | Stage IV | G3 | DECEASED | 10.28 | Recurred/Progressed | 9.99 |
| TCGA-G6-A8L7-01A-11D-A36Y-05 | Cancer | FEMALE | 81 | T1b | N0 | MX | Stage I | G3 | LIVING | 70.07 | DiseaseFree | 70.07 |
| TCGA-G6-A8L8-01A-21D-A36Y-05 | Cancer | FEMALE | 62 | T1b | NX | MX | Stage I | G3 | DECEASED | 35.84 | Unknown | Unknown |
| TCGA-GK-A6C7-01A-11D-A33L-05 | Cancer | FEMALE | 76 | T1a | NX | MX | Stage I | Unknown | LIVING | 2 | DiseaseFree | 2 |
| TCGA-MM-A563-01A-11D-A264-05 | Cancer | MALE | 41 | T3 | NX | MX | Unknown | G2 | LIVING | 19.42 | DiseaseFree | 19.42 |
| TCGA-MM-A564-01A-11D-A264-05 | Cancer | MALE | 68 | T2a | NX | MX | Stage II | G2 | LIVING | 19.94 | DiseaseFree | 19.94 |
| TCGA-MM-A84U-01A-11D-A36Y-05 | Cancer | FEMALE | 58 | T1a | NX | MX | Stage I | G2 | LIVING | 23 | DiseaseFree | 23 |
| TCGA-MW-A4EC-01A-11D-A264-05 | Cancer | FEMALE | 72 | T1a | NX | MX | Stage I | G2 | LIVING | 16.36 | DiseaseFree | 16.36 |
| TCGA-T7-A92I-01A-11D-A36Y-05 | Cancer | FEMALE | 47 | T1a | NX | MX | Stage I | G1 | LIVING | 11.7 | DiseaseFree | 11.7 |
| GSM2386069 | Cancer | FEMALE | Unknown | Unknown | Unknown | Unknown | Stage III | Unknown | Unknown | Unknown | Unknown | Unknown |
| GSM2386065 | Cancer | FEMALE | Unknown | Unknown | Unknown | Unknown | Stage III | Unknown | Unknown | Unknown | Unknown | Unknown |
| GSM2386064 | Cancer | MALE | Unknown | Unknown | Unknown | Unknown | Stage II | Unknown | Unknown | Unknown | Unknown | Unknown |
| GSM1633616 | Cancer | Unknown | Unknown | Unknown | Unknown | Unknown | Unknown | Unknown | Unknown | Unknown | Unknown | Unknown |
| GSM2386073 | Cancer | MALE | Unknown | Unknown | Unknown | Unknown | Stage II | Unknown | Unknown | Unknown | Unknown | Unknown |
| GSM1633646 | Cancer | Unknown | Unknown | Unknown | Unknown | Unknown | Unknown | Unknown | Unknown | Unknown | Unknown | Unknown |
| GSM2386072 | Cancer | MALE | Unknown | Unknown | Unknown | Unknown | Stage II | Unknown | Unknown | Unknown | Unknown | Unknown |
| GSM2386067 | Cancer | MALE | Unknown | Unknown | Unknown | Unknown | Stage II | Unknown | Unknown | Unknown | Unknown | Unknown |
| GSM2386063 | Cancer | FEMALE | Unknown | Unknown | Unknown | Unknown | Stage III | Unknown | Unknown | Unknown | Unknown | Unknown |
| GSM2386070 | Cancer | MALE | Unknown | Unknown | Unknown | Unknown | Stage III | Unknown | Unknown | Unknown | Unknown | Unknown |
| GSM2386071 | Cancer | MALE | Unknown | Unknown | Unknown | Unknown | Stage II | Unknown | Unknown | Unknown | Unknown | Unknown |
| GSM2386068 | Cancer | FEMALE | Unknown | Unknown | Unknown | Unknown | Stage I | Unknown | Unknown | Unknown | Unknown | Unknown |
| GSM2386062 | Cancer | MALE | Unknown | Unknown | Unknown | Unknown | Stage I | Unknown | Unknown | Unknown | Unknown | Unknown |
| GSM2386066 | Cancer | MALE | Unknown | Unknown | Unknown | Unknown | Stage II | Unknown | Unknown | Unknown | Unknown | Unknown |
| TCGA-A3-3357-11A-01D-1418-05 | Normal | MALE | 62 | - | - | - | - | - | - | - | - | - |
| TCGA-A3-3367-11A-01D-1418-05 | Normal | MALE | 72 | - | - | - | - | - | - | - | - | - |
| TCGA-A3-3370-11A-01D-1418-05 | Normal | FEMALE | 48 | - | - | - | - | - | - | - | - | - |
| TCGA-A3-3376-11A-01D-1418-05 | Normal | MALE | 51 | - | - | - | - | - | - | - | - | - |
| TCGA-A3-3385-11A-01D-1418-05 | Normal | FEMALE | 46 | - | - | - | - | - | - | - | - | - |
| TCGA-B0-4688-11A-01D-1275-05 | Normal | MALE | 46 | - | - | - | - | - | - | - | - | - |
| TCGA-B0-4690-11A-01D-1275-05 | Normal | MALE | 65 | - | - | - | - | - | - | - | - | - |
| TCGA-B0-4691-11A-01D-1275-05 | Normal | MALE | 55 | - | - | - | - | - | - | - | - | - |
| TCGA-B0-4693-11A-01D-1275-05 | Normal | FEMALE | 72 | - | - | - | - | - | - | - | - | - |
| TCGA-B0-4694-11A-01D-1275-05 | Normal | MALE | 72 | - | - | - | - | - | - | - | - | - |
| TCGA-B0-4696-11A-01D-1275-05 | Normal | MALE | 58 | - | - | - | - | - | - | - | - | - |
| TCGA-B0-4697-11A-01D-1275-05 | Normal | FEMALE | 46 | - | - | - | - | - | - | - | - | - |
| TCGA-B0-4699-11A-01D-1275-05 | Normal | MALE | 74 | - | - | - | - | - | - | - | - | - |
| TCGA-B0-4701-11A-01D-1275-05 | Normal | FEMALE | 66 | - | - | - | - | - | - | - | - | - |
| TCGA-B0-4703-11A-01D-1275-05 | Normal | MALE | 51 | - | - | - | - | - | - | - | - | - |
| TCGA-B0-4706-11A-02D-1500-05 | Normal | MALE | 61 | - | - | - | - | - | - | - | - | - |
| TCGA-B0-4707-11A-01D-1275-05 | Normal | MALE | 63 | - | - | - | - | - | - | - | - | - |
| TCGA-B0-4710-11A-02D-1500-05 | Normal | FEMALE | 75 | - | - | - | - | - | - | - | - | - |
| TCGA-B0-4712-11A-02D-1500-05 | Normal | MALE | 76 | - | - | - | - | - | - | - | - | - |
| TCGA-B0-4713-11A-01D-1275-05 | Normal | FEMALE | 76 | - | - | - | - | - | - | - | - | - |
| TCGA-B0-4714-11A-01D-1275-05 | Normal | MALE | 81 | - | - | - | - | - | - | - | - | - |
| TCGA-B0-4718-11A-01D-1275-05 | Normal | MALE | 57 | - | - | - | - | - | - | - | - | - |
| TCGA-B0-4810-11A-02D-1500-05 | Normal | MALE | 47 | - | - | - | - | - | - | - | - | - |
| TCGA-B0-4811-11A-02D-1500-05 | Normal | MALE | 48 | - | - | - | - | - | - | - | - | - |
| TCGA-B0-4813-11A-01D-1275-05 | Normal | MALE | 68 | - | - | - | - | - | - | - | - | - |
| TCGA-B0-4814-11A-01D-1275-05 | Normal | MALE | 58 | - | - | - | - | - | - | - | - | - |
| TCGA-B0-4815-11A-02D-1500-05 | Normal | MALE | 65 | - | - | - | - | - | - | - | - | - |
| TCGA-B0-4816-11A-02D-1500-05 | Normal | MALE | 49 | - | - | - | - | - | - | - | - | - |
| TCGA-B0-4817-11A-01D-1275-05 | Normal | MALE | 81 | - | - | - | - | - | - | - | - | - |
| TCGA-B0-4818-11A-01D-1500-05 | Normal | FEMALE | 68 | - | - | - | - | - | - | - | - | - |
| TCGA-B0-4819-11A-01D-1275-05 | Normal | FEMALE | 60 | - | - | - | - | - | - | - | - | - |
| TCGA-B0-4821-11A-01D-1500-05 | Normal | FEMALE | 68 | - | - | - | - | - | - | - | - | - |
| TCGA-B0-4822-11A-01D-1275-05 | Normal | MALE | 78 | - | - | - | - | - | - | - | - | - |
| TCGA-B0-4823-11A-01D-1418-05 | Normal | MALE | 88 | - | - | - | - | - | - | - | - | - |
| TCGA-B0-4824-11A-01D-1275-05 | Normal | FEMALE | 49 | - | - | - | - | - | - | - | - | - |
| TCGA-B0-4827-11A-01D-1418-05 | Normal | FEMALE | 77 | - | - | - | - | - | - | - | - | - |
| TCGA-B0-4828-11A-01D-1275-05 | Normal | MALE | 79 | - | - | - | - | - | - | - | - | - |
| TCGA-B0-4841-11A-01D-1275-05 | Normal | MALE | 63 | - | - | - | - | - | - | - | - | - |
| TCGA-B0-4842-11A-01D-1418-05 | Normal | FEMALE | 73 | - | - | - | - | - | - | - | - | - |
| TCGA-B0-4843-11A-01D-1275-05 | Normal | MALE | 57 | - | - | - | - | - | - | - | - | - |
| TCGA-B0-4844-11A-01D-1275-05 | Normal | MALE | 60 | - | - | - | - | - | - | - | - | - |
| TCGA-B0-4845-11A-01D-1275-05 | Normal | MALE | 70 | - | - | - | - | - | - | - | - | - |
| TCGA-B0-4846-11A-01D-1275-05 | Normal | MALE | 52 | - | - | - | - | - | - | - | - | - |
| TCGA-B0-4847-11A-01D-1275-05 | Normal | MALE | 60 | - | - | - | - | - | - | - | - | - |
| TCGA-B0-4848-11A-01D-1275-05 | Normal | MALE | 54 | - | - | - | - | - | - | - | - | - |
| TCGA-B0-4849-11A-01D-1275-05 | Normal | MALE | 51 | - | - | - | - | - | - | - | - | - |
| TCGA-B0-4852-11A-01D-1500-05 | Normal | FEMALE | 78 | - | - | - | - | - | - | - | - | - |
| TCGA-B0-4945-11A-01D-1418-05 | Normal | FEMALE | 75 | - | - | - | - | - | - | - | - | - |
| TCGA-B0-5080-11A-01D-1500-05 | Normal | MALE | 63 | - | - | - | - | - | - | - | - | - |
| TCGA-B0-5083-11A-01D-1418-05 | Normal | MALE | 63 | - | - | - | - | - | - | - | - | - |
| TCGA-B0-5092-11A-01D-1418-05 | Normal | FEMALE | 53 | - | - | - | - | - | - | - | - | - |
| TCGA-B0-5094-11A-01D-1418-05 | Normal | MALE | 62 | - | - | - | - | - | - | - | - | - |
| TCGA-B0-5095-11A-01D-1418-05 | Normal | MALE | 81 | - | - | - | - | - | - | - | - | - |
| TCGA-B0-5096-11A-01D-1418-05 | Normal | FEMALE | 72 | - | - | - | - | - | - | - | - | - |
| TCGA-B0-5097-11A-01D-1418-05 | Normal | FEMALE | 59 | - | - | - | - | - | - | - | - | - |
| TCGA-B0-5098-11A-01D-1418-05 | Normal | FEMALE | 53 | - | - | - | - | - | - | - | - | - |
| TCGA-B0-5099-11A-01D-1418-05 | Normal | FEMALE | 88 | - | - | - | - | - | - | - | - | - |
| TCGA-B0-5100-11A-01D-1418-05 | Normal | MALE | 72 | - | - | - | - | - | - | - | - | - |
| TCGA-B0-5102-11A-01D-1418-05 | Normal | FEMALE | 74 | - | - | - | - | - | - | - | - | - |
| TCGA-B0-5104-11A-01D-1418-05 | Normal | FEMALE | 90 | - | - | - | - | - | - | - | - | - |
| TCGA-B0-5106-11A-01D-1418-05 | Normal | MALE | 64 | - | - | - | - | - | - | - | - | - |
| TCGA-B0-5107-11A-01D-1418-05 | Normal | FEMALE | 65 | - | - | - | - | - | - | - | - | - |
| TCGA-B0-5108-11A-01D-1418-05 | Normal | MALE | 54 | - | - | - | - | - | - | - | - | - |
| TCGA-B0-5109-11A-01D-1418-05 | Normal | MALE | 69 | - | - | - | - | - | - | - | - | - |
| TCGA-B0-5110-11A-01D-1418-05 | Normal | FEMALE | 71 | - | - | - | - | - | - | - | - | - |
| TCGA-B0-5113-11A-01D-1418-05 | Normal | FEMALE | 69 | - | - | - | - | - | - | - | - | - |
| TCGA-B0-5115-11A-01D-1418-05 | Normal | MALE | 43 | - | - | - | - | - | - | - | - | - |
| TCGA-B0-5116-11A-01D-1418-05 | Normal | MALE | 52 | - | - | - | - | - | - | - | - | - |
| TCGA-B0-5117-11A-01D-1418-05 | Normal | MALE | 40 | - | - | - | - | - | - | - | - | - |
| TCGA-B0-5119-11A-01D-1418-05 | Normal | FEMALE | 61 | - | - | - | - | - | - | - | - | - |
| TCGA-B0-5120-11A-01D-1418-05 | Normal | FEMALE | 72 | - | - | - | - | - | - | - | - | - |
| TCGA-B0-5121-11A-01D-1418-05 | Normal | MALE | 56 | - | - | - | - | - | - | - | - | - |
| TCGA-B0-5400-11A-01D-1500-05 | Normal | FEMALE | 59 | - | - | - | - | - | - | - | - | - |
| TCGA-B0-5402-11A-01D-1500-05 | Normal | MALE | 64 | - | - | - | - | - | - | - | - | - |
| TCGA-B0-5710-11A-01D-1670-05 | Normal | MALE | 57 | - | - | - | - | - | - | - | - | - |
| TCGA-B0-5711-11A-01D-1670-05 | Normal | MALE | 50 | - | - | - | - | - | - | - | - | - |
| TCGA-B0-5712-11A-01D-1670-05 | Normal | FEMALE | 68 | - | - | - | - | - | - | - | - | - |
| TCGA-B0-5713-11A-01D-1670-05 | Normal | FEMALE | 75 | - | - | - | - | - | - | - | - | - |
| TCGA-BP-4760-11A-01D-1418-05 | Normal | MALE | 69 | - | - | - | - | - | - | - | - | - |
| TCGA-BP-4770-11A-01D-1500-05 | Normal | FEMALE | 73 | - | - | - | - | - | - | - | - | - |
| TCGA-BP-4782-11A-01D-1418-05 | Normal | FEMALE | 55 | - | - | - | - | - | - | - | - | - |
| TCGA-BP-4795-11A-01D-1418-05 | Normal | FEMALE | 74 | - | - | - | - | - | - | - | - | - |
| TCGA-BP-4801-11A-01D-1418-05 | Normal | MALE | 57 | - | - | - | - | - | - | - | - | - |
| TCGA-BP-4993-11A-01D-1418-05 | Normal | MALE | 58 | - | - | - | - | - | - | - | - | - |
| TCGA-BP-5010-11A-01D-1418-05 | Normal | MALE | 63 | - | - | - | - | - | - | - | - | - |
| TCGA-BP-5168-11A-01D-1418-05 | Normal | MALE | 75 | - | - | - | - | - | - | - | - | - |
| TCGA-BP-5169-11A-01D-1424-05 | Normal | MALE | 70 | - | - | - | - | - | - | - | - | - |
| TCGA-BP-5170-11A-01D-1424-05 | Normal | MALE | 55 | - | - | - | - | - | - | - | - | - |
| TCGA-BP-5173-11A-01D-1424-05 | Normal | MALE | 75 | - | - | - | - | - | - | - | - | - |
| TCGA-BP-5174-11A-01D-1424-05 | Normal | FEMALE | 45 | - | - | - | - | - | - | - | - | - |
| TCGA-BP-5175-11A-01D-1424-05 | Normal | MALE | 60 | - | - | - | - | - | - | - | - | - |
| TCGA-BP-5176-11A-01D-1424-05 | Normal | FEMALE | 78 | - | - | - | - | - | - | - | - | - |
| TCGA-BP-5177-11A-01D-1424-05 | Normal | FEMALE | 46 | - | - | - | - | - | - | - | - | - |
| TCGA-BP-5178-11A-01D-1424-05 | Normal | MALE | 71 | - | - | - | - | - | - | - | - | - |
| TCGA-BP-5180-11A-01D-1424-05 | Normal | MALE | 53 | - | - | - | - | - | - | - | - | - |
| TCGA-BP-5181-11A-01D-1424-05 | Normal | FEMALE | 58 | - | - | - | - | - | - | - | - | - |
| TCGA-BP-5182-11A-01D-1424-05 | Normal | MALE | 56 | - | - | - | - | - | - | - | - | - |
| TCGA-BP-5183-11A-01D-1424-05 | Normal | MALE | 57 | - | - | - | - | - | - | - | - | - |
| TCGA-BP-5185-11A-01D-1424-05 | Normal | MALE | 56 | - | - | - | - | - | - | - | - | - |
| TCGA-BP-5186-11A-01D-1424-05 | Normal | FEMALE | 50 | - | - | - | - | - | - | - | - | - |
| TCGA-BP-5187-11A-01D-1424-05 | Normal | MALE | 54 | - | - | - | - | - | - | - | - | - |
| TCGA-BP-5189-11A-01D-1424-05 | Normal | MALE | 60 | - | - | - | - | - | - | - | - | - |
| TCGA-BP-5190-11A-01D-1424-05 | Normal | MALE | 61 | - | - | - | - | - | - | - | - | - |
| TCGA-BP-5191-11A-01D-1424-05 | Normal | MALE | 79 | - | - | - | - | - | - | - | - | - |
| TCGA-BP-5192-11A-01D-1424-05 | Normal | MALE | 59 | - | - | - | - | - | - | - | - | - |
| TCGA-BP-5194-11A-01D-1424-05 | Normal | MALE | 39 | - | - | - | - | - | - | - | - | - |
| TCGA-BP-5195-11A-01D-1424-05 | Normal | MALE | 75 | - | - | - | - | - | - | - | - | - |
| TCGA-BP-5196-11A-01D-1424-05 | Normal | MALE | 53 | - | - | - | - | - | - | - | - | - |
| TCGA-BP-5198-11A-01D-1424-05 | Normal | MALE | 72 | - | - | - | - | - | - | - | - | - |
| TCGA-BP-5199-11A-01D-1424-05 | Normal | MALE | 58 | - | - | - | - | - | - | - | - | - |
| TCGA-BP-5200-11A-01D-1424-05 | Normal | MALE | 44 | - | - | - | - | - | - | - | - | - |
| TCGA-BP-5201-11A-01D-1424-05 | Normal | MALE | 63 | - | - | - | - | - | - | - | - | - |
| TCGA-BP-5202-11A-01D-1424-05 | Normal | MALE | 75 | - | - | - | - | - | - | - | - | - |
| TCGA-CJ-4869-11A-01D-1424-05 | Normal | MALE | 49 | - | - | - | - | - | - | - | - | - |
| TCGA-CJ-4882-11A-01D-1424-05 | Normal | MALE | 57 | - | - | - | - | - | - | - | - | - |
| TCGA-CJ-4901-11A-01D-1424-05 | Normal | MALE | 47 | - | - | - | - | - | - | - | - | - |
| TCGA-CJ-4902-11A-01D-1424-05 | Normal | MALE | 61 | - | - | - | - | - | - | - | - | - |
| TCGA-CJ-4903-11A-01D-1424-05 | Normal | MALE | 50 | - | - | - | - | - | - | - | - | - |
| TCGA-CJ-4904-11A-01D-1424-05 | Normal | FEMALE | 60 | - | - | - | - | - | - | - | - | - |
| TCGA-CJ-4905-11A-01D-1424-05 | Normal | FEMALE | 62 | - | - | - | - | - | - | - | - | - |
| TCGA-CJ-4907-11A-01D-1424-05 | Normal | MALE | 58 | - | - | - | - | - | - | - | - | - |
| TCGA-CJ-4908-11A-01D-1424-05 | Normal | MALE | 38 | - | - | - | - | - | - | - | - | - |
| TCGA-CJ-4912-11A-01D-1424-05 | Normal | MALE | 61 | - | - | - | - | - | - | - | - | - |
| TCGA-CJ-4913-11A-01D-1424-05 | Normal | FEMALE | 45 | - | - | - | - | - | - | - | - | - |
| TCGA-CJ-4916-11A-01D-1424-05 | Normal | FEMALE | 69 | - | - | - | - | - | - | - | - | - |
| TCGA-CJ-4918-11A-01D-1424-05 | Normal | MALE | 64 | - | - | - | - | - | - | - | - | - |
| TCGA-CJ-4920-11A-01D-1424-05 | Normal | FEMALE | 64 | - | - | - | - | - | - | - | - | - |
| TCGA-CJ-4923-11A-01D-1424-05 | Normal | FEMALE | 63 | - | - | - | - | - | - | - | - | - |
| TCGA-CZ-4853-11A-01D-1424-05 | Normal | MALE | 82 | - | - | - | - | - | - | - | - | - |
| TCGA-CZ-4856-11A-01D-1424-05 | Normal | FEMALE | 62 | - | - | - | - | - | - | - | - | - |
| TCGA-CZ-4859-11A-01D-1424-05 | Normal | FEMALE | 59 | - | - | - | - | - | - | - | - | - |
| TCGA-CZ-4863-11A-01D-1500-05 | Normal | FEMALE | 51 | - | - | - | - | - | - | - | - | - |
| TCGA-CZ-4864-11A-01D-1500-05 | Normal | MALE | 86 | - | - | - | - | - | - | - | - | - |
| TCGA-CZ-4865-11A-01D-1500-05 | Normal | FEMALE | 70 | - | - | - | - | - | - | - | - | - |
| TCGA-CZ-4866-11A-01D-1500-05 | Normal | FEMALE | 79 | - | - | - | - | - | - | - | - | - |
| TCGA-CZ-5451-11A-01D-1500-05 | Normal | MALE | 74 | - | - | - | - | - | - | - | - | - |
| TCGA-CZ-5452-11A-01D-1500-05 | Normal | MALE | 69 | - | - | - | - | - | - | - | - | - |
| TCGA-CZ-5453-11A-01D-1500-05 | Normal | MALE | 67 | - | - | - | - | - | - | - | - | - |
| TCGA-CZ-5454-11A-01D-1500-05 | Normal | MALE | 63 | - | - | - | - | - | - | - | - | - |
| TCGA-CZ-5455-11A-01D-1500-05 | Normal | MALE | 63 | - | - | - | - | - | - | - | - | - |
| TCGA-CZ-5456-11A-01D-1500-05 | Normal | MALE | 57 | - | - | - | - | - | - | - | - | - |
| TCGA-CZ-5457-11A-01D-1500-05 | Normal | MALE | 62 | - | - | - | - | - | - | - | - | - |
| TCGA-CZ-5458-11A-01D-1500-05 | Normal | MALE | 43 | - | - | - | - | - | - | - | - | - |
| TCGA-CZ-5459-11A-01D-1500-05 | Normal | MALE | 63 | - | - | - | - | - | - | - | - | - |
| TCGA-CZ-5460-11A-01D-1500-05 | Normal | MALE | 55 | - | - | - | - | - | - | - | - | - |
| TCGA-CZ-5461-11A-01D-1500-05 | Normal | MALE | 52 | - | - | - | - | - | - | - | - | - |
| TCGA-CZ-5462-11A-01D-1500-05 | Normal | MALE | 83 | - | - | - | - | - | - | - | - | - |
| TCGA-CZ-5463-11A-01D-1500-05 | Normal | MALE | 76 | - | - | - | - | - | - | - | - | - |
| TCGA-CZ-5464-11A-01D-1500-05 | Normal | MALE | 69 | - | - | - | - | - | - | - | - | - |
| TCGA-CZ-5465-11A-01D-1500-05 | Normal | FEMALE | 76 | - | - | - | - | - | - | - | - | - |
| TCGA-CZ-5466-11A-01D-1500-05 | Normal | MALE | 67 | - | - | - | - | - | - | - | - | - |
| TCGA-CZ-5467-11A-01D-1500-05 | Normal | FEMALE | 86 | - | - | - | - | - | - | - | - | - |
| TCGA-CZ-5468-11A-01D-1500-05 | Normal | MALE | 84 | - | - | - | - | - | - | - | - | - |
| TCGA-CZ-5469-11A-01D-1500-05 | Normal | MALE | 41 | - | - | - | - | - | - | - | - | - |
| TCGA-CZ-5470-11A-01D-1500-05 | Normal | FEMALE | 72 | - | - | - | - | - | - | - | - | - |
| GSM1429549 | Normal | FEMALE | 38 | - | - | - | - | - | - | - | - | - |
| GSM1429547 | Normal | FEMALE | 54 | - | - | - | - | - | - | - | - | - |
| GSM2060851 | Normal | Unknown | Unknown | - | - | - | - | - | - | - | - | - |
| GSM2085568 | Normal | FEMALE | Unknown | - | - | - | - | - | - | - | - | - |
| GSM2085576 | Normal | MALE | Unknown | - | - | - | - | - | - | - | - | - |
| GSM1322746 | Normal | Unknown | Unknown | - | - | - | - | - | - | - | - | - |
| GSM1702150 | Normal | Unknown | Unknown | - | - | - | - | - | - | - | - | - |
| GSM2085570 | Normal | FEMALE | Unknown | - | - | - | - | - | - | - | - | - |
| GSM1429557 | Normal | FEMALE | 42 | - | - | - | - | - | - | - | - | - |
| GSM1429534 | Normal | MALE | 41 | - | - | - | - | - | - | - | - | - |
| GSM1429532 | Normal | FEMALE | 12 | - | - | - | - | - | - | - | - | - |
| GSM2085575 | Normal | FEMALE | Unknown | - | - | - | - | - | - | - | - | - |
| GSM2085552 | Normal | MALE | Unknown | - | - | - | - | - | - | - | - | - |
| GSM1702198 | Normal | Unknown | Unknown | - | - | - | - | - | - | - | - | - |
| GSM1702118 | Normal | Unknown | Unknown | - | - | - | - | - | - | - | - | - |
| GSM1429528 | Normal | MALE | Unknown | - | - | - | - | - | - | - | - | - |
| GSM2085562 | Normal | FEMALE | Unknown | - | - | - | - | - | - | - | - | - |
| GSM1429561 | Normal | FEMALE | 20 | - | - | - | - | - | - | - | - | - |
| GSM2085566 | Normal | FEMALE | Unknown | - | - | - | - | - | - | - | - | - |
| GSM2085556 | Normal | FEMALE | Unknown | - | - | - | - | - | - | - | - | - |
| GSM1429562 | Normal | FEMALE | 20 | - | - | - | - | - | - | - | - | - |
| GSM1278511 | Normal | Unknown | Unknown | - | - | - | - | - | - | - | - | - |
| GSM2085554 | Normal | FEMALE | Unknown | - | - | - | - | - | - | - | - | - |
| GSM1702093 | Normal | Unknown | Unknown | - | - | - | - | - | - | - | - | - |
| GSM2085569 | Normal | MALE | Unknown | - | - | - | - | - | - | - | - | - |
| GSM2085567 | Normal | MALE | Unknown | - | - | - | - | - | - | - | - | - |
| GSM1429548 | Normal | FEMALE | 23 | - | - | - | - | - | - | - | - | - |
| GSM2085577 | Normal | FEMALE | Unknown | - | - | - | - | - | - | - | - | - |
| GSM2085572 | Normal | FEMALE | Unknown | - | - | - | - | - | - | - | - | - |
| GSM1429550 | Normal | FEMALE | 29 | - | - | - | - | - | - | - | - | - |
| GSM2085553 | Normal | MALE | Unknown | - | - | - | - | - | - | - | - | - |
| GSM1278510 | Normal | Unknown | Unknown | - | - | - | - | - | - | - | - | - |
| GSM1702168 | Normal | Unknown | Unknown | - | - | - | - | - | - | - | - | - |
| GSM2085558 | Normal | MALE | Unknown | - | - | - | - | - | - | - | - | - |
| GSM1278509 | Normal | Unknown | Unknown | - | - | - | - | - | - | - | - | - |
| GSM1702071 | Normal | Unknown | Unknown | - | - | - | - | - | - | - | - | - |
| GSM1322745 | Normal | Unknown | Unknown | - | - | - | - | - | - | - | - | - |
| GSM1429539 | Normal | FEMALE | 29 | - | - | - | - | - | - | - | - | - |
| GSM1429560 | Normal | FEMALE | 144 | - | - | - | - | - | - | - | - | - |
| GSM1429563 | Normal | FEMALE | 49 | - | - | - | - | - | - | - | - | - |
| GSM1702202 | Normal | Unknown | Unknown | - | - | - | - | - | - | - | - | - |
| GSM2085549 | Normal | MALE | Unknown | - | - | - | - | - | - | - | - | - |
| GSM1429553 | Normal | MALE | 47 | - | - | - | - | - | - | - | - | - |
| GSM2085550 | Normal | MALE | Unknown | - | - | - | - | - | - | - | - | - |
| GSM1429540 | Normal | MALE | 10 | - | - | - | - | - | - | - | - | - |
| GSM1429537 | Normal | MALE | 10 | - | - | - | - | - | - | - | - | - |
| GSM2060852 | Normal | Unknown | Unknown | - | - | - | - | - | - | - | - | - |
| GSM2085578 | Normal | FEMALE | Unknown | - | - | - | - | - | - | - | - | - |
| GSM2085574 | Normal | MALE | Unknown | - | - | - | - | - | - | - | - | - |
| GSM2085560 | Normal | FEMALE | Unknown | - | - | - | - | - | - | - | - | - |
| GSM1429558 | Normal | FEMALE | 31 | - | - | - | - | - | - | - | - | - |
| GSM2060853 | Normal | Unknown | Unknown | - | - | - | - | - | - | - | - | - |
| GSM1278513 | Normal | Unknown | Unknown | - | - | - | - | - | - | - | - | - |
| GSM2060855 | Normal | Unknown | Unknown | - | - | - | - | - | - | - | - | - |
| GSM1429554 | Normal | FEMALE | 28 | - | - | - | - | - | - | - | - | - |
| GSM1429559 | Normal | FEMALE | 12 | - | - | - | - | - | - | - | - | - |
| GSM1702241 | Normal | Unknown | Unknown | - | - | - | - | - | - | - | - | - |
| GSM1429535 | Normal | MALE | 21 | - | - | - | - | - | - | - | - | - |
| GSM1429552 | Normal | MALE | 62 | - | - | - | - | - | - | - | - | - |
| GSM1429544 | Normal | FEMALE | 10 | - | - | - | - | - | - | - | - | - |
| GSM2085563 | Normal | FEMALE | Unknown | - | - | - | - | - | - | - | - | - |
| GSM2085564 | Normal | MALE | Unknown | - | - | - | - | - | - | - | - | - |
| GSM2085571 | Normal | MALE | Unknown | - | - | - | - | - | - | - | - | - |
| GSM1702210 | Normal | Unknown | Unknown | - | - | - | - | - | - | - | - | - |
| GSM1060202 | Normal | MALE | Unknown | - | - | - | - | - | - | - | - | - |
| GSM1429551 | Normal | FEMALE | 16 | - | - | - | - | - | - | - | - | - |
| GSM1702219 | Normal | Unknown | Unknown | - | - | - | - | - | - | - | - | - |
| GSM1702200 | Normal | Unknown | Unknown | - | - | - | - | - | - | - | - | - |
| GSM1060201 | Normal | MALE | Unknown | - | - | - | - | - | - | - | - | - |
| GSM1278512 | Normal | Unknown | Unknown | - | - | - | - | - | - | - | - | - |
| GSM2060854 | Normal | Unknown | Unknown | - | - | - | - | - | - | - | - | - |
| GSM1429530 | Normal | FEMALE | 33 | - | - | - | - | - | - | - | - | - |
| GSM1702143 | Normal | Unknown | Unknown | - | - | - | - | - | - | - | - | - |
| GSM1702083 | Normal | Unknown | Unknown | - | - | - | - | - | - | - | - | - |
| GSM1429538 | Normal | MALE | 47 | - | - | - | - | - | - | - | - | - |
| GSM1429542 | Normal | MALE | 44 | - | - | - | - | - | - | - | - | - |
| GSM1429529 | Normal | FEMALE | 39 | - | - | - | - | - | - | - | - | - |
| GSM1429543 | Normal | FEMALE | 71 | - | - | - | - | - | - | - | - | - |
| GSM1702074 | Normal | Unknown | Unknown | - | - | - | - | - | - | - | - | - |
| GSM1278508 | Normal | Unknown | Unknown | - | - | - | - | - | - | - | - | - |
| GSM1429545 | Normal | MALE | 42 | - | - | - | - | - | - | - | - | - |
| GSM1702231 | Normal | Unknown | Unknown | - | - | - | - | - | - | - | - | - |
| GSM2085557 | Normal | MALE | Unknown | - | - | - | - | - | - | - | - | - |
| GSM1429536 | Normal | MALE | 44 | - | - | - | - | - | - | - | - | - |
| GSM1429533 | Normal | MALE | 10 | - | - | - | - | - | - | - | - | - |
| GSM1429541 | Normal | FEMALE | 11 | - | - | - | - | - | - | - | - | - |
| GSM1429531 | Normal | FEMALE | 58 | - | - | - | - | - | - | - | - | - |
| GSM2085555 | Normal | MALE | Unknown | - | - | - | - | - | - | - | - | - |
| GSM2085573 | Normal | FEMALE | Unknown | - | - | - | - | - | - | - | - | - |
| GSM1429546 | Normal | MALE | 42 | - | - | - | - | - | - | - | - | - |
| GSM2085565 | Normal | FEMALE | Unknown | - | - | - | - | - | - | - | - | - |
| GSM1702099 | Normal | Unknown | Unknown | - | - | - | - | - | - | - | - | - |
| GSM2085579 | Normal | FEMALE | Unknown | - | - | - | - | - | - | - | - | - |
| GSM1429555 | Normal | MALE | 39 | - | - | - | - | - | - | - | - | - |
| GSM2085559 | Normal | MALE | Unknown | - | - | - | - | - | - | - | - | - |
| GSM1429556 | Normal | MALE | 49 | - | - | - | - | - | - | - | - | - |
