# Supplementary material for: Multi-omics analysis reveals epithelial-mesenchymal transition-related gene FOXM1 as a novel prognostic biomarker in clear cell renal carcinoma
Source: Aging (Albany NY). 2019 Nov 19;11(22):10316–37. doi: 10.18632/aging.102459 (PMC6914426; doi:10.18632/aging.102459)
Supplement: Supplementary Code [file aging-11-102459-s002..docx]

**Supplementary Code** for manuscript “Multi-omics analysis reveals Epithelial-Mesenchymal Transition-related gene FOXM1 as a novel prognostic biomarker in clear cell renal carcinoma”

i) The code (Perl language) used to perform text mining in order to extract raw EMT related genes/proteins from Pubmed query xml results:

#!/usr/bin/env perl -w

use strict;

use warnings;

use Storable;

use Data::Dumper;

=head1

This script was to extract EMT related genes/proteins from Pubmed xml search result.

In the process, user need to manurally run geniatagger and Enju softwares use intermediate outputs. And then, run remining code.

The final output 'Pubmed_QueryArticles_info_AbstractOrConclusion_tagged_refined2.GeneList' contains the raw genes/proteins extract

from literature abstract. Additional annotaiton and fitering are required to obtain standard gene symbols or other kinds of ids.

=cut

my $pubmed_xml = "./pubmed_result.xml";

my $InfoFromLiterature;

if(-e "./Dumper_Pubmed_QueryArticles_info"){

$InfoFromLiterature = main::retrieve("./Dumper_Pubmed_QueryArticles_info");

}else{

open XML,"$pubmed_xml" or die $!;

my @EachQuery;

my ($start,$count,$PMID) = (0,0,"");

while(<XML>){

if(/<PubmedArticle>/){

$start = 1;

}elsif(/<\/PubmedArticle>/){

push @EachQuery,$_;

$count++;

$InfoFromLiterature->{"PMID$PMID"} = &RetrieveInfoFromLiterature(\@EachQuery);

$PMID = "";

@EachQuery = ();

$start = 0;

print "$count articles Processed.\n" if($count % 10000 == 0);

}else{

$PMID = $1 if(/>(\d+)<\/PMID/);

}

push @EachQuery,$_ if($start == 1);

#print "@EachQuery\n";

#print Dumper $InfoFromLiterature if($count >=1);

#last if($count >=1);

}

close XML;

main::store $InfoFromLiterature,"./Dumper_Pubmed_QueryArticles_info";

&DumperToFile("./DataStructure_Pubmed_QueryArticles_info",$InfoFromLiterature);

}

unless(-e "./Pubmed_QueryArticles_info_AbstractOrConclusion_tagged_refined2"){

open PRE,">./Pubmed_QueryArticles_info_AbstractOrConclusion2" or die $!;

my @Reference;

for my $PMID (sort keys %$InfoFromLiterature){

my $title = $InfoFromLiterature->{$PMID}->{'PaperTitle'};

my $abstract = $InfoFromLiterature->{$PMID}->{'Abstract'};

my $conclusion = $InfoFromLiterature->{$PMID}->{'Conclusion'};

my $info = join ";",($title,$abstract,$conclusion);

next unless($info=~/\sEMT|\sVIM|(?i:epithelial.?(?:to)?.?mesenchymal.transition)|(?i:E-cadherin|N-cadherin|\sCDH1|\sCDH2|vimentin|migration|invasion)/);

my $Ref_AMA = "$InfoFromLiterature->{$PMID}->{'Author1st'} $InfoFromLiterature->{$PMID}->{'PaperTitle'} $InfoFromLiterature->{$PMID}->{'JornalName'} ";

$Ref_AMA .= "$InfoFromLiterature->{$PMID}->{'PubYear'};$InfoFromLiterature->{$PMID}->{'Vol'}($InfoFromLiterature->{$PMID}->{'Issue'}):";

$Ref_AMA .= "$InfoFromLiterature->{$PMID}->{'Page'}. doi:$InfoFromLiterature->{$PMID}->{'Doi'}.";

push @Reference,"[$PMID]$Ref_AMA";

print PRE "PMIDPMID\n";

$title =~s/\n//g;

if($conclusion=~/\w/){

my @sentences = split /\. /,$conclusion;

print PRE "$_\n\n" for(@sentences);

print PRE "$title\n\n\n";

}else{

my @sentences = split /\. /,$abstract;

print PRE "$sentences[$_]\n\n" for(3..$#sentences);

print PRE "$title\n\n\n";

}

}

close PRE;

open REF,">./Pubmed_QueryArticles_info_AbstractOrConclusion_tagged_refined2" or die $!;

#`./geniatagger Pubmed_QueryArticles_info_AbstractOrConclusion2 > Pubmed_QueryArticles_info_AbstractOrConclusion_tagged2.geniatagger`;

#`./enju-master/run-super <Pubmed_QueryArticles_info_AbstractOrConclusion2 > Pubmed_QueryArticles_info_AbstractOrConclusion_tagged2.Enju`;

open TAG,"./Pubmed_QueryArticles_info_AbstractOrConclusion_tagged2.geniatagger" or die $!;

my ($HaveProtein,$Pros);

my ($empty,$paper,$sentence,$status) = (0,0,0,0);

my (@Bprotes,@Bpro);

while(<TAG>){

next if(/^loading/);

chomp;

if(/PMIDPMID/){

$paper++;

$empty=0;

$sentence =0;

}else{

if((split /\s/)>=5){

$empty = 1;

}else{

$empty++;

}

if($empty==2){

$sentence++;

my @s;

push @s,$_ for(@Bprotes);

push @{$HaveProtein->[$paper-1]},[$status,\@s];

$status = 0;

@Bprotes = ();

}

if(/B-protein/){

unless(/E-cadherin|N-cadherin|twist|snail|vimentin|CDH1|CDH2|ERK|antibodies/i){

$status = 1;

my $pro = (split /\s/)[0];

$pro =~s/\[|\]|\(|\)|\+|\?//g;

push @Bprotes,$pro unless(grep {$pro eq $_} @Bprotes);

}

}

}

}

close TAG;

open TAG2,"./Pubmed_QueryArticles_info_AbstractOrConclusion_tagged2.Enju" or die $!;

($paper,$sentence,$empty) = (0,0,0);

my @info;

while(<TAG2>){

chomp;

if(/PMIDPMID/){

$paper++;

my $ref=shift @Reference;

print REF "\n" unless($.==1);

print REF "$ref\n";

my $PMID = $1 if($ref=~/(PMID\d+)/);

my $Keywords = join ",",@{$InfoFromLiterature->{$PMID}->{'Keywords'}};

print REF "[KEYWORDS]: $Keywords\n";

$empty =0;

$sentence = 0;

}else{

my @line =split /\s+/;

if(@line>=7){

$empty = 1;

}else{

$empty++;

}

if($empty==2){

$sentence++;

my ($status,$Bprotein) = ($HaveProtein->[$paper-1]->[$sentence-1]->[0],$HaveProtein->[$paper-1]->[$sentence-1]->[1]);

if($status == 0){@info = ();next}

my $event = &EventMining(\@info,$Bprotein);

print REF "[INFO]:$event\n";

@info=();

}

push @info,[$line[0],$line[1]] if(@line>=7);

}

}

close REF;

close TAG2;

}

unless(-e "./Pubmed_QueryArticles_info_AbstractOrConclusion_tagged_refined2.GeneList"){

open REFINED,"./Pubmed_QueryArticles_info_AbstractOrConclusion_tagged_refined2" or die $!;

open LIST,">./Pubmed_QueryArticles_info_AbstractOrConclusion_tagged_refined2.GeneList" or die $!;

my @List;

while(<REFINED>){

chomp;

if(/^\[INFO\]/){

$_=~s/^\[INFO\]://g;

my @genes = split /\s+/;

for my $gene (@genes){

next if(/^[0-9]|^-|-$|\w \w/);

push @List,$gene unless(grep {$gene =~ /$_/} @List);

}

}

}

close REFINED;

print LIST "$_\n" for(@List);

close LIST;

}

sub EventMining {

#EventMining for each sentence.

my ($info,$Bproteins) = (shift,shift);

my (@words,@POS,@loc_Bproteins);

for my $index(0..$#$info){

my @info2 = @{$info->[$index]};

push @words,$info2[0];

push @POS,$info2[1];

if(grep {$info2[0]=~/$_/} @$Bproteins){

push @loc_Bproteins,$index;

}

}

my $sentence = join " ",@words;

my $loc = $loc_Bproteins[0];

return "" if(!defined $loc);

my $ss = join " ",@$Bproteins;

if($loc == 0){

if(@loc_Bproteins == 1){

return "" if($words[0] =~/^EMT$/);

return "" unless(grep {$_=~/VB/} @POS);

return "" unless($sentence=~/EMT|\sVIM|(?i:epithelial.?(?:to)?.?mesenchymal.transition)|(?i:mesenchymal.?(?:to)?.?epithelial.transition)|(?i:E-cadherin|N-cadherin|\sCDH1|\sCDH2|vimentin|migration|invasion|metastasis|invasive|tumor formation)/);

return $ss;

}else{

return "" unless($sentence=~/induce|increase|decrease|suppress|require|acquire|modulate|inhibit|conduct|bind(?:ing|s)?.to|regulate|enhance|mediate|implicated in|activat|effector|silenc|reduce|degradation|active|support|promote/);

return $ss;

}

}else{

return "" if($sentence=~/survival|not EMT/);

return "" unless($sentence=~/induce|increase|decrease|suppress|require|acquire|modulate|inhibit|conduct|bind(?:ing|s)?.to|regulate|enhance|mediate|implicated in|activat|effector|silenc|reduce|degradation|active|support|promote/);

return $ss;

}

}

sub RetrieveInfoFromLiterature {

my $info = shift;

my $InfoThisPaper;

my ($PMID,$JornalName,$Author1st,$PaperTitle,$PubYear,$PubMonth,$PubDay,$Page,$Vol,$Issue,$Doi,$Background,$Methods,$Results,$Conclusion,$Abstract)=("","","","","","","","","","","","","","","","");

my $Keywords = [];

my ($lastname,$initials);

my $pubdate = 0;

for (@$info){

$PMID = $1 if(/<PMID.+>(\d+)<\/PMID>/);

$JornalName = $1 if(/<ISOAbbreviation>(.+)<\/ISOAbbreviation>/);

$lastname = $1 if(/<LastName>(.+)<\/LastName>/);

$initials = $1 if(/<Initials>(.+)<\/Initials>/);

$Author1st = "$lastname $initials, et al." if((defined $lastname)&&(defined $initials)&&($Author1st eq ""));

$PaperTitle = $1 if(/<ArticleTitle>(.+)<\/ArticleTitle>/);

$pubdate = 1 if(/<PubDate>/);

$pubdate = 0 if(/<\/PubDate>/);

$PubYear = $1 if(($pubdate == 1)&&(/<Year>(.+)<\/Year>/));

$PubMonth = $1 if(($pubdate == 1)&&(/<Month>(.+)<\/Month>/));

$PubDay = $1 if(($pubdate == 1)&&(/<Day>(.+)<\/Day>/));

$Page = $1 if(/<MedlinePgn>(.+)<\/MedlinePgn>/);

$Vol = $1 if(/<Volume>(.+)<\/Volume>/);

$Issue = $1 if(/<Issue>(.+)<\/Issue>/);

$Doi = $1 if(/<ELocationID EIdType="doi".+>(.+)<\/ELocationID>/);

push @$Keywords,$1 if(/<Keyword .+>(.+)<\/Keyword>/);

if((/AbstractText/)&&(/BACKGROUND|AIMS|OBJECTIVE/)){

$Background = $1 if(/>(.+)</);

}elsif((/AbstractText/)&&(/METHODS/)){

$Methods = $1 if(/>(.+)</);

}elsif((/AbstractText/)&&(/RESULTS/)){

$Results = $1 if(/>(.+)</);

}elsif((/AbstractText/)&&(/CONCLUSION/)){

$Conclusion = $1 if(/>(.+)</);

}elsif(/AbstractText/){

$Abstract = $1 if(/>(.+)</);

}

}

$InfoThisPaper = {

'PMID' => $PMID,

'JornalName' => $JornalName,

'Author1st' => $Author1st,

'PaperTitle' => $PaperTitle,

'PubYear' => $PubYear,

'PubMonth' => $PubMonth,

'PubDay' => $PubDay,

'Page' => $Page,

'Vol' => $Vol,

'Issue' => $Issue,

'Doi' => $Doi,

'Background' => $Background,

'Methods' => $Methods,

'Results' => $Results,

'Conclusion' => $Conclusion,

'Abstract' => $Abstract,

'Keywords' => $Keywords,

};

return $InfoThisPaper;

}

sub DumperToFile {

my ($outfile,$reference) = (shift,shift);

open B, ">&STDOUT";

open STDOUT, ">$outfile";

print main::Dumper $reference;

open STDOUT, ">&B";

close B;

}

ii) The code (R language) for unsupervised clustering analysis based on expression levels of 756 EMT-related genes using multiple R packages:

library(pheatmap)

library(ggplot2)

library("ConsensusClusterPlus")

require(graphics)

expr <- read.csv("KIRC_gene_log2CPM_expression_selected_heatmap.txt",sep="\t",header=T,check.names=F)

expr1 <- as.matrix(expr[c(-0,-1)])

myrows <-expr$Gene

myrows <-as.character(myrows)

rownames(expr1) <- myrows

expr2 = as.matrix(scale(expr1))[1:nrow(expr1),1:ncol(expr1)]

#expr3 = RefineRanges(expr2,-3,3)

clin = read.csv("KIRC_gene_log2CPM_expression_selected_heatmap_clinical.txt",sep="\t",header=T,check.names=F)

#clin ->: 1.all normal = NX/MX/TX/StageX/GX 2.all "Unknown" = NX/MX/TX/StageX/GX 3. T stage refined to T1/T2/T3/T4

#T refine

clin$AJCC_TUMOR_PATHOLOGIC_PT = factor(substr(clin$AJCC_TUMOR_PATHOLOGIC_PT,1,2))

tmp = as.character(clin$AJCC_TUMOR_PATHOLOGIC_PT)

tmp[c(532:603)] = "Unknown"

clin$AJCC_TUMOR_PATHOLOGIC_PT = factor(tmp)

#N refine

tmp = as.character(clin$AJCC_NODES_PATHOLOGIC_PN)

tmp = gsub("NX","Unknown",tmp)

tmp[c(532:603)] = "Unknown"

clin$AJCC_NODES_PATHOLOGIC_PN = factor(tmp,levels=c("N0","N1","Unknown"))

#M refine

tmp = as.character(clin$AJCC_METASTASIS_PATHOLOGIC_PM)

tmp = gsub("MX","Unknown",tmp)

tmp[c(532:603)] = "Unknown"

clin$AJCC_METASTASIS_PATHOLOGIC_PM = factor(tmp,levels=c("M0","M1","Unknown"))

#Stage refine

clin$AJCC_PATHOLOGIC_TUMOR_STAGE = factor(gsub(" ","",as.character(clin$AJCC_PATHOLOGIC_TUMOR_STAGE)))

tmp = as.character(clin$AJCC_PATHOLOGIC_TUMOR_STAGE)

tmp[c(532:603)] = "Unknown"

clin$AJCC_PATHOLOGIC_TUMOR_STAGE = factor(tmp,levels=c("StageI","StageII","StageIII","StageIV","Unknown"))

#Grade refine

tmp = as.character(clin$GRADE)

tmp = gsub("GX","Unknown",tmp)

tmp[c(532:603)] = "Unknown"

clin$GRADE = factor(tmp,levels=c("G1","G2","G3","G4","Unknown"))

#gender

tmp = as.character(clin$GENDER)

tmp[c(532:603)] = "Unknown"

clin$GENDER = tmp

#cutree_rows= 5,cutree_cols=2,

ann_col = data.frame(

Group = factor(rep(c("Cancer", "Normal"), c(531, 72))),

PT = clin$AJCC_TUMOR_PATHOLOGIC_PT,

PN = clin$AJCC_NODES_PATHOLOGIC_PN,

PM = clin$AJCC_METASTASIS_PATHOLOGIC_PM,

Stage=clin$AJCC_PATHOLOGIC_TUMOR_STAGE,

Grade=clin$GRADE,

row.names =colnames(expr2)

)

DiffExpr = read.csv("EMT_related_gene_ByLiterature_ExprStatus.Allgroups.txt",sep="\t",header=T,check.names=F,stringsAsFactors=F)

DiffExpr = DiffExpr[match(rownames(expr2),DiffExpr$Gene),]

DiffExpr[DiffExpr == 1] = "Up"

DiffExpr[DiffExpr == -1] = "Down"

DiffExpr[DiffExpr == 0] = "NonChange"

ann_row = data.frame(

CancerVsNormal = as.factor(DiffExpr$CancerVsNormal),

LowStageVsNormal = as.factor(DiffExpr$LowStageVsNormal),

HighStageVsLowStage = as.factor(DiffExpr$LowStageVsHighStage),

LowGradeVsNormal = as.factor(DiffExpr$LowGradeVsNormal),

HighGradeVsLowGrade = as.factor(DiffExpr$LowGradeVsHighGrade),

NonLymphMeta.VsNormal = as.factor(DiffExpr$NoLymphMetastasisVsNormal),

LymphMeta.VsNonLymphMeta. = as.factor(DiffExpr$LymphMetastasisVsNoLymphMetastasis),

ProgressionFreeVsNormal = as.factor(DiffExpr$DiseaseFreeVsNormal),

RecurredVsProgressionFree = as.factor(DiffExpr$RecurrenceVsDiseaseFree),

NoDistantMeta.VsNormal = as.factor(DiffExpr$NoDistantMetastasisVsNormal),

DistantMeta.VsNoDistantMeta. = as.factor(DiffExpr$DistantMetastasisVsNoDistantMetastasis),

row.names = rownames(expr2)

)

#levels = c("Baseline","Down","Up")

ann_colors = list(

Group = c(Cancer="red", Normal="green"),

PT = c(T1 = "#7FFF00", T2 = "#09ec64",T3= "#B03060",T4= "#7D5021",Unknown = "#F8F8FF"),

PN = c(N0 = "#09ec64", N1 = "#8A360F",Unknown = "#F8F8FF"),

PM = c(M0 = "#09ec64", M1 = "#8A360F",Unknown = "#F8F8FF"),

Stage = c(StageI = "#87CEFA",StageII = "#0965EC",StageIII = "#FE7b82",StageIV = "#F50041",Unknown = "#F8F8FF"),

Grade = c(G1 = "#00FFFF",G2 = "#008B8B",G3 = "#FF7D40", G4 = "#8A360F",Unknown = "#F8F8FF"),

CancerVsNormal = c(Up = "#F50041", Down = "#0706EA", NonChange = "#F8F8FF"),

LowStageVsNormal = c(Up = "#F50041", Down = "#0706EA", NonChange = "#F8F8FF"),

HighStageVsLowStage = c(Up = "#F50041", Down = "#0706EA", NonChange = "#F8F8FF"),

LowGradeVsNormal = c(Up = "#F50041", Down = "#0706EA", NonChange = "#F8F8FF"),

HighGradeVsLowGrade = c(Up = "#F50041", Down = "#0706EA", NonChange = "#F8F8FF"),

NonLymphMeta.VsNormal = c(Up = "#F50041", Down = "#0706EA", NonChange = "#F8F8FF"),

LymphMeta.VsNonLymphMeta. = c(Up = "#F50041", Down = "#0706EA", NonChange = "#F8F8FF"),

ProgressionFreeVsNormal = c(Up = "#F50041", Down = "#0706EA", NonChange = "#F8F8FF"),

RecurredVsProgressionFree = c(Up = "#F50041", Down = "#0706EA", NonChange = "#F8F8FF"),

NoDistantMeta.VsNormal = c(Up = "#F50041", Down = "#0706EA", NonChange = "#F8F8FF"),

DistantMeta.VsNoDistantMeta. = c(Up = "#F50041", Down = "#0706EA", NonChange = "#F8F8FF")

)

pdf("KIRC_gene_expr_pheatmap.pdf",family="Times",width =18,height=14)

clustering = pheatmap(expr2,col=colorRampPalette(c("blue","white","red"))(256),scale="row",border_color=NA,

annotation_col = ann_col, annotation_row = ann_row,fontsize=17,

annotation_colors = ann_colors,

cutree_rows= 7,cutree_cols= 4,drop_levels = FALSE,

legend_breaks=seq(-10,10,by=10),legend_labels=c("Low","","High"),

clustering_method = "ward.D",

labels_row = c(rep("",112),"gC4(123)",rep("",115),"gC2(181)",rep("",118),"gC3(155)",rep("",95),"gC1(95)",rep("",55),"gC6(35)",rep("",3),"gC5(63)",rep("",52),"gC7(52)",rep("",147)),fontsize_row =30,

labels_col = c(rep("",114),"sC4(220)",rep("",303),"sC2(244)",rep("",96),"sC3(68)",rep("",38),"sC1(71)",rep("",48)),fontsize_col =30,

main = "Clustering of mRNA(MRN)"

)

dev.off()

#k=7#c(rep("",112),"gC4(123)",rep("",115),"gC2(181)",rep("",118),"gC3(155)",rep("",95),"gC1(95)",rep("",55),"gC6(35)",rep("",3),"gC5(63)",rep("",52),"gC7(52)",rep("",147))

#k=4#c(rep("",114),"sC4(220)",rep("",303),"sC2(244)",rep("",96),"sC3(68)",rep("",38),"sC1(71)",rep("",48))

cutree_samples = cutree(clustering$tree_col,4)

sC1 = cutree_samples[cutree_samples == 4] #71

sC2 = cutree_samples[cutree_samples == 1] #244

sC3 = cutree_samples[cutree_samples == 3] #68

sC4 = cutree_samples[cutree_samples == 2] #220

cutree_genes = cutree(clustering$tree_row,7)

gC1 = cutree_genes[cutree_genes == 3] #95

gC2 = cutree_genes[cutree_genes == 2] #181

gC3 = cutree_genes[cutree_genes == 1] #155

gC4 = cutree_genes[cutree_genes == 5] #123

gC5 = cutree_genes[cutree_genes == 6] #63

gC6 = cutree_genes[cutree_genes == 7] #35

gC7 = cutree_genes[cutree_genes == 4] #52

#determined sample cluster K by ConcensusClusterPlus # k = 4

mydata1 = expr2

mads=apply(mydata1,1,mad)

mydata1=mydata1[rev(order(mads)),]

mydata1 = sweep(mydata1,1, apply(mydata1,1,median,na.rm=T))

title="C:/Users/songjing/Desktop/cluster"

colors=c("#000000","#0f0303","#180505","#250808","#420d0d","#4b1111",

"#671313","#7e1818","#8B1A1A","#961717","#8B0000")

results = ConsensusClusterPlus(mydata1,maxK=10,reps=1000,pItem=0.95,pFeature=1,

title=title,clusterAlg="hc",distance="euclidean",tmyPal=colors,innerLinkage="ward.D",

plot="pdf")

#determined gene cluster K by ConcensusClusterPlus # k = 7

results = ConsensusClusterPlus(t(mydata1),maxK=10,reps=1000,pItem=0.95,pFeature=1,

title=title,clusterAlg="hc",distance="euclidean",tmyPal=colors,innerLinkage="ward.D",

plot="pdf")

iii) The code (Perl language) of our custom Perl functions to perform batch effect evaluation using MBatch v1.0 software:

#!/usr/bin/env perl -w

BEGIN {$| = 1} #no cache permitted for print

use strict;

use warnings;

use Getopt::Long;

use Cwd qw(abs_path);

use File::Basename;

use File::Path;

use Data::Dumper qw(Dumper);

use Storable;

use Statistics::R;

use POSIX ":sys_wait_h";

use threads;

use threads::shared;

use XML::LibXML;

use Term::ANSIColor;

use LWP::Simple;

use Thread::Semaphore;

use List::Util;

use List::MoreUtils qw{duplicates};

use ICC::Profile;

use Math::Matrix;

sub BatchVariableEvaluation {

#Use R version 3.0.2

my ($project,$analyte) = (shift,shift);

my ($WorkDir,$BatchInfoAndMatrixDir,$MatchIDs,$SampleSize,$dataset);

if(($project eq 'TTG')&&($analyte eq 'Expression')){

$WorkDir = "$Variables::dir/Processing/$Variables::Res_TTG";

$BatchInfoAndMatrixDir = "$WorkDir/$Variables::TTG_tmpname";

$MatchIDs = main::retrieve("$WorkDir/Dumper_TCGA_GTEX_match_ids");

$SampleSize = main::retrieve("$Variables::TTG_sample_size_dumper");

$dataset = "TCGA-GTEX-rsem-count";

print "Evaluating Batch variables of TCGA-GTEX-rsem-count of each cancer type for 7 variables...\n";

}elsif(($project eq 'GDC')&&($analyte eq 'Expression')){

$WorkDir = "$Variables::dir/Processing/$Variables::Res_GDC";

$BatchInfoAndMatrixDir = "$WorkDir/$Variables::GDC_tmpname";

$MatchIDs =main::retrieve("$WorkDir/Dumper_GDC_sample_ids_info");

$SampleSize = main::retrieve("$Variables::GDC_sample_size_dumper");

$dataset = "GDC-htseq-count";

print "Evaluating Batch variables of GDC-htseq-count of each cancer type for 7 variables...\n";

}elsif(($project eq 'GEO')&&($analyte eq 'Expression')){

$WorkDir = "$Variables::dir/Processing/$Variables::Res_GEO";

$BatchInfoAndMatrixDir = "$WorkDir/$Variables::GEO_tmpname";

$MatchIDs = main::retrieve("$WorkDir/Dumper_GEOexpr_MatchIDs");

$SampleSize = main::retrieve("$Variables::GEO_sample_size_dumper");

$dataset = "GEO-microarray-expression";

print "Evaluating Batch variables of GEO-microarray-expression of each cancer type for 7 variables...\n";

}elsif(($project eq 'GDC-GEO')&&($analyte eq 'Methylation')){

$WorkDir = "$Variables::dir/Processing/$Variables::Res_GEO";

$BatchInfoAndMatrixDir = "$WorkDir/$Variables::ALL_methy_tmpname";

$MatchIDs = main::retrieve("$WorkDir/Dumper_AllMethy_MatchIDs");

$SampleSize = main::retrieve("$Variables::ALL_methy_SampleSize");

$dataset = "GDC-GEO-methylation";

print "Evaluating Batch variables of GDC-GEO-methylation of each cancer type for 7 variables...\n";

}

mkdir "$WorkDir" unless (-d "$WorkDir");

for my $cancer (keys %$MatchIDs){

next if(!defined $MatchIDs->{$cancer}->[1]);

&BatchVarEvalEachCancer($project,$cancer,$WorkDir,$analyte,$dataset,$BatchInfoAndMatrixDir,$SampleSize);

}

sub BatchVarEvalEachCancer {

my ($project,$cancer,$WorkDir,$analyte,$dataset,$BatchInfoAndMatrixDir,$SampleSize) = @{$_[0]};

my $MBatch_input_dir = "$BatchInfoAndMatrixDir/BatchVariableEvaluation/2018_7_26_1729/$cancer/$analyte/$dataset";

main::mkpath "$MBatch_input_dir" unless (-d "$MBatch_input_dir");

my $ExpSubmatrix;

$ExpSubmatrix = "$BatchInfoAndMatrixDir/$cancer\_gene_count_expression.txt" if(($dataset eq 'TCGA-GTEX-rsem-count')||($dataset eq 'GDC-htseq-count'));

$ExpSubmatrix = "$BatchInfoAndMatrixDir/$cancer\_gene_fpkm_QuantileNormalized.gz" if($dataset eq 'GDC-fpkm-expression');

$ExpSubmatrix = "$BatchInfoAndMatrixDir/$cancer\_gene_microarray_QuantileNormalized.gz" if($dataset eq 'GEO-microarray-expression');

$ExpSubmatrix = "$BatchInfoAndMatrixDir/$cancer\_CpG_MValues_QuantileNormalized.gz" if($dataset eq 'GDC-GEO-methylation');

$ExpSubmatrix = "$BatchInfoAndMatrixDir/$cancer\_gene_count.txt" if($dataset eq 'GDC-miRNA-expression');

my ($TumorSampleSize,$NormalSampleSize);

for (@$SampleSize){

($TumorSampleSize,$NormalSampleSize) = ($_->[1],$_->[2]) if($_->[0] eq $cancer);

}

my $TotalSizePlus1 = $TumorSampleSize + $NormalSampleSize + 1;

if($ExpSubmatrix =~/.gz$/){

open INFH,"zcat $ExpSubmatrix|" or die $!;

}else{

open INFH,"$ExpSubmatrix" or die $!;

}

open OUTFH,">$MBatch_input_dir/bea_input_cleansed.tsv" or die $!;

print OUTFH "sample\tpoint\tvalue\n";

my @sample_ids;

while(<INFH>){

chomp;

if($.==1){push @sample_ids,split /\t/;next}

push my @values,split /\t/;

for my $index (1..$#values){

print OUTFH "$sample_ids[$index]\t$values[0]\t$values[$index]\n";

}

}

close INFH;

close OUTFH;

my %BatchTypeLocation = (

'TSS' => '2',

'PlateID' => '3',

'CGCCandGSC' => '4',

'ShipDate' => '5',

'Instrument' => '6',

'BCR' => '8',

'BatchID' => '9',

);

for my $BatchType (keys %BatchTypeLocation){

my $BatchInfoSubMatrix;

$BatchInfoSubMatrix = "$BatchInfoAndMatrixDir/$cancer\_rnaseq_BatchInfo.txt" if(($dataset eq 'TCGA-GTEX-rsem-count')||($dataset eq 'GDC-htseq-count'));

$BatchInfoSubMatrix = "$BatchInfoAndMatrixDir/$cancer\_rnaseq_BatchInfo.txt" if(($dataset eq 'GDC-fpkm-expression')||($dataset eq 'GDC-miRNA-expression'));

$BatchInfoSubMatrix = "$BatchInfoAndMatrixDir/$cancer\_microarray_BatchInfo.txt" if($dataset eq 'GEO-microarray-expression');

$BatchInfoSubMatrix = "$BatchInfoAndMatrixDir/$cancer\_methylation_BatchInfo.txt" if($dataset eq 'GDC-GEO-methylation');

open INFH,"$BatchInfoSubMatrix" or die $!;

open OUTFH,">$MBatch_input_dir/bea_batch_$BatchType.tsv" or die $!;

print OUTFH "sample\tbatch\n";

while(<INFH>){

chomp;

next if($.==1);

my @line = split /\t/;

print OUTFH "$line[0]\t$line[$BatchTypeLocation{$BatchType}]\n";

}

close INFH;

close OUTFH;

}

open BVEV,">./$cancer\_$project\_BatchVariableEvaluation.R" or die $!;

print BVEV "###Batch Variable Evaluation using MBatch v1.0.0###\n";

print BVEV "library(\"MBatch\")\nlibrary(\"Cairo\")\nlibrary(\"gtools\")\n";

print BVEV "dataDir <- file.path(\"$MBatch_input_dir\")\nfile.exists(dataDir)\n";

print BVEV "myTitle <- \"2018_7_26_1729/$cancer/$analyte/$dataset\"\n";

print BVEV "myGeneDataFile <- file.path(dataDir, \"bea_input_cleansed.tsv\")\n";

print BVEV "myListOfBatchFiles <- c(\n";

print BVEV " file.path(dataDir, \"bea_batch_TSS.tsv\"),\n";

print BVEV " file.path(dataDir, \"bea_batch_PlateID.tsv\"),\n";

print BVEV " file.path(dataDir, \"bea_batch_CGCCandGSC.tsv\"),\n";

print BVEV " file.path(dataDir, \"bea_batch_ShipDate.tsv\"),\n";

print BVEV " file.path(dataDir, \"bea_batch_Instrument.tsv\"),\n";

print BVEV " file.path(dataDir, \"bea_batch_BCR.tsv\"),\n";

print BVEV " file.path(dataDir, \"bea_batch_BatchID.tsv\"))\n";

print BVEV "myListOfBatchTypes <- c(\n";

print BVEV " \"TSS\",\n";

print BVEV " \"PlateID\",\n";

print BVEV " \"CGCCandGSC\",\n";

print BVEV " \"ShipDate\",\n";

print BVEV " \"Instrument\",\n";

print BVEV " \"BCR\",\n";

print BVEV " \"BatchID\")\n";

print BVEV "myOutputPath <- file.path(\"$MBatch_input_dir\",\"MBatchEvalOut\")\n";

print BVEV "if(!file.exists(myOutputPath)){dir.create(myOutputPath)}\n";

print BVEV "myInputOutputObject <- new(\"InputOutput\",\n";

print BVEV " myGeneDataFile,\n";

print BVEV " myListOfBatchFiles,\n";

print BVEV " myListOfBatchTypes,\n";

print BVEV " myTitle, myOutputPath,\n";

print BVEV " theDataFileFormat = \"BEA-Columns\",\n";

print BVEV " theSeperator=\"\\t\",\n";

print BVEV " theQuoteChar=\"\")\n";

print BVEV "myDataFiltersObject <- new(\"DataFilters\",\n";

print BVEV " theMinIqr = 0,\n";

print BVEV " theMinSd = 0,\n";

print BVEV " theMinMad = 0,\n";

print BVEV " theListOfBatchesToRemove = c(\"unknown\"))\n";

print BVEV "myHCObject <- new(\"HC\",theDoHCFlag = TRUE)\n";

print BVEV "isTrendBatch <- function(theBatchTypeName, theListOfBatchIds){return(is.element(theBatchTypeName, c(\"ShipDate\")))}\n";

print BVEV "myPCAObject <- new(\"PCA\",\n";

print BVEV " theIsPcaTrendFunction=isTrendBatch,\n";

print BVEV " theDoCentroidsMtoMFlag=TRUE,\n";

print BVEV " theDoPlainMtoMFlag=TRUE,\n";

print BVEV " theDoCentroidsOtoMFlag=TRUE,\n";

print BVEV " theDoPlainOtoMFlag=TRUE,\n";

print BVEV " theDoDSCFlag=TRUE,\n";

print BVEV " theDoSampleLocatorFlag=TRUE,\n";

print BVEV " theListOfComponentsToPlot=c(1, 2, 1, 3, 2, 3),\n";

print BVEV " theListForDoCentroidDualBatchType=c( \"TSS\", \"ShipDate\",\"BCR\",\"PlateID\" ,\"CGCCandGSC\",\"BatchID\"),\n";

print BVEV " theDSCPermutations=100,\n";

print BVEV " theMinBatchSize=5)\n";

print BVEV "myBatchCorrObject <- new(\"BatchCorr\",\n";

print BVEV " theMinNumberOfGenes=500,\n";

print BVEV " theNumberOfPermutatedGenes=500,\n";

print BVEV " theNumberOfPermutations=100,\n";

print BVEV " theMinBatchSize=5,\n";

print BVEV " theAdjustedFlag=TRUE,\n";

print BVEV " theNumberOfThreads=1,\n";

print BVEV " theSeed=0,\n";

print BVEV " theDoMtoMFlag=FALSE,\n";

print BVEV " theDoOtoMFlag=FALSE)\n";

print BVEV "myEB_withPriors <- new (\"Corrections_EB\",\n";

print BVEV " theEB_DoCorrectionFlag=FALSE,\n";

print BVEV " theEB_BatchIdsNotToCorrect=c(\"\"),\n";

print BVEV " theEB_DoCheckPlotsFlag=FALSE)\n";

print BVEV "myEB_withoutPriors <- new (\"Corrections_EB\",\n";

print BVEV " theEB_DoCorrectionFlag=FALSE,\n";

print BVEV " theEB_BatchIdsNotToCorrect=c(\"\"),\n";

print BVEV " theEB_DoCheckPlotsFlag=FALSE)\n";

print BVEV "myMP_Overall <- new (\"Corrections_MP\",\n";

print BVEV " theMP_DoCorrectionFlag=FALSE)\n";

print BVEV "myMP_ByBatch <- new (\"Corrections_MP\",\n";

print BVEV " theMP_DoCorrectionFlag=FALSE)\n";

print BVEV "myAN_Adjusted <- new (\"Corrections_AN\",\n";

print BVEV " theAN_DoCorrectionFlag=FALSE)\n";

print BVEV "myAN_Unadjusted <- new (\"Corrections_AN\",\n";

print BVEV " theAN_DoCorrectionFlag=FALSE)\n";

print BVEV "myCorrectionsObject <- new(\"Corrections_Setup\",\n";

print BVEV " theALL_MinBatchSize=5,\n";

print BVEV " theALL_BatchTypeToCorrect=\"*\",\n";

print BVEV " theALL_DoCorrectionOnlyFlag=FALSE,\n";

print BVEV " theEB_withPriors=myEB_withPriors,\n";

print BVEV " theEB_withoutPriors=myEB_withoutPriors,\n";

print BVEV " theMP_Overall=myMP_Overall,\n";

print BVEV " theMP_ByBatch=myMP_ByBatch,\n";

print BVEV " theAN_Adjusted=myAN_Adjusted,\n";

print BVEV " theAN_Unadjusted=myAN_Unadjusted)\n";

print BVEV "doRunBEA_Files(myInputOutputObject,\n";

print BVEV " myDataFiltersObject,\n";

print BVEV " myHCObject,\n";

print BVEV " myPCAObject,\n";

print BVEV " myBatchCorrObject,\n";

print BVEV " myCorrectionsObject)\n\n";

print BVEV "###Batch Variable Evaluation using Pi-plot,dot-plot,3-dimention PCA et.al.###\n";

print BVEV "AddGrids3d <- function(x, y=NULL, z=NULL, grid = TRUE,\n";

print BVEV " col.grid = \"grey\", lty.grid = par(\"lty\"),\n";

print BVEV " lab = par(\"lab\"), lab.z = mean(lab[1:2]),\n";

print BVEV " scale.y = 1, angle = 40,\n";

print BVEV " xlim=NULL, ylim=NULL, zlim=NULL){\n";

print BVEV " if(inherits(x, c(\"matrix\", \"data.frame\"))){\n";

print BVEV " x <- as.data.frame(x)\n";

print BVEV " y <- unlist(x[,2])\n";

print BVEV " z <- unlist(x[,3])\n";

print BVEV " x <- unlist(x[,1])}\n";

print BVEV " p.lab <- par(\"lab\")\n";

print BVEV " angle <- (angle%%360)/90\n";

print BVEV " yz.f <- scale.y * abs(if (angle < 1) angle else if (angle >3) angle - 4 else 2 - angle)\n";

print BVEV " yx.f <- scale.y * (if (angle < 2) 1 - angle else angle - 3)\n";

print BVEV " \n # x axis range\n";

print BVEV " x.range <- range(x[is.finite(x)], xlim)\n";

print BVEV " x.prty <- pretty(x.range, n = lab[1], min.n = max(1, min(0.5 *lab[1], p.lab[1])))\n";

print BVEV " x.scal <- round(diff(x.prty[1:2]), digits = 12)\n";

print BVEV " x <- x/x.scal\n";

print BVEV " x.range <- range(x.prty)/x.scal\n";

print BVEV " x.max <- ceiling(x.range[2])\n";

print BVEV " x.min <- floor(x.range[1])\n";

print BVEV " if (!is.null(xlim)) {\n";

print BVEV " x.max <- max(x.max, ceiling(xlim[2]/x.scal))\n";

print BVEV " x.min <- min(x.min, floor(xlim[1]/x.scal))}\n";

print BVEV " x.range <- range(x.min, x.max)\n\n";

print BVEV " \n # y axis range\n";

print BVEV " y.range <- range(y[is.finite(y)], ylim)\n";

print BVEV " y.prty <- pretty(y.range, n = lab[2], min.n = max(1, min(0.5 *lab[2], p.lab[2])))\n";

print BVEV " y.scal <- round(diff(y.prty[1:2]), digits = 12)\n";

print BVEV " y.add <- min(y.prty)\n";

print BVEV " y <- (y - y.add)/y.scal\n";

print BVEV " y.max <- (max(y.prty) - y.add)/y.scal\n";

print BVEV " if (!is.null(ylim)) \n\ty.max <- max(y.max, ceiling((ylim[2] - y.add)/y.scal))\n";

print BVEV " \n # Z axis range\n";

print BVEV " z.range <- range(z[is.finite(z)], zlim)\n";

print BVEV " z.prty <- pretty(z.range, n = lab.z, min.n = max(1, min(0.5 *lab.z, p.lab[2])))\n";

print BVEV " z.scal <- round(diff(z.prty[1:2]), digits = 12)\n";

print BVEV " z <- z/z.scal\n";

print BVEV " z.range <- range(z.prty)/z.scal\n";

print BVEV " z.max <- ceiling(z.range[2])\n";

print BVEV " z.min <- floor(z.range[1])\n";

print BVEV " if (!is.null(zlim)) {\n";

print BVEV " z.max <- max(z.max, ceiling(zlim[2]/z.scal))\n";

print BVEV " z.min <- min(z.min, floor(zlim[1]/z.scal)) }\n";

print BVEV " z.range <- range(z.min, z.max)\n\n";

print BVEV " \n #Add grid\n";

print BVEV " if (\"xy\" \%in% grid || grid == TRUE) {\n";

print BVEV " i <- x.min:x.max\n";

print BVEV " segments(i, z.min, i + (yx.f * y.max), yz.f * y.max +\n";

print BVEV " z.min, col = col.grid, lty = lty.grid)\n";

print BVEV " i <- 0:y.max\n";

print BVEV " segments(x.min + (i * yx.f), i * yz.f + z.min, x.max + \n";

print BVEV " (i * yx.f), i * yz.f + z.min, col = col.grid, lty = lty.grid) }\n\n";

print BVEV " if (\"xz\" \%in% grid) {\n";

print BVEV " i <- x.min:x.max\n";

print BVEV " segments(i + (yx.f * y.max), yz.f * y.max + z.min, \n";

print BVEV " i + (yx.f * y.max), yz.f * y.max + z.max, \n";

print BVEV " col = col.grid, lty = lty.grid)\n";

print BVEV " temp <- yx.f * y.max\n";

print BVEV " temp1 <- yz.f * y.max\n";

print BVEV " i <- z.min:z.max\n";

print BVEV " segments(x.min + temp,temp1 + i, \n";

print BVEV " x.max + temp,temp1 + i , col = col.grid, lty = lty.grid) }\n\n";

print BVEV " if (\"yz\" \%in% grid) {\n";

print BVEV " i <- 0:y.max\n";

print BVEV " segments(x.min + (i * yx.f), i * yz.f + z.min, \n";

print BVEV " x.min + (i * yx.f) ,i * yz.f + z.max, \n";

print BVEV " col = col.grid, lty = lty.grid)\n";

print BVEV " temp <- yx.f * y.max\n";

print BVEV " temp1 <- yz.f * y.max\n";

print BVEV " i <- z.min:z.max\n";

print BVEV " segments(x.min + temp,temp1 + i, \n";

print BVEV " x.min, i , col = col.grid, lty = lty.grid) }\n\n";

print BVEV "}\n\n";

print BVEV "setwd(\"$BatchInfoAndMatrixDir\")\n";

print BVEV "library(\"RColorBrewer\")\n";

print BVEV "library(\"scatterplot3d\")\n";

if(($project eq 'GDC-GEO')||($project eq 'GEO')){

$ExpSubmatrix =~ s/\.gz//;

`gunzip -c $ExpSubmatrix.gz > $ExpSubmatrix`;

}

print BVEV "countData=read.table(\"$ExpSubmatrix\",sep=\"\\t\",header=T)\n";

print BVEV "genes<-countData\$gene_id\n";

print BVEV "countData <-countData[,c(1,2:$TotalSizePlus1)]\n";

print BVEV "countData <- as.matrix(countData[c(-0,-1)])\n";

print BVEV "rownames(countData) <- genes\n";

print BVEV "cancer_samp_size = $TumorSampleSize\n";

print BVEV "normal_samp_size = $NormalSampleSize\n";

print BVEV "condition <- factor(c(rep(\"Cancer\",cancer_samp_size),rep(\"Normal\",normal_samp_size)))\n";

print BVEV "###PCA calculation ###\n";

if(($project eq 'GDC-GEO')||($project eq 'GEO')){

print BVEV "project.pca <- prcomp(t(countData))\n";

}else{

print BVEV "countData_log2 <-log(as.matrix(countData) +1)\n";

print BVEV "project.pca <- prcomp(t(countData_log2))\n";

}

print BVEV "summary(project.pca)\n";

print BVEV "project.pca.proportionvariances <- ((project.pca\$sdev^2) / (sum(project.pca\$sdev^2)))*100\n";

for my $BatchType (keys %BatchTypeLocation){

my $BaseTitle = "2018_7_26_1729\_$cancer\_$analyte\_$dataset\_$BatchType";

my $OurEvalOutDir = "$MBatch_input_dir/OurEvalOutDir";

main::mkpath "$OurEvalOutDir/$BatchType" unless (-d "$OurEvalOutDir/$BatchType");

print BVEV "BatchInfo_$BatchType <- read.table(\"$MBatch_input_dir/bea_batch_$BatchType.tsv\",sep=\"\\t\",header=T)\n";

print BVEV "Batchs <- factor(BatchInfo_$BatchType\$batch)\n";

print BVEV "FactorsToColors <- function(A){\n";

print BVEV " QualColors = c(\"#000000\",\"#E62000\",\"#B805FF\",\"#6A5ACD\",\"#DDF000\",\"#303745\",\n";

print BVEV " \"#EB8E55\",\"#4169E1\",\"#00FFFF\",\"#802A2A\",\"#FF0000\",\"#8A2BE2\",\n";

print BVEV " \"#008B45\",\"#385E0F\",\"#082E54\",\"#3A0085\",\"#7FFF00\",\"#B03060\",\n";

print BVEV " \"#008B8B\",\"#FF7D40\",\"#8A360F\",\"#CDB38B\",\"#0965EC\",\"#708069\",\n";

print BVEV " \"#5C0515\",\"#7D5021\",\"#FFB83D\",\"#FF3D49\",\"#DA70D6\",\"#00C957\",\n";

print BVEV " \"#7d7921\",\"#020450\",\"#FE7b82\",\"#F50041\",\"#0706EA\",\"#09ec64\")\n";

print BVEV " colors = QualColors[1:length(levels(A))]\n";

print BVEV " mapp = matrix(c(as.character(levels(A)),as.character(colors)),ncol=2)\n";

print BVEV " A = as.character(A)\n";

print BVEV " for(i in 1:length(A)){\n";

print BVEV " if (A[i] \%in% mapp[,1]) { \n";

print BVEV " A[i]=mapp[which(mapp[,1]==A[i]),2]\n";

print BVEV " }\n\t}\n";

print BVEV " list(colors,A)\n";

print BVEV "}\n";

print BVEV "color_Batchs <- FactorsToColors(Batchs)[[2]]\n";

print BVEV "color_legend <- FactorsToColors(Batchs)[[1]]\n";

print BVEV "##screen plot##\npdf(\"$OurEvalOutDir/$BatchType/$BaseTitle\_ScreenPlot.pdf\",width=12,height=12)\n";

print BVEV "barplot(project.pca.proportionvariances, cex.names=1, xlab=paste(\"Principal component (PC), 1-\",\n";

print BVEV " length(project.pca\$sdev)), ylab=\"Proportion of variation (%)\",\n";

print BVEV " main=\"$BaseTitle\_ScreenPlot\", ylim=c(0,100))\n";

print BVEV "dev.off()\n";

print BVEV "##Pairs plot##\npdf(\"$OurEvalOutDir/$BatchType/$BaseTitle\_PairsPlot.pdf\",width=12,height=12)\n";

print BVEV "par(cex=1.0, cex.axis=0.8, cex.main=0.8)\n";

print BVEV "pairs(project.pca\$x[,1:10], col=\"black\",main=\"$BaseTitle\_Pairs-plot\\nPCs 1-10\", pch=16)\n";

print BVEV "dev.off()\n";

print BVEV "##Bi plot##\npdf(\"$OurEvalOutDir/$BatchType/$BaseTitle\_BiPlot_Pc1vsPc2.pdf\",width=12,height=12)\n";

print BVEV "plot(project.pca\$x, type=\"n\",\n";

print BVEV " main=\"$BaseTitle\_BiPlot_Pc1vsPc2\", xlab=paste(\"PC1, \",\n";

print BVEV " round(project.pca.proportionvariances[1], 2), \"%\"), ylab=paste(\"PC2, \",\n";

print BVEV " round(project.pca.proportionvariances[2], 2), \"%\"))\n";

print BVEV "points(project.pca\$x, col=color_Batchs, pch=16, cex=1)\n";

print BVEV "legend(\"topleft\",levels(Batchs),pch=c(15,15),col=color_legend,cex=0.5,xpd=TRUE,ncol=2)\n";

print BVEV "dev.off()\n";

print BVEV "##Bi plot##\npdf(\"$OurEvalOutDir/$BatchType/$BaseTitle\_BiPlot_Pc1vsPc3.pdf\",width=12,height=12)\n";

print BVEV "plot(project.pca\$x[,1],project.pca\$x[,3],type=\"n\",\n";

print BVEV " main=\"$BaseTitle\_BiPlot_Pc1vsPc3\", xlab=paste(\"PC1, \",\n";

print BVEV " round(project.pca.proportionvariances[1], 2), \"%\"), ylab=paste(\"PC3, \",\n";

print BVEV " round(project.pca.proportionvariances[3], 2), \"%\"))\n";

print BVEV "points(project.pca\$x[,1],project.pca\$x[,3], col=color_Batchs, pch=16, cex=1)\n";

print BVEV "legend(\"topleft\",levels(Batchs),pch=c(15,15),col=color_legend,cex=0.5,xpd=TRUE,ncol=2)\n";

print BVEV "dev.off()\n";

print BVEV "##Bi plot##\npdf(\"$OurEvalOutDir/$BatchType/$BaseTitle\_BiPlot_Pc2vsPc3.pdf\",width=12,height=12)\n";

print BVEV "plot(project.pca\$x[,2],project.pca\$x[,3],type=\"n\",\n";

print BVEV " main=\"$BaseTitle\_BiPlot_Pc2vsPc3\", xlab=paste(\"PC2, \",\n";

print BVEV " round(project.pca.proportionvariances[2], 2), \"%\"), ylab=paste(\"PC3, \",\n";

print BVEV " round(project.pca.proportionvariances[3], 2), \"%\"))\n";

print BVEV "points(project.pca\$x[,1],project.pca\$x[,3], col=color_Batchs, pch=16, cex=1)\n";

print BVEV "legend(\"topleft\",levels(Batchs),pch=c(15,15),col=color_legend,cex=0.5,xpd=TRUE,ncol=2)\n";

print BVEV "dev.off()\n";

print BVEV "#Tri-plot#\npdf(\"$OurEvalOutDir/$BatchType/$BaseTitle\_3DiemsionPCAPlot.pdf\",width=12,height=12)\n";

print BVEV "par(mar=c(4,4,4,4), cex=1.0, cex.main=0.8, cex.axis=0.8)\n";

print BVEV "scatterplot3d(project.pca\$x[,1:3], main=\"\", color=color_Batchs, pch=16,\n";

print BVEV " xlab=paste(\"PC1, \", round(project.pca.proportionvariances[1], 2), \"%\"),\n";

print BVEV " ylab=paste(\"PC2, \", round(project.pca.proportionvariances[2], 2), \"%\"),\n";

print BVEV " zlab=paste(\"PC3, \", round(project.pca.proportionvariances[3], 2), \"%\"),grid=T,box=T)\n";

print BVEV "par(new=T)\n";

print BVEV "AddGrids3d(project.pca\$x[,1:3], grid = c(\"xy\", \"xz\", \"yz\"))\n";

print BVEV "par(new=T)\n";

print BVEV "scatterplot3d(project.pca\$x[,1:3], main=\"$BaseTitle\_3DimensionPCAPlot\",\n";

print BVEV " color=color_Batchs, pch=16,\n";

print BVEV " xlab=paste(\"PC1, \", round(project.pca.proportionvariances[1], 2), \"%\"),\n";

print BVEV " ylab=paste(\"PC2, \", round(project.pca.proportionvariances[2], 2), \"%\"),\n";

print BVEV " zlab=paste(\"PC3, \", round(project.pca.proportionvariances[3], 2), \"%\"),grid=T)\n";

print BVEV "legend(\"topleft\",levels(Batchs),pch=c(16,16),col=color_legend,cex=0.5,xpd=TRUE,ncol=2)\n";

print BVEV "dev.off()\n\n";

}

close BVEV;

`Rscript ./$cancer\_$project\_BatchVariableEvaluation.R`;

if(($project eq 'GDC-GEO')||($project eq 'GEO')){

`rm $ExpSubmatrix` if(-e $ExpSubmatrix);

}

`rm ./$cancer\_$project\_BatchVariableEvaluation.R`;

`rm $MBatch_input_dir/bea_input_cleansed.tsv` if(-e "$MBatch_input_dir/bea_input_cleansed.tsv");

Print "Project $project $analyte $cancer evaluated.\n";

}

Print "All Successful\n";

}

iv) The code (Perl language) used to perform expression quantitative trait methylation (eQTM) and expression quantitative trait copy number alterations (eQTCN) analysis:

#!/usr/bin/env perl -w

use strict;

use warnings;

use Storable;

use Data::Dumper;

=head1

This script was to perform eQTM and eQTCN analysis

The script reflect the pipline of the two types of analysis

=cut

unless(-e "./Results/Dumper_KIRC_EMTgenes_mQTLs_FDR05"){

unless(-e "./Results/Dumper_KIRC_EMTgenes_mQTLs"){

open GLIST,"./Results/EMT_related_gene_ByLiterature.txt" or die $!;

my $EMT_genes;

while(<GLIST>){

chomp;

push @$EMT_genes,$_;

}

close GLIST;

my $info_sub1;

if(-e "./Results/Dumper_meth_data_subset1"){

$info_sub1 = retrieve("./Results/Dumper_meth_data_subset1");

}else{

print "Preparing meth data subset1...\n";

open SUB1,"KIRC_CpG_MValues.DEG.CancerVsNormal.DMPs.MultiPkgs.Subset1.txt" or die $!;

while(<SUB1>){

chomp;

next if($.==1);

my @line = split / /;

my ($cpg,$genes,$logfc,$qval) = ($line[0],$line[13],$line[23],$line[27]);

my @genes_uniq;

if($genes=~/;/){

@genes_uniq = split /;/,$genes;

my %hash;

@genes_uniq = grep { ++$hash{$_} < 2 } @genes_uniq;

}else{@genes_uniq = ($genes)}

for my $gene (@$EMT_genes){

if((grep {$_ eq uc($gene)} @genes_uniq)&&($qval<=0.01)){

push @{$info_sub1->{$gene}->{'CpGs'}},$cpg;

push @{$info_sub1->{$gene}->{'LogFCs'}},$logfc;

}

}

}

close SUB1;

main::store $info_sub1,"./Results/Dumper_meth_data_subset1";

}

my $info_sub2;

if(-e "./Results/Dumper_meth_data_subset2"){

$info_sub2 = retrieve("./Results/Dumper_meth_data_subset2");

}else{

print "Preparing meth data subset2...\n";

open SUB2,"KIRC_CpG_MValues.DEG.CancerVsNormal.DMPs.MultiPkgs.Subset2.txt" or die $!;

while(<SUB2>){

chomp;

next if($.==1);

my @line = split / /;

my ($cpg,$genes,$logfc,$qval) = ($line[0],$line[13],$line[23],$line[27]);

my @genes_uniq;

if($genes=~/;/){

@genes_uniq = split /;/,$genes;

my %hash;

@genes_uniq = grep { ++$hash{$_} < 2 } @genes_uniq;

}else{@genes_uniq = ($genes)}

for my $gene (@$EMT_genes){

if((grep {$_ eq uc($gene)} @genes_uniq)&&($qval<=0.01)){

push @{$info_sub2->{$gene}->{'CpGs'}},$cpg;

push @{$info_sub2->{$gene}->{'LogFCs'}},$logfc;

}

}

}

close SUB2;

main::store $info_sub2,"./Results/Dumper_meth_data_subset2";

}

my $info_signGenes;

if(-e "./Results/Dumper_KIRC_SMGs_info"){

$info_signGenes = main::retrieve("./Results/Dumper_KIRC_SMGs_info");

}else{

print "Detecting significant DMGs...\n";

for my $gene (@$EMT_genes){

next unless((exists $info_sub1->{$gene}->{'CpGs'})&&(exists $info_sub2->{$gene}->{'CpGs'}));

my @cpgs_sub1 = @{$info_sub1->{$gene}->{'CpGs'}};

my @logfcs = @{$info_sub1->{$gene}->{'LogFCs'}};

next unless(exists $info_sub2->{$gene}->{'CpGs'});

my @cpgs_sub2 = @{$info_sub2->{$gene}->{'CpGs'}};

for my $num (0..$#cpgs_sub1){

if(grep {$cpgs_sub1[$num] eq $_} @cpgs_sub2){

push @{$info_signGenes->{$gene}->{'CpGs'}},$cpgs_sub1[$num];

push @{$info_signGenes->{$gene}->{'LogFCs'}},$logfcs[$num];

}

}

}

store $info_signGenes,"./Results/Dumper_KIRC_SMGs_info";

}

my @expr_samples = split /\n/,`less KIRC_rnaseq_BatchInfo.txt|grep TCGA|cut -f1`;

my @meth_samples = split /\n/,`less KIRC_methylation_BatchInfo.txt|grep TCGA|cut -f1`;

print "Detecting Common patients of expr and meth data...\n";

my @common_patients;

for my $sample(@expr_samples){

my $patient = $1 if($sample=~/(TCGA-.+-.+-.+)-.+-.+-.+/);#e.g. (TCGA-AK-3460-01A)-02D-1275-05

if(grep {$_=~/$patient/} @meth_samples){

push @common_patients,$patient unless(grep {$patient eq $_} @common_patients);

}

}

my $s = @common_patients;

print "Common patients: $s\n";

print "Detecting loci of common patients in expr and meth data...\n";

my (@loc_common_patients_expr,@loc_common_patients_meth);

my @chains = split /\t/,`less KIRC_gene_log2CPM_expression.txt|head -1`;

my @chains2 =split /\t/,`less KIRC_CpG_MValues_QuantileNormalized|head -1`;

for my $pat(@common_patients){

for my $loc(0..$#chains){

if($chains[$loc]=~/$pat/){

push @loc_common_patients_expr,$loc+1;

last;

}

}

for my $loc(0..$#chains2){

if($chains2[$loc]=~/$pat/){

push @loc_common_patients_meth,$loc;

last;

}

}

}

my $anno_info;

if(-e "./Results/Dumper_symbol2EMBL"){

$anno_info = retrieve("./Results/Dumper_symbol2EMBL");

}else{

my $gene_anno_file = "gencode.v22.annotation.gene.ProbeMap";

open ANNO,"$gene_anno_file" or die $!;

while(<ANNO>){

chomp;

my @line = split /\s+/;

$line[0]=~s/\.\d+//g;

$anno_info->{$line[2]}->{'EMBLGene'} = $line[0];

$anno_info->{$line[2]}->{'Type'} = $line[3];

}

close ANNO;

store $anno_info,"./Results/Dumper_symbol2EMBL";

}

unless(-e "KIRC_gene_log2CPM_expression_selected.txt"){

print "Selecting expr data subset..\n";

open EXPR,"KIRC_gene_log2CPM_expression.txt" or die $!;

open EXPRO,">KIRC_gene_log2CPM_expression_selected.txt" or die $!;

while(<EXPR>){

chomp;

if($.==1){

print EXPRO "Gene\t";

my $samples = join "\t",@common_patients;

print EXPRO "$samples\n";next;

}

my @line = split /\t/;

for my $gene (keys %$info_signGenes){

if($line[0] =~/$anno_info->{$gene}->{'EMBLGene'}/){

print EXPRO "$gene\t";

my @out;

for my $loc(0..$#line){

if(grep {$loc eq $_} @loc_common_patients_expr){

push @out,$line[$loc];

}

}

my $str =join "\t",@out;

print EXPRO "$str\n";

last;

}

}

}

close EXPR;

close EXPRO;

}

unless(-e "KIRC_CpG_MValues_QuantileNormalized_selected.txt"){

print "Selecting meth data subset..\n";

open METH,"KIRC_CpG_MValues_QuantileNormalized" or die $!;

open METHO,">KIRC_CpG_MValues_QuantileNormalized_selected.txt" or die $!;

my @array;

push @array,@{$info_signGenes->{$_}->{'CpGs'}} for(keys %$info_signGenes);

while(<METH>){

chomp;

if($.==1){

print METHO "CpG\t";

my $samples = join "\t",@common_patients;

print METHO "$samples\n";next;

}

my @line = split /\t/;

if(grep {$line[0] =~/$_/} @array){

print METHO "$line[0]\t";

my @out;

for my $loc(0..$#line){

if(grep {$loc eq $_} @loc_common_patients_meth){

push @out,$line[$loc];

}

}

my $str =join "\t",@out;

print METHO "$str\n";

}

}

close METH;

close METHO;

}

my $mir_symbol2alias = main::retrieve("./Results/Dumper_miRNA_symbol2alias");

unless(-e "KIRC_mirna_log2CPM_selected.txt"){

print "Selecting mirna expr data..\n";

@chains = split /\t/,`less KIRC_mirna_log2CPM.txt|head -1`;

my @loc_common_patients_mir;

for my $pat(@common_patients){

for my $loc(0..$#chains){

if($chains[$loc]=~/$pat/){

push @loc_common_patients_mir,$loc+1;

last;

}

}

}

open MIR,"./KIRC_mirna_log2CPM.txt" or die $!;

my @mirs;

for my $gene(keys %$info_signGenes){

push @mirs,$gene if(grep {$gene eq $_} keys %$mir_symbol2alias);

}

open MIRO,">./KIRC_mirna_log2CPM_selected.txt" or die $!;

while(<MIR>){

chomp;

if($.==1){

print MIRO "Gene\t";

my $samples = join "\t",@common_patients;

print MIRO "$samples\n";next;

}

my @line = split /\t/;

for my $mir(@mirs){

if($line[0]=~/$mir_symbol2alias->{$mir}$/){

print MIRO "$mir\t";

my @out;

for my $loc(0..$#line){

if(grep {$loc eq $_} @loc_common_patients_mir){

push @out,$line[$loc];

}

}

my $str =join "\t",@out;

print MIRO "$str\n";

last;

}

}

}

close MIR;

close MIRO;

}

my $KIRC_EMTgenes_mQTLs;

for my $gene(keys %$info_signGenes){

if($anno_info->{$gene}->{'Type'}=~/mirna/){

my @cpgs = @{$info_signGenes->{$gene}->{'CpGs'}};

chomp(my $expr_gene = `less KIRC_mirna_log2CPM_selected.txt|awk '{if(\$1 == "$gene") print}'`);

my @vals_gene = split /\s+/,$expr_gene;

for my $num(0..$#cpgs){

chomp(my $expr_cpg = `less KIRC_CpG_MValues_QuantileNormalized_selected.txt|grep $cpgs[$num]`);

my @vals_cpg = split /\s+/,$expr_cpg;

my $corr = &PearsonCorr(\@vals_gene,\@vals_cpg);

push @{$KIRC_EMTgenes_mQTLs->{$gene}->{'mQTLsPval'}},$corr->[0];

push @{$KIRC_EMTgenes_mQTLs->{$gene}->{'mQTLs'}},$cpgs[$num];

push @{$KIRC_EMTgenes_mQTLs->{$gene}->{'mQTLsCoef'}},$corr->[1];

push @{$KIRC_EMTgenes_mQTLs->{$gene}->{'DMG_LogFC'}},$info_signGenes->{$gene}->{'LogFCs'}->[$num];

}

}else{

next unless(`less KIRC_gene_log2CPM_expression_selected.txt|grep $gene`);

my @cpgs = @{$info_signGenes->{$gene}->{'CpGs'}};

chomp(my $expr_gene = `less KIRC_gene_log2CPM_expression_selected.txt|awk '{if(\$1 == "$gene") print}'`);

my @vals_gene = split /\s+/,$expr_gene;

next if(@vals_gene <300);

for my $num(0..$#cpgs){

print "testing $cpgs[$num] $vals_gene[0]\n";

chomp(my $expr_cpg = `less KIRC_CpG_MValues_QuantileNormalized_selected.txt|grep $cpgs[$num]`);

my @vals_cpg = split /\s+/,$expr_cpg;

my $corr = &PearsonCorr(\@vals_gene,\@vals_cpg);

push @{$KIRC_EMTgenes_mQTLs->{$gene}->{'mQTLsPval'}},$corr->[0];

push @{$KIRC_EMTgenes_mQTLs->{$gene}->{'mQTLs'}},$cpgs[$num];

push @{$KIRC_EMTgenes_mQTLs->{$gene}->{'mQTLsCoef'}},$corr->[1];

push @{$KIRC_EMTgenes_mQTLs->{$gene}->{'DMG_LogFC'}},$info_signGenes->{$gene}->{'LogFCs'}->[$num];

}

}

}

store $KIRC_EMTgenes_mQTLs,"./Results/Dumper_KIRC_EMTgenes_mQTLs";

}

unless(-e "./Results/Dumper_KIRC_EMTgenes_mQTLs_Table_FDR05.txt"){

my $KIRC_EMTgenes_mQTLs = main::retrieve("./Results/Dumper_KIRC_EMTgenes_mQTLs");

open TAB,">./Results/Dumper_KIRC_EMTgenes_mQTLs_Table.txt" or die $!;

print TAB "mQTLs\tgene\tcoef\tlogFC\tpvalue\n";

for my $gene(keys %$KIRC_EMTgenes_mQTLs){

my @mqtls = @{$KIRC_EMTgenes_mQTLs->{$gene}->{'mQTLs'}};

my @coefs = @{$KIRC_EMTgenes_mQTLs->{$gene}->{'mQTLsCoef'}};

my @logfc = @{$KIRC_EMTgenes_mQTLs->{$gene}->{'DMG_LogFC'}};

my @pvals = @{$KIRC_EMTgenes_mQTLs->{$gene}->{'mQTLsPval'}};

for my $num(0..$#mqtls){

print TAB "$mqtls[$num]\t$gene\t$coefs[$num]\t$logfc[$num]\t$pvals[$num]\n";

}

}

close TAB;

open Rscript,">ttt.R" or die $!;

print Rscript "data = read.csv(\"./Results/Dumper_KIRC_EMTgenes_mQTLs_Table.txt\",header=T,sep=\"\\t\")\n";

print Rscript "data\$FDR = p.adjust(data\$pvalue,method=\"fdr\")\n";

print Rscript "data2 = data[order(data\$FDR),]\n";

print Rscript "data3 = subset(data2,FDR < 0.05)\n";

print Rscript "write.table(data2,\"./Results/Dumper_KIRC_EMTgenes_mQTLs_Table_FDRall.txt\",quote=F,sep=\"\\t\",row.names=F)\n";

print Rscript "write.table(data3,\"./Results/Dumper_KIRC_EMTgenes_mQTLs_Table_FDR05.txt\",quote=F,sep=\"\\t\",row.names=F)\n";

close Rscript;

`Rscript3.5.1 ttt.R`;

`rm ttt.R`;

}

my $KIRC_EMTgenes_mQTLs = main::retrieve("./Results/Dumper_KIRC_EMTgenes_mQTLs");

my @mQTLs_FDR05 = split /\n/,`less ./Results/Dumper_KIRC_EMTgenes_mQTLs_Table_FDR05.txt|cut -f1`;

my $KIRC_EMTgenes_mQTLs_FDR05;

for my $gene (keys %$KIRC_EMTgenes_mQTLs){

my @mqtls = @{$KIRC_EMTgenes_mQTLs->{$gene}->{'mQTLs'}};

my @coefs = @{$KIRC_EMTgenes_mQTLs->{$gene}->{'mQTLsCoef'}};

my @logfc = @{$KIRC_EMTgenes_mQTLs->{$gene}->{'DMG_LogFC'}};

my @pvals = @{$KIRC_EMTgenes_mQTLs->{$gene}->{'mQTLsPval'}};

for my $num(0..$#mqtls){

if(grep {$mqtls[$num] eq $_} @mQTLs_FDR05){

push @{$KIRC_EMTgenes_mQTLs_FDR05->{$gene}->{'mQTLsPval'}},$pvals[$num];

push @{$KIRC_EMTgenes_mQTLs_FDR05->{$gene}->{'mQTLs'}},$mqtls[$num];

push @{$KIRC_EMTgenes_mQTLs_FDR05->{$gene}->{'mQTLsCoef'}},$coefs[$num];

push @{$KIRC_EMTgenes_mQTLs_FDR05->{$gene}->{'DMG_LogFC'}},$logfc[$num];

}

}

}

store $KIRC_EMTgenes_mQTLs_FDR05,"./Results/Dumper_KIRC_EMTgenes_mQTLs_FDR05";

}

unless(-e "Results/KIRC_CNA_affected_expr_correlation.pval.txt"){

print "Process CNA affected expression genes...\n";

my $anno_info;

if(-e "./Results/Dumper_EMBL2symbol"){

$anno_info = retrieve("./Results/Dumper_EMBL2symbol");

}else{

my $gene_anno_file = "gencode.v22.annotation.gene.ProbeMap";

open ANNO,"$gene_anno_file" or die $!;

while(<ANNO>){

chomp;

my @line = split /\s+/;

$line[0]=~s/\.\d+//g;

$anno_info->{$line[0]} = $line[2];

}

close ANNO;

store $anno_info,"./Results/Dumper_EMBL2symbol";

}

my @candgenes = split /\n/,`less ./Results/CNVcandidates_genes.txt|cut -f2`;

my ($CNA_data,@CNA_header);

open CNA,"KIRC_GISTIC2.0/all_thresholded.by_genes.txt" or die $!;

while(<CNA>){

chomp;

my @line =split /\t/;

if($.==1){

for my $index(0..$#line){

$line[$index] = $1 if($line[$index]=~/(TCGA-.+-.+-.+)-.+-.+-.+/);

@CNA_header = @line;

}

next;

}

if(grep {$line[0] eq $_} @candgenes){

for my $key(2..$#CNA_header){

$CNA_data->{$line[0]}->{$CNA_header[$key]} = $line[$key];

}

}

}

close CNA;

my ($EXP_data,@EXP_header);

open EXP,"./KIRC_gene_log2CPM_expression.txt" or die $!;

while(<EXP>){

chomp;

my @line =split /\t/;

if($.==1){

for my $index(0..$#line){

$line[$index] = $1 if($line[$index]=~/(TCGA-.+-.+-.+)-.+-.+-.+/);

@EXP_header = @line;

}

next;

}

$line[0]=~s/\.\d+//g;

my $symbol = $anno_info->{$line[0]};

next if(!defined $symbol);

next unless(grep {$_ eq $symbol} @candgenes);

for my $key(0..$#EXP_header){

$EXP_data->{$symbol}->{$EXP_header[$key]} = $line[$key+1];

}

}

close EXP;

my $matchdata;

open OUT,">Results/KIRC_CNA_affected_expr_correlation.pval.txt" or die $!;

print OUT "Gene\tlogfc\tpvalue\tFDR\tInfo\tDel_NC\tAmp_NC\n";

my $count;

for my $gene(keys %$CNA_data){

for my $samp(keys %{$CNA_data->{$gene}}){

next unless(exists $EXP_data->{$gene}->{$samp});

if($CNA_data->{$gene}->{$samp} < 0){

push @{$matchdata->{$gene}->{'Del'}},$EXP_data->{$gene}->{$samp};

}elsif($CNA_data->{$gene}->{$samp} > 0){

push @{$matchdata->{$gene}->{'Amp'}},$EXP_data->{$gene}->{$samp};

}else{

push @{$matchdata->{$gene}->{'NC'}},$EXP_data->{$gene}->{$samp};

}

}

my (@res,$status);

@{$matchdata->{$gene}->{'NC'}} = () unless(exists $matchdata->{$gene}->{'NC'});

@{$matchdata->{$gene}->{'Amp'}} = () unless(exists $matchdata->{$gene}->{'Amp'});

@{$matchdata->{$gene}->{'Del'}} = () unless(exists $matchdata->{$gene}->{'Del'});

next if((@{$matchdata->{$gene}->{'Amp'}} == 0) && @{$matchdata->{$gene}->{'Del'}} == 0);

if(@{$matchdata->{$gene}->{'Del'}} >= @{$matchdata->{$gene}->{'Amp'}}){

my @array= @{$matchdata->{$gene}->{'NC'}};

push @array,@{$matchdata->{$gene}->{'Amp'}} if(@{$matchdata->{$gene}->{'Amp'}}>0);

@array = &dropnull(@array);

my $n1 = @{$matchdata->{$gene}->{'Del'}};

my $n2 = @{$matchdata->{$gene}->{'NC'}};

my $n3 = @{$matchdata->{$gene}->{'Amp'}};

$status = "DelvsNCAmp,Del:$n1,NC:$n2,Amp:$n3";

@res = &Rttest([$matchdata->{$gene}->{'Del'},\@array]);

}else{

my @array= @{$matchdata->{$gene}->{'NC'}};

push @array,@{$matchdata->{$gene}->{'Del'}} if(@{$matchdata->{$gene}->{'Del'}}>0);

@array = &dropnull(@array);

my $n1 = @{$matchdata->{$gene}->{'Del'}};

my $n2 = @{$matchdata->{$gene}->{'NC'}};

my $n3 = @{$matchdata->{$gene}->{'Amp'}};

$status = "DelNCvsAmp,Del:$n1,NC:$n2,Amp:$n3";

@res = &Rttest([\@array,$matchdata->{$gene}->{'Amp'}]);

}

$res[2] =~s/S/,/g;

$res[3] =~s/S/,/g;

print "$gene\t$status\n";

print OUT "$gene\t$res[0]\t$res[1]\t1.000\t$status\t$res[2]\t$res[3]\n";

}

open Rscript,">ttt.R" or die $!;

print Rscript "data = read.csv(\"./Results/KIRC_CNA_affected_expr_correlation.pval.txt\",header=T,sep=\"\\t\")\n";

print Rscript "data\$FDR = p.adjust(data\$pvalue,method=\"fdr\")\n";

print Rscript "data2 = data[order(data\$FDR),]\n";

print Rscript "data3 = subset(data2,FDR < 0.05)\n";

print Rscript "write.table(data2,\"./Results/KIRC_CNA_affected_expr_correlation.pval_FDRall.txt\",quote=F,sep=\"\\t\",row.names=F)\n";

print Rscript "write.table(data3,\"./Results/KIRC_CNA_affected_expr_correlation.pval_FDR05.txt\",quote=F,sep=\"\\t\",row.names=F)\n";

close Rscript;

`Rscript3.5.1 ttt.R`;

`rm ttt.R`;

sub dropnull {

my @arr = @_;

my @out;

for my $i(@arr){

push @out,$i if($i=~/\d/);

}

return @out;

}

sub Rttest {

my $argus = shift;

my $strlow = join "S",@{$argus->[0]};

my $strhig = join "S",@{$argus->[1]};

open RSC,">Rsc.R" or die $!;

print RSC "low = as.numeric(strsplit(\"$strlow\",\"S\")[[1]])\n";

print RSC "high = as.numeric(strsplit(\"$strhig\",\"S\")[[1]])\n";

print RSC "res = t.test(low,high)\n";

print RSC "logfc = as.numeric(res\$estimate[2] - res\$estimate[1])\n";

print RSC "write.table(c(logfc,res\$p.value),\"tmpres\",quote=F,row.names=F)\n";

close RSC;

`Rscript3.2.5 Rsc.R`;

chomp(my $logfc = `less tmpres|sed -n '2p'`);

chomp(my $pval = `less tmpres|sed -n '3p'`);

`rm tmpres Rsc.R`;

return ($logfc,$pval,$strlow,$strhig);

}

}

sub PearsonCorr {

my ($expr_gene,$expr_cpg) = (shift,shift);

open TMP,">tmpfile2" or die $!;

my $exp = join "\t",@$expr_gene;

print TMP "$exp\n";

my $exp2 = join "\t",@$expr_cpg;

print TMP "$exp2\n";

close TMP;

print "$expr_gene->[0]\n";

open RSC,">Rscript.R" or die $!;

print RSC "text = read.table(\"tmpfile2\",sep=\"\\t\",header=F)\n";

print RSC "x = cor.test(as.numeric(text[1,]),as.numeric(text[2,]))\n";

print RSC "res = as.vector(c(x\$p.value,as.numeric(x\$estimate)))\n";

print RSC "write.table(res,\"tmpfile3\",sep=\"\\t\",quote=F,row.names=F)";

close RSC;

`Rscript3.2.5 Rscript.R`;

chomp(my $pval = `less tmpfile3|sed -n '2p'`);

chomp(my $coef = `less tmpfile3|sed -n '3p'`);

`rm Rscript.R`;

`rm tmpfile2` if(-e "tmpfile2");

`rm tmpfile3` if(-e "tmpfile3");

my @res = ($pval,$coef);

return \@res;

}

sub Symbol2Alias {

my $list = shift;

open TMP,">tmpfile0" or die $!;

print TMP "symbol\n";

print TMP "$_\n" for(@$list);

close TMP;

open Rscript,">tmpRscript.R" or die $!;

print Rscript "MirSymbol2alias = function(alias){\n";

print Rscript " library(org.Hs.eg.db,quietly = TRUE)\n";

print Rscript " alias = as.character(alias)\n";

print Rscript " for (i in alias){\n";

print Rscript " alias = select(org.Hs.eg.db,i,\"ALIAS\",\"SYMBOL\")\$ALIAS\n";

print Rscript " if(length(grep(\"hsa-\",alias))>=1){\n";

print Rscript " alias = alias[grep(\"hsa-\",alias)[1]]\n";

print Rscript " }else{\n";

print Rscript " if(length(grep(\"mir-\",alias))>=1){\n";

print Rscript " alias = alias[grep(\"mir-\",alias)[1]]\n";

print Rscript " }else{\n";

print Rscript " alias = alias[grep(\"[a-z]\",alias,perl=T)[1]]\n";

print Rscript " }\n";

print Rscript " }\n";

print Rscript " }\nalias\n}\n";

print Rscript "array = read.table(\"./tmpfile0\",header=T)\n";

print Rscript "array\$ALIAS = apply(array,1,MirSymbol2alias)\n";

print Rscript "write.table(array,\"tmpfile1\",quote=F,row.names=F)\n";

close Rscript;

`Rscript3.5.1 tmpRscript.R`;

open SYMBOL,"tmpfile1" or die $!;

my %info;

while(<SYMBOL>){

chomp;

next if($.==1);

my @line = split /\s+/;

$info{$line[0]} = $line[1];

}

close SYMBOL;

`rm tmpfile0 tmpfile1 tmpRscript.R`;

return \%info;

}
